# Site-Selective C–H Sulfinamidation through Electron-Donor–Acceptor Complex Photoactivation and Radical Addition into Sulfinylamine Reagents

**DOI:** 10.1021/acs.orglett.5c03827

**Published:** 2025-10-14

**Authors:** Joshua T. Baxter, Adrian Hall, Michael C. Willis

**Affiliations:** † Chemistry Research Laboratory, Department of Chemistry, 6396 University of Oxford, Mansfield Road, Oxford OX1 3TA, U.K.; ‡ 439704 UCB Biopharma SPRL, Chemin du Foriest, Braine-L’Alleud, Brussels 1070 Belgium

## Abstract

Aryl sulfinamides are versatile synthetic intermediates for accessing diverse and medicinally relevant S­(VI) functionalities, such as sulfon­amides and sulfon­imid­amides. Herein, we report a thianthrenium-enabled, site-selective C–H sulfin­amidation procedure via a blue-light-promoted electron-donor–acceptor (EDA) complex to afford the key aryl sulfin­amide species. This operationally simple procedure combines site-selective C–H sulfonium salt preparation followed by single-electron transfer (SET) from a photoactive EDA complex formed with readily available amines. The resultant aryl radicals react with sulfinyl­amine reagents to deliver aryl sulfin­amides under mild conditions. The method displays broad generality and regio­selectively constructs key aryl sulfin­amides from medicinally relevant arenes without using expensive transition metal or photoredox catalysts, highlighting its use as a late-stage functionalization tool for medicinal and drug discovery chemistry.

## Graphical abstract

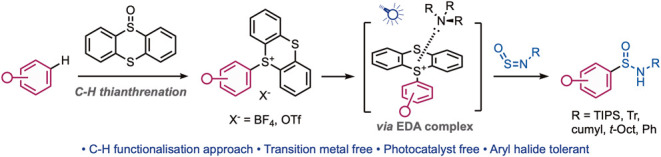

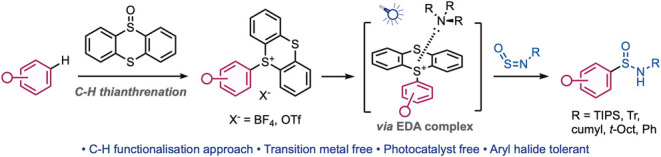

Aryl sulfinamides have found diverse uses in organic and medicinal chemistry. They are interesting final motifs for medicinal chemistry in their own right, finding use as amide bond bioisosteres and as treatments for leukemia.^1^ Enantiopure sulfin­amides have also found significant use as chiral ligands for transition metal catalysts and as auxiliaries in asymmetric transformations.^2^ Moreover, sulfin­amides are most often employed as intermediates for accessing related medicinally relevant S­(VI) functionalities, such as sulfon­amides, sulfon­imid­amides, and sulfon­imidoyl fluorides (Scheme 1A).^3^ Sulfon­amides make up the most prevalent class of S­(VI) bioactive molecules. Their mono-aza analogues, sulfon­imid­amides, are increasing in popularity in medicinal and agrochemical programs due to their potential for diversification at the additional nitrogen atom and the presence of a stereocenter at sulfur.^4^ Moreover, sulfon­imidoyl fluorides have also garnered increased attention as effective electrophiles in SuFEX applications to access alternative S­(VI) functionalities.^5^

**Scheme 1:**
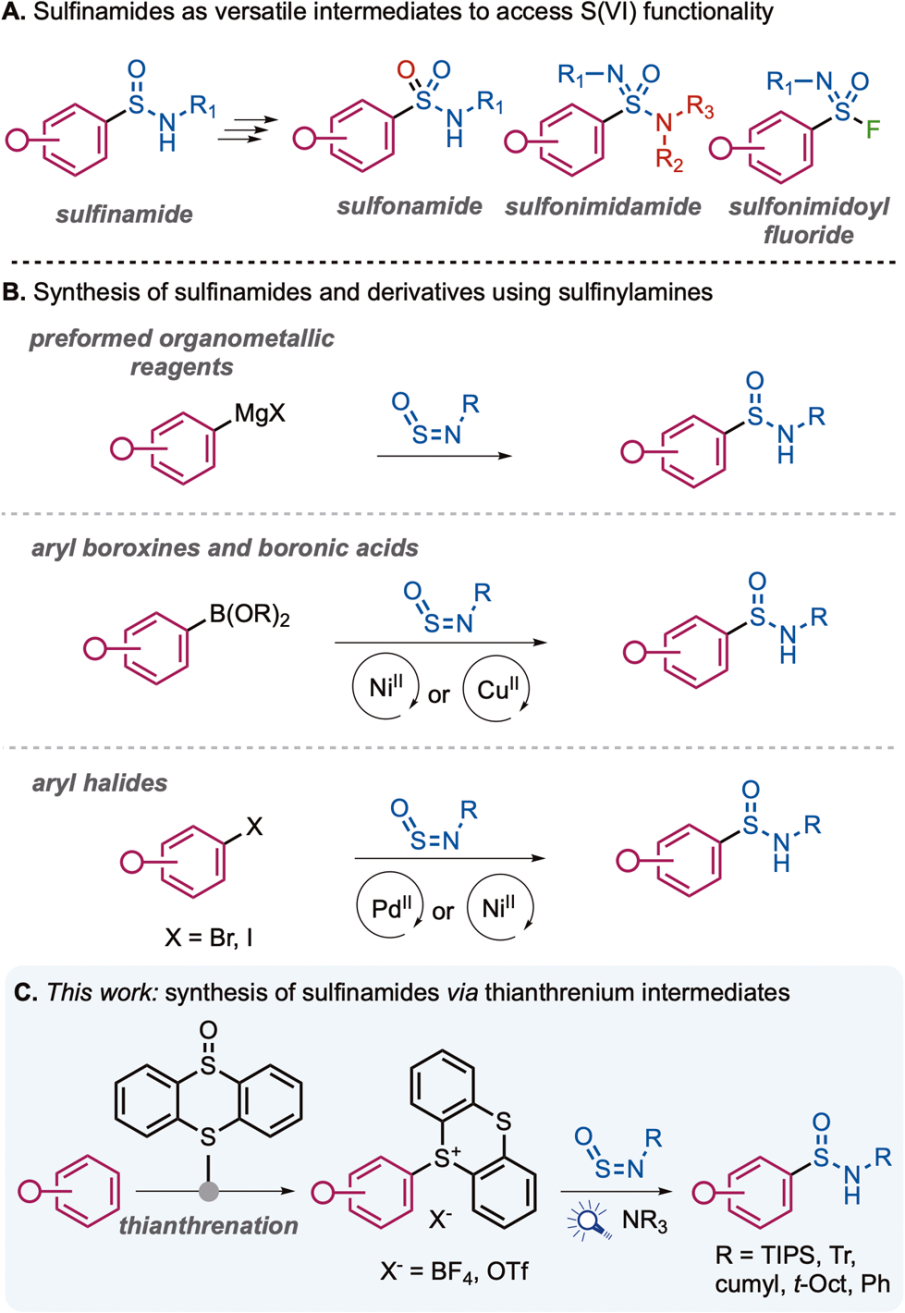
**A.** Uses of Aryl Sulfinamides; **B.** Previous Routes to Sulfinamides; **C.** *This Work*: EDA-Facilitated Synthesis of Sulfinamides from Thianthrenium Salts

Existing methods for aryl sulfin­amide synthesis typically employ manipulation of pre-installed sulfur centers such as thiols,^6^ disulfides,^7^ sulfenamides,^8^ sulfonyl chlorides,^9^ sulfinyl chlorides,^10^ and esters.^11^ Although effective, these methods often suffer from multistep procedures, the formation or utility of malodorous mercaptan compounds, harsh reaction conditions limiting functional group tolerance, and/or limited commercial availability of substrates. An alternative approach employs sulfinyl­amine reagents (R-NSO) as “linchpin” sulfur electrophiles,^12^ in combination with aryl partners such as Grignard reagents,^13^ aryl boroxines,^14^ or aryl halides,^15^ to forge the C–S bond (Scheme 1B). Recently, the groups of Shi and Zhang have also reported enantio­selective sulfin­amidation variants.^16^ These earlier approaches, albeit affording sulfin­amides in good yields, are limited by the use of costly precious metal catalysts and/or challenging substrates.

Designing a direct approach to aryl sulfin­amides that operates under mild reaction conditions, uses readily handled substrates and low cost or no catalyst, remains a challenge. Recently, the Ritter and Procter groups have demonstrated that aryl sulfonium salts under visible light irradiation serve as versatile aryl radical precursors for combination with varied electrophilic species.^17^ These bench-stable sulfonium salts can be generated from readily available arenes as well as complex, functionalized bioactive cores with exceptional regio­selectivity, underscoring their potential as powerful tools for late-stage functionalization,^18^ and they have been used to achieve varied transformations.^19^ We envisaged a mild photochemical approach to sulfin­amides whereby aryl radical species derived from sulfonium salts combine with the sulfinyl­amine reagents previously developed by this laboratory^20^ to afford sulfin­amides with enhanced functional group tolerance, employing bench-stable, easy-to-handle substrates (Scheme 1C).^21,200^

On the basis of our hypothesis, we selected aryl thianthrenium salt **1a** as a model substrate, employing DABCO as the donor in the electron-donor–acceptor (EDA) complex, following the success observed by Yu and co-workers in the photoactivation of arenes for construction of *N*-heterocycles.^22^ *N*-Triisopropylsilyl sulfinyl­amine (TIPS-NSO, **2a**) was chosen as the sulfinyl­amine reagent and DIPEA as the HAT source and reductant; selected optimization data is shown in Scheme 2 (see the Supporting Information for full details). To our delight, sulfin­amide **3a** was obtained in 45% yield when MeCN was used as the solvent and 427 nm light was used (entry 1). Optimization of the amine components revealed that higher yields were achieved when NEt_3_ was used in place of DIPEA (entries 1 and 2). Experiments excluding DABCO (entries 3 and 4) demonstrated that NEt_3_ could act as both EDA donor and HAT source with an improvement in yield to 57%. The Supporting Information provides details of further optimization studies, including the use of different bases, wavelengths of light, solvents, sulfonium salt forms and counterions, and reagent stoichiometry, along with UV–vis absorption data for EDA complex formation. Control experiments established that both NEt_3_ and light were essential for the formation of sulfin­amide **3a**, demonstrating the importance of the EDA complex formed between NEt_3_ and thianthrenium salt **1a** (entries 5 and 6). When the photocatalyst *fac*-Ir­(ppy)_3_ (2 mol%) was used in place of an EDA system, a slightly reduced yield of sulfin­amide **3a** was obtained (entry 7). Rather than optimize the catalyst-mediated system further, we decided to pursue the EDA method, attracted by the potential economic and environmental advantages offered by a (transition metal) catalyst-free system. The optimized reaction conditions involved using NEt_3_ (5.0 equiv) as EDA donor and HAT source and TIPS-NSO (2.0 equiv) in MeCN (0.1 M) at ambient temperature for 24 h under 427 nm irradiation, delivering sulfin­amide **3a** in 57% isolated yield (entry 4).

**Scheme 2:**
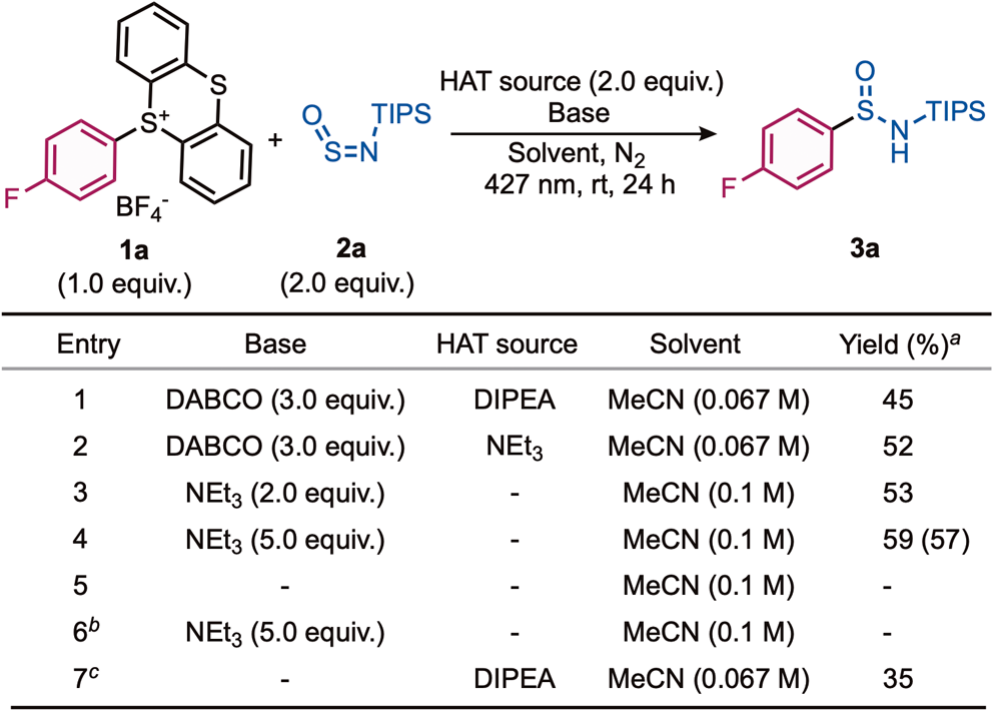
Investigation of the Reaction Conditions for the Synthesis of Sulfinamide **3a** ^a^

a Reactions conducted on a 0.1 mmol scale. Yields determined by quantitative ^19^F NMR spectroscopy of the crude reaction mixtures using α,α,α-trifluorotoluene as internal standard. Isolated yields in parentheses.
b Reaction performed in the dark.
c
*fac*-Ir­(ppy)_3_ (2 mol%) used as photocatalyst, 450 nm.

With optimized conditions in hand, we investigated the scope of the reaction with regard to the aryl thianthrenium salts, which were prepared directly from the relevant arenes (Scheme 3). Overall, a variety of *N*-TIPS-aryl sulfin­amides (**3a**–**s**) were obtained in moderate to good yields, with excellent functional group compatibility being demonstrated. Pleasingly, the reaction is tolerant to substitution at the *ortho-*, *meta-* and *para*-positions of the arene, with minimal variations in yield observed for the methyl- (**3b**), alkynyl- (**3f**), alkenyl- (**3k**), cyano- (**3l**), and ester- containing (**3m**) examples, further highlighting the good functional group compatibility of the reaction. A nitro substituent (**3e**) was also tolerated, which is often challenging under photochemical reaction conditions.^23^ Halides (**3d**) and (pseudo)­halides (**3c**) could also be transformed into the respective aryl sulfin­amides, providing orthogonal reactivity to classic transition metal catalysis or the use of pre-formed organometallic reagents. The synthesis of sulfin­amide **3d** could be scaled to 2 mmol, delivering 326 mg of the sulfin­amide in 36% yield. N-Containing heterocyclic sulfin­amides could also be constructed, evidenced by the quinoline (**3o**) and pyridine (**3p**) examples. Additionally, several biologically relevant sulfin­amides could be prepared, such as the flurbiprofen- (**3r**) and pyriproxyfen-derived (**3s**) analogues. Pleasingly, amides were also tolerated under the mild reaction conditions, including the base-sensitive trifluoro­acetamide group (**3h**) and, importantly for medicinal chemistry applications, a secondary amide (**3g**). Thianthrenium salts bearing electron-withdrawing substituents needed to be prepared from the corresponding boronic acids rather than the parent arene,^24^ but these substrates were also used successfully, affording the trifluoro­methyl (**3j**) and *p*-cyano (**3i**) sulfin­amides in 48% and 44% yields, respectively. Finally, alternative sulfinyl­amine reagents were also compatible with the developed method, with the *N*-cumyl-,^25^ *N*-*t*-octyl-, *N*-trityl-, and *N*-phenyl-substituted sulfin­amides obtained in comparable yields.

**Scheme 3:**
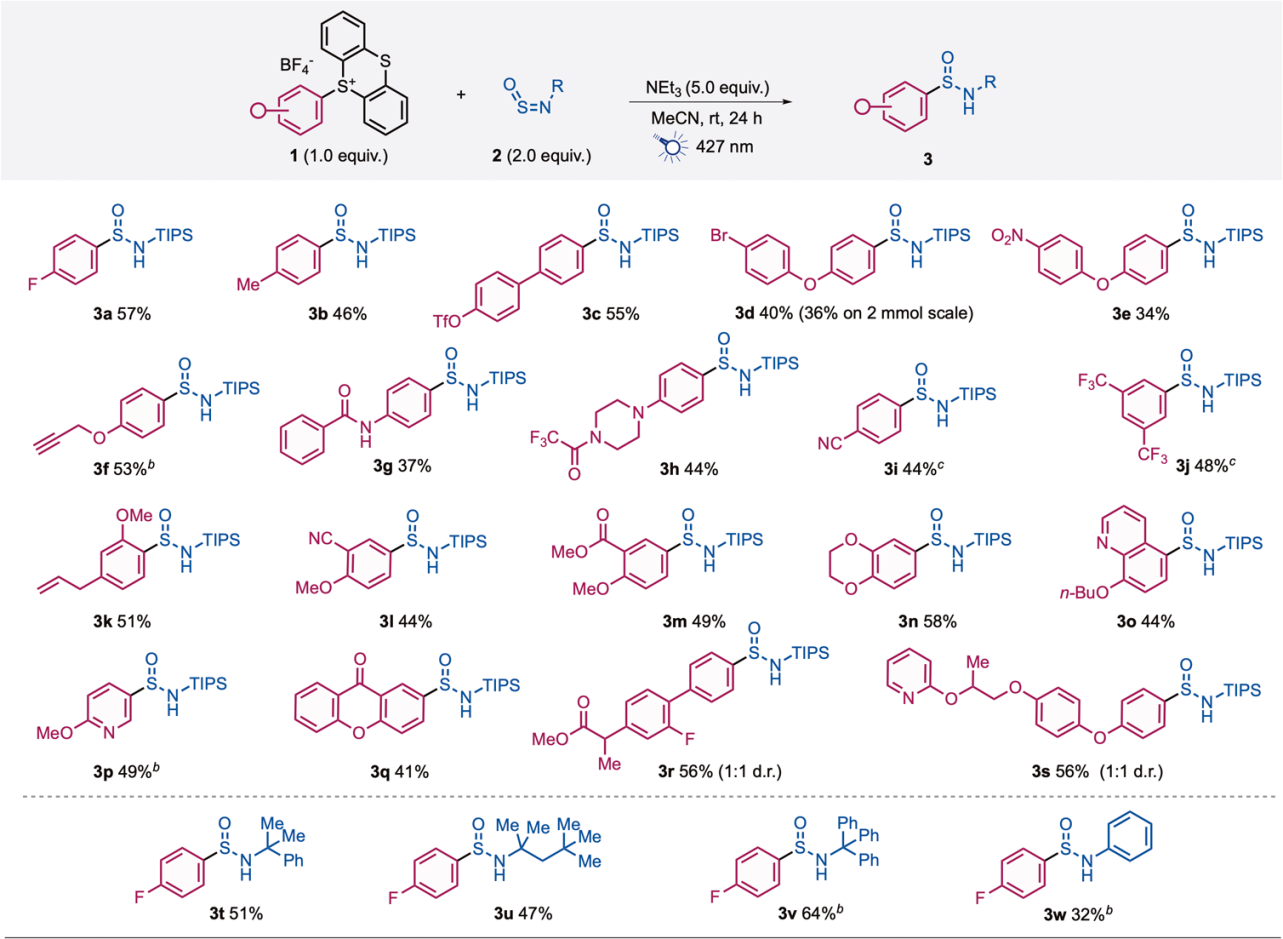
Scope of Aryl Sulfinamide Synthesis from Aryl Thianthrenium Salts and Sulfinylamine Reagents^a^

a Reaction conditions: tetrafluoroborate thianthrenium salt (0.2 mmol, 1.0 equiv), sulfinyl­amine (2.0 equiv), NEt_3_ (5.0 equiv), MeCN (0.1 M), rt, 24 h.
b 48 h instead of 24 h.
c Starting material prepared from the corresponding boronic acid.

A telescoped process was developed whereby sulfin­amides were obtained directly from the corresponding arene (Scheme 4). The arene was first transformed into the thianthrenium salt under electrophilic thianthrenation conditions using triflic anhydride as the activating species. After sulfonium salt formation, the excess triflic anhydride was quenched by addition of 2,6-lutidine. After a further 10 min stirring, NEt_3_ and TIPS-NSO were subsequently added, and the reaction mixture was stirred under blue light irradiation for 24 to 48 h. Using this approach, both the toluene-derived sulfin­amide **3b** and the more complex pyriproxyfen-derived sulfin­amide **3s** were obtained in reasonable yields.

**Scheme 4:**
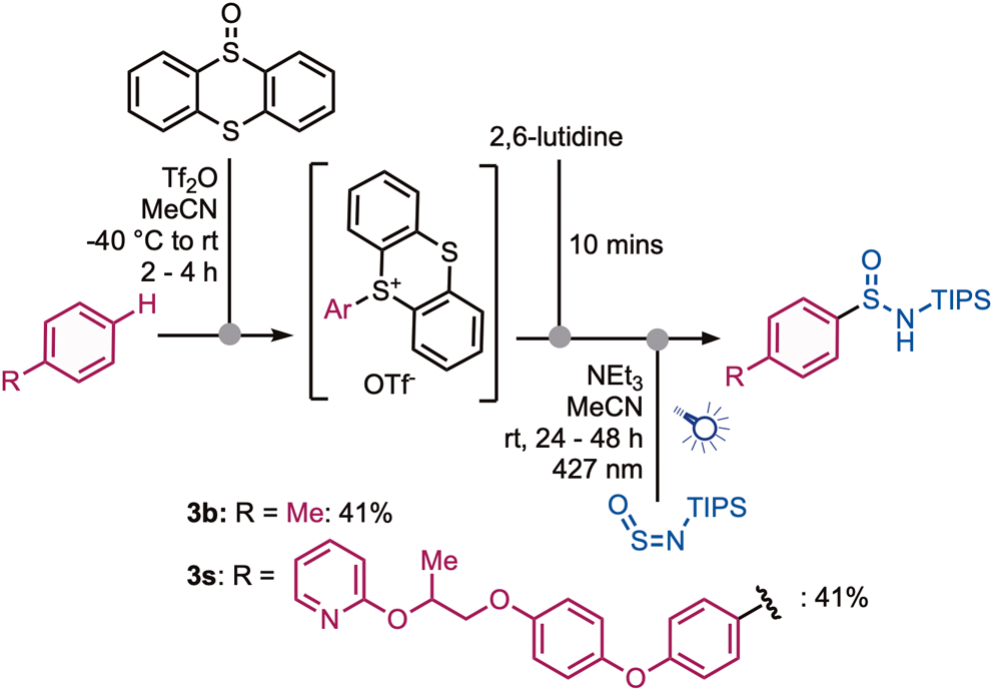
One-Pot C–H Sulfinamidation Sequence for the Synthesis of Sulfinamides **3b** and **3s**

With successful exploration of the scope of the reaction and expansion to a one-pot procedure achieved, we briefly explored functionalization of the aryl sulfin­amide products (Scheme 5). Sulfin­amide **3d** could be readily transformed into the deprotected sulfin­amide **4a**, sulfon­amide **4b**, sulfon­imid­amide **4c**, and sulfon­imidoyl fluoride **4d**, using fluoride deprotection, oxidation, oxidative amination, and oxidative fluorination conditions, respectively. The aryl bromide substituent present in sulfon­amide **4b** was utilized in a Suzuki–Miyaura coupling reaction, affording the C–C coupled product **4e** in 71% yield.

**Scheme 5:**
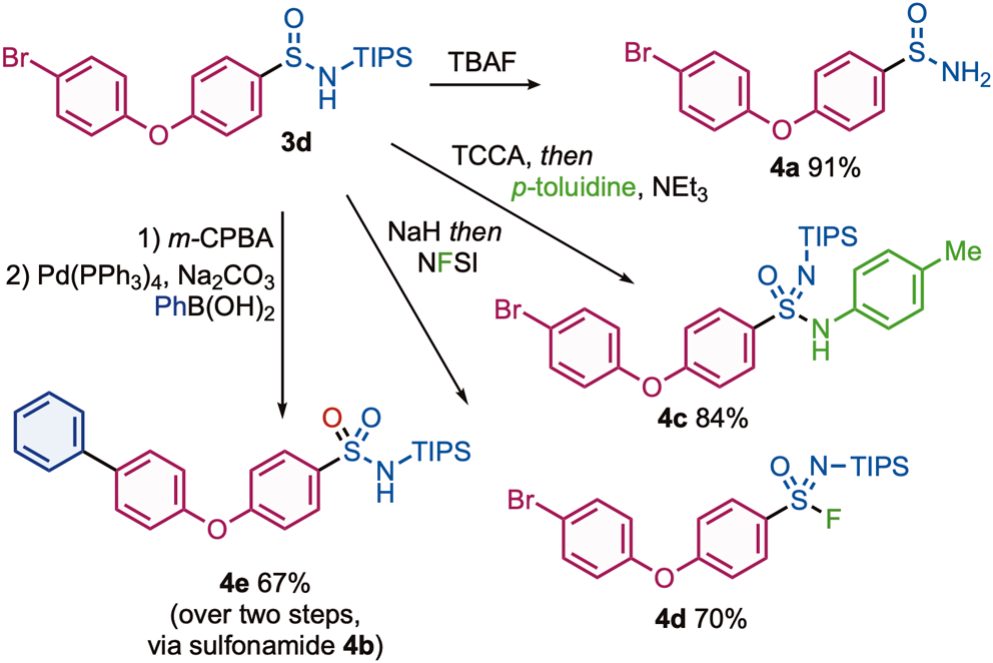
Functionalization of Sulfinamide **3d**

In summary, we have demonstrated that simple EDA complexes, generated from aryl sulfonium salts and triethyl­amine, can be transformed into aryl sulfin­amides using visible light and sulfinyl­amine reagents. The reactions are operationally straightforward and catalyst-free, use mild reaction conditions, display excellent functional group compatibility, and are broad in scope. The method can be adapted into a telescoped process to start from the corresponding C–H arene, highlighting its potential for use as a late-stage C–H functionalization tool for medicinal chemistry applications. The aryl sulfin­amide products can be utilized as a versatile platform to access related sulfur functionalities, such as sulfon­amides, sulfon­imid­amides, and sulfon­imidoyl fluorides.

## Supplementary Material



## Data Availability

The data underlying this study are available in the published article and its Supporting Information.

## Notes

Experimental procedures and supporting characterization data and spectra (PDF)
