# Supplementary material for: Site-Selective C–H Sulfinamidation through Electron-Donor–Acceptor Complex Photoactivation and Radical Addition into Sulfinylamine Reagents
Source: Org Lett. 2025 Oct 14;27(42):11901–5. doi: 10.1021/acs.orglett.5c03827 (PMC12560070; doi:10.1021/acs.orglett.5c03827)
Supplement: Supplementary file 1 [file ol5c03827_si_001.pdf]

Site-Selective C-H Sulfinamidation Through Electron-Donor-Acceptor Complex Photoactivation and Radical Addition into Sulfinylamine Reagents

Joshua T. Baxter,[a] Adrian Hall,[b] and Michael C. Willis[a,*]

[*] michael.willis@chem.ox.ac.uk

[a] Department of Chemistry, University of Oxford, Chemistry Research Laboratory, Mansfield Road, Oxford, OX1 3TA, UK

[b] UCB Biopharma SPRL, Chemin du Foriest, Braine-l'Alleud, Belgium

Supporting Information

1. General considerations **2**

1.1 Photochemical reaction set-up 3

2. Preparation of starting materials **4**

2.1 Preparation of thianthrenium salts 4

2.2 Preparation of sulfinylamines 10

3. Optimization of the reaction conditions **11**

3.1 Optimization of the dual amine EDA system 11

3.2 Optimization of single amine EDA system 16

3.3 Mass balance investigation 20

4. Experimental procedures **21**

4.1 General procedures 21

4.2 Sulfinamide substrate scope 21

4.3 Telescoped one-pot sulfinamidation procedures for sulfinamides **3b** and **3s** 34

4.4 Scale up procedure 35

4.5 Derivatisation 36

5. Radical trapping experiment **40**

6. UV-Vis absorption spectrum **41**

7. References **42**

8. NMR Spectra **44**

1. General considerations

Unless otherwise stated, all reactions were conducted under an atmosphere of nitrogen with anhydrous solvents using standard Schlenk techniques. Glassware was dried in an oven (> 100 °C) and allowed to cool to room temperature under vacuum before use. Cooling of reaction mixtures to 0 °C was achieved using an ice-water bath. Cooling of reaction mixtures to -41 °C was achieved using a dry ice/acetonitrile bath. Cooling of reaction mixtures to -78 °C was achieved using a dry ice/acetone bath. Heating of reaction mixtures to the stated temperature was achieved using an oil bath. Unless otherwise stated, all chemicals were purchased from commercial sources (Sigma Aldrich, Fluorochem, Fisher Scientific, Alfa Aesar or Apollo Scientific) and were used without further purification. Anhydrous solvents and NEt_3 were purified by filtration through dried alumina columns using the University of Oxford internal solvent drying system (Innovative Technology Inc. PS-400- 7) and sparged with nitrogen before use. Solvents used were kept under an atmosphere of N_2 and stored over 3Å molecular sieves. All inert gases were sourced from the University of Oxford internal supplies and dried through $CaCl_2$ drying columns. Thin-layer chromatography (TLC) was performed on Merck silica gel 60 F254 pre-coated aluminium backed TLC sheets with visualisation under a UV lamp (λ_{max} = 254 nm) and/or by staining with $KMnO_4$ solution. Flash column chromatography was performed by loading the compound as an oil or concentrated solution onto a column, using Merck silica gel 60 (230-400 mesh) with the appropriate solvent. ^{1}H NMR, ^{13}C NMR and ^{19}F NMR spectra were recorded on a Brüker AVIII spectrometers (400-600 MHz). Acquisitions were carried out at room temperature unless otherwise stated. All reported ^{1}H and ^{13}C chemical shifts (δH, δC) are referenced to the residual signal of deuterated solvents ($CDCl_3$: δH = 7.26 ppm, δC = 77.2 ppm; $(CD_3)_2SO$: δH = 2.50 ppm, δC = 39.5 ppm). Chemical shifts (δ) are reported in parts per million (ppm) to the nearest 0.01 ppm for ^{1}H NMR, and 0.1 ppm for ^{13}C and ^{19}F NMR. Coupling constants (*J*) are reported in Hertz (Hz) and rounded to the nearest 0.5 Hz. Multiplicities are reported as followings: s (singlet), d (doublet), t (triplet), q (quartet), m (multiplet), hep (heptet), br. (broad signal), app. (apparent). Infrared spectra were recorded on a Bruker Tensor 27 Fourier Transform spectrometer with an internal range 600-4000 cm^{-1} and all absorption maximum (ν_{max}) are given in wavenumbers (cm^{-1}). Melting points were recorded in degrees Celsius (°C) using a Reichert melting point apparatus and are reported uncorrected. High resolution mass spectra (HRMS) were recorded using a Waters BioAccord TOF spectrometer spectrometer, through electrospray ionisation (ESI). m/z values are reported in Daltons (Da) and high-resolution values are calculated to four decimal places from the molecular formula, with all found values within a tolerance of 5 ppm. Samples for mass spectra were prepared in 1 mg/mL solution in MeCN or MeOH.

1.1 Photochemical reaction set-up

Photochemical reactions were carried out in an EvoluChem^{TM} PhotoRedOx Box at room temperature with fan cooling, under visible light irradiation by a PR160L-427 nm Kessil blue LED. A single 427 nm 19V Kessil lamp was used in this system with intensity set at 100. Reactions were performed in microwave vials fitted with an aluminium seal with a blue PTFE/white silicone septum. Fan cooling was used during the reactions to give an internal temperature of ~31 °C.

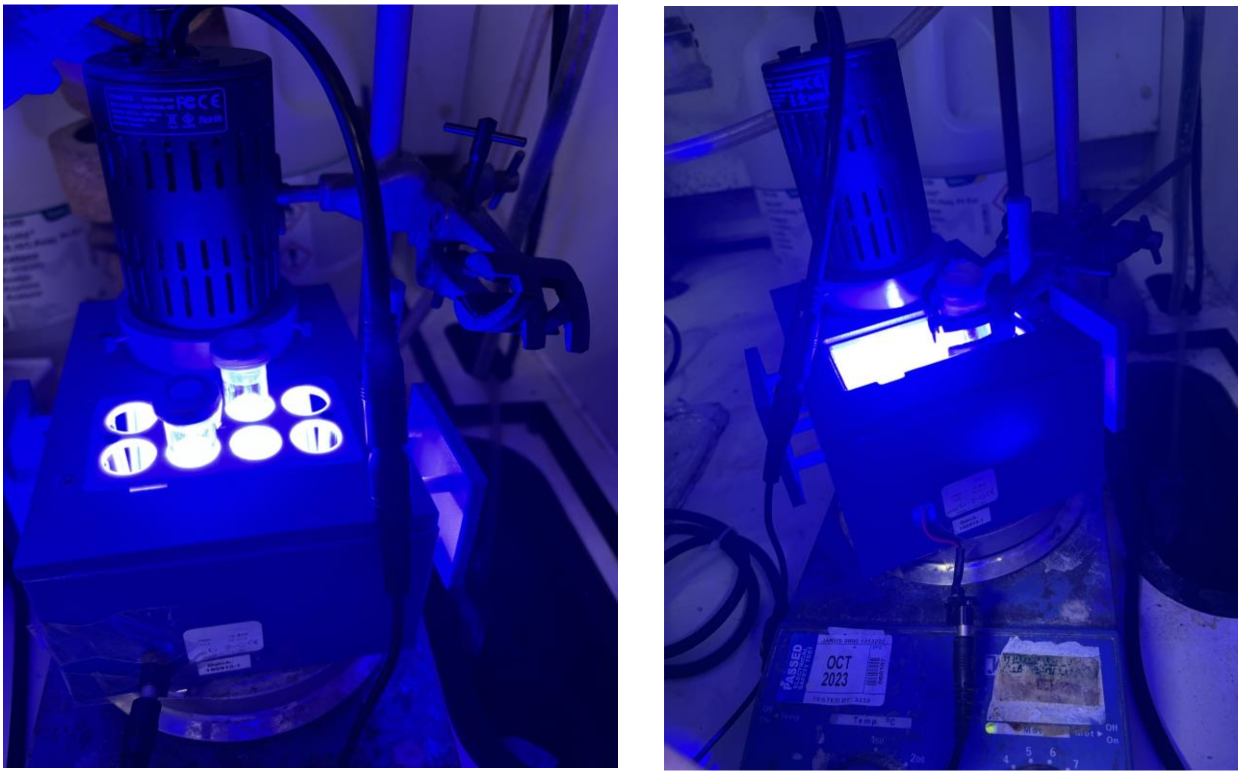

Figure S1 – General photochemical set-up for 0.2 mmol reactions (left) and 2 mmol scale reactions (right)

2. Preparation of starting materials

2.1 Preparation of thianthrenium salts

Thianthrene-5-oxide (**S1**) and thianthrenium tetrafluoroborate salts **1b-d, g-h, k-o, q-s** were prepared according to previously described methods in the literature.[1,2,3,4,5, 6]

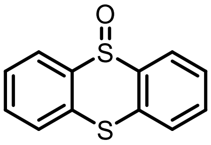

S1, Ref. 5

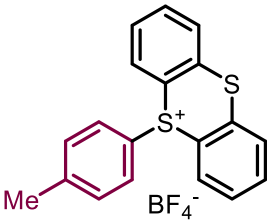

1b, Ref. 6

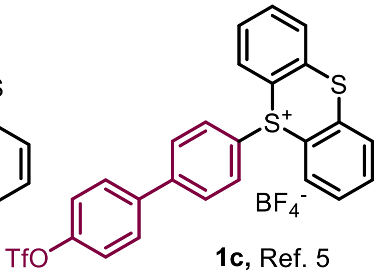

1c, Ref. 5

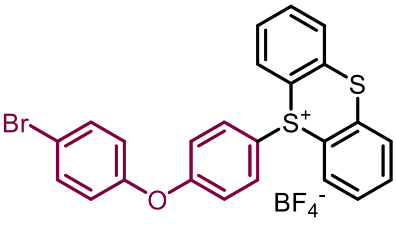

1d, Ref. 5

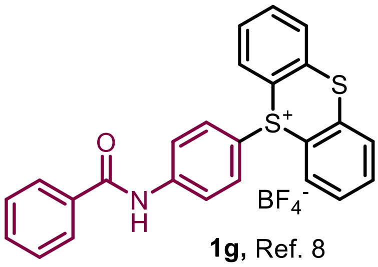

1g, Ref. 8

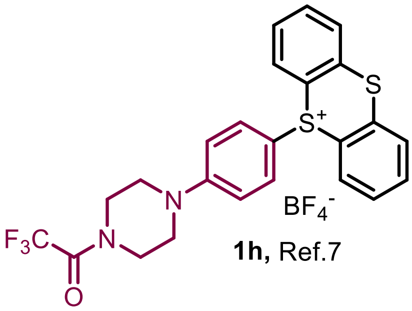

1h, Ref.7

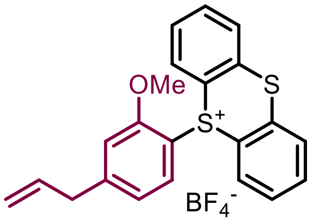

1k, Ref. 8

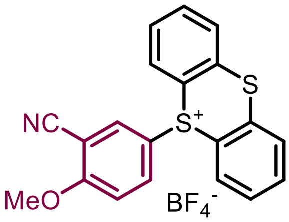

1l, Ref. 8

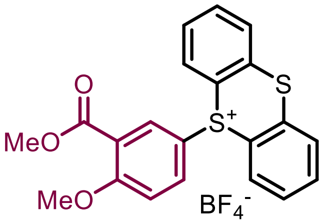

1m, Ref. 8

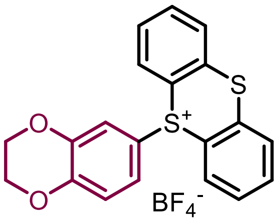

1n, Ref. 9

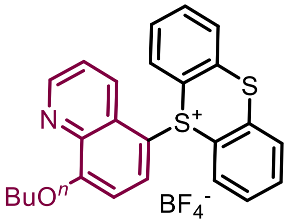

1o, Ref. 7

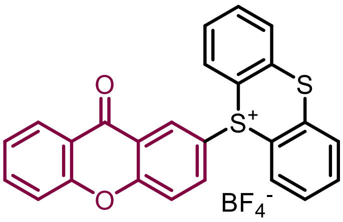

1q, Ref. 10

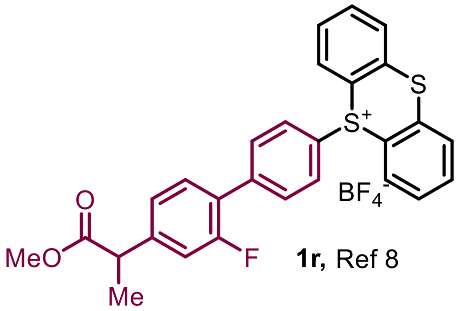

1r, Ref 8

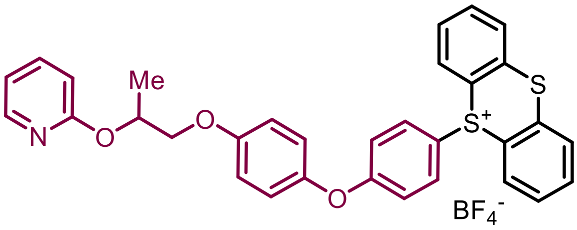

1s, Ref. 5

Thianthrenium tetrafluoroborate salts **1a, e-f, i-j, p** were either novel or prepared according to adapted literature procedures.

4-Fluorophenylthianthrenium tetrafluoroborate 1a

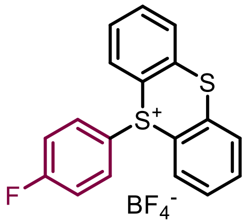

Under ambient conditions, a 50 mL round-bottomed flask was equipped with a magnetic stirrer bar and charged with thianthrene-5-oxide **S1** (928 mg, 4.00 mmol, 1.0 equiv.), fluorobenzene (8.00 mL, 85.0 mmol, 21 equiv.) and MeCN (0.5 M, 8 mL). The flask was cooled to 0 °C and $HBF_4 \cdot OEt_2$ (0.82 mL, 6.00 mmol, 1.5 equiv.) was added dropwise. Subsequently TFAA (1.69 mL, 12.0 mmol, 3.0 equiv.) was added dropwise at 0 °C, resulting in a colour change to deep purple. The mixture was stirred at 0 °C for 1 h, then warmed to rt and stirred for 20 h. The mixture was then concentrated *in vacuo*. The residue was dissolved in CH_2Cl_2 (40 mL) and poured into sat. aq. $NaHCO_3$ (40 mL). The phases were separated and the organic layer was washed with 10% w/w $NaBF_4$ solution (3 × 40 mL). The organic layer was dried over anhydrous $MgSO_4$, filtered and concentrated *in vacuo*. The solid residue was dissolved in CH_2Cl_2 (~ 5 mL) and precipitated with Et_2O. The solid was collected and dried *in vacuo* to afford **1a** as a light orange solid (1.08 g, 68%) without further purification.

R_f = 0.21 (40% acetone in CH_2Cl_2); **^{1}H NMR** (400 MHz, DMSO-d_6) δ 8.58 (dd, *J* = 7.9, 1.4 Hz, 2H), 8.08 (dd, *J* = 7.9, 1.4 Hz, 2H), 7.93 (td, *J* = 7.7, 1.5 Hz, 2H), 7.86 (td, *J* = 7.7, 1.4 Hz, 2H), 7.46 – 7.40 (m, 2H), 7.33 – 7.27 (m, 2H); **^{13}C NMR** (101 MHz, DMSO-d_6) δ 164.2 (d, *J* = 252.3 Hz), 135.6, 135.3, 134.8, 131.1 (d, *J* = 9.5 Hz), 130.3, 129.6, 120.5 (d, *J* = 3.2 Hz), 119.5, 117.8 (d, *J* = 23.8 Hz); **^{19}F NMR** (377 MHz, DMSO-d_6) δ -106.3 (tt, *J* = 8.7, 4.7 Hz), -148.2 (br. s), -148.3 (br. s); **HRMS** (ESI$^+$, *m/z*) calcd. for $[C_{18}H_{12}FS_2]^+$ $[M-BF_4]^+$: 311.0359; found: 311.0355.

Data for this compound is in accordance with the previous literature.[7]

5-(4-(4-Nitrophenoxy)phenyl)-5*H*-thianthren-5-ium tetrafluoroborate 1e

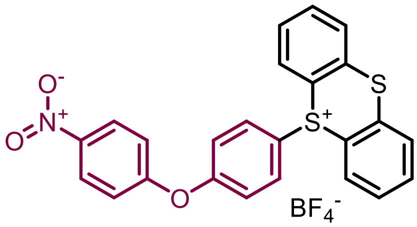

The title compound was prepared according to a modified procedure from literature.[8] An oven-dried, 25 mL round-bottomed flask was equipped with a magnetic stirrer bar and was charged with thianthrene 5-oxide **S1** (232 mg, 1.0 mmol, 1.0 equiv.) and 1-nitro-4-phenoxybenzene (215 mg, 1.0 mmol, 1.0 equiv.). Under an N_2 atmosphere, the flask was charged with anhydrous, degassed MeCN (0.4 M, 2.5 mL) and cooled to -41 °C. Subsequently TFAA (0.42 mL, 3.0 mmol, 3.0 equiv.) was added slowly and TfOH (0.13 mL, 1.5 mmol, 1.5 equiv.) dropwise. The mixture was stirred at -41 °C for 1 h, then warmed to rt and stirred for 18 h. The solution was concentrated, diluted with CH_2Cl_2 (10 mL) and quenched with sat. aq. $NaHCO_3$ (10 mL). The layers were separated and the organic layer further washed with 10% w/w $NaBF_4$ solution (3 × 10 mL). The organic layer was dried over anhydrous $MgSO_4$, filtered and concentrated *in vacuo*. The solid residue was dissolved in CH_2Cl_2 (≈ 5 mL) and precipitated with Et_2O. The solid was collected and dried *in vacuo* to afford **1e** as a light-grey solid (353 mg, 68%) without further purification.

R_f = 0.37 (40% acetone in CH_2Cl_2); **^{1}H NMR** (400 MHz, $CDCl_3$) δ 8.59 (t, *J* = 6.6 Hz, 2H), 8.25 – 8.18 (m, 2H), 7.89 – 7.74 (m, 6H), 7.29 (d, *J* = 8.7 Hz, 2H), 7.12 – 7.01 (m, 4H); **^{13}C NMR** (101 MHz, $CDCl_3$) δ 160.5, 159.7, 144.3, 136.5, 135.5, 135.2, 130.9, 130.6, 130.5, 126.3, 121.2, 119.4, 119.0, 118.7; **^{19}F NMR** (377 MHz, $CDCl_3$) -150.7 (br. s), -150.8 (br. s); **HRMS** (ESI^+, *m/z*) calcd. for $[C_{24}H_{16}NO_3S_2]^+$ $[M-BF_4]^+$: 430.0566; found: 430.0572; **IR** (ν_{max}, cm^{-1}) 3090, 2361, 2341, 1727, 1578, 1486, 1345, 1250, 1062, 878, 768.

5-(4-(Prop-2-yn-1-yloxy)phenyl)-*5H*-thianthren-5-ium tetrafluoroborate 1f

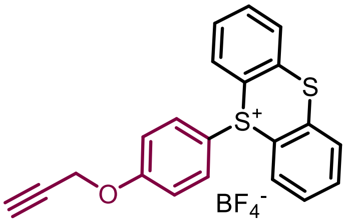

An oven-dried, 50 mL round bottomed flask was equipped with a magnetic stirrer bar and was charged with thianthrene 5-oxide **S1** (464 mg, 2.0 mmol, 1.0 equiv.) and (prop-2-yn-1-yloxy)benzene (264 µL, 2.0 mmol, 1.0 equiv.). The flask was sealed and subjected to three evacuation/N_2 refill cycles before anhydrous, degassed MeCN (0.25 M, 8.0 mL) was added under N_2. The flask was cooled to 0 °C and subsequently TfOH (0.27 mL, 3.0 mmol, 1.5 equiv.) and TFAA (0.83 mL, 6.0 mmol, 3.0 equiv.) were added dropwise at 0 °C. The mixture was stirred at 0 °C for 1 h, then at rt for 2 h. The solution was quenched with MeOH (30 mL) and concentrated *in vacuo*. The residue was dissolved in

CH_2Cl_2 (20 mL) and washed with 10% w/w $NaBF_4$ solution (3 × 20 mL). The organic layer was dried over anhydrous $MgSO_4$, filtered and concentrated *in vacuo*. The residue was dissolved in CH_2Cl_2 (≈ 5 mL) and precipitation with Et_2O and drying *in vacuo* afforded **1f** as a light-yellow solid (706 mg, 81%).

R_f = 0.39 (40% acetone in CH_2Cl_2); **^{1}H NMR** (400 MHz, DMSO-d_6) δ 8.52 (dt, J = 7.9, 1.3 Hz, 2H), 8.06 (d, J = 7.8 Hz, 2H), 7.90 (tt, J = 7.8, 1.3 Hz, 2H), 7.83 (tt, J = 7.5, 1.3 Hz, 2H), 7.27 (dd, J = 9.2, 1.2 Hz, 2H), 7.20 – 7.11 (m, 2H), 4.86 (dd, J = 2.5, 1.2 Hz, 2H), 3.62 (td, J = 2.5, 1.2 Hz, 1H); **^{13}C NMR** (101 MHz, DMSO-d_6) δ 160.5, 135.0, 135.0, 134.6, 130.3, 130.2, 129.6, 119.7, 117.0, 115.4, 79.1, 78.2, 56.0; **^{19}F NMR** (377 MHz, DMSO-d_6) δ -148.2 (br. s), -148.2 (br. s); **HRMS** (ESI$^+$, *m/z*) calcd. for $[C_{21}H_{15}OS_2]^+$ $[M\text{-}BF_4]^+$: 347.0559; found: 347.0562; **IR** (v_{max}, cm^{-1}) 3269, 2361, 2341, 1726, 1587, 1493, 1451, 1266, 1184, 1059, 831, 763.

5-(4-Cyanophenyl)-5*H*-thianthren-5-ium tetrafluoroborate 1i

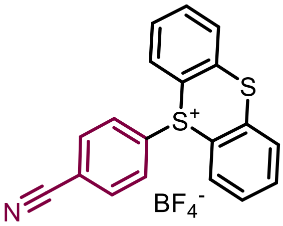

The title compound was prepared according to a modified procedure from literature.[9] An oven-dried, 10 mL microwave vial was equipped with a magnetic stirrer bar and was charged with 4-cyanophenylboronic acid (147 mg, 1.0 mmol, 1.0 equiv.), thianthrene (162 mg, 1.5 mmol, 1.5 equiv.) and $Cu(OTf)_2$ (724 mg, 2.0 mmol, 2.0 equiv.) The vial was sealed and subjected to three evacuation/N_2 refill cycles before degassed MeCN (1.0 M, 1.0 mL) and H_2O (36 µL, 2.0 mmol, 2.0 equiv.) was added under N_2. The reaction mixture was stirred at 100 °C for 24 h. The reaction mixture was cooled to rt and added into ammonia solution (100 mL, 25-28% solution in water). The aqueous phase was extracted with CH_2Cl_2 (2 x 30 mL). The combined organic layers were washed with 10% w/w $NaBF_4$ solution (3 × 30 mL). The organic layer was dried over anhydrous $MgSO_4$, filtered and concentrated *in vacuo*. The crude precipitate was purified by flash column chromatography (CH_2Cl_2/acetone 1:0 to 8.5:1.5) affording **1i** as an off-white solid (129 mg, 32%).

R_f = 0.29 (40% MeOH in CH_2Cl_2); **^{1}H NMR** (400 MHz, DMSO-d_6) δ 8.62 (dd, J = 7.8, 1.6 Hz, 2H), 8.08 (dd, J = 7.8, 1.5 Hz, 2H), 8.03 – 7.84 (m, 6H), 7.35 (d, J = 8.3 Hz, 2H); **^{13}C NMR** (101 MHz, DMSO-d_6) δ 136.3, 135.6, 135.1, 133.8, 130.8, 130.4, 129.6, 129.1, 119.2, 117.2, 115.0; **^{19}F NMR** (377 MHz, DMSO-d_6) -148.2 (br. s), -148.3 (br. s); **HRMS** (ESI$^+$, *m/z*) calcd. for $[C_{19}H_{12}NS_2]^+$ [M-

$BF_4]^+$: 318.0406; found: 318.0394; **IR** (ν_{max}, cm^{-1}) 3093, 2398, 1724, 1568, 1450, 1353, 1281, 1145, 1058, 770.

5-(3,5-bis(Trifluoromethyl)phenyl)-5*H*-thianthren-5-ium tetrafluoroborate 1j

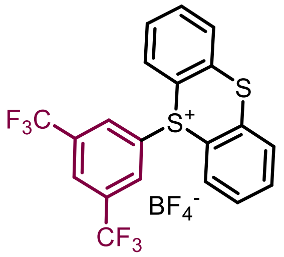

The title compound was prepared according to a modified procedure from literature.[9] An oven-dried, 10 mL microwave vial was equipped with a magnetic stirrer bar and was charged with [3,5-bis(trifluoromethyl)phenyl]boronic acid (258 mg, 1.0 mmol, 1.0 equiv.), thianthrene (162 mg, 1.5 mmol, 1.5 equiv.) and $Cu(OTf)_2$ (724 mg, 2.0 mmol, 2.0 equiv.) The vial was sealed and subjected to three evacuation/N_2 refill cycles before degassed MeCN (1.0 M, 1.0 mL) and H_2O (36 µL, 2.0 mmol, 2.0 equiv.) was added under N_2. The reaction mixture was stirred at 100 °C for 24 h. The reaction mixture was cooled to rt and added into ammonia solution (100 mL, 25-28% solution in water). The aqueous phase was extracted with CH_2Cl_2 (2 x 30 mL). The combined organic layers were washed with 10% w/w $NaBF_4$ solution (3 × 30 mL). The organic layer was dried over anhydrous $MgSO_4$, filtered and concentrated *in vacuo*. The crude precipitate was purified by flash column chromatography (CH_2Cl_2/acetone 3:1) affording **1j** as an off-white solid (224 mg, 43%).

R_f = 0.41 (40% acetone in CH_2Cl_2); **^{1}H NMR** (400 MHz, DMSO-*d*$_6$) δ 8.65 (dd, *J* = 7.8, 1.6 Hz, 2H), 8.52 (s, 1H), 8.10 (dd, *J* = 7.8, 1.5 Hz, 2H), 7.94 (dtd, *J* = 19.9, 7.5, 1.5 Hz, 4H), 7.76 (s, 2H); **^{13}C NMR** (101 MHz, DMSO-*d*$_6$) δ 136.7, 135.6, 135.2, 131.4 (q, *J* = 34.1 Hz), 130.4, 129.7, 129.4, 129.2 (q, *J* = 3.7 Hz), 126.9 (q, *J* = 2.6 Hz), 122.1 (q, *J* = 273.4 Hz), 119.2; **^{19}F NMR** (377 MHz, DMSO-*d*$_6$) δ -61.6 (s), -148.2 (br. s), -148.3 (br. s); **HRMS** (ESI^+, *m/z*) calcd. for $[C_{20}H_{11}F_6S_2]^+$ $[M-BF_4]^+$: 429.0201; found: 429.0203; **IR** (ν_{max}, cm^{-1}) 1724, 1568, 1450, 1352, 1280, 1244, 1057, 892, 758.

5-(6M-3-yl)-5*H*-thianthren-5-ium tetrafluoroborate 1p

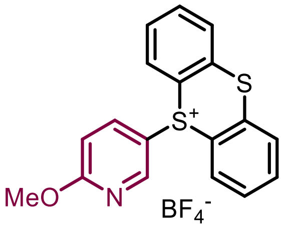

The title compound was prepared according to a modified procedure from literature.[10] An oven-dried, 100 mL round-bottomed flask was equipped with a magnetic stirrer bar and was charged with thianthrene 5-oxide **S1** (929 mg, 4.0 mmol, 1.0 equiv.) and anhydrous K_3PO_4 (1.83 g, 8.6 mmol, 2.15 equiv.). The flask was sealed and subjected to three evacuation/N_2 refill cycles before anhydrous, degassed MeCN (0.2 M, 20 mL) and 2-methoxypyridine (1.03 mL, 9.76 mmol, 2.44 equiv.) was added. The flask was cooled to -41 °C and subsequently Tf_2O (1.02 mL, 6.08 mmol, 1.52 equiv.) was added dropwise. The mixture was stirred at -41 °C for 1 h, then warmed to rt and stirred for 22 h. The solution was quenched with sat. aq. $NaHCO_3$ (100 mL) and extracted with CH_2Cl_2 (3 × 75 mL). After separating the phases, the organic layer was washed with 10% w/w $NaBF_4$ solution (3 × 75 mL). The organic layer was dried over anhydrous $MgSO_4$, filtered and concentrated *in vacuo*. The crude precipitate was purified by flash column chromatography (CH_2Cl_2/*i*-PrOH 1:0 to 8.5:1.5). The residue was dissolved in CH_2Cl_2 (≈ 5 mL) and precipitation with Et_2O at 0 °C and drying *in vacuo* afforded **1p** as a light grey solid (210 mg, 13%).

R_f = 0.26 (40% acetone in CH_2Cl_2); **^{1}H NMR** (400 MHz, $CDCl_3$) δ 8.64 – 8.53 (m, 2H), 7.93 (d, *J* = 2.8 Hz, 1H), 7.87 – 7.72 (m, 6H), 7.63 (dd, *J* = 9.1, 2.8 Hz, 1H), 6.80 (d, *J* = 9.1 Hz, 1H), 3.89 (s, 3H); **^{13}C NMR** (101 MHz, $CDCl_3$) δ 166.9, 147.6, 138.5, 136.4, 135.3, 135.1, 130.6, 130.3, 118.6, 113.9, 112.8, 54.7; **^{19}F NMR** (377 MHz, $CDCl_3$) δ -150.6 (br. s), -150.7 (br. s); **HRMS** (ESI$^+$, *m/z*) calcd. for $[C_{18}H_{14}NOS_2]^+$ $[M-BF_4]^+$: 324.0511; found: 324.0500;

Data for this compound is in accordance with the previous literature.[5]

2.2 Preparation of sulfinylamines

Sulfinylamines were prepared according to literature procedures.[11,12,13,14,15]

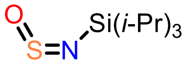

TIPS-NSO **2a**, Ref. 11

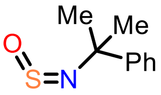

Cumyl-NSO **2b**, Ref. 12

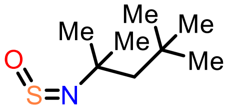

t-Oct-NSO **2c**, Ref. 13

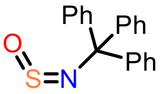

Tr-NSO **2d**, Ref. 14

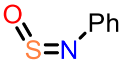

Ph-NSO **2e**, Ref. 15

Note:

1) The density of TIPS-NSO **2a** was measured as 0.97 g ml^{-1} and used in subsequent experiments.

3. Optimization of the reaction conditions

3.1 Optimization of dual amine EDA system

Table 1. Initial HAT source and photocatalyst screen

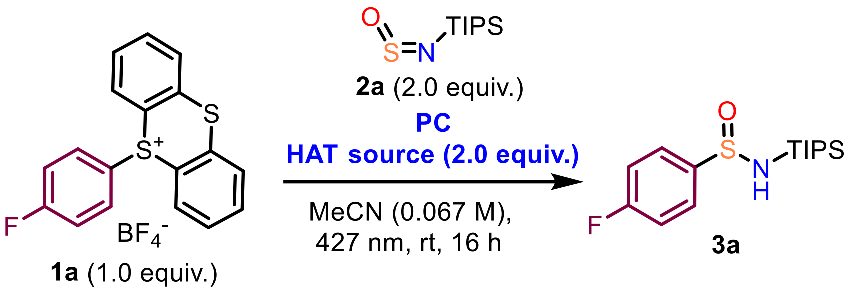

Entry	PC	HAT source	Yield of **3a***
**1**	**DABCO (3.0 equiv.)**	**NEt_3**	**52**
2	DABCO (3.0 equiv.)	DIPEA	45
3	*fac*-Ir(ppy)$_3$ (2 mol%)	DIPEA	35
4	DABCO (3.0 equiv.)	$(TMS)_3Si$-H	17
5	DABCO (3.0 equiv.)	Ph_3CH	11
6	DABCO (3.0 equiv.)	1,4-cyclohexadiene	29

Reactions performed on 0.1 mmol scale. *Yields determined by quantitative ^{19}F NMR spectroscopy of the crude reaction mixture using *α,α,α*-trifluorotoleuene as the internal standard

Table 2. Solvent screen

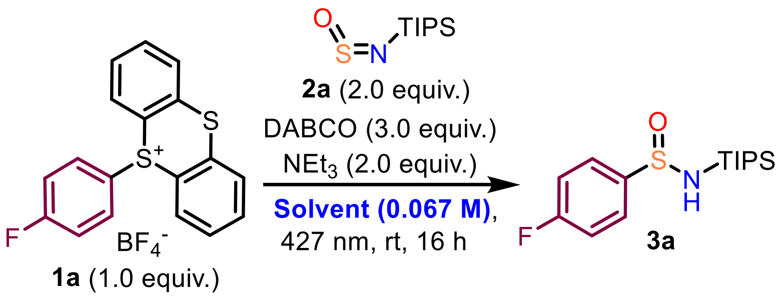

Entry	Solvent	Yield of **3a***
**1**	**MeCN**	**52**
2	DMF	29[a]
3	DCM	33
4	DMSO	30
5	1,4-dioxane	8
6	Toluene	2
7	*t*-amyl alcohol	0
8	EtOAc	0
9	Acetone	0
10	DCM/MeCN 1:1	51

Reactions performed on 0.1 mmol scale. *Yields determined by quantitative ^{19}F NMR spectroscopy of the crude reaction mixture using *α,α,α*-trifluorotoleuene as the internal standard.[a] DIPEA instead of NEt_3

Table 3. EDA donor screen I

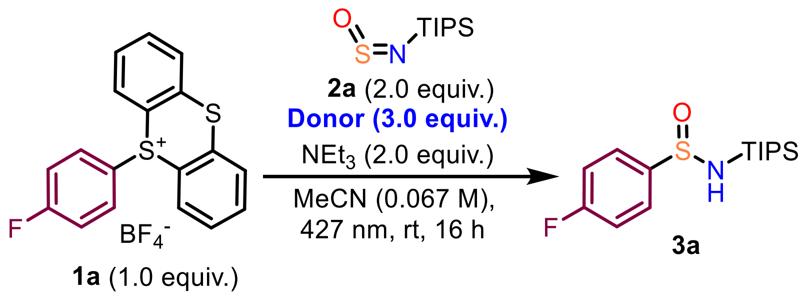

Entry	EDA donor	Yield of **3a***
1	DABCO	52
2	Ph_3N	46
3	quinuclidine	30
4	DBU	33
5	pyrrolopyridine	42
6	K_2CO_3	42[a]
7	DMAP (4.0 equiv.)	57[b]
**8**	**2,6-lutidine**	**58[b]**

Reactions performed on 0.1 mmol scale.*Yields determined by quantitative ^{19}F NMR spectroscopy of the crude reaction mixture using *α,α,α*-trifluorotoleuene as the internal standard. [a] 390 nm. [b] 24h

Table 4. Further screening and additive addition

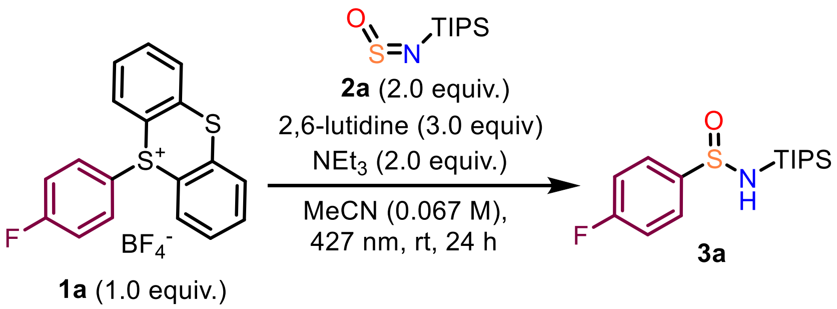

Entry	Variations to conditions above	Yield of **3a***
1	None	58
**2**	**0.1 M MeCN**	**65 (58)**
3	0.2 M MeCN	63
4	$Cu(MeCN)_4BF_4$ (10 mol%) additive	50
5	CuI (10 mol%) additive	44
**6**	CuBr (10 mol%) additive	45
7	CuTC (10 mol%) additive	44
8	$Cu(OAc)_2$ (10 mol%) additive	44
9	$Cu(OTf)_2$ (10 mol%) additive	57
10	$Ca(NTf)_2$ (10 mol%) additive	13
11	1,3-bis-(4-methoxyphenyl)urea (2.0 equiv.) additive	48
12	NBu_3 instead of NEt_3	59
13	$MeN(Cy)_2$ instead of NEt_3	52
14	TMEDA instead of NEt_3	40

Reactions performed on 0.1 mmol scale. *Yields determined by quantitative ^{19}F NMR spectroscopy of the crude reaction mixture using *α,α,α*-trifluorotoleuene as the internal standard. Isolated yields in parenthesis.

Table 5. Amine loading control reactions

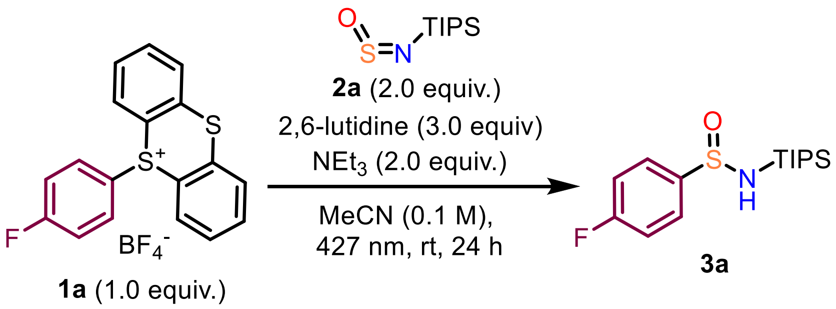

Entry	Variations to conditions above	Yield of **3a***
1	None	65 **(58)**
2	0 equiv. NEt_3 insead of 2.0 equiv.	0
**3**	0 equiv. 2,6-lutidine instead of 3.0 equiv.	57 **(56)**
**4**	**0 equiv. 2,6-lutidine instead of 3.0 equiv., 5.0 equiv. NEt_3 instead of 2.0 equiv.**	59 **(57)**

Reactions performed on 0.1 mmol scale. *Yield determined by quantitative ^{19}F NMR spectroscopy of the crude reaction using *α,α,α*-trifluorotoluene as internal standard. Isolated yields in parenthesis.

Due to the simplicity of the single amine system (**entry 4**) and comparable yields to the dual-amine system (**entry 1**), **entry 4** conditions were selected for further investigation, whereby NEt_3 performs the role of the electron donor partner and HAT reagent.

3.2 Optimization of single amine EDA system

Table 6. Further EDA donor screen

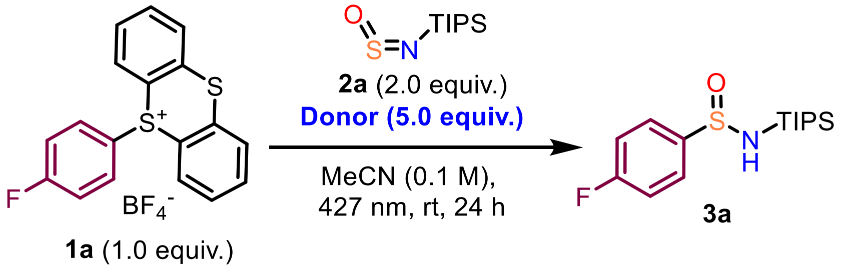

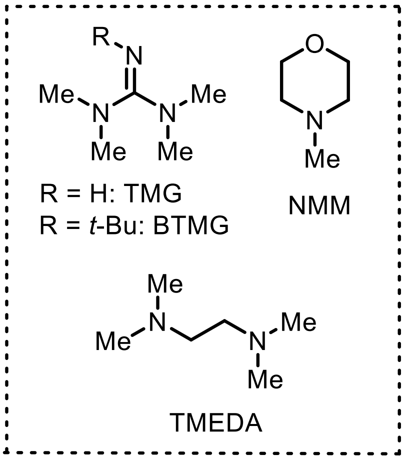

Entry	EDA Donor	Yield of **3a***
**1**	**None**	59 **(57)**
2	$MeNCy_2$	55
**3**	DIPEA	46
4	NBu_3	58
5	DBU	42
6	Et_2NPh	15
7	TMG	33
8	BTMG	26
9	morpholine	27
10	NMM	48
11	TMEDA	47

Reactions performed on 0.1 mmol scale. *Yield determined by quantitative ^{19}F NMR spectroscopy of the crude reaction using α,α,α-trifluorotoluene as internal standard. Isolated yields in parenthesis.

Table 7. Wavelength, time and temperature studies

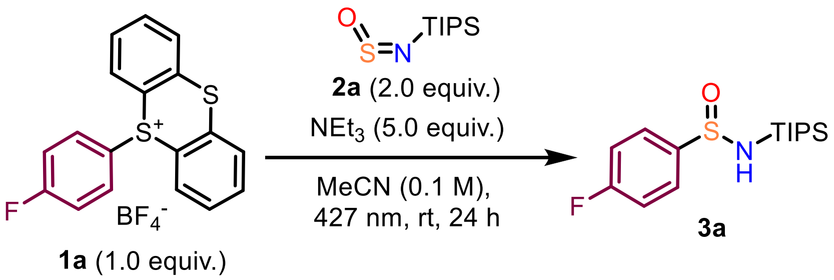

Entry	Variations to conditions above	Yield of **3a***
**1**	**None**	59 **(57)**
2	390 nm instead of 427 nm	56
3	405 nm instead of 427 nm	58
4	450 nm instead of 427 nm	56
5	17 h instead of 24 h	52
6	-10 °C instead of rt	43
7	40 °C instead of rt	42
8	dark	0
9	50% lamp intensity	50

Reactions performed on 0.1 mmol scale. *Yield determined by quantitative ^{19}F NMR spectroscopy of the crude reaction using *α,α,α*-trifluorotoluene as internal standard. Isolated yields in parenthesis.

Table 8. Solvent, concentration, and sulfinylamine loading studies

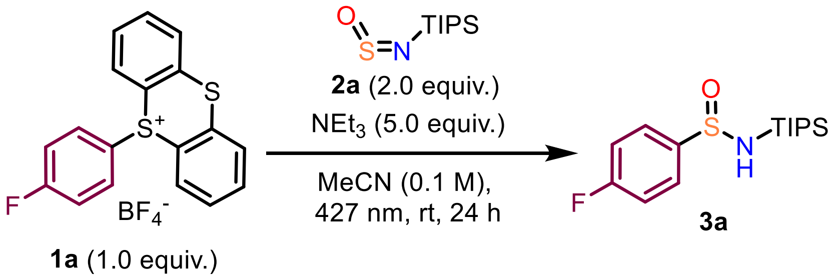

Entry	Variations to conditions above	Yield of **3a***
**1**	**None**	59 **(57)**
2	DCM instead of MeCN	46
3	DMSO instead of MeCN	8
4	DMF instead of MeCN	33
5	0.05 M instead of 0.1 M	42
6	0.2 M instead of 0.1 M	52
7	0.5 M instead of 0.1 M	49
8	1.1 equiv. TIPSNSO	40
9	3.0 equiv. TIPSNSO	55

Reactions performed on 0.1 mmol scale. *Yield determined by quantitative ^{19}F NMR spectroscopy of the crude reaction using *α,α,α*-trifluorotoluene as internal standard. Isolated yields in parenthesis.

Table 9. Salt form and counterion study

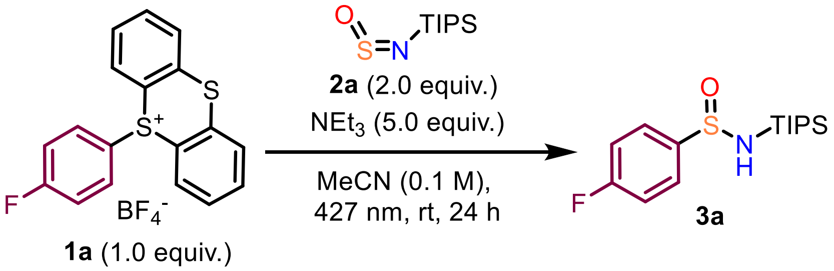

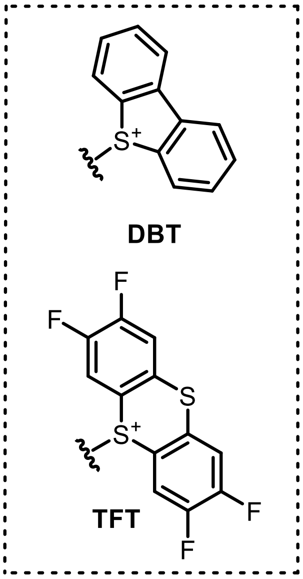

Entry	Variations to conditions above	Yield of **3a***
**1**	**None**	59 **(57)**
2	OTf salt instead of BF_4	58
3	PF_6 salt instead of BF_4	47
4	DBT BF_4^- salt	58
5	TFT BF_4^- salt	50

Reactions performed on 0.1 mmol scale. *Yield determined by quantitative ^{19}F NMR spectroscopy of the crude reaction using *α,α,α*-trifluorotoluene as internal standard. Isolated yields in parenthesis.

Conveniently the reaction performs well for other salt forms and counter-ions. The thianthrenium salt, tetrafluoroborate counter-ion pairing was selected going forward on account of its ease of synthesis under standard electrophilic thianthrenation procedures.

3.3 Mass balance investigation

Table 10. Mass balance NMR experiments

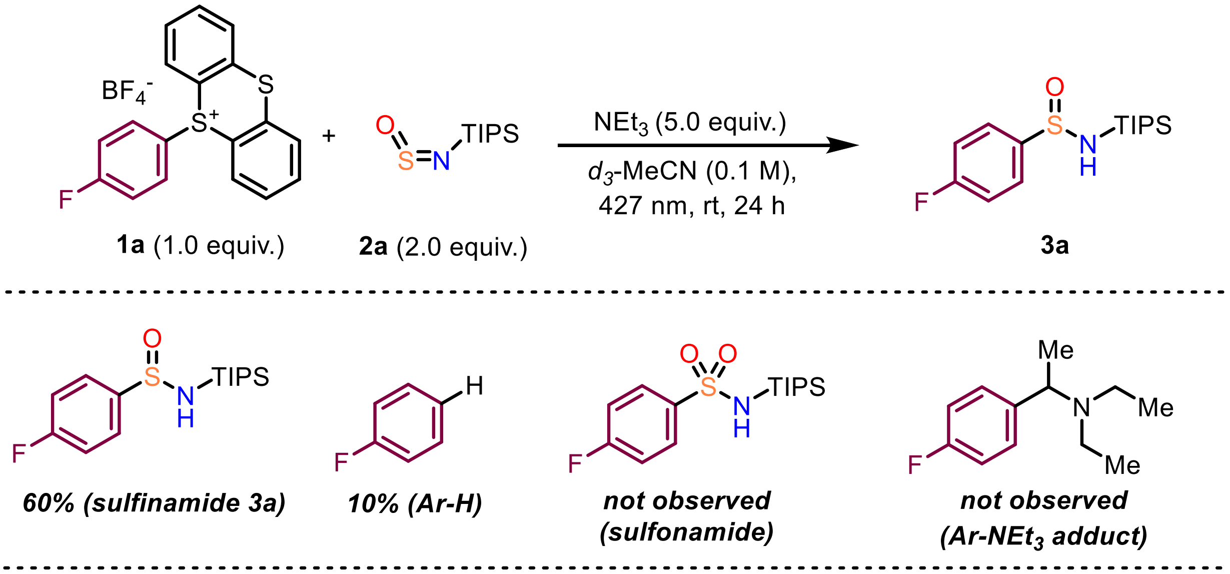

Reaction performed on 0.1 mmol scale. Yields determined by quantitative ^{19}F NMR spectroscopy using α,α,α-trifluorotoleuene as the internal standard.

The results displayed in **Table 10** display typical products for the EDA-sulfinamidation reaction of thianthrenium salts with sulfinylamine reagent **2a**. HAT to the intermediate aryl radical species accounts for notable formation of the protodethianthrenation product (***Ar-H***). Formation of the sulfonamide or aryl-triethylamine radical adduct was not observed under these conditions. The remainder of the mass balance can be attributed to uncharacterised decomposition products from the intermediate aryl- or aryl sulfonimidoyl radical.

4. Experimental procedures:

4.1 General procedures:

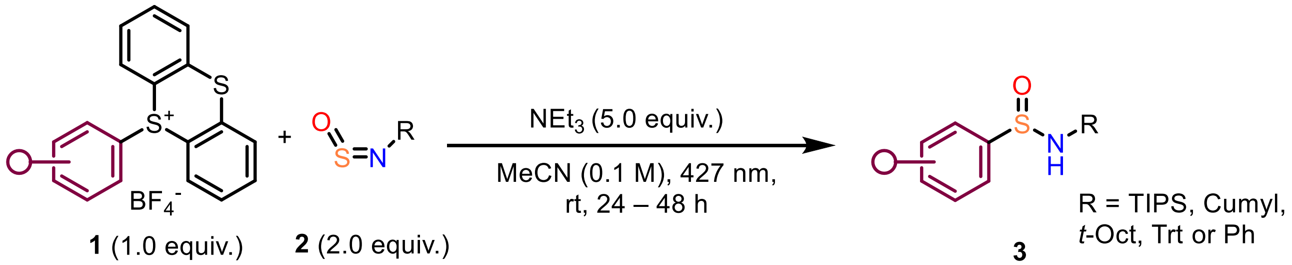

1 (1.0 equiv.) **2** (2.0 equiv.) **3**

General procedure: An oven-dried microwave vial containing the respective tetrafluoroborate thianthrenium salt **1** (1.0 equiv.) and sulfinylamine reagent **2** (if solid), were sealed and subjected to three evacuation/N_2 refill cycles before anhydrous, degassed MeCN (0.1 M) was added. After stirring for 1 minute, ensuring all solids had dissolved, NEt_3 (5.0 equiv.) and sulfinylamine reagent **2** (if liquid) (2.0 equiv.) were then added to the reaction mixture under N_2. The reaction was then stirred for 24 to 48 h under 427 nm irradiation at ambient temperature (31°C, photoreactor fans on). The solution was concentrated *in vacuo* and purified by silica gel flash chromatography to afford the sulfinamide product.

4.2 Sulfinamide substrate scope

4-Fluoro-*N*-(triisopropylsilyl)benzenesulfinamide 3a

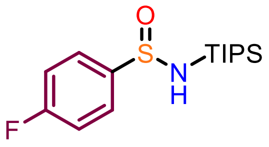

Prepared according to the **General Procedure**, with thianthrenium salt **1a** (79.6 mg, 0.20 mmol, 1.0 equiv.), NEt_3 (0.14 mL, 1.0 mmol, 5.0 equiv.), TIPS-NSO **2a** (90 µL, 0.40 mmol, 2.0 equiv.) and MeCN (2.0 mL). After stirring for 24 h, purification by silica gel flash chromatography (pentane: EtOAc 9:1 to 4:1) afforded **3a** as an off-white solid (35.9 mg, 57%).

$\mathbf{R_f}$ = 0.25 (10% EtOAc in pentane); **^{1}H NMR** (400 MHz, $CDCl_3$) δ 7.74 – 7.69 (m, 2H), 7.19 – 7.14 (m, 2H), 3.68 (s, 1H), 1.31 – 1.22 (m, 3H), 1.14 (dd, *J* = 7.3, 5.8 Hz, 18H); **^{13}C NMR** (151 MHz, $CDCl_3$) δ 164.3 (d, *J* = 250.7 Hz), 146.0 (d, *J* = 3.0 Hz), 127.4 (d, *J* = 9.1 Hz), 116.2 (d, *J* = 22.3 Hz), 18.1, 18.0, 12.0; [note: for methyl groups in TIPS group, 2 peaks were found instead of 1 due to loss

of symmetry caused by chiral sulfur atom]; **^{19}F NMR** (377 MHz, $CDCl_3$) δ -109.8 (tt, J = 8.5, 5.1 Hz); **HRMS** (ESI$^+$, *m/z*) calcd. for $[C_{15}H_{27}FNOSSi]^+$ $[M+H]^+$: 316.1561; found: 316.1564.

Data for this compound is in accordance with the previous literature.[16]

4-Methyl-*N*-(triisopropylsilyl)benzenesulfinamide 3b

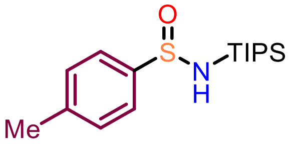

Prepared according to the **General Procedure**, with thianthrenium salt **1b** (78.8 mg, 0.20 mmol, 1.0 equiv.), NEt_3 (0.14 mL, 1.0 mmol, 5.0 equiv.), TIPS-NSO **2a** (90 µL, 0.40 mmol, 2.0 equiv.) and MeCN (2.0 mL). After stirring for 24 h, purification by silica gel flash chromatography (pentane: EtOAc 9:1 to 4:1) afforded **3b** as an off-white solid (28.8 mg, 46%).

R_f = 0.36 (25% EtOAc in pentane); **^{1}H NMR** (400 MHz, $CDCl_3$) δ 7.61 (d, J = 8.2 Hz, 2H), 7.29 (d, J = 8.2 Hz, 2H), 3.66 (s, 1H), 2.40 (s, 3H), 1.31 – 1.21 (m, 3H), 1.14 (t, J = 6.5 Hz, 18H); **^{13}C NMR** (101 MHz, $CDCl_3$) δ 147.3, 141.1, 129.7, 125.1, 21.4, 18.1, 18.0, 11.9; [note: for methyl groups in TIPS group, 2 peaks were found instead of 1 due to loss of symmetry caused by chiral sulfur atom]; **HRMS** (ESI$^+$, *m/z*) calcd. for $[C_{16}H_{30}NOSSi]^+$ $[M+H]^+$: 312.1812; found: 312.1825.

Data for this compound is in accordance with the previous literature.[16]

4'-(((Triisopropylsilyl)amino)sulfinyl)-[1,1'-biphenyl]-4-yl trifluoromethanesulfonate 3c

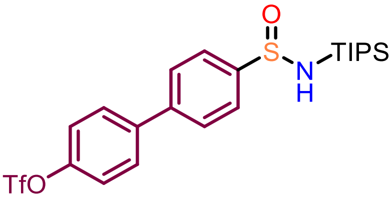

Prepared according to the **General Procedure**, with thianthrenium salt **1c** (120.8 mg, 0.20 mmol, 1.0 equiv.), NEt_3 (0.14 mL, 1.0 mmol, 5.0 equiv.), TIPS-NSO **2a** (90 µL, 0.40 mmol, 2.0 equiv.) and MeCN (2.0 mL). After stirring for 24 h, purification by silica gel flash chromatography (pentane: EtOAc 9:1 to 7:3) afforded **3c** as an off-white solid (57.7 mg, 55%).

Rf = 0.22 (20% EtOAc in pentane); **^{1}H NMR** (400 MHz, $CDCl_3$) δ 7.86 – 7.80 (m, 2H), 7.67 (ddd, J = 8.9, 4.5, 1.6 Hz, 4H), 7.41 – 7.32 (m, 2H), 3.75 (s, 1H), 1.35 – 1.24 (m, 3H), 1.17 (dd, J = 7.2, 5.4 Hz, 18H); **^{13}C NMR** (101 MHz, $CDCl_3$) δ 150.0, 149.5, 141.8, 140.6, 129.2, 127.9, 125.9, 122.0,

118.9 (q, J = 320.7 Hz), 18.1, 18.0, 12.0; [note: for methyl groups in TIPS group, 2 peaks were found instead of 1 due to loss of symmetry caused by chiral sulfur atom]; **^{19}F NMR** (377 MHz, $CDCl_3$) δ -72.8 (s); **IR** (ν_{max}, cm^{-1}) 2954, 2868, 2360, 2341, 1511, 1426, 1213, 1142, 884; **HRMS** (ESI^+, *m/z*) calcd. for $[C_{22}H_{31}F_3NO_4S_2Si]^+$ $[M+H]^+$ 522.1410; found: 522.1422; **MP**: 104 – 105 °C (CH_2Cl_2).

4-(4-Bromophenoxy)-*N*-(triisopropylsilyl)benzenesulfinamide 3d

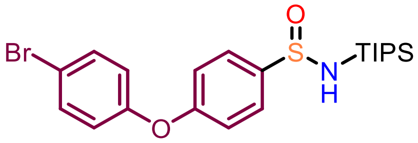

Prepared according to the **General Procedure**, with thianthrenium salt **1d** (110.0 mg, 0.20 mmol, 1.0 equiv.), NEt_3 (0.14 mL, 1.0 mmol, 5.0 equiv.), TIPS-NSO **2a** (90 µL, 0.40 mmol, 2.0 equiv.) and MeCN (2.0 mL). After stirring for 24 h, purification by silica gel flash chromatography (pentane: EtOAc 5:1 to 4:1) afforded **3d** as a light-orange solid (37.0 mg, 40%).

R_f = 0.36 (20% EtOAc in pentane); **^{1}H NMR** (400 MHz, $CDCl_3$) δ 7.69 (d, J = 8.8 Hz, 2H), 7.48 (d, J = 8.9 Hz, 2H), 7.07 (d, J = 8.8 Hz, 2H), 6.93 (d, J = 8.9 Hz, 2H), 3.68 (s, 1H), 1.34 – 1.20 (m, 3H), 1.15 (t, J = 6.7 Hz, 18H); **^{13}C NMR** (101 MHz, $CDCl_3$) δ 159.4, 155.5, 144.7, 133.1, 127.2, 121.5, 118.6, 117.0, 18.1, 18.0, 12.0; [note: for methyl groups in TIPS group, 2 peaks were found instead of 1 due to loss of symmetry caused by chiral sulfur atom]; **IR** (ν_{max}, cm^{-1}) 3173, 2944, 2867, 1579, 1483, 1243, 1087, 1069, 880; **HRMS** (ESI^+, *m/z*) calcd. for $[C_{21}H_{31}{}^{79}BrNO_2SSi]^+$ $[M+H]^+$ 468.1023; found 468.1035; $[C_{21}H_{31}{}^{81}BrNO_2SSi]^+$ $[M+H]^+$ 470.1002; found: 470.1011; **MP**: 105 – 106 °C (CH_2Cl_2).

4-(4-Nitrophenoxy)-*N*-(triisopropylsilyl)benzenesulfinamide 3e

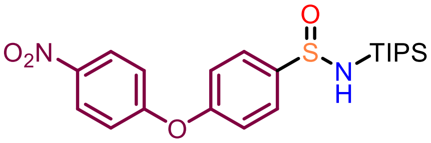

Prepared according to the **General Procedure**, with thianthrenium salt **1e** (103.4 mg, 0.20 mmol, 1.0 equiv.), NEt_3 (0.14 mL, 1.0 mmol, 5.0 equiv.), TIPS-NSO **2a** (90 µL, 0.40 mmol, 2.0 equiv.) and MeCN (2.0 mL). After stirring for 24 h, purification by silica gel flash chromatography (pentane: EtOAc 9:1 to 4:1) afforded **3e** as an off-white solid (29.8 mg, 34%).

R_f = 0.21 (20% EtOAc in pentane); **^{1}H NMR** (400 MHz, $CDCl_3$) δ 8.27 – 8.18 (m, 2H), 7.83 – 7.75 (m, 2H), 7.23 – 7.15 (m, 2H), 7.11 – 7.01 (m, 2H), 3.73 (s, 1H), 1.28 (ddd, *J* = 16.3, 8.1, 1.6 Hz, 3H), 1.15 (t, *J* = 6.7 Hz, 18H); **^{13}C NMR** (101 MHz, $CDCl_3$) δ 162.4, 157.2, 146.8, 143.4, 127.6, 126.2, 120.5, 118.1, 18.1, 18.0, 11.9; [note: for methyl groups in TIPS group, 2 peaks were found instead of 1 due to loss of symmetry caused by chiral sulfur atom]; **IR** (ν_{max}, cm^{-1}) 2944, 2867, 1580, 1519, 1485, 1343, 1246, 1111, 1086, 877; **HRMS** (ESI^+, *m/z*) calcd. for $[C_{21}H_{31}N_2O_4SSi]^+$ $[M+H]^+$ 435.1768; found 435.1778; **MP**: 79 – 80 °C (CH_2Cl_2).

4-(Prop-2-yn-1-yloxy)-*N*-(triisopropylsilyl)benzenesulfinamide 3f

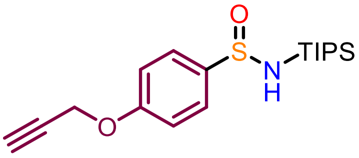

Prepared according to the **General Procedure**, with thianthrenium salt **1f** (86.8 mg, 0.20 mmol, 1.0 equiv.), NEt_3 (0.14 mL, 1.0 mmol, 5.0 equiv.), TIPS-NSO **2a** (90 µL, 0.40 mmol, 2.0 equiv.) and MeCN (2.0 mL). After stirring for 48 h, purification by silica gel flash chromatography (pentane: EtOAc 4:1 to 3:1) afforded **3f** as an off-white solid (36.9 mg, 53%).

Rf = 0.2 (20% EtOAc in pentane); **^{1}H NMR** (400 MHz, $CDCl_3$) δ 7.71 – 7.63 (m, 2H), 7.13 – 7.03 (m, 2H), 4.74 (d, *J* = 2.4 Hz, 2H), 3.66 (s, 1H), 2.54 (t, *J* = 2.4 Hz, 1H), 1.26 (ddt, *J* = 13.3, 8.8, 6.5 Hz, 3H), 1.14 (t, *J* = 6.7 Hz, 18H); **^{13}C NMR** (101 MHz, $CDCl_3$) δ 159.5, 142.8, 126.9, 115.4, 78.0, 76.2, 56.1, 18.1, 18.0, 11.9; [note: for methyl groups in TIPS group, 2 peaks were found instead of 1 due to loss of symmetry caused by chiral sulfur atom]; **IR** (ν_{max}, cm^{-1}) 3300, 2945, 2868, 2361, 2342, 1593, 1493, 1463, 1385, 1303, 1224, 1176, 1088, 1064, 1025, 8801, 765; **HRMS** (ESI^+, *m/z*) calcd. for $[C_{18}H_{30}NO_2SSi]^+$ $[M+H]^+$: 352.1761; found: 352.1752; **MP**: 78 – 80 °C (CH_2Cl_2).

***N*-(4-(((Triisopropylsilyl)amino)sulfinyl)phenyl)benzamide 3g**

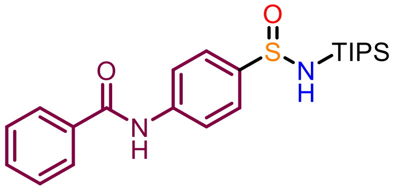

Prepared according to the **General Procedure**, with thianthrenium salt **1g** (99.8 mg, 0.20 mmol, 1.0 equiv.), NEt_3 (0.14 mL, 1.0 mmol, 5.0 equiv.), TIPS-NSO **2a** (90 µL, 0.40 mmol, 2.0 equiv.) and MeCN (2.0 mL). After stirring for 24 h, purification by silica gel flash chromatography (pentane: EtOAc 4:1 to 1:1) afforded **3g** as an off-white solid (30.8 mg, 37%).

R_f = 0.13 (30% EtOAc in pentane); **^{1}H NMR** (400 MHz, DMSO-d_6) δ 10.47 (s, 1H), 7.96 (t, J = 8.9 Hz, 4H), 7.67 – 7.51 (m, 5H), 6.37 (s, 1H), 1.26 – 1.14 (m, 3H), 1.10 (t, J = 6.7 Hz, 18H): **^{13}C NMR** (101 MHz, DMSO-d_6) δ 165.8, 144.4, 140.9, 134.7, 131.8, 128.4, 127.7, 125.7, 120.1, 17.9, 17.8, 11.4; [note: for methyl groups in TIPS group, 2 peaks were found instead of 1 due to loss of symmetry caused by chiral sulfur atom]; **IR** (ν_{max}, cm^{-1}) 3301, 2944, 2867, 1664, 1592, 1530, 1497, 1397, 1319, 1056, 879, 760, 707; **HRMS** (ESI$^+$, *m/z*) calcd. for $[C_{22}H_{33}N_2O_2SSi]^+$ $[M+H]^+$ 417.2027; found: 417.2043; **MP**: 165 – 166 °C (CH_2Cl_2).

4-(4-(2,2,2-Trifluoroacetyl)piperazin-1-yl)-*N*-(triisopropylsilyl)benzenesulfinamide 3h

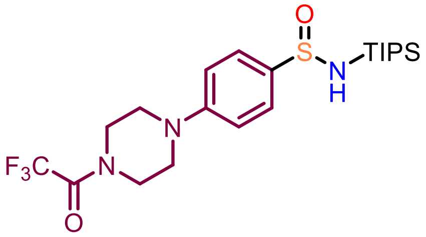

Prepared according to the **General Procedure**, with thianthrenium salt **1h** (112.0 mg, 0.20 mmol, 1.0 equiv.), NEt_3 (0.14 mL, 1.0 mmol, 5.0 equiv.), TIPS-NSO **2a** (90 µL, 0.40 mmol, 2.0 equiv.) and MeCN (2.0 mL). After stirring for 24 h, purification by silica gel flash chromatography (pentane: EtOAc 4:1 to 1:1) afforded **3h** as an off-white solid (42.1 mg, 44%).

Rf = 0.39 (50% EtOAc in pentane); **^{1}H NMR** (400 MHz, $CDCl_3$) δ 7.62 (d, J = 8.6 Hz, 2H), 6.98 (d, J = 8.6 Hz, 2H), 3.87 – 3.79 (m, 2H), 3.77 (t, J = 5.1 Hz, 2H), 3.63 (s, 1H), 3.33 (t, J = 5.2 Hz, 4H), 1.31 – 1.17 (m, 3H), 1.13 (t, J = 6.7 Hz, 18H); **^{13}C NMR** (101 MHz, $CDCl_3$) δ 155.6 (q, J = 36.3 Hz), 152.1, 141.2, 126.6, 116.5 (q, J = 287.7 Hz), 116.1, 48.9, 48.4, 45.5 (q, J = 3.0 Hz), 43.12, 18.1, 18.0, 11.9; [note: for methyl groups in TIPS group, 2 peaks were found instead of 1 due to loss of symmetry caused by chiral sulfur atom]; **^{19}F NMR** (377 MHz, $CDCl_3$) δ -68.9 (s); **IR** (ν_{max}, cm^{-1}); 2944, 2867, 2360, 2342, 1694, 1593, 1501, 1453, 1197, 1145, 1019, 882; **HRMS** (ESI$^+$, *m/z*) calcd. for $[C_{21}H_{35}F_3N_3O_2SSi]^+$ $[M+H]^+$; 478.2166; found: 478.2161; **MP**: 136 – 138 °C (CH_2Cl_2).

4-Cyano-*N*-(triisopropylsilyl)benzenesulfinamide 3i

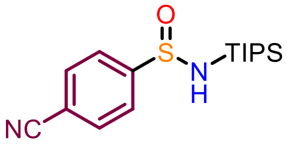

Prepared according to the **General Procedure**, with thianthrenium salt **1i** (81.0 mg, 0.20 mmol, 1.0 equiv.), NEt_3 (0.14 mL, 1.0 mmol, 5.0 equiv.), TIPS-NSO **2a** (90 µL, 0.40 mmol, 2.0 equiv.) and

MeCN (2.0 mL). After stirring for 24 h, purification by silica gel flash chromatography (pentane: EtOAc 4:1) afforded **3i** as an off-white solid (28.6 mg, 44%).

Rf = 0.19 (20% EtOAc in pentane); **^{1}H NMR** (400 MHz, $CDCl_3$) δ 7.86 (d, *J* = 8.3 Hz, 2H), 7.79 (d, *J* = 8.4 Hz, 2H), 3.72 (s, 1H), 1.36 – 1.20 (m, 3H), 1.14 (dd, *J* = 7.3, 5.3 Hz, 18H); **^{13}C NMR** (101 MHz, $CDCl_3$) δ 155.1, 132.9, 126.1, 118.1, 114.7, 18.1, 17.9, 11.9 [note: for methyl groups in TIPS group, 2 peaks were found instead of 1 due to loss of symmetry caused by chiral sulfur atom]; **HRMS** (ESI^+, *m/z*) calcd. for $[C_{16}H_{27}N_2OSSi]^+$ $[M+H]^+$: 323.1608; found: 323.1612.

Data for this compound is in accordance with the previous literature.[16]

3,5-bis(Trifluoromethyl)-*N*-(triisopropylsilyl)benzenesulfinamide 3j

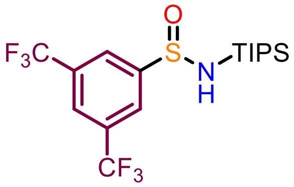

Prepared according to the **General Procedure**, with thianthrenium salt **1j** (103.2 mg, 0.20 mmol, 1.0 equiv.), NEt_3 (0.14 mL, 1.0 mmol, 5.0 equiv.), TIPS-NSO **2a** (90 µL, 0.40 mmol, 2.0 equiv.) and MeCN (2.0 mL). After stirring for 24 h, purification by silica gel flash chromatography (pentane: Et_2O 10:1 to 9:1) afforded **3j** as an off-white solid (41.5 mg, 48%).

Rf = 0.45 (10% EtOAc in pentane); **^{1}H NMR** (400 MHz, $CDCl_3$) δ 8.20 (s, 2H), 7.97 (s, 1H), 3.78 (s, 1H), 1.37 – 1.22 (m, 3H), 1.16 (dd, *J* = 7.4, 4.7 Hz, 18H); **^{13}C NMR** (101 MHz, $CDCl_3$) δ 153.4, 132.7 (q, *J* = 34.2 Hz), 126.0 (q, *J* = 3.2 Hz), 124.7 (q, *J* = 3.6 Hz), 123.0 (q, *J* = 273.4 Hz), 18.0, 17.9, 11.9; [note: for methyl groups in TIPS group, 2 peaks were found instead of 1 due to loss of symmetry caused by chiral sulfur atom]; **^{19}F NMR** (377 MHz, $CDCl_3$) δ -63.0 (s); **IR** (ν_{max}, cm^{-1}) 3088, 2873, 2349, 1728, 1358, 1280, 1189, 1132, 913; **HRMS** (ESI^+, *m/z*) calcd. for $[C_{17}H_{26}F_6NOSSi]^+$ $[M+H]^+$: 434.1403; found: 434.1405; **MP**: 138 – 140 °C (CH_2Cl_2).

4-Allyl-2-methoxy-*N*-(triisopropylsilyl)benzenesulfinamide 3k

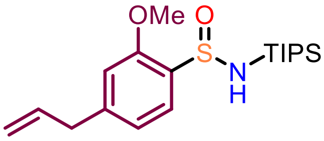

Prepared according to the **General Procedure A**, with thianthrenium salt **1k** (90.0 mg, 0.20 mmol, 1.0 equiv.), NEt_3 (0.14 mL, 1.0 mmol, 5.0 equiv.), TIPS-NSO **2a** (90 µL, 0.40 mmol, 2.0 equiv.) and

MeCN (2.0 mL). After stirring for 24 h, purification by silica gel flash chromatography (pentane: EtOAc 5:1 to 4:1) afforded **3k** as an off-white solid (37.4 mg, 51%).

Rf = 0.25 (20% EtOAc in pentane); **^{1}H NMR** (400 MHz, $CDCl_3$) δ 7.68 (d, *J* = 2.3 Hz, 1H), 7.22 (dd, *J* = 8.4, 2.3 Hz, 1H), 6.86 (d, *J* = 8.3 Hz, 1H), 5.94 (ddt, *J* = 16.9, 10.1, 6.7 Hz, 1H), 5.13 – 5.02 (m, 2H), 4.05 (s, 1H), 3.85 (s, 3H), 3.38 (d, *J* = 6.7 Hz, 2H), 1.27 – 1.17 (m, 3H), 1.10 (dd, *J* = 7.3, 4.5 Hz, 18H); **^{13}C NMR** (101 MHz, $CDCl_3$) δ 154.4, 137.3, 137.2, 132.9, 132.1, 125.0, 116.3, 111.4, 55.5, 39.5, 18.0, 17.9, 12.0; [note: for methyl groups in TIPS group, 2 peaks were found instead of 1 due to loss of symmetry caused by chiral sulfur atom]; **IR** (ν_{max}, cm^{-1}) 3111, 2942, 2866, 1492, 1463, 1273, 1068, 882, 770, 677; **HRMS** (ESI$^+$, *m/z*) calcd. for $[C_{19}H_{34}NO_2SSi]^+$ $[M+H]^+$ 368.2074; found: 368.2075; **MP**: 88 – 89 °C (CH_2Cl_2).

3-Cyano-4-methoxy-*N*-(triisopropylsilyl)benzenesulfinamide 3l

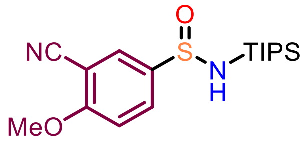

Prepared according to the **General Procedure**, with thianthrenium salt **1l** (87.0 mg, 0.20 mmol, 1.0 equiv.), NEt_3 (0.14 mL, 1.0 mmol, 5.0 equiv.), TIPS-NSO **2a** (90 µL, 0.40 mmol, 2.0 equiv.) and MeCN (2.0 mL). After stirring for 24 h, purification by silica gel flash chromatography (pentane: EtOAc 3:1 to 1:1) afforded **3l** as an off-white solid (31.2 mg, 44%).

Rf = 0.27 (50% EtOAc in pentane); **^{1}H NMR** (400 MHz, $CDCl_3$) δ 7.93 – 7.85 (m, 2H), 7.12 – 7.05 (m, 1H), 3.99 (s, 3H), 3.72 (s, 1H), 1.26 (ddt, *J* = 13.3, 8.7, 6.5 Hz, 3H), 1.14 (dd, *J* = 7.3, 6.1 Hz, 18H); **^{13}C NMR** (101 MHz, $CDCl_3$) δ 163.0, 142.6, 131.6, 131.1, 115.6, 111.8, 102.7, 56.7, 18.1, 17.9, 11.9; [note: for methyl groups in TIPS group, 2 peaks were found instead of 1 due to loss of symmetry caused by chiral sulfur atom]; **IR** (ν_{max}, cm^{-1}) 3069, 2943, 2867, 2230, 1598, 1491, 1462, 1282, 1264, 1063, 1044, 1020, 900, 882; **HRMS** (ESI$^+$, *m/z*) calcd. for $[C_{17}H_{29}N_2O_2SSi]^+$ $[M+H]^+$ 353.1714; found: 353.1721; **MP**: 124 – 127 °C (CH_2Cl_2).

Methyl 2-Methoxy-5-(((triisopropylsilyl)amino)sulfinyl)benzoate 3m

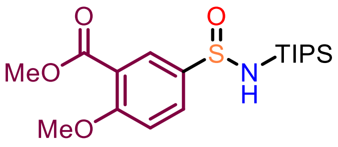

Prepared according to the **General Procedure**, with thianthrenium salt **1m** (93.6 mg, 0.20 mmol, 1.0 equiv.), NEt_3 (0.14 mL, 1.0 mmol, 5.0 equiv.), TIPS-NSO **2a** (90 µL, 0.40 mmol, 2.0 equiv.) and MeCN (2.0 mL). After stirring for 24 h, purification by silica gel flash chromatography (pentane: EtOAc 2:1 to 1:1) afforded **3m** as an off-white solid (37.5 mg, 49%).

R_f = 0.44 (50% EtOAc in pentane); **^{1}H NMR** (400 MHz, $CDCl_3$) δ 8.16 (d, *J* = 2.4 Hz, 1H), 7.84 (dd, *J* = 8.7, 2.5 Hz, 1H), 7.09 (d, *J* = 8.8 Hz, 1H), 3.96 (s, 3H), 3.89 (s, 3H), 3.66 (s, 1H), 1.29 – 1.24 (m, 3H), 1.15 (t, *J* = 6.7 Hz, 18H); **^{13}C NMR** (101 MHz, $CDCl_3$) δ 165.7, 161.1, 141.6, 130.5, 129.1, 120.5, 112.5, 56.5, 52.4, 18.1, 18.0, 12.0; [note: for methyl groups in TIPS group, 2 peaks were found instead of 1 due to loss of symmetry caused by chiral sulfur atom]; **IR** (ν_{max}, cm^{-1}) 2947, 1730, 1489, 1278, 1080, 880, 759; **HRMS** (ESI^+, *m/z*) calcd. for $[C_{18}H_{32}NO_4SSi]^+$ $[M+H]^+$: 386.1816; found: 386.1823; **MP**: 106 – 108 °C (CH_2Cl_2).

***N*-(Triisopropylsilyl)-2,3-dihydrobenzo[*b*][1,4]dioxine-6-sulfinamide 3n**

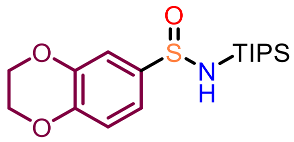

Prepared according to the **General Procedure**, with thianthrenium salt **1n** (87.6 mg, 0.20 mmol, 1.0 equiv.), NEt_3 (0.14 mL, 1.0 mmol, 5.0 equiv.), TIPS-NSO **2a** (90 µL, 0.40 mmol, 2.0 equiv.) and MeCN (2.0 mL). After stirring for 24 h, purification by silica gel flash chromatography (pentane: EtOAc 8:1 to 4:1) afforded **3n** as an off-white solid (41.5 mg, 0.12 mmol, 58%).

Rf = 0.14 (20% EtOAc in pentane); **^{1}H NMR** (400 MHz, $CDCl_3$) δ 7.23 (d, *J* = 2.1 Hz, 1H), 7.17 (dd, *J* = 8.4, 2.1 Hz, 1H), 6.94 (d, *J* = 8.4 Hz, 1H), 4.27 (s, 4H), 3.67 (s, 1H), 1.25 (dt, *J* = 14.6, 7.4 Hz, 3H), 1.12 (t, *J* = 6.9 Hz, 18H); **^{13}C NMR** (101 MHz, $CDCl_3$) δ 145.7, 143.9, 142.9, 118.3, 117.8, 114.4, 64.5, 64.4, 18.1, 18.0, 11.9; [note: for methyl groups in TIPS group, 2 peaks were found instead of 1 due to loss of symmetry caused by chiral sulfur atom]; **IR** (ν_{max}, cm^{-1}) 2943, 2867, 2360, 2340, 1583, 1492, 1462, 1311, 1283, 1250, 1063, 876, 762; **HRMS** (ESI^+, *m/z*) calcd. for $[C_{17}H_{30}NO_3SSi]^+$ $[M+H]^+$ 356.1710; found: 356.1721; **MP**: 92 – 93 °C (CH_2Cl_2).

8-Butoxy-*N*-(triisopropylsilyl)quinoline-5-sulfinamide 3o

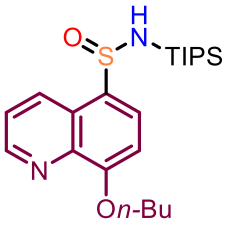

Prepared according to the **General Procedure**, with thianthrenium salt **1o** (100.6 mg, 0.20 mmol, 1.0 equiv.), NEt_3 (0.14 mL, 1.0 mmol, 5.0 equiv.), TIPS-NSO **2a** (90 µL, 0.40 mmol, 2.0 equiv.) and MeCN (2.0 mL). After stirring for 24 h, purification by silica gel flash chromatography (pentane: EtOAc 9:1 to 1:1) afforded **3o** as an off-white solid (37.4 mg, 44%).

R_f = 0.31 (50% EtOAc in pentane); **^{1}H NMR** (400 MHz, $CDCl_3$) δ 9.03 – 8.97 (m, 1H), 8.66 (dd, *J* = 8.6, 1.6 Hz, 1H), 8.15 (d, *J* = 8.3 Hz, 1H), 7.48 (dd, *J* = 8.6, 4.2 Hz, 1H), 7.13 (d, *J* = 8.3 Hz, 1H), 4.28 (t, *J* = 7.0 Hz, 2H), 3.79 (s, 1H), 2.07 – 1.95 (m, 2H), 1.64 – 1.50 (m, 2H), 1.30 – 1.22 (m, 3H), 1.11 (t, *J* = 7.6 Hz, 18H), 1.01 (t, *J* = 7.4 Hz, 3H); **^{13}C NMR** (101 MHz, $CDCl_3$) δ 157.4, 149.8, 140.4, 136.4, 131.6, 125.5, 123.8, 121.9, 107.6, 69.2, 31.0, 19.4, 18.1, 18.0, 14.0, 12.1; [note: for methyl groups in TIPS group, 2 peaks were found instead of 1 due to loss of symmetry caused by chiral sulfur atom]; **IR** (ν_{max}, cm^{-1}) 2958, 2943, 2867, 2360, 1566, 1500, 1463, 1370, 1309, 1242, 1097, 865; **HRMS** (ESI$^+$, *m/z*) calcd. for $[C_{22}H_{37}N_2O_2SSi]^+$ $[M+H]^+$: 421.2340; found: 421.2348; **MP**: 117 – 120 °C (CH_2Cl_2).

6-Methoxy-*N*-(triisopropylsilyl)pyridine-3-sulfinamide 3p

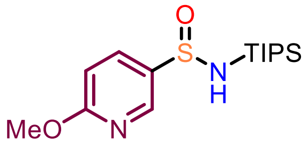

Prepared according to the **General Procedure**, with thianthrenium salt **1p** (82.2 mg, 0.20 mmol, 1.0 equiv.), NEt_3 (0.14 mL, 1.0 mmol, 5.0 equiv.), TIPS-NSO **2a** (90 µL, 0.40 mmol, 2.0 equiv.) and MeCN (2.0 mL). After stirring for 48 h, purification by silica gel flash chromatography (pentane: EtOAc 4:1 to 2:1) afforded **3p** as an off-white solid (32.0 mg, 49%).

Rf = 0.25 (20% EtOAc in pentane); **^{1}H NMR** (400 MHz, $CDCl_3$) δ 8.45 (dd, *J* = 2.6, 0.7 Hz, 1H), 7.89 (dd, *J* = 8.7, 2.5 Hz, 1H), 6.82 (d, *J* = 8.7 Hz, 1H), 3.97 (s, 3H), 3.74 (s, 1H), 1.26 (ddt, *J* = 13.4, 8.8, 6.4 Hz, 3H), 1.14 (t, *J* = 7.2 Hz, 18H); **^{13}C NMR** (101 MHz, $CDCl_3$) δ 165.8, 145.2, 138.9, 136.0, 111.5, 54.1, 18.1, 18.0, 11.9; [note: for methyl groups in TIPS group, 2 peaks were found instead of

1 due to loss of symmetry caused by chiral sulfur atom]; **HRMS** (ESI$^+$, *m/z*) calcd. for [$C_{15}H_{29}N_2O_2$ SSi]$^+$ [M+H]$^+$ 329.1714; found: 329.1720.

Data for this compound is in accordance with the previous literature.[16]

9-Oxo-*N*-(triisopropylsilyl)-9*H*-xanthene-2-sulfinamide 3q

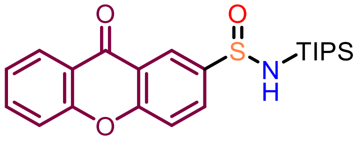

Prepared according to the **General Procedure**, with thianthrenium salt **1q** (99.6 mg, 0.20 mmol, 1.0 equiv.), NEt_3 (0.14 mL, 1.0 mmol, 5.0 equiv.), TIPS-NSO **2a** (90 µL, 0.40 mmol, 2.0 equiv.) and MeCN (2.0 mL). After stirring for 24 h, purification by silica gel flash chromatography (pentane: EtOAc 5:1 to 4:1) afforded **3q** as an off-white solid (33.9 mg, 41%).

R_f = 0.09 (20% EtOAc in pentane); **^{1}H NMR** (400 MHz, $CDCl_3$) δ 8.65 (d, *J* = 2.3 Hz, 1H), 8.33 (dd, *J* = 7.9, 1.7 Hz, 1H), 8.13 (dd, *J* = 8.7, 2.4 Hz, 1H), 7.80 – 7.71 (m, 1H), 7.62 (d, *J* = 8.8 Hz, 1H), 7.52 (d, *J* = 8.4 Hz, 1H), 7.42 (t, *J* = 7.6 Hz, 1H), 3.86 (s, 1H), 1.30 (ddd, *J* = 20.3, 13.9, 5.9 Hz, 3H), 1.18 (t, *J* = 6.6 Hz, 18H); **^{13}C NMR** (101 MHz, $CDCl_3$) δ 176.5, 157.4, 156.2, 146.1, 135.4, 131.3, 126.9, 124.7, 124.5, 122.0, 121.9, 119.3, 118.2, 18.1, 18.0, 12.0; [note: for methyl groups in TIPS group, 2 peaks were found instead of 1 due to loss of symmetry caused by chiral sulfur atom]; **IR** (ν_{max}, cm^{-1}) 3058, 2942, 2866, 1662, 1615, 1468, 1315, 1061, 1042, 902, 880, 756; **HRMS** (ESI$^+$, *m/z*) calcd. for [$C_{22}H_{30}NO_3SSi$]$^+$ [M+H]$^+$: 416.1710; found: 416.1713; **MP**: 166 – 170 °C (CH_2Cl_2).

Methyl 2-(2-Fluoro-4’-(((triisopropylsilyl)amino)sulfinyl)-[1,1’-biphenyl]-4-yl)propanoate 3r

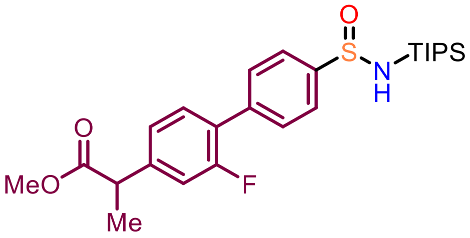

Prepared according to the **General Procedure**, with thianthrenium salt **1r** (112.0 mg, 0.20 mmol, 1.0 equiv.), NEt_3 (0.14 mL, 1.0 mmol, 5.0 equiv.), TIPS-NSO **2a** (90 µL, 0.40 mmol, 2.0 equiv.) and MeCN (2.0 mL). After stirring for 24 h, purification by silica gel flash chromatography (pentane: EtOAc 5:1 to 4:1) afforded **3r** as an off-white solid (53.4 mg, 56%).

Rf = 0.14 (20% EtOAc in pentane); **^{1}H NMR** (400 MHz, $CDCl_3$) δ 7.82 – 7.76 (m, 2H), 7.65 (dd, *J* = 8.4, 1.6 Hz, 2H), 7.40 (t, *J* = 8.0 Hz, 1H), 7.19 – 7.10 (m, 2H), 3.80 – 3.74 (m, 2H), 3.70 (s, 3H), 1.54 (d, *J* = 7.2 Hz, 3H), 1.35 – 1.23 (m, 3H), 1.16 (t, *J* = 7.3 Hz, 18H); **^{13}C NMR** (101 MHz, $CDCl_3$) δ 174.4, 159.8 (d, *J* = 249.2 Hz), 149.4, 142.8 (d, *J* = 7.8 Hz), 138.1, 130.9 (d, *J* = 3.6 Hz), 129.6 (d, *J* = 3.1 Hz), 126.8 (d, *J* = 13.5 Hz), 125.4, 123.9 (d, *J* = 3.3 Hz), 115.6 (d, *J* = 23.4 Hz), 52.4, 45.1, 18.5, 18.1, 18.0, 12.0; [note: for methyl groups in TIPS group, 2 peaks were found instead of 1 due to loss of symmetry caused by chiral sulfur atom]; **^{19}F NMR** (377 MHz, $CDCl_3$) δ -117.2 – -117.3 (m); [note: isolated as 1:1 diastereomeric mixture, no peak separation in ^{1}H, ^{13}C and ^{19}F NMR]; **IR** (ν_{max}, cm^{-1}) 2945, 2867, 2359, 2337, 1739, 1624, 1577, 1461, 1429, 1391, 1199, 1173, 1068, 1006, 921, 877, 770; **HRMS** (ESI$^+$, *m/z*) calcd. for $[C_{25}H_{37}FNO_3SSi]^+$ $[M+H]^+$ 478.2242; found: 478.2256; **MP**: 98 – 101 °C (CH_2Cl_2).

4-(4-(2-(Pyridine-2-yloxy)propoxy)phenoxy)-*N*-(triisopropylsilyl)benzenesulfinamide 3s

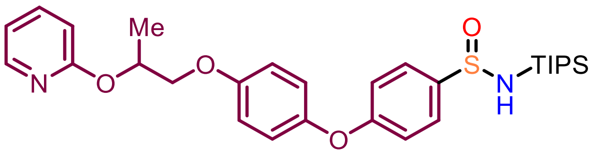

Prepared according to the **General Procedure**, with thianthrenium salt **1s** (124.6 mg, 0.20 mmol, 1.0 equiv.), NEt_3 (0.14 mL, 1.0 mmol, 5.0 equiv.), TIPS-NSO **2a** (90 µL, 0.40 mmol, 2.0 equiv.) and MeCN (2.0 mL). After stirring for 24 h, purification by silica gel flash chromatography (pentane: EtOAc 9:1 to 7:3) afforded **3s** as a dark-brown oil (60.8 mg, 56%).

Rf = 0.27 (20% EtOAc in pentane); **^{1}H NMR** (400 MHz, $CDCl_3$) δ 8.18 – 8.12 (m, 1H), 7.67 – 7.61 (m, 2H), 7.60 – 7.52 (m, 1H), 7.03 – 6.90 (m, 6H), 6.89 – 6.82 (m, 1H), 6.77 – 6.71 (m, 1H), 5.61 – 5.57 (m, 1H), 4.14 (ddd, *J* = 46.4, 9.9, 5.1 Hz, 2H), 3.67 (s, 1H), 1.48 (d, *J* = 6.4 Hz, 3H), 1.27 (dtd, *J* = 14.2, 7.2, 1.5 Hz, 3H), 1.14 (t, *J* = 6.9 Hz, 18H); **^{13}C NMR** (101 MHz, $CDCl_3$) δ 163.3, 161.1, 156.0, 149.2, 146.9, 143.4, 138.8, 126.9, 121.6, 117.3, 116.9, 116.1, 111.8, 71.2, 69.3, 18.1, 18.0 17.1, 11.9 [note: for methyl groups in TIPS group, 2 peaks were found instead of 1 due to loss of symmetry caused by chiral sulfur atom]; [note: isolated as 1:1 diastereomeric mixture, no peak separation in ^{1}H and ^{13}C NMR]; **IR** (ν_{max}, cm^{-1}) 2943, 2861, 2360, 1595, 1571, 1504, 1471, 1432, 1286, 1226, 1086, 875, 778; **HRMS** (ESI$^+$, *m/z*) calcd. for $[C_{29}H_{41}N_2O_4SSi]^+$ $[M+H]^+$ 541.2551; found: 541.2551.

4-Fluoro-*N*-(2-phenylpropan-2-yl)benzenesulfinamide 3t

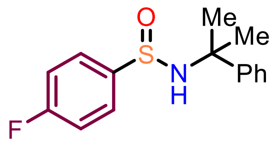

Prepared according the **General Procedure**, with thianthrenium salt **1a** (79.6 mg, 0.20 mmol, 1.0 equiv.), NEt_3 (0.14 mL, 1.0 mmol, 5.0 equiv.), Cumyl-NSO **2b** (72.4 mg, 0.40 mmol, 2.0 equiv.) and MeCN (2.0 mL). After stirring for 24 h, purification by silica gel flash chromatography (pentane: EtOAc 4:1 to 2:1) afforded **3t** as a waxy light-brown oil (28.0 mg, 51%).

Rf = 0.12 (20% EtOAc in pentane); ^{1}**H NMR** (400 MHz, $CDCl_3$) δ 7.76 – 7.66 (m, 2H), 7.58 – 7.50 (m, 2H), 7.43 – 7.33 (m, 2H), 7.32 – 7.26 (m, 1H), 7.23 – 7.12 (m, 2H), 4.19 (s, 1H), 1.86 (s, 3H), 1.76 (s, 3H); ^{13}**C NMR** (101 MHz, $CDCl_3$) δ 164.4 (d, *J* = 250.9 Hz), 146.5, 142.2 (d, *J* = 2.8 Hz), 128.6, 128.0 (d, *J* = 9.0 Hz), 127.5, 125.9, 116.1 (d, *J* = 22.3 Hz), 59.2, 32.1, 30.3 [note: for methyl groups in cumyl group, 2 peaks were found instead of 1 due to loss of symmetry caused by chiral sulfur atom]; ^{19}**F NMR** (377 MHz, $CDCl_3$) δ -109.7 (tt, *J* = 8.3, 5.2 Hz); **IR** (ν_{max}, cm^{-1}) 3184, 2979, 2854, 1589, 1490, 1228, 1153, 1086, 1058, 833, 765, 700; **HRMS** (ESI^+, *m/z*) calcd. for $[C_{15}H_{17}FNOS]^+$ $[M+H]^+$ 278.1009; found: 278.1009.

4-Fluoro-*N*-(2,4,4-trimethylpentan-2-yl)benzenesulfinamide 3u

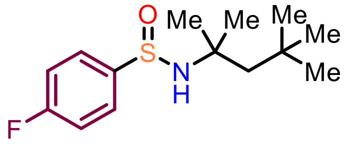

Prepared according to the **General Procedure**, with thianthrenium salt **1a** (79.6 mg, 0.20 mmol, 1.0 equiv.), NEt_3 (0.14 mL, 1.0 mmol, 5.0 equiv.), *t*-Oct-NSO **2c** (70.0 mg, 0.40 mmol, 2.0 equiv.) and MeCN (2.0 mL). After stirring for 24 h, purification by silica gel flash chromatography (pentane: EtOAc 9:1 to 7:3) afforded **3u** as an off-white solid (25.5 mg, 47%).

$\mathbf{R_f}$ = 0.36 (30% EtOAc in pentane); ^{1}**H NMR** (400 MHz, $CDCl_3$) δ 7.73 – 7.63 (m, 2H), 7.16 (t, *J* = 8.6 Hz, 2H), 3.84 (s, 1H), 1.71 – 1.55 (m, 2H), 1.52 (s, 3H), 1.47 (s, 3H), 1.02 (s, 9H); ^{13}**C NMR** (101 MHz, $CDCl_3$) δ 164.3 (d, *J* = 250.7 Hz), 142.3 (d, *J* = 3.0 Hz), 128.0 (d, *J* = 8.7 Hz), 116.0 (d, *J* = 22.7 Hz), 58.6, 56.4, 32.6, 32.0, 31.9, 29.4; [note: for methyl carbons in *t*-Octyl group, 2 peaks were found instead of 1 due to loss of symmetry caused by chiral sulfur atom]; ^{19}**F NMR** (377 MHz, $CDCl_3$) δ -110.0 – -110.1 (m); **IR** (ν_{max}, cm^{-1}) 3188, 2954, 2905, 1589, 1491, 1368, 1229, 1152, 1087,

1059, 835; **HRMS** (ESI$^+$, *m/z*) calcd. for $[C_{14}H_{23}FNOS]^+$ $[M+H]^+$: 272.1479; found: 272.1478; **MP**: 68 – 69 °C (CH_2Cl_2).

4-Fluoro-*N*-tritylbenzenesulfinamide 3v

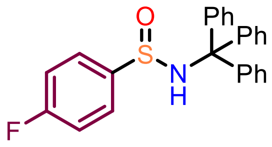

Prepared according to the **General Procedure**, with thianthrenium salt **1a** (79.6 mg, 0.20 mmol, 1.0 equiv), NEt_3 (0.14 mL, 1.0 mmol, 5.0 equiv.), Tr-NSO **2d** (122.0 mg, 0.40 mmol, 2.0 equiv.) and MeCN (2.0 mL). After stirring for 48 h, purification by silica gel flash chromatography (pentane: acetone 49:1 to 9:1) afforded **3v** as an off-white solid (51.1 mg, 64%).

Rf = 0.27 (10% acetone in pentane); **^{1}H NMR** (400 MHz, $CDCl_3$) δ 7.70 – 7.61 (m, 2H), 7.39 – 7.28 (m, 15H), 7.20 – 7.11 (m, 2H), 5.22 (s, 1H); **^{13}C NMR** (101 MHz, $CDCl_3$) δ 164.4 (d, *J* = 251.1 Hz), 144.6, 142.9 (d, *J* = 2.8 Hz), 129.6, 128.2, 128.1 (d, *J* = 8.7 Hz), 127.7, 116.2 (d, *J* = 22.3 Hz), 73.7; **^{19}F NMR** (377 MHz, $CDCl_3$) δ -109.4 – -109.5 (m); **HRMS** (ESI$^+$, *m/z*) calcd. for $[C_{25}H_{20}FNOSNa]^+$ $[M+Na]^+$ 424.1142; found: 424.1142.

Data for this compound is in accordance with the previous literature.[17]

4-Fluoro-*N*-phenylbenzenesulfinamide 3w

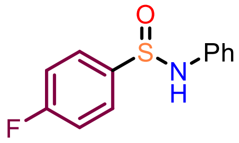

Prepared according to the **General Procedure**, with thianthrenium salt **1a** (79.6 mg, 0.20 mmol, 1.0 equiv), NEt_3 (0.14 mL, 1.0 mmol, 5.0 equiv.), Ph-NSO **2e** (55.6 mg, 0.40 mmol, 2.0 equiv.) and MeCN (2.0 mL). After stirring for 48 h, purification by silica gel flash chromatography (pentane: EtOAc 9:1 to 4:1) afforded **3w** as an orange solid (14.9 mg, 32%).

Rf = 0.24 (20% EtOAc in pentane); **^{1}H NMR** (400 MHz, $CDCl_3$) δ 7.80 (ddd, *J* = 8.9, 5.0, 1.6 Hz, 2H), 7.37 – 7.27 (m, 2H), 7.24 (td, *J* = 8.5, 1.6 Hz, 2H), 7.14 – 7.07 (m, 3H), 6.21 (s, 1H); **^{13}C NMR** (101 MHz, $CDCl_3$) δ 164.8 (d, *J* = 252.4 Hz), 140.4, 140.3 (d, *J* = 2.4 Hz), 129.7, 128.1 (d, *J* = 8.9

Hz), 124.2, 119.5, 116.5 (d, $J = 22.7$ Hz); **^{19}F NMR** (377 MHz, $CDCl_3$) δ -108.2 – -108.4 (m); **HRMS** (ESI^+, *m/z*) calcd. for $[C_{12}H_{11}FNOS]^+$ $[M+H]^+$ 236.0540; found: 236.0545.

Data for this compound is in accordance with the previous literature.[18]

4.3 Telescoped one-pot sulfinamidation procedures for sulfinamides 3b and 3s

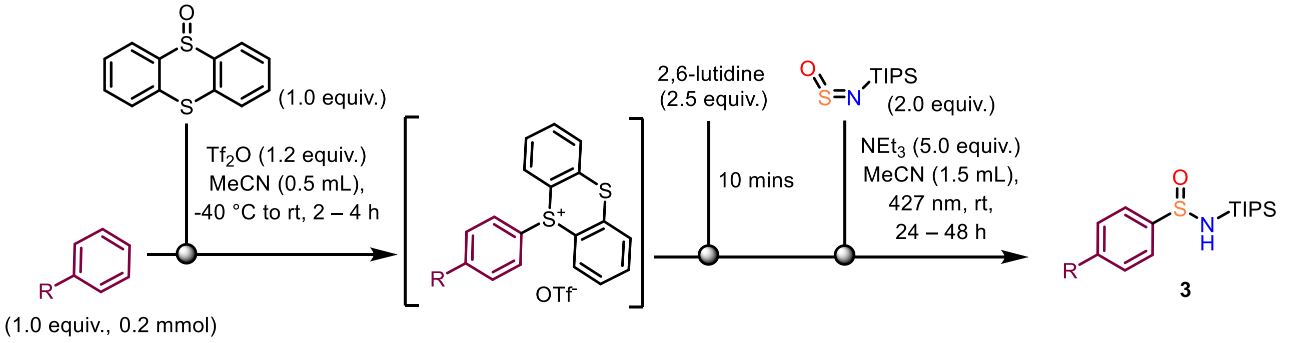

General Telescoped Procedure: An oven-dried microwave vial containing thianthrene-5-oxide **S1** (0.2 mmol, 1.0 equiv.) and arene (0.2 mmol, 1.0 equiv.) (if solid), was sealed and subjected to three evacuation/N_2 refill cycles before anhydrous, degassed MeCN (0.4 M, 0.5 mL) and arene (0.2 mmol, 1.0 equiv.) (if liquid) were added under N_2. After stirring for 1 minute, ensuring all solids had dissolved, the vial was then cooled to -40 °C and Tf_2O (0.24 mmol, 1.2 equiv.) was added dropwise. The mixture was stirred at -40 °C for 1 h, then warmed to rt and stirred for 1 to 3 h (until full consumption of arene and formation of sulfonium salt, as judged by TLC). 2,6-Lutidine (0.5 mmol, 2.5 equiv.) was then added and the reaction mixture stirred for 10 mins at rt. Subsequently, anhydrous degassed MeCN (1.5 mL), NEt_3 (1.0 mmol, 5.0 equiv.) and TIPS-NSO **2a** (0.4 mmol, 2.0 equiv.) were added under N_2. The reaction mixture was stirred for 24 to 48 h under 427 nm irradiation at ambient temperature (31°C, photoreactor fans on). The solution was concentrated *in vacuo* and purified by silica gel flash chromatography **to** afford the sulfinamide product.

4-Methyl-*N*-(triisopropylsilyl)benzenesulfinamide 3b

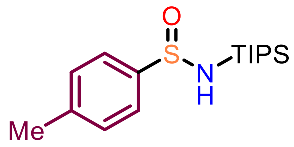

Prepared according to the **General Telescoped Procedure**, with thianthrene-5-oxide **S1** (46.4 mg, 0.2 mmol, 1.0 equiv.), toluene (21.3 µL, 0.2 mmol, 1.0 equiv.) and MeCN (0.5 mL) then Tf_2O (40 µL, 0.24 mmol, 1.2 equiv.), 2,6-Lutidine (58 µL, 0.5 mmol, 2.5 equiv.), MeCN (1.5 mL), NEt_3 (0.14 mL, 1.0 mmol, 5.0 equiv.) and TIPS-NSO **2a** (90 µL, 0.4 mmol, 2.0 equiv.). After stirring for 24 h

under 427 nm irradiation, purification by silica gel flash chromatography (pentane: EtOAc 9:1 to 4:1) afforded **3b** as an off-white solid (25.7 mg, 41%).

4-(4-(2-(Pyridine-2-yloxy)propoxy)phenoxy)-*N*-(triisopropylsilyl)benzenesulfinamide 3s

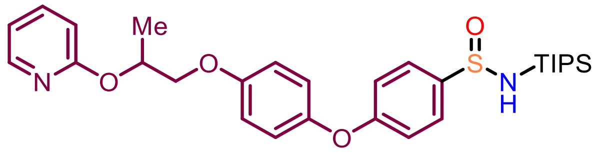

Prepared according to the **General Telescoped Procedure**, with thianthrene-5-oxide **S1** (46.4 mg, 0.2 mmol, 1.0 equiv.), pyriproxyfen (64.3 mg, 0.2 mmol, 1.0 equiv.) and MeCN (0.5 mL) then Tf_2O (40 µL, 0.24 mmol, 1.2 equiv.), 2,6-Lutidine (58 µL, 0.5 mmol, 2.5 equiv.), MeCN (1.5 mL), NEt_3 (0.14 mL, 1.0 mmol, 5.0 equiv.) and TIPS-NSO **2a** (90 µL, 0.4 mmol, 2.0 equiv.). After stirring for 48 h under 427 nm irradiation, purification by silica gel flash chromatography (pentane: EtOAc 9:1 to 4:1) afforded **3s** as a dark brown oil (44.1 mg, 41%).

4.4 Scale up procedure

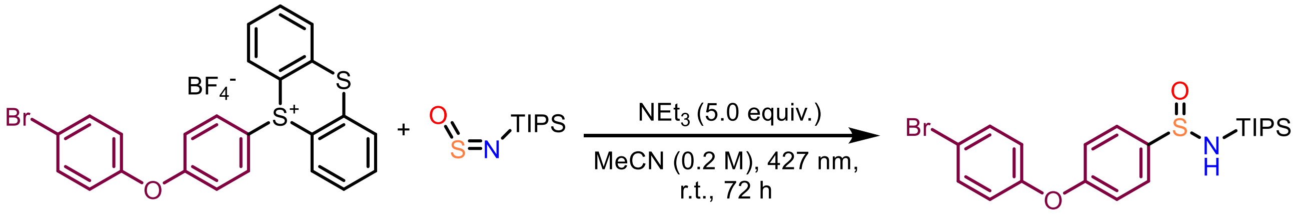

1d (2 mmol, 1.0 equiv.) **2a** (2.0 equiv.) **3d 34%**

An oven-dried 20 mL microwave vial containing tetrafluoroborate thianthrenium salt **1d** (2 mmol, 1.0 equiv.) was sealed and subjected to three evacuation/N_2 refill cycles before anhydrous, degassed MeCN (0.1 M, 10 mL) was added. After stirring for 1 minute, ensuring all solids had dissolved, NEt_3 (1.39 mL, 5.0 equiv.) and TIPS-NSO **2a** (903 µL, 2.0 equiv.) were then added to the reaction mixture under N_2. The reaction was then stirred for 72 h under 427 nm irradiation at ambient temperature (31°C, photoreactor fans on) (See **Figure S1** for photochemical set-up). The reaction mixture was concentrated *in vacuo* and purified by silica gel flash chromatography (pentane: EtOAc 9:1 to 4:1) affording **3d** as a light-orange solid (317.6 mg, 34%).

Note:

1) Ensure very efficient stirring throughout reaction time as solid precipitate will form on sides of vessel over time

4.5 Derivatisation

4-(4-Bromophenoxy)-*N*-(triisopropylsilyl)benzenesulfinamide 4a

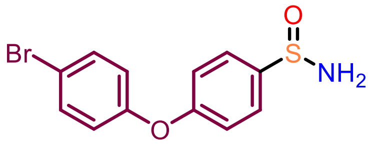

4-(4-Bromophenoxy)-*N*-(triisopropylsilyl)benzenesulfinamide **3d** (46.7 mg, 0.10 mmol, 1.0 equiv.) was added to a 10 mL vial. Then CH_2Cl_2 (0.5 M, 0.2 mL) was added followed by addition of TBAF (1.5 mL, 1 M in THF, 0.15 mmol, 1.5 equiv.). After 1 h, the reaction was concentrated *in vacuo* and purified by column chromatography (pentane: EtOAc 1:1 to 0:1) affording **4a** as a white solid (28.3 mg, 91%).

Rf = 0.39 (50% EtOAc in pentane); **^{1}H NMR** (400 MHz, DMSO-d_6) δ 7.69 – 7.63 (m, 2H), 7.63 – 7.56 (m, 2H), 7.17 – 7.14 (m, 2H), 7.05 – 7.01 (m, 2H), 6.25 (s, 2H); **^{13}C NMR** (101 MHz, DMSO-d_6) δ 158.1, 155.4, 143.0, 133.0, 127.6, 121.4, 118.4, 115.9; **IR** (ν_{max}, cm^{-1}) 3327, 3191, 3093, 2926, 2854, 2360, 1722, 1584, 1485, 1256, 1028, 1009, 818, 762; **HRMS** (ESI$^+$, m/z) calcd. for $[C_{12}H_{10}{}^{79}BrNO_2SNa]^+$ $[M+Na]^+$ 333.9508; found: 333.9515; $[C_{12}H_{10}{}^{81}BrNO_2SNa]^+$ $[M+Na]^+$ 335.9487; found: 335.9413; **MP**: 150 – 152 °C (EtOAc).

4-(4-Bromophenoxy)-*N*-(triisopropylsilyl)benzenesulfonamide 4b

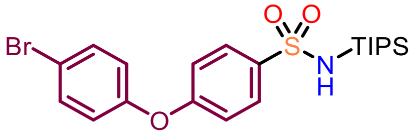

4-(4-Bromophenoxy)-*N*-(triisopropylsilyl)benzenesulfinamide **3d** (46.7 mg, 0.10 mmol, 1.0 equiv.) and *m*-chloroperoxybenzoic acid (60% by weight) (57.5 mg, 0.2 mmol, 2.0 equiv.) were added to a 5 mL vial. CH_2Cl_2 (0.1 M, 1 mL) was added at rt. The reaction was stirred at rt for 30 mins. Then the reaction was diluted with EtOAc (15 mL) and quenched with aq. $Na_2S_2O_3$ (15 mL). After collecting the organic phase, the aqueous phase was extracted with EtOAc (2 × 5 mL). The combined organic phases were dried over $MgSO_4$, filtered and concentrated *in vacuo*. The resulting crude residue was

purified by silica gel flash chromatography (pentane: EtOAc 10:1 to 9:1) affording **4b** as a colourless solid (45.5 mg, 94%).

Rf = 0.35 (10% EtOAc in pentane); **^{1}H NMR** (400 MHz, $CDCl_3$) δ 7.91 – 7.83 (m, 2H), 7.54 – 7.46 (m, 2H), 7.05 – 6.97 (m, 2H), 6.97 – 6.89 (m, 2H), 4.37 (s, 1H), 1.26 (h, *J* = 7.3 Hz, 3H), 1.05 (d, *J* = 7.4 Hz, 18H); **^{13}C NMR** (101 MHz, $CDCl_3$) δ 160.3, 154.9, 138.4, 133.3, 128.5, 121.8, 118.0, 117.5, 18.1, 12.0; **IR** (ν_{max}, cm^{-1}) 2982, 2948, 1726, 1578, 1483, 1291, 1249, 1155, 1120, 936, 731, 638; **HRMS** (ESI^+, *m/z*) calcd. for $[C_{21}H_{31}{}^{79}BrNO_3SSi]^+$ $[M+H]^+$ 484.0972; found: 484.0976; $[C_{21}H_{31}{}^{81}BrNO_3SSi]^+$ $[M+H]^+$ 486.0951; found: 486.0957; **MP**: 130 – 131 °C (CH_2Cl_2).

4-(4-Bromophenoxy)-*N*-(*p*-tolyl)-*N*'-(triisopropylsilyl)benzenesulfonimidamide 4c

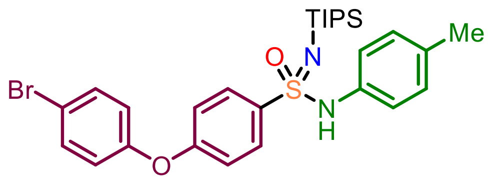

4-(4-Bromophenoxy)-*N*-(triisopropylsilyl)benzenesulfinamide **3d** (46.7 mg, 0.10 mmol, 1.0 equiv.) and trichloroisocyanuric acid (11.6 mg, 0.05 mmol, 0.5 equiv.) was added to a 5 mL vial. Anhydrous, degassed MeCN (0.1 M, 1.0 mL) was added at rt, and the reaction was stirred for 20 mins. NEt_3 (21 µL, 0.15 mmol, 1.5 equiv.) and *p*-toluidine (16.1 mg, 0.15 mmol, 1.5 equiv.) were added and the reaction was stirred at rt for 1 h. After completion of the reaction (as judged by TLC), the resulting mixture was diluted with EtOAc (5 mL) and filtered through a pad of silica (1.5 to 2 cm), washing with EtOAc (40 mL). The resulting crude mixture was concentrated *in vacuo* and purified by silica gel flash chromatography (pentane: EtOAc 20:1 to 4:1) affording **4c** as a light-yellow oil (48.1 mg, 84%).

Rf = 0.50 (6% EtOAc in pentane); **^{1}H NMR** (400 MHz, $CDCl_3$) δ 7.77 – 7.68 (m, 2H), 7.51 – 7.43 (m, 2H), 6.99 (d, *J* = 8.2 Hz, 2H), 6.94 – 6.85 (m, 6H), 6.25 (s, 1H), 2.26 (s, 3H), 1.21 – 1.02 (m, 21H); **^{13}C NMR** (101 MHz, $CDCl_3$) δ 159.8, 155.0, 138.5, 136.1, 134.1, 133.1, 129.6, 129.2, 121.7, 117.5, 117.2, 20.9, 18.5, 13.2. (Note: Missing a carbon signal due to overlapping peaks at 121.7 ppm); **IR** (ν_{max}, cm^{-1}) 3221, 2942, 2864, 2359, 1727, 1578, 1481, 1344, 1293, 1243, 1148, 1011, 883, 830, 766; **HRMS** (ESI+, *m/z*) calcd. for $[C_{28}H_{38}{}^{79}BrN_2O_2SSi]^+$ $[M+H]^+$ 573.1601; found: 573.1610; $[C_{28}H_{38}{}^{81}BrN_2O_2SSi]^+$ $[M+H]^+$ 575.1581; found: 575.1553;

4-(4-Bromophenoxy)-*N*-(triisopropylsilyl)benzenesulfonimidoyl fluoride 4d

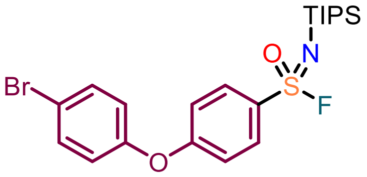

4-(4-Bromophenoxy)-*N*-(triisopropylsilyl)benzenesulfinamide **3d** (46.7 mg, 0.10 mmol, 1.0 equiv.) and NaH (6.0 mg, 0.15 mmol, 1.5 equiv., 60% in mineral oil) were added to an oven-dried 5 mL microwave vial. The vial was sealed and subjected to three evacuation/N_2 refill cycles before, anhydrous, degassed THF (0.1 M, 1 mL) was added at 0 ˚C. The reaction was stirred for 5 mins, then warmed to rt and stirred for another 25 mins. NFSI (47.3 mg, 0.15 mmol, 1.5 equiv.) was added and the reaction was stirred at rt for 3 h. After reaction completion (as judged by TLC), the resulting mixture was diluted with EtOAc (5 mL) and filtered through a pad of silica (1.5 to 2 cm), washing with EtOAc (40 mL). The resulting crude mixture was concentrated *in vacuo* and purified by silica gel flash chromatography (pentane: EtOAc 15:1 to 9:1) affording **4d** as a colourless oil (33.9 mg, 70%).

Rf = 0.41 (6% EtOAc in pentane); **^{1}H NMR** (400 MHz, $CDCl_3$) δ 8.02 – 7.94 (m, 2H), 7.58 – 7.48 (m, 2H), 7.08 – 7.02 (m, 2H), 7.01 – 6.91 (m, 2H), 1.22 – 1.15 (m, 3H), 1.11 (d, *J* = 4.9 Hz, 18H); **^{13}C NMR** (101 MHz, $CDCl_3$) δ 161.9, 154.4, 133.4, 132.8 (d, *J* = 33.8 Hz), 130.3, 122.2, 118.0, 117.7, 18.1, 12.7; **^{19}F NMR** (377 MHz, $CDCl_3$) δ 93.9 (s); **IR** (ν_{max}, cm^{-1}) 2944, 2867, 1727, 1578, 1482, 1410, 1248, 1217, 1167, 1070, 1011, 883, 833, 731; **HRMS** (ESI$^+$, *m/z*) calcd. for $[C_{21}H_{30}{}^{79}BrFNO_2SSi]^+$ $[M+H]^+$ 486.0928; found: 486.0951; $[C_{21}H_{30}{}^{81}BrFNO_2SSi]^+$ $[M+H]^+$ 488.0908; found: 488.0912.

4-([1,1’-Biphenyl]-4-yloxy)-*N*-(triisopropylsilyl)benzenesulfonamide 4e

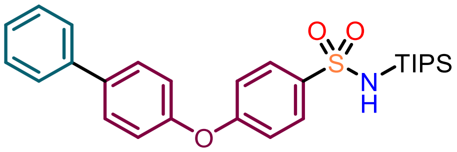

An oven-dried 5 mL microwave vial containing 4-(4-bromophenoxy)-*N*-(triisopropylsilyl)benzenesulfonamide **4b** (48.3 mg, 0.1 mmol, 1.0 equiv.), K_2CO_3 (27.6 mg, 0.20 mmol, 2.0 equiv.) and $PhB(OH)_2$ (18.3 mg, 0.15 mmol, 1.5 equiv.) was sealed and subjected to three evacuation/N_2 refill cycles before anhydrous, degassed toluene (0.45 mL) and H_2O (45 μL) were added. $Pd(PPh_3)_4$ (5.78 mg, 0.005 mmol, 5 mol%) was subsequently added in one portion under N_2 and the vial was sealed and N_2 bubbled through the reaction mixture for 1 min. The reaction was then

stirred for 19 h at 100 °C. The reaction mixture was cooled to rt and water (5 mL) was added. The aqueous phase was extracted with EtOAc (3 x 5 mL). The combined organic layer was dried over anhydrous $MgSO_4$, filtered and concentrated *in vacuo*. The crude mixture was purified by silica gel flash column chromatography (pentane: EtOAc 1:0 to 6:1) affording **4e** as an off-white solid (34.0 mg, 71%).

Rf = 0.44 (15% EtOAc in pentane); **^{1}H NMR** (400 MHz, $CDCl_3$) δ 7.92 – 7.84 (m, 2H), 7.63 – 7.57 (m, 4H), 7.50 – 7.41 (m, 2H), 7.40 – 7.31 (m, 1H), 7.15 – 7.05 (m, 4H), 4.27 (s, 1H), 1.28 (ddt, *J* = 14.2, 9.2, 6.9 Hz, 3H), 1.07 (d, *J* = 7.4 Hz, 18H); **^{13}C NMR** (101 MHz, $CDCl_3$) δ 160.9, 155.2, 140.4, 138.0, 137.9, 129.0, 128.9, 128.5, 127.5, 127.1, 120.4, 117.9, 18.1, 12.0; **IR** (ν_{max}, cm^{-1}) 3247, 2947, 2868, 2359, 1727, 1589, 1515, 1486, 1347, 1287, 1245, 1152, 1098, 1008, 934, 882, 764; **HRMS** (ESI^+, *m/z*) calcd. for $[C_{27}H_{36}NO_3SSi]^+$ $[M+H]^+$ 482.2180; found: 482.2164; **MP**: 120 – 121 °C (CH_2Cl_2).

5. Radical trapping experiment

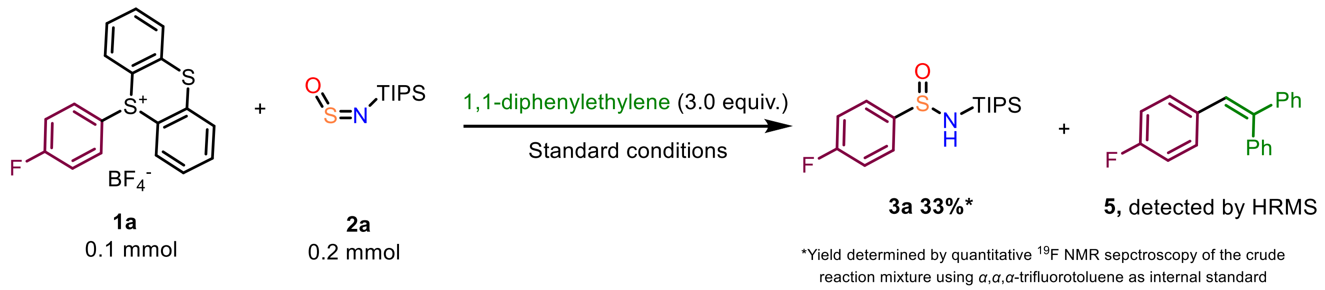

Figure S2 – Radical trapping experiment performed on **1a**

Tetrafluoroborate thianthrenium salt **1a** (39.8 mg, 0.1 mmol, 1.0 equiv.) and 1,1-diphenylethylene (53 µL, 0.3 mmol, 3.0 equiv.) were sealed and subjected to three evacuation/N_2 refill cycles before anhydrous, degassed MeCN (0.1 M, 1 mL) was added. After stirring for 1 minute, ensuring all solids had dissolved, NEt_3 (70 µL, 0.5 mmol, 5.0 equiv.) and TIPS-NSO **2a** (45 µL, 0.2 mmol, 2.0 equiv.) were then added to the reaction mixture. The reaction was then stirred for 24 h under 427 nm irradiation at ambient temperature (31°C, photoreactor fans on). *The formation of aryl sulfinamide product **3a** was determined by quantitative ^{19}F NMR spectroscopy of the crude reaction mixture using α,α,α-trifluorotoleuene as the internal standard.

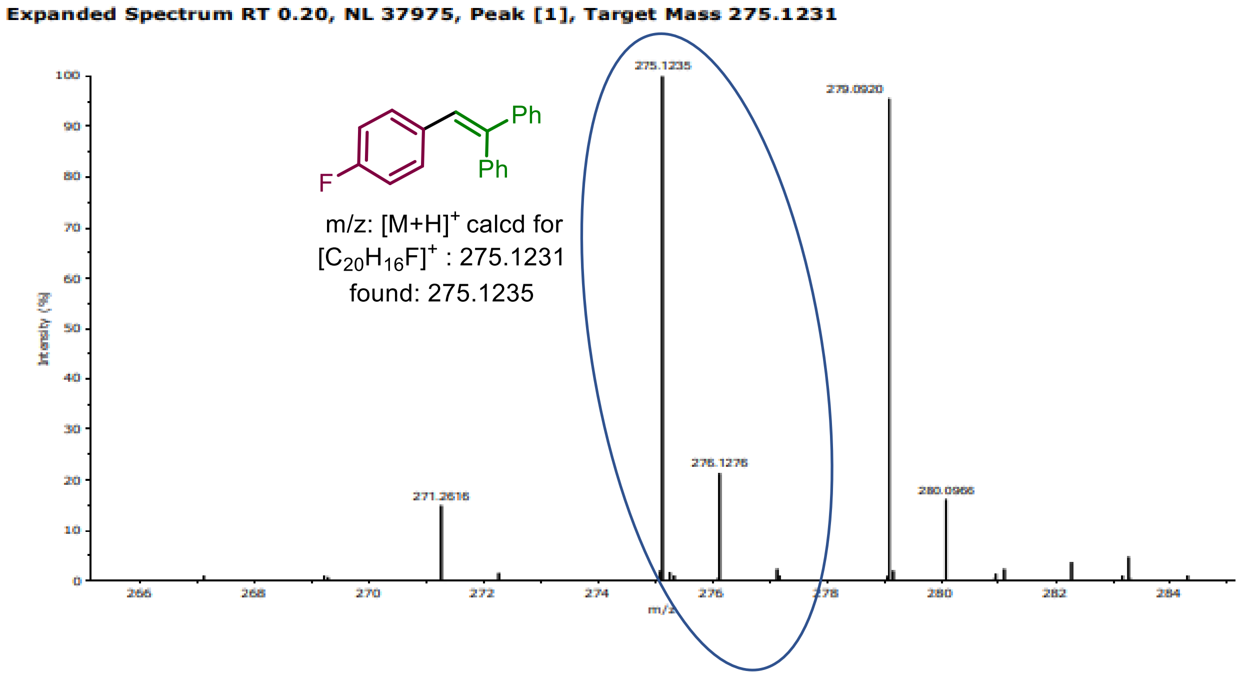

Figure S3 – HRMS analysis of compound **5**

Under these radical trapping conditions formation of sulfinamide **3a** was markedly suppressed to 33% along with detection of radical trapping intermediate **5** by HRMS (**Figure S3**). This data strongly indicates that the reaction involves a free radical pathway and reaffirms that sulfinylamines are effective radical traps for aryl radicals as well as well as the previously studied alkyl radicals.[19]

6. UV-Vis absorption spectrum

The UV-Vis absorption spectra of thianthrenium salt **1a** (0.33 mM), NEt_3 (1.67 mM) and combinations thereof in MeCN were recorded in 1 cm path quartz cuvettes using an Agilent Cary 60 UV-Vis spectrophotometer.

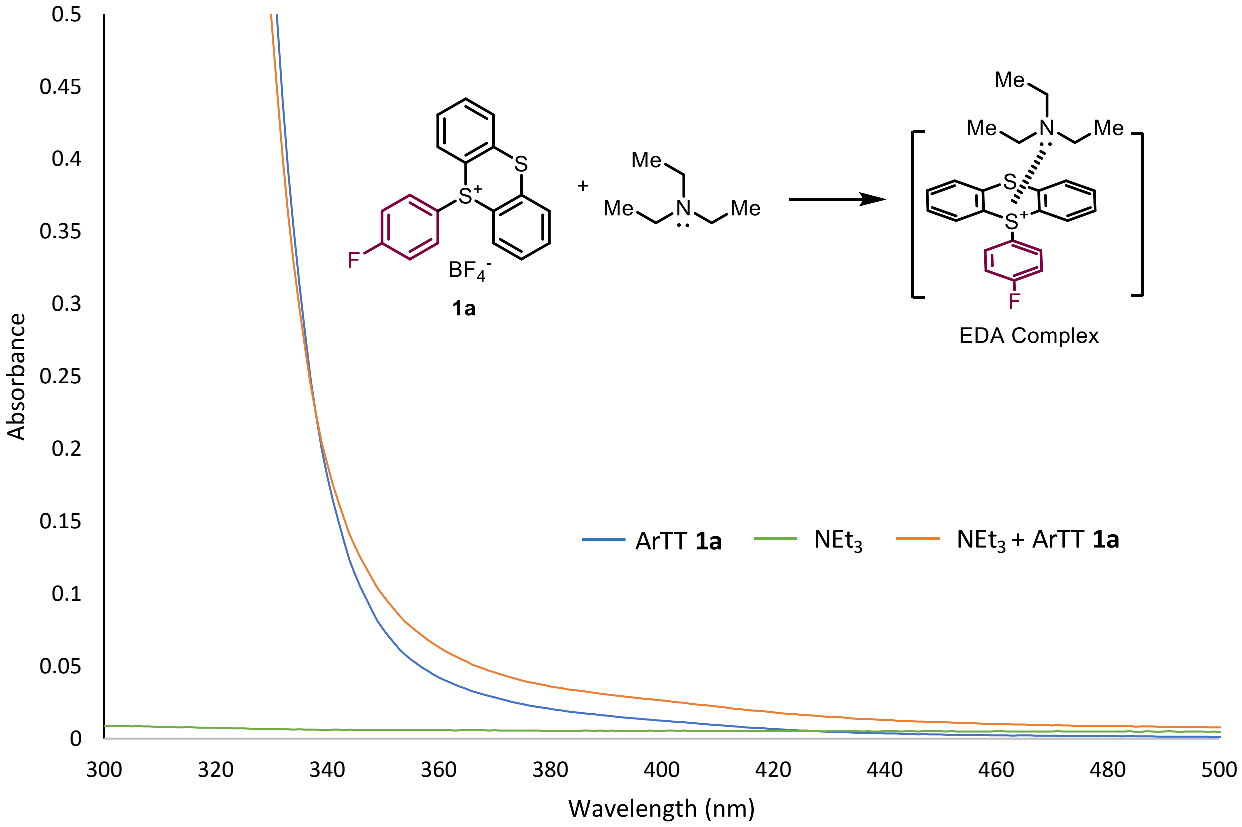

Figure S4 – UV-Vis absorption spectra of various combinations of **1a** and NEt_3 in MeCN

Figure S4 displays that upon mixing a solution of thianthrenium salt **1a** in MeCN with NEt_3, a clear bathochromic shift to wavelengths above 400 nm is observed, strongly suggesting that NEt_3 acts as the electron donor in a photoactive EDA complex with thianthrenium salt **1a**, which is consistent with previous literature.[20]

7. References

(1) Berger, F.; Plutschack, M. B.; Riegger, J.; Yu, W.; Speicher, S.; Ho, M.; Frank, N.; Ritter, T. Site-selective and versatile aromatic C−H functionalization by thianthrenation. *Nature* **2019**, *567* (7747), 223–228. DOI: 10.1038/s41586-019-0982-0.

(2) Juliá, F.; Shao, Q.; Duan, M.; Plutschack, M. B.; Berger, F.; Mateos, J.; Lu, C.; Xue, X.-S.; Houk, K. N.; Ritter, T. High Site Selectivity in Electrophilic Aromatic Substitutions: Mechanism of C–H Thianthrenation. *Journal of the American Chemical Society* **2021**, *143* (39), 16041–16054. DOI: 10.1021/jacs.1c06281.

(3) Ahmadli, D.; Müller, S.; Xie, Y.; Smejkal, T.; Jaeckh, S.; Iosub, A. V.; Williams, S. R.; Ritter, T. Standardized Approach for Diversification of Complex Small Molecules via Aryl Thianthrenium Salts. *Journal of the American Chemical Society* **2025**, *147* (5), 4268–4283. DOI: 10.1021/jacs.4c14391.

(4) Engl, P. S.; Häring, A. P.; Berger, F.; Berger, G.; Pérez-Bitrián, A.; Ritter, T. C–N Cross-Couplings for Site-Selective Late-Stage Diversification via Aryl Sulfonium Salts. *Journal of the American Chemical Society* **2019**, *141* (34), 13346–13351. DOI: 10.1021/jacs.9b07323.

(5) Tan, X.; Xiong, W.; Zhu, B.; Liu, H.; Wu, W.; Jiang, H. Photoinduced Arene C−H Amination with Ammonia: A Practical and Regioselective Strategy for Primary Amines. *Advanced Synthesis & Catalysis* **2023**, *365* (13), 2165–2170. DOI: https://doi.org/10.1002/adsc.202300384.

(6) Li, L.; Müller, S.; Petzold, R.; Ritter, T. Late-Stage Diazoester Installation via Arylthianthrenium Salts. *Angewandte Chemie International Edition* **2025**, *64* (5), e202419931. DOI: https://doi.org/10.1002/anie.202419931.

(7) Liu, T.; Cai, Y.-P.; Song, Q.-H. Visible-Light-Mediated Borylation of Arenes via an Electron Donor–Acceptor Complex of Thianthrenium Salts. *The Journal of Organic Chemistry* **2025**, *90* (19), 6569–6576. DOI: 10.1021/acs.joc.5c00469.

(8) Tian, Z.-Y.; Lin, Z.-H.; Zhang, C.-P. Pd/Cu-Catalyzed C–H/C–H Cross Coupling of (Hetero)Arenes with Azoles through Arylsulfonium Intermediates. *Organic Letters* **2021**, *23* (11), 4400–4405. DOI: 10.1021/acs.orglett.1c01322.

(9) Chen, X.-Y.; Li, Y.-N.; Wu, Y.; Bai, J.; Guo, Y.; Wang, P. Cu-Mediated Thianthrenation and Phenoxathiination of Arylborons. *Journal of the American Chemical Society* **2023**, *145* (18), 10431–10440. DOI: 10.1021/jacs.3c03413.

(10) Cheng, K.; Webb, E. W.; Bowden, G. D.; Wright, J. S.; Shao, X.; Sanford, M. S.; Scott, P. J. H. Photo- and Cu-Mediated 11C Cyanation of (Hetero)Aryl Thianthrenium Salts. *Organic Letters* **2024**, *26* (16), 3419–3423. DOI: 10.1021/acs.orglett.4c00929.

(11) Ding, M.; Zhang, Z.-X.; Davies, T. Q.; Willis, M. C. A Silyl Sulfinylamine Reagent Enables the Modular Synthesis of Sulfonimidamides via Primary Sulfinamides. *Organic Letters* **2022**, *24* (8), 1711–1715. DOI: 10.1021/acs.orglett.2c00347.

(12) Davies, T. Q.; Hall, A.; Willis, M. C. One-Pot, Three-Component Sulfonimidamide Synthesis Exploiting the Sulfinylamine Reagent N-Sulfinyltritylamine, TrNSO. *Angewandte Chemie International Edition* **2017**, *56* (47), 14937–14941. DOI: https://doi.org/10.1002/anie.201708590.

(13) Zhang, Z.-X.; Davies, T. Q.; Willis, M. C. Modular Sulfondiimine Synthesis Using a Stable Sulfinylamine Reagent. *Journal of the American Chemical Society* **2019**, *141* (33), 13022–13027. DOI: 10.1021/jacs.9b06831.

(14) Andresini, M.; Marraffa, L.; Şerbetçi, D.; Natho, P.; Colella, M.; Degennaro, L.; Luisi, R. Synthesis of Aza-S(VI) Fluorides and Primary Sulfonimidamides from Sulfinylamines. *Advanced Synthesis & Catalysis* **2025**, *367* (2), e202400908. DOI: https://doi.org/10.1002/adsc.202400908.

(15) Lei, T.; Zhou, Y.; Cheng, C.-Y.; Cao, Y.; Peng, Y.; Bian, J.; Pei, J. Aceno[2,1,3]thiadiazoles for Field-Effect Transistors: Synthesis and Crystal Packing. *Organic Letters* **2011**, *13* (10), 2642–2645. DOI: 10.1021/ol200748c.

(16) Wei, M.-K.; Moseley, D. F.; Bär, R. M.; Sempere, Y.; Willis, M. C. Palladium-Catalyzed Addition of Aryl Halides to N-Sulfinylamines for the Synthesis of Sulfinamides. *Journal of the American Chemical Society* **2024**, *146* (29), 19690–19695. DOI: 10.1021/jacs.4c06726.

(17) Shi, Y.; Yuan, Y.; Li, J.; Yang, J.; Zhang, J. Catalytic Asymmetric Synthesis of Sulfinamides via Cu-Catalyzed Asymmetric Addition of Aryl Boroxines to Sulfinylamines. *Journal of the American Chemical Society* **2024**, *146* (26), 17580–17586. DOI: 10.1021/jacs.4c03473.

(18) Lo, P. K. T.; Oliver, G. A.; Willis, M. C. Sulfinamide Synthesis Using Organometallic Reagents, DABSO, and Amines. *The Journal of Organic Chemistry* **2020**, *85* (9), 5753–5760. DOI: 10.1021/acs.joc.0c00334.

(19) Andrews, J. A.; Kalepu, J.; Palmer, C. F.; Poole, D. L.; Christensen, K. E.; Willis, M. C. Photocatalytic Carboxylate to Sulfinamide Switching Delivers a Divergent Synthesis of Sulfonamides and Sulfonimidamides. *Journal of the American Chemical Society* **2023**, *145* (39), 21623–21629. DOI: 10.1021/jacs.3c07974.

(20) Zhang, G.; Luo, Z.; Mei, G.; Wang, H.; Ding, C. EDA Complex from BCP-Thianthrenium Salt: A Catalyst-Free Strategy To Access 1-Trifluoromethyl-3-quinoxaline Derivatives Bicyclo[1.1.1]pentanes. *European Journal of Organic Chemistry* **2024**, *27* (27), e202400386. DOI: https://doi.org/10.1002/ejoc.202400386.

8. NMR spectra

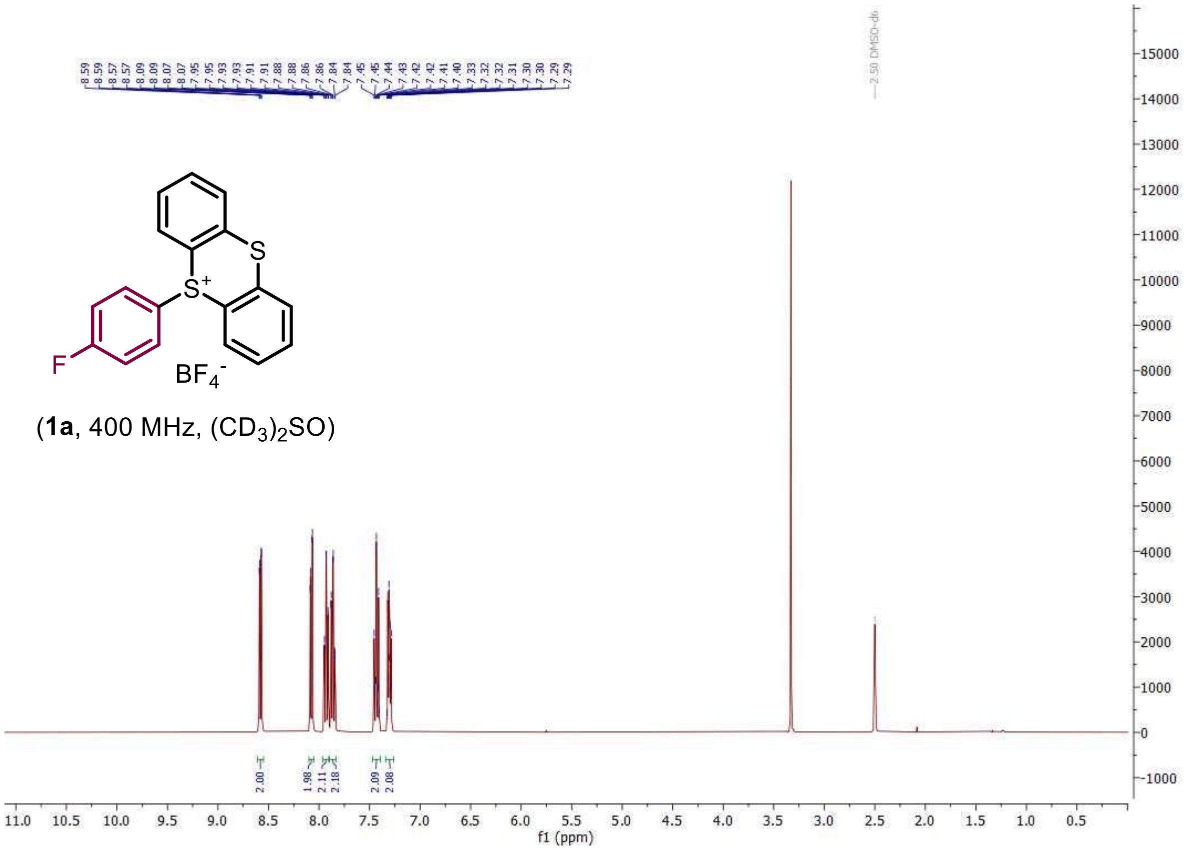

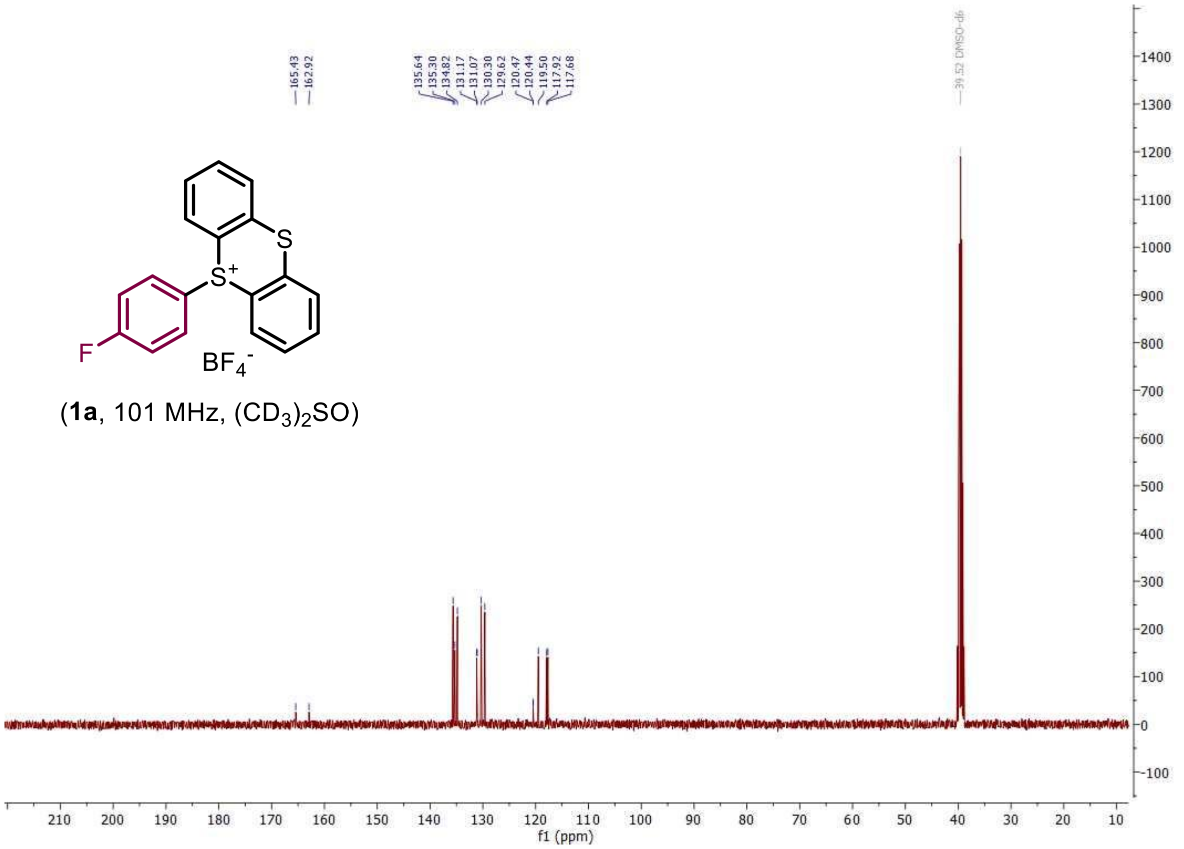

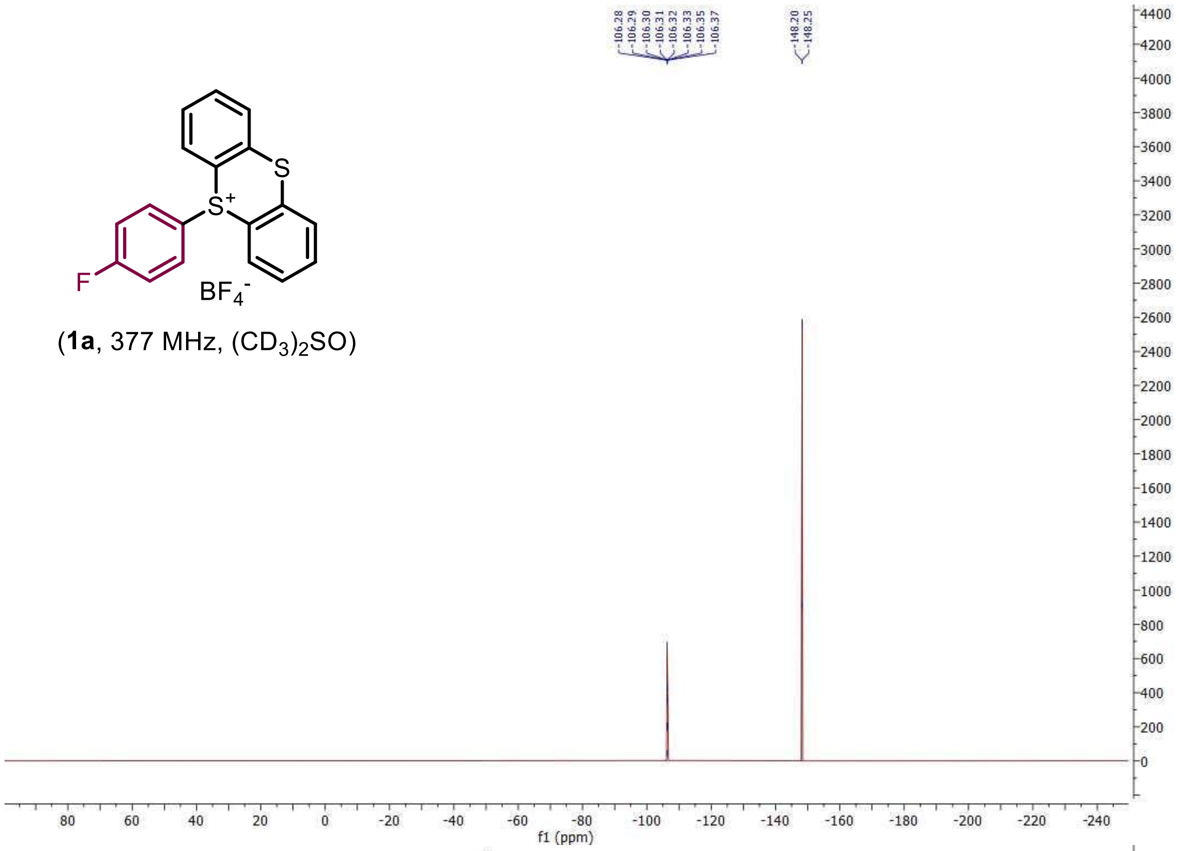
S
S+
F
BF4-
(**1a**, 377 MHz, $(CD_3)_2SO$)
f1 (ppm)

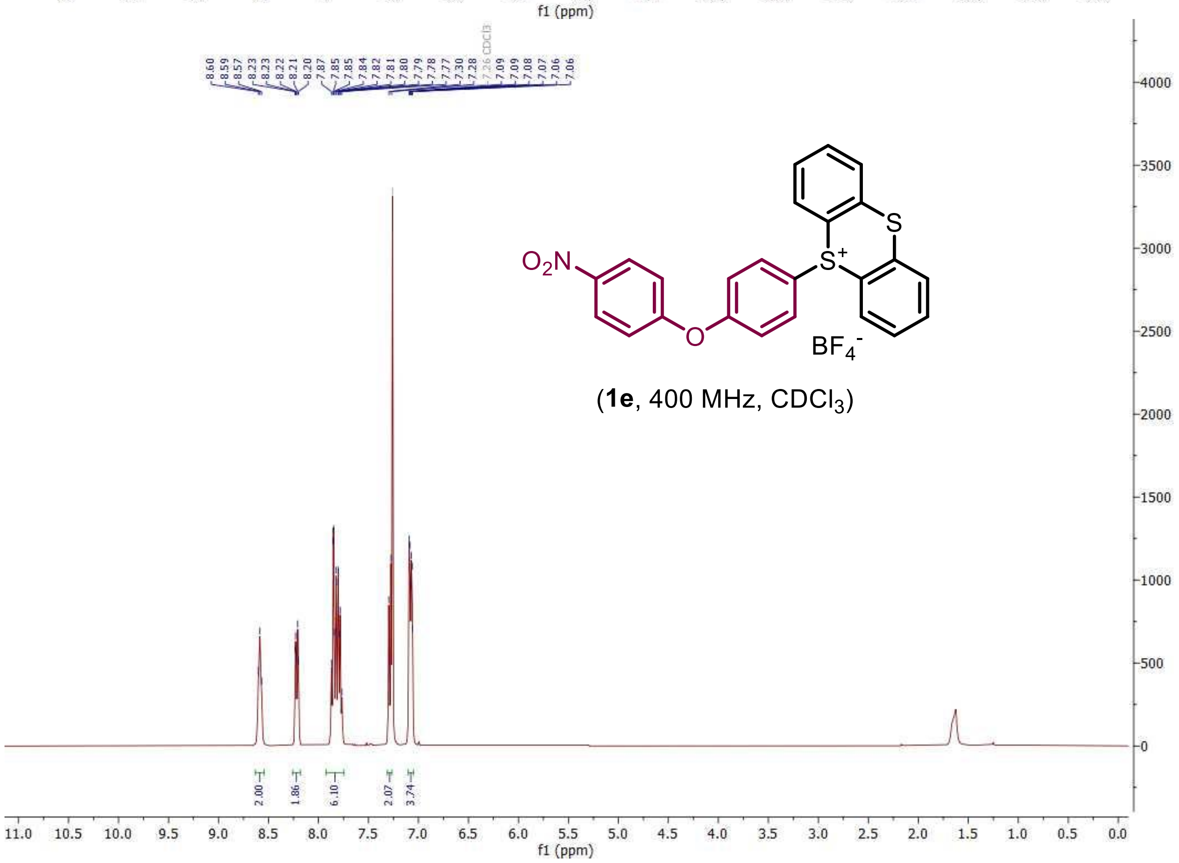
S
S+
O2N
O
BF4-
(**1e**, 400 MHz, $CDCl_3$)
f1 (ppm)

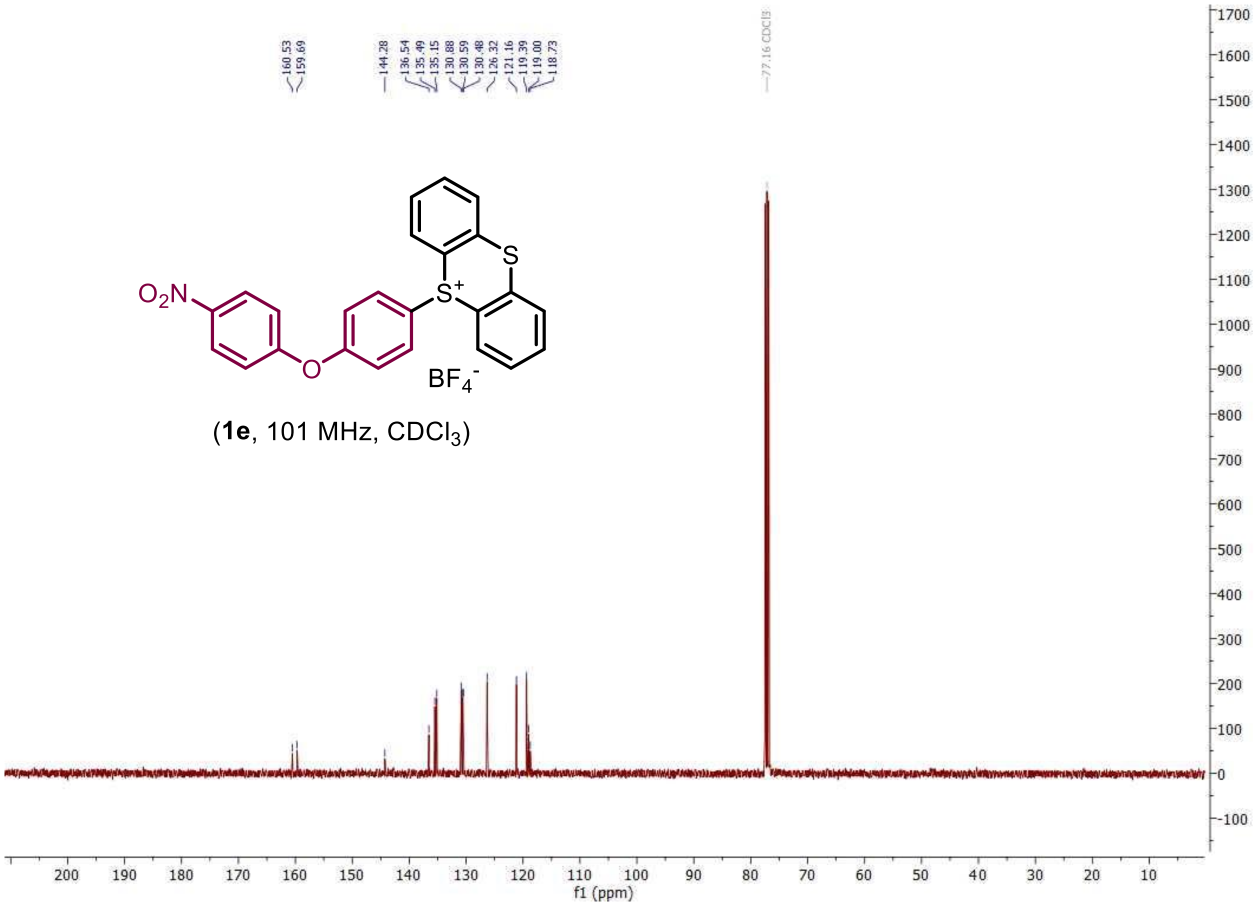

(**1e**, 101 MHz, $CDCl_3$)

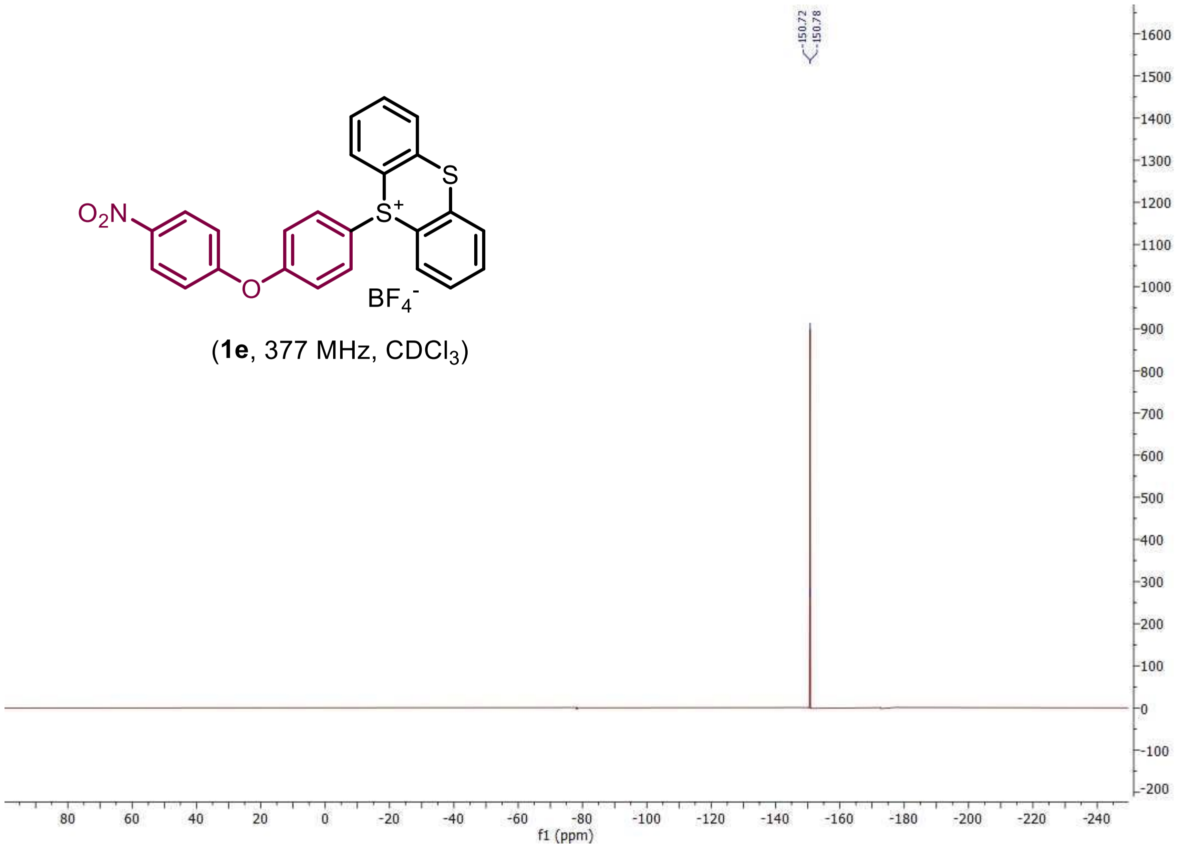

(**1e**, 377 MHz, $CDCl_3$)

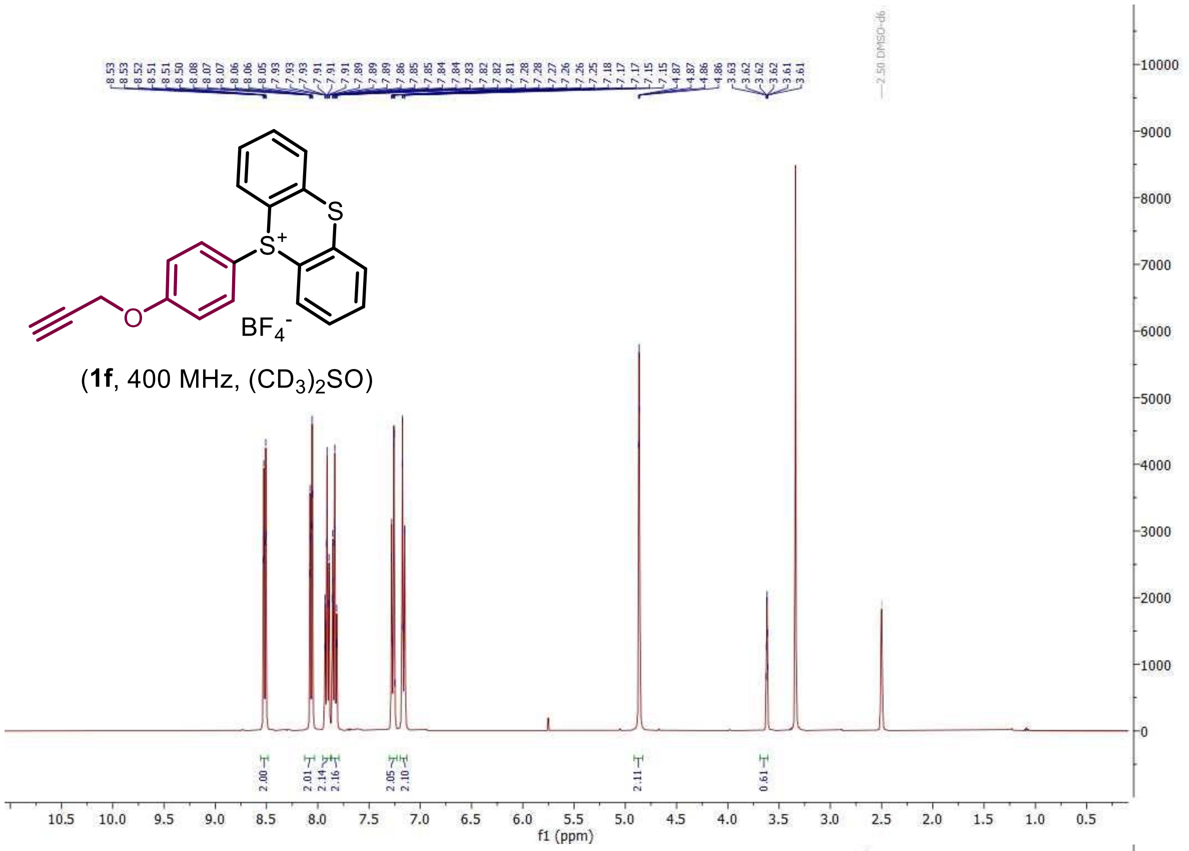
8.53 8.53 8.52 8.51 8.51 8.50 8.08 8.07 8.07 8.06 8.06 8.05 7.93 7.93 7.93 7.91 7.91 7.91 7.89 7.89 7.89 7.86 7.85 7.85 7.84 7.84 7.83 7.82 7.82 7.81 7.28 7.28 7.27 7.26 7.26 7.25 7.18 7.17 7.17 7.15 4.87 4.87 4.86 4.86 3.63 3.62 3.62 3.62 3.61 3.61
2.50 DMSO-d6
S
S+
O
BF4-
(1f, 400 MHz, (CD3)2SO)
2.00 2.01 2.14 2.16 2.05 2.10 2.11 0.61
f1 (ppm)

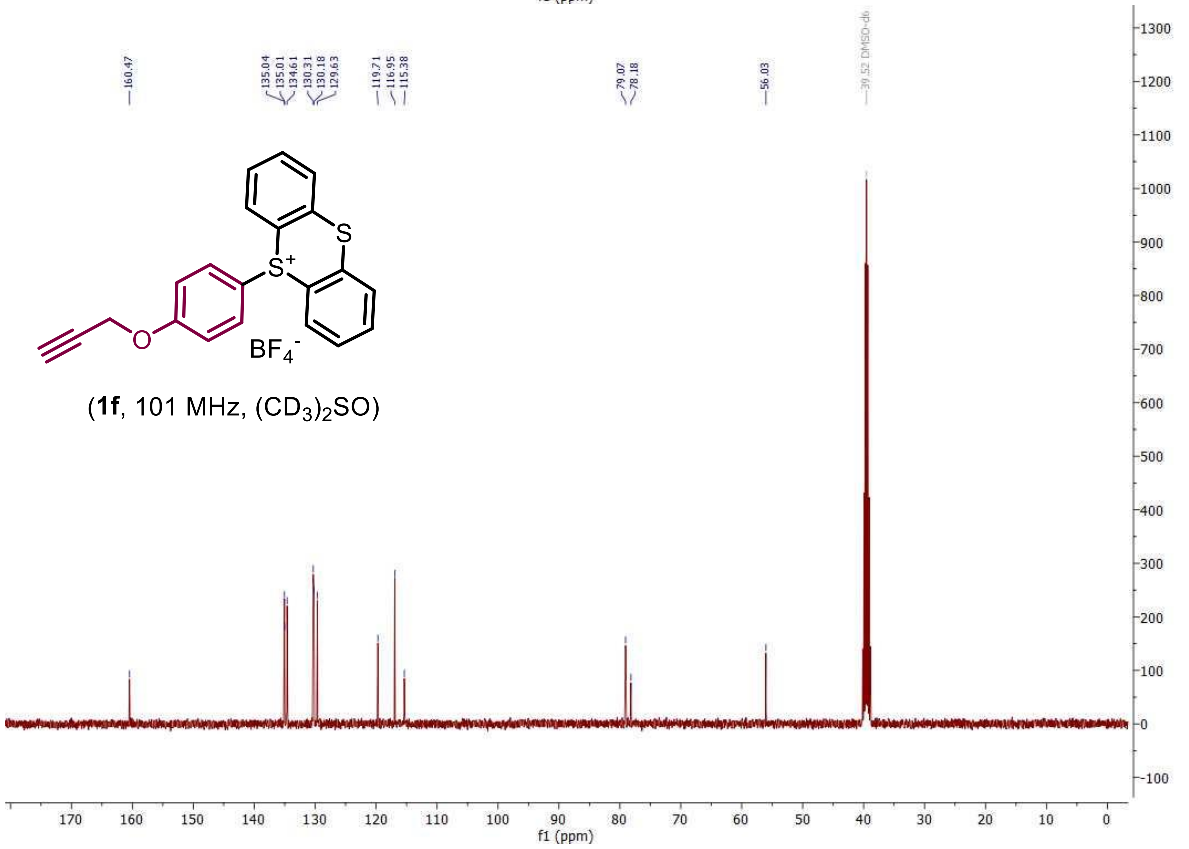
160.47
135.04 135.01 134.61 130.31 130.18 129.63
119.71 116.95 115.38
79.07 78.18
56.03
39.52 DMSO-d6
S
S+
O
BF4-
(1f, 101 MHz, (CD3)2SO)
f1 (ppm)

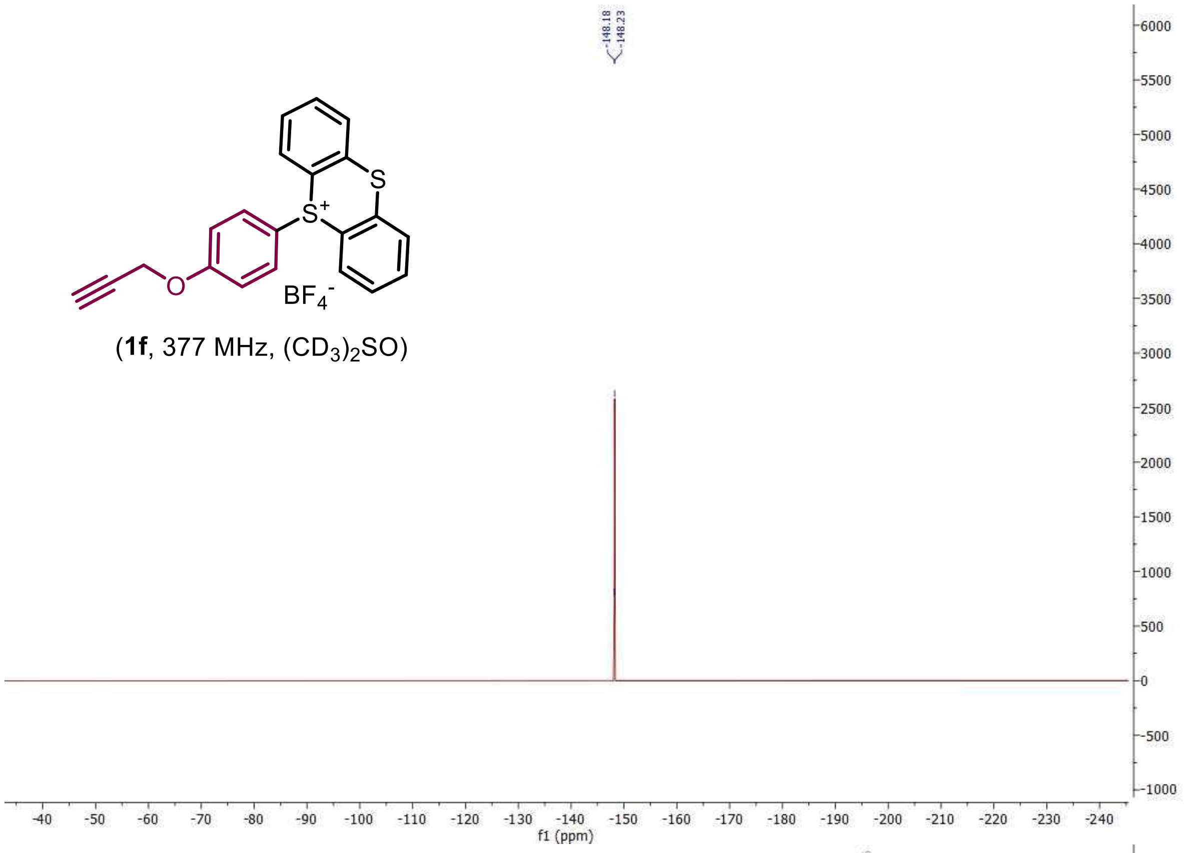

-148.18
-148.23
BF4-
(1f, 377 MHz, (CD3)2SO)
f1 (ppm)

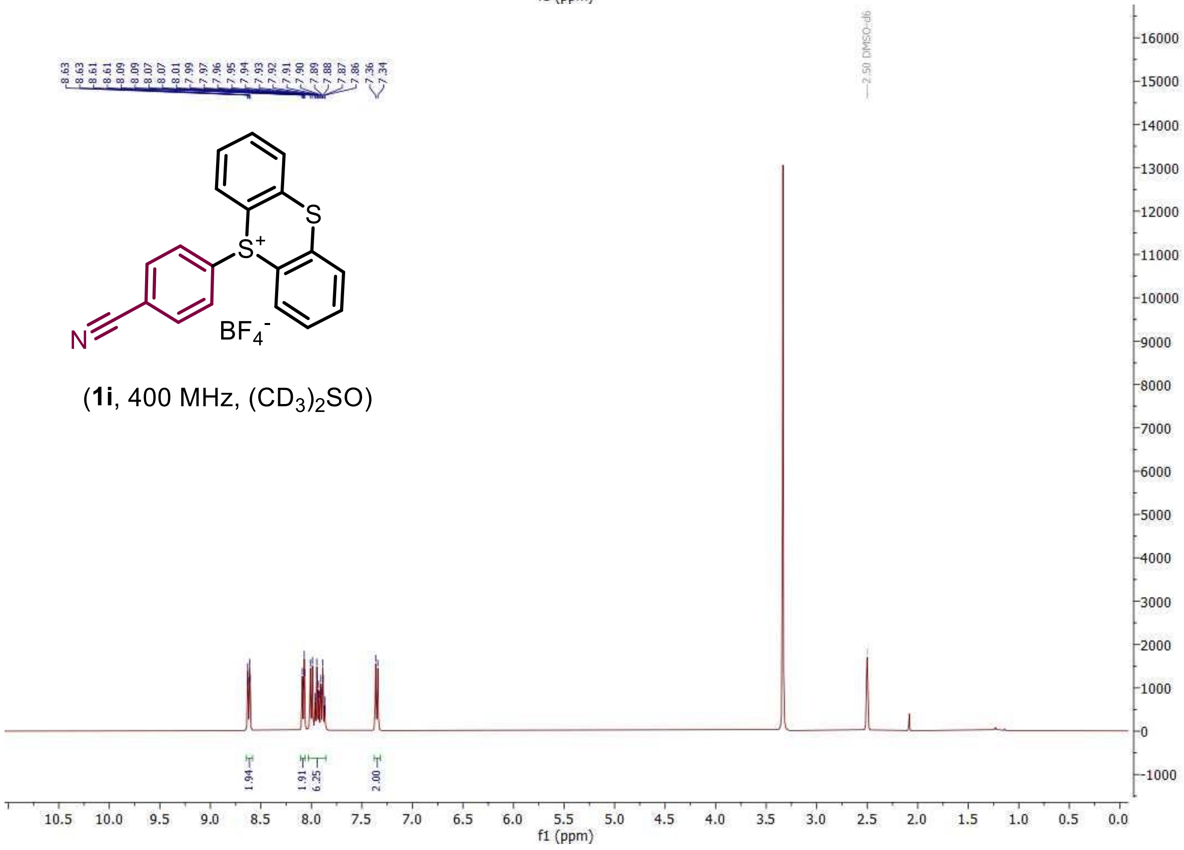

2.50 DMSO-d6
BF4-
(1i, 400 MHz, (CD3)2SO)
f1 (ppm)

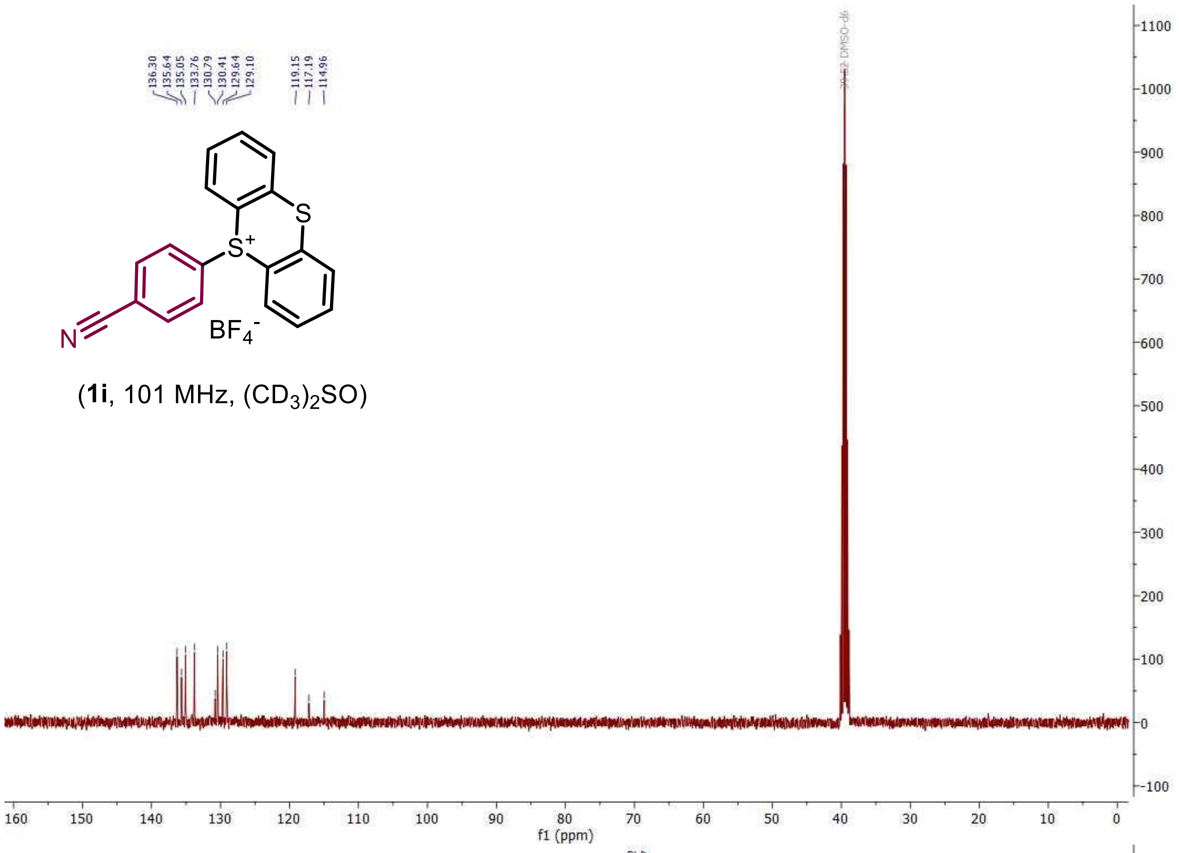

(**1i**, 101 MHz, $(CD_3)_2SO$)

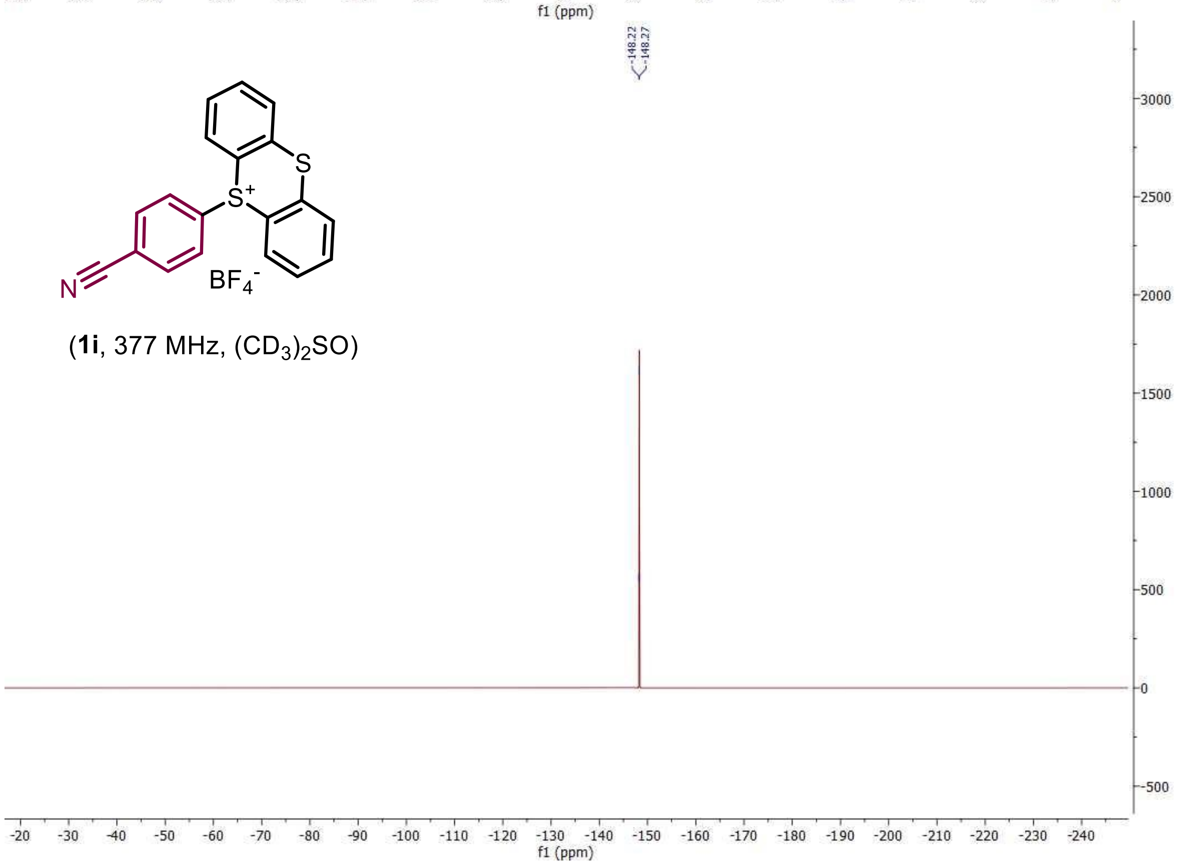

(**1i**, 377 MHz, $(CD_3)_2SO$)

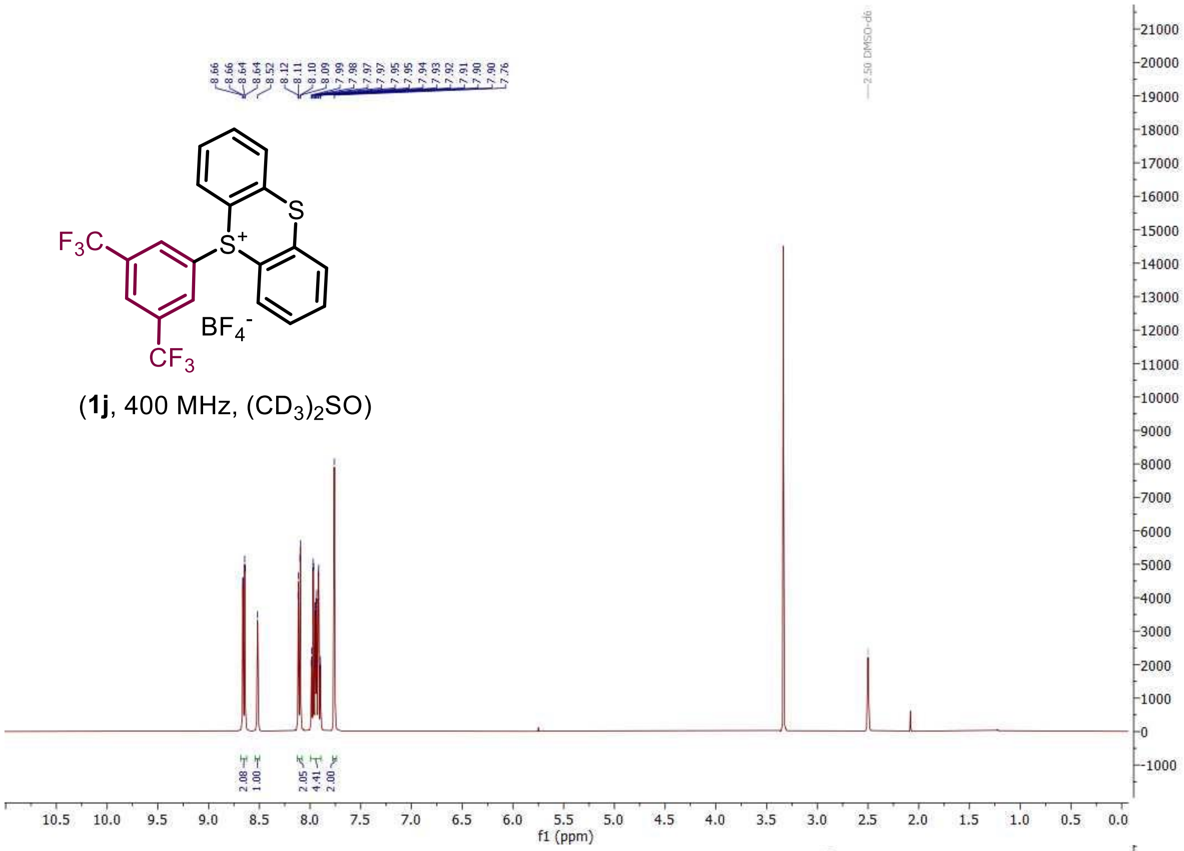

(**1j**, 400 MHz, $(CD_3)_2SO$)

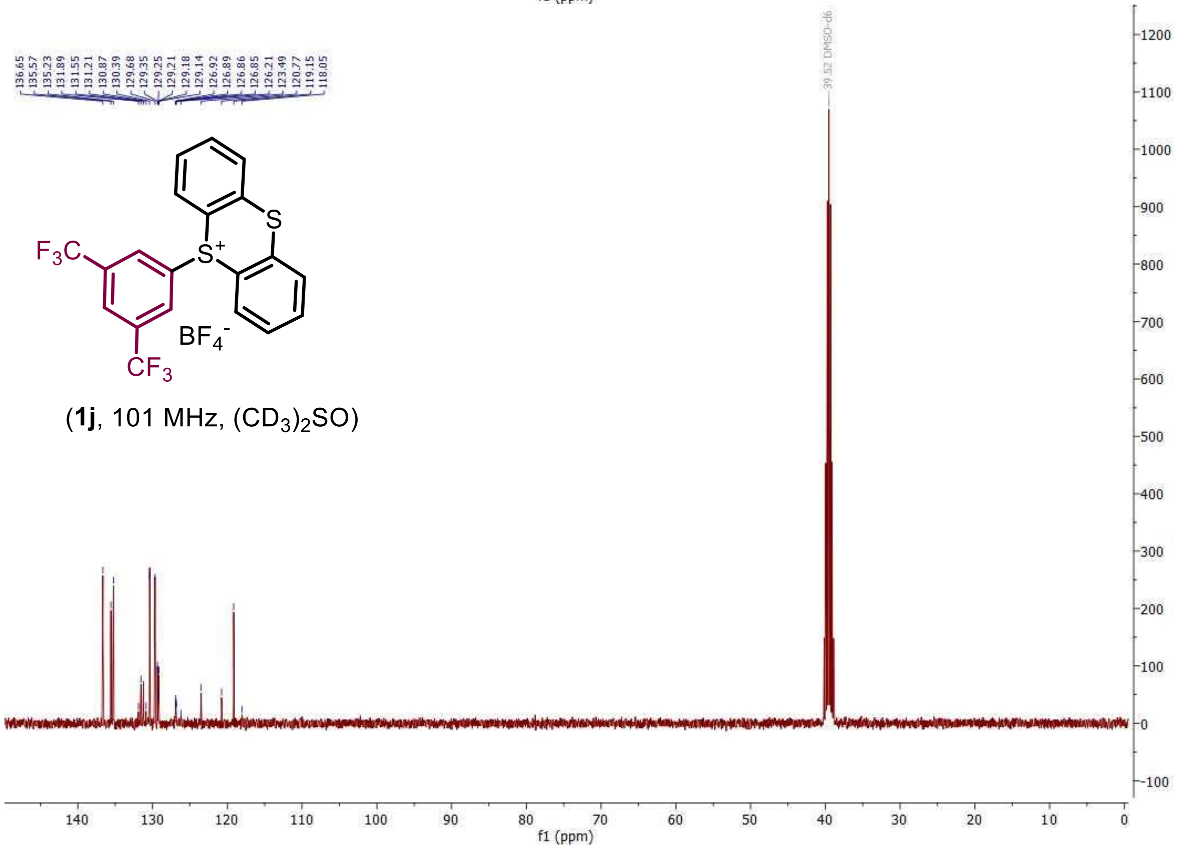

(**1j**, 101 MHz, $(CD_3)_2SO$)

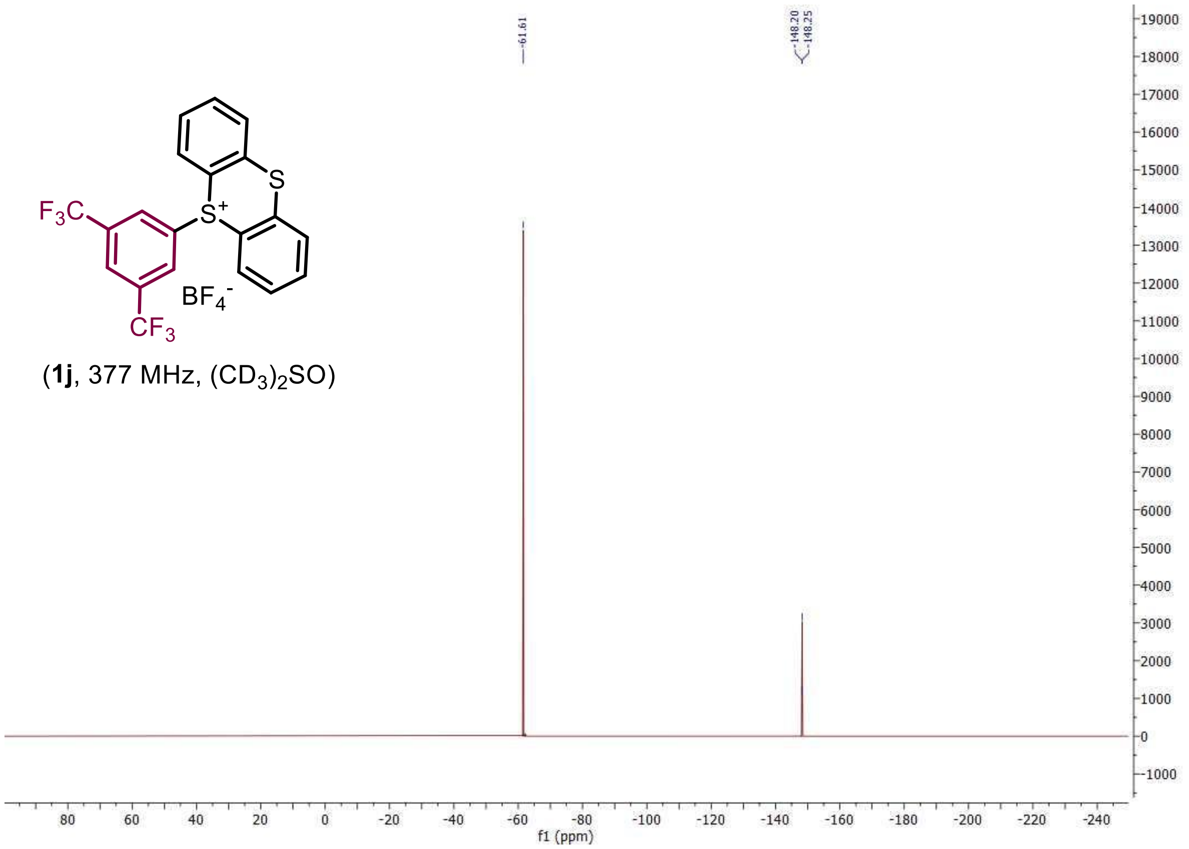

(**1j**, 377 MHz, $(CD_3)_2SO$)

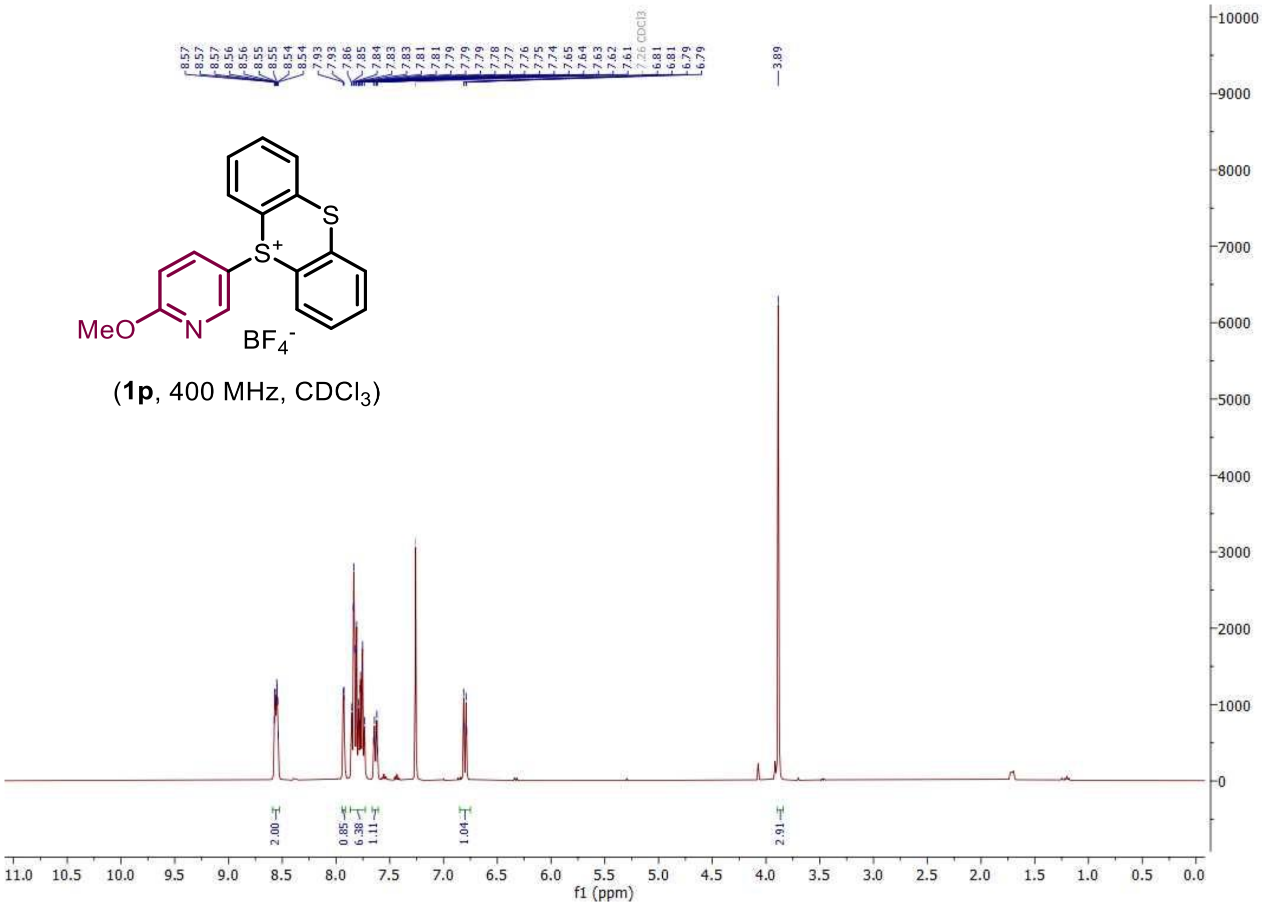

(**1p**, 400 MHz, $CDCl_3$)

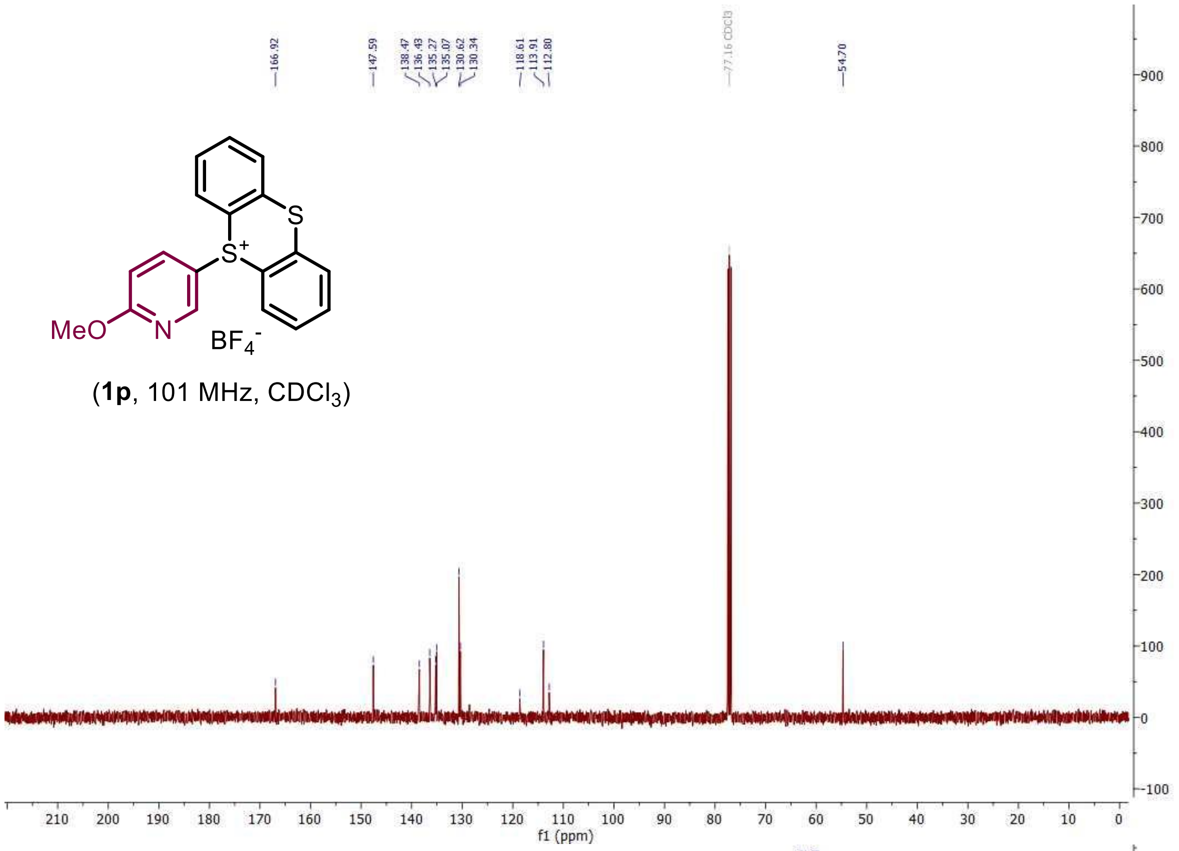

(**1p**, 101 MHz, $CDCl_3$)

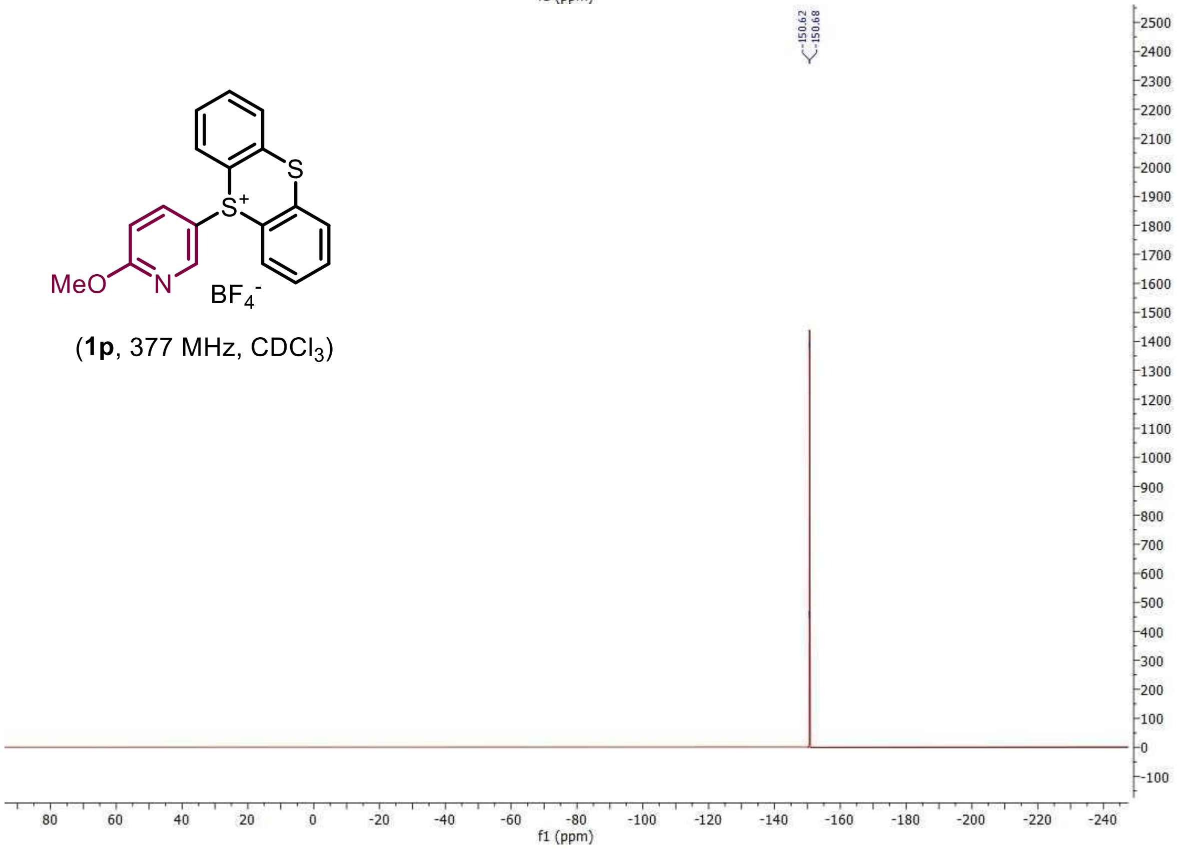

(**1p**, 377 MHz, $CDCl_3$)

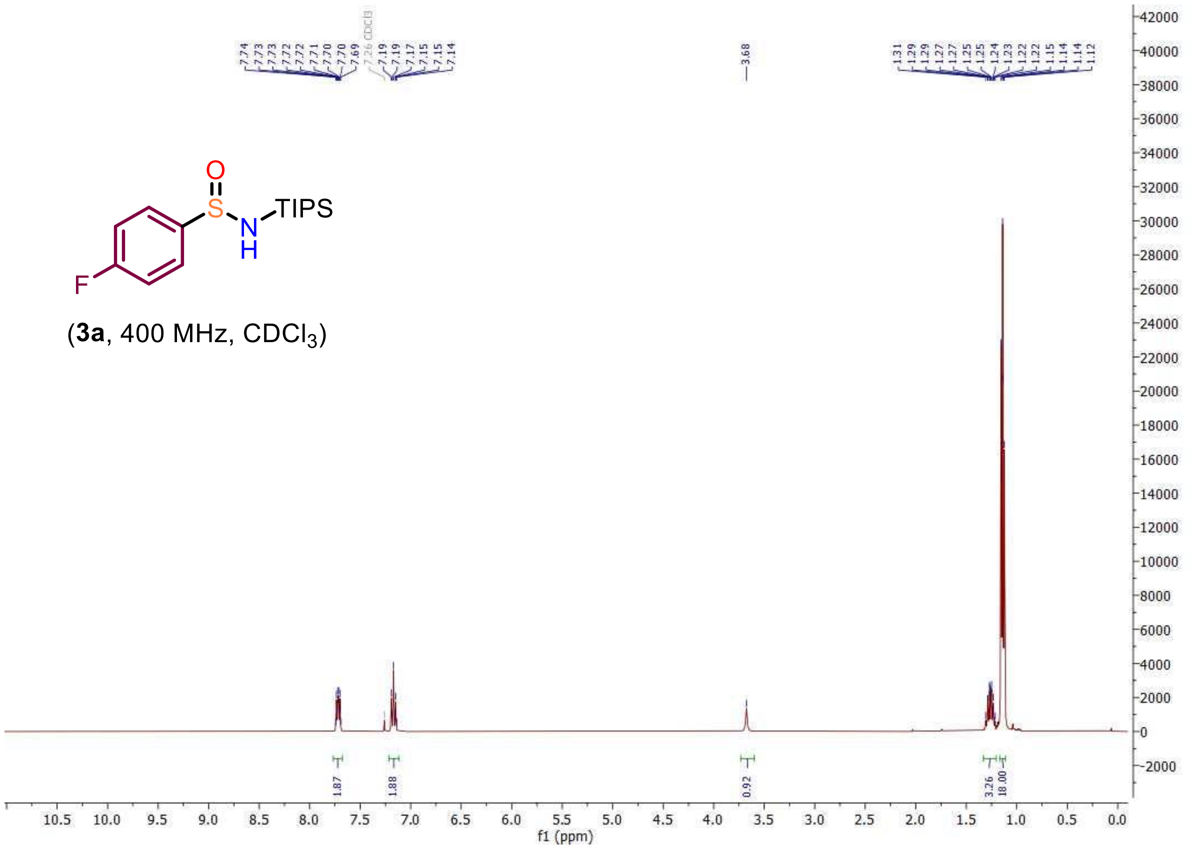
TIPS
(**3a**, 400 MHz, $CDCl_3$)
f1 (ppm)

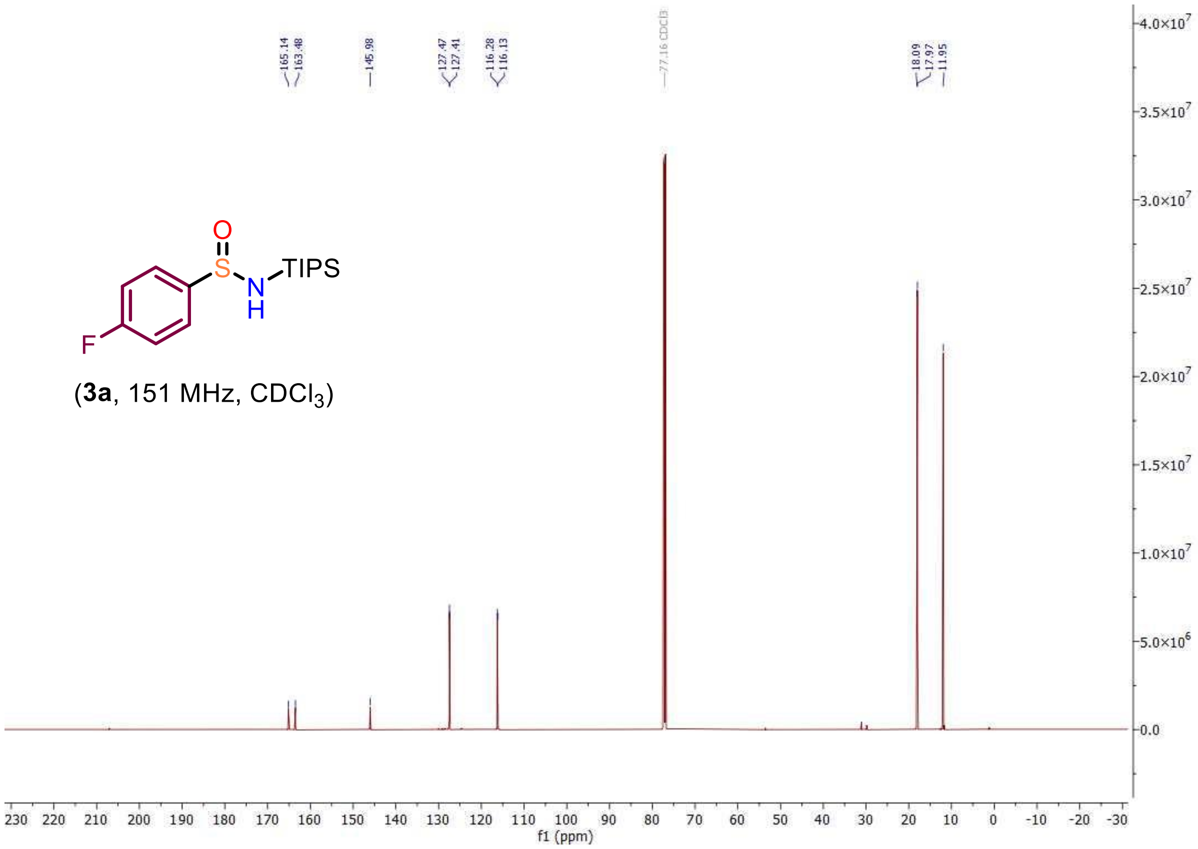
TIPS
(**3a**, 151 MHz, $CDCl_3$)
f1 (ppm)

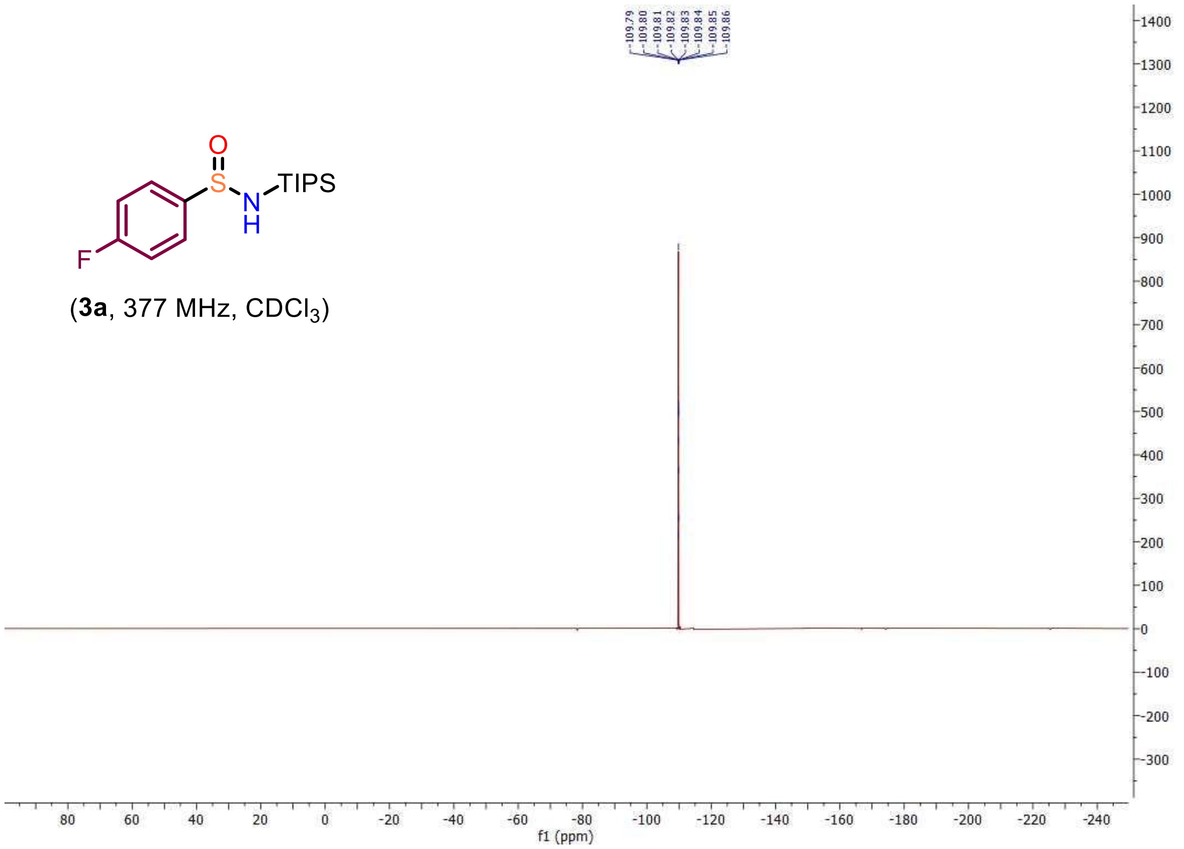

(**3a**, 377 MHz, $CDCl_3$)

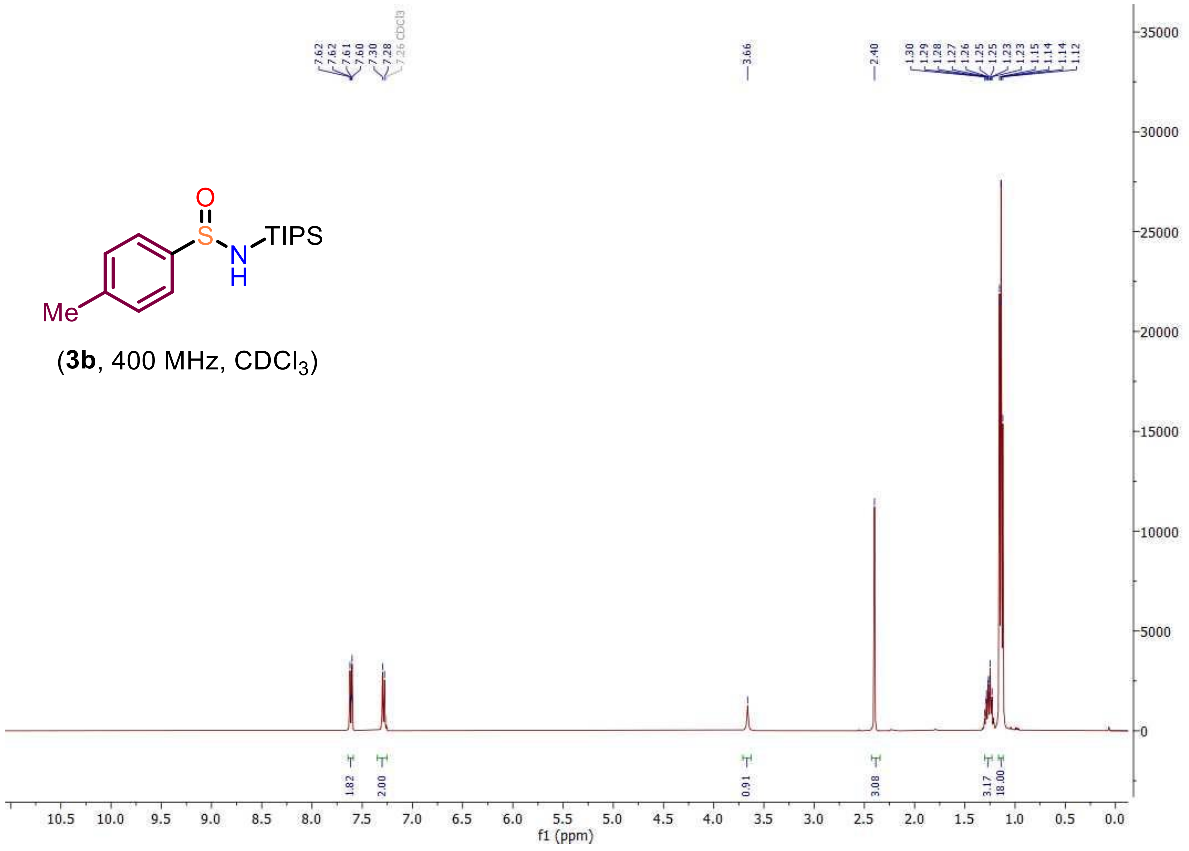

(**3b**, 400 MHz, $CDCl_3$)

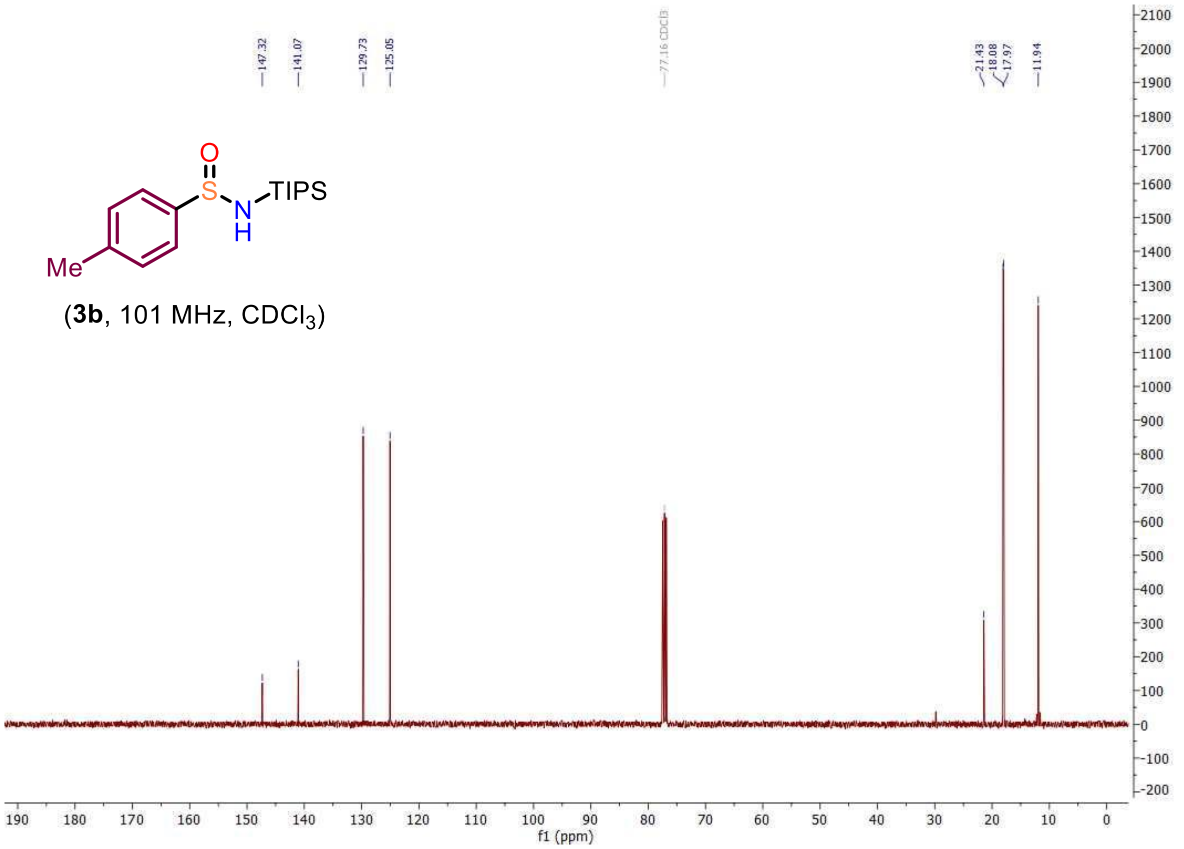

TIPS
N
H
Me
(3b, 101 MHz, CDCl3)
f1 (ppm)

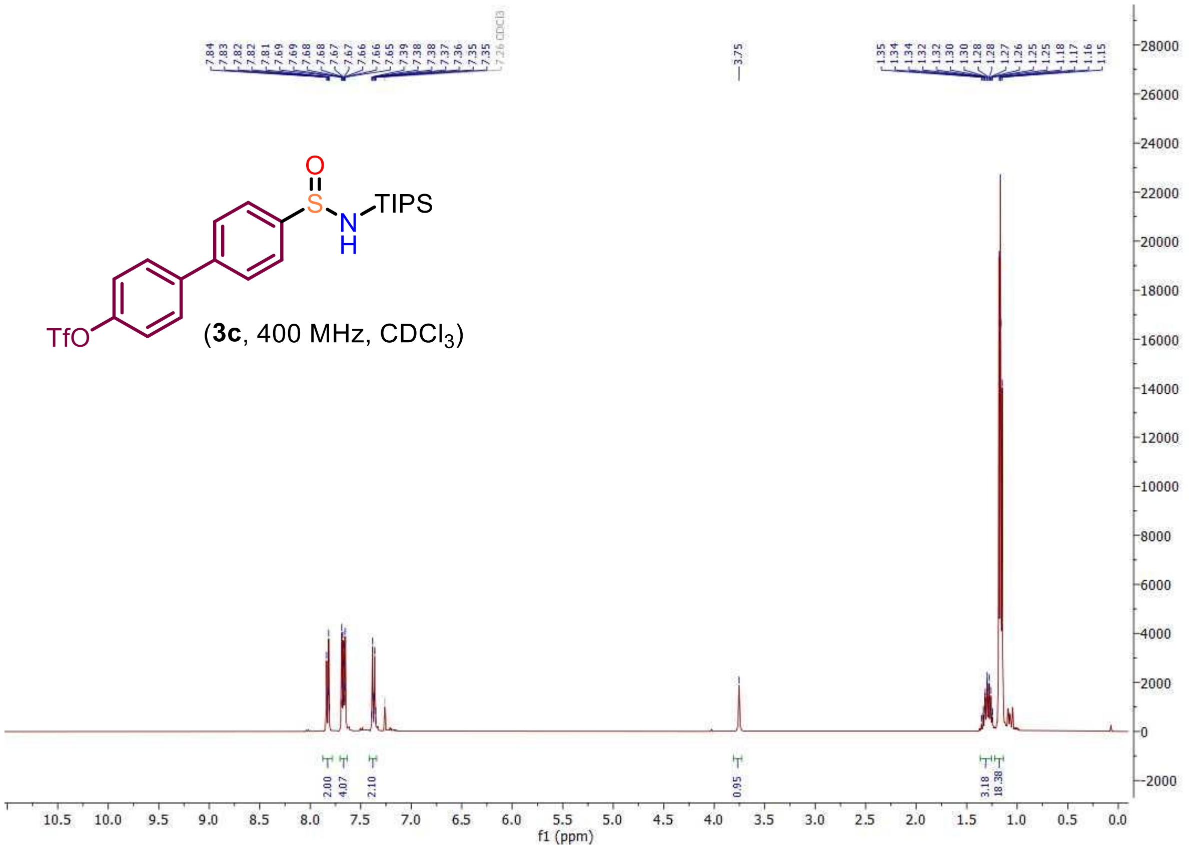

TIPS
N
H
TfO
(3c, 400 MHz, CDCl3)
f1 (ppm)

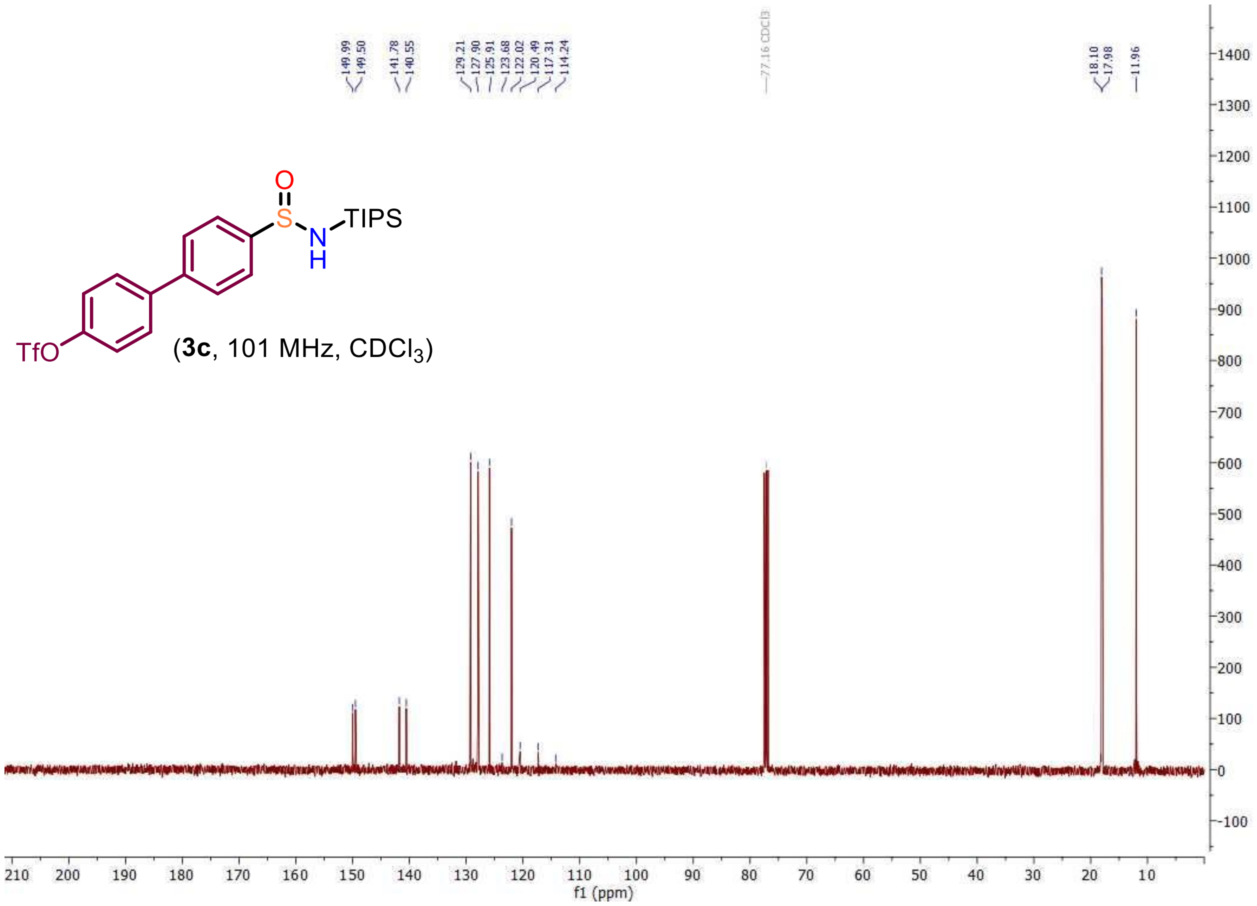
149.99
149.50
141.78
140.55
129.21
127.90
125.91
123.68
122.02
120.49
117.31
114.24
77.16 CDCl3
18.10
17.98
11.96
TIPS
TfO
(3c, 101 MHz, CDCl3)
f1 (ppm)

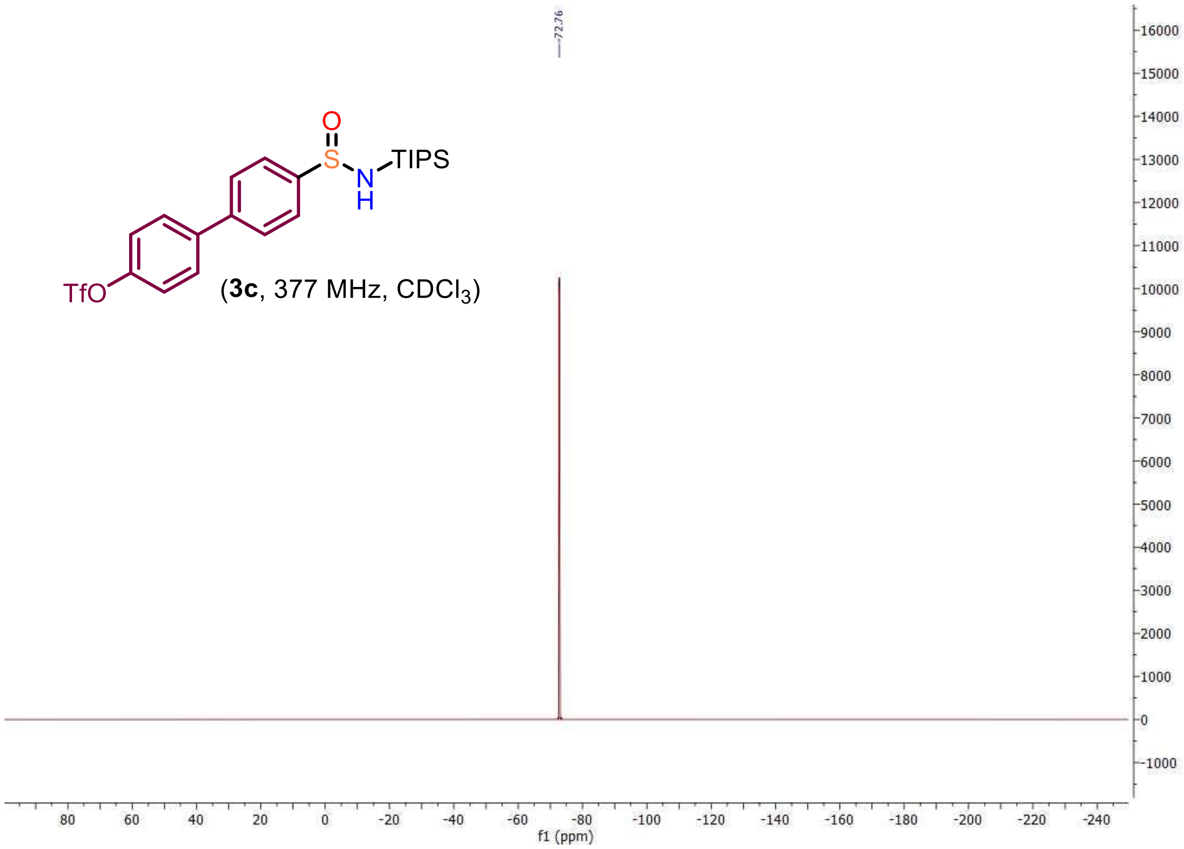
-72.76
TIPS
TfO
(3c, 377 MHz, CDCl3)
f1 (ppm)

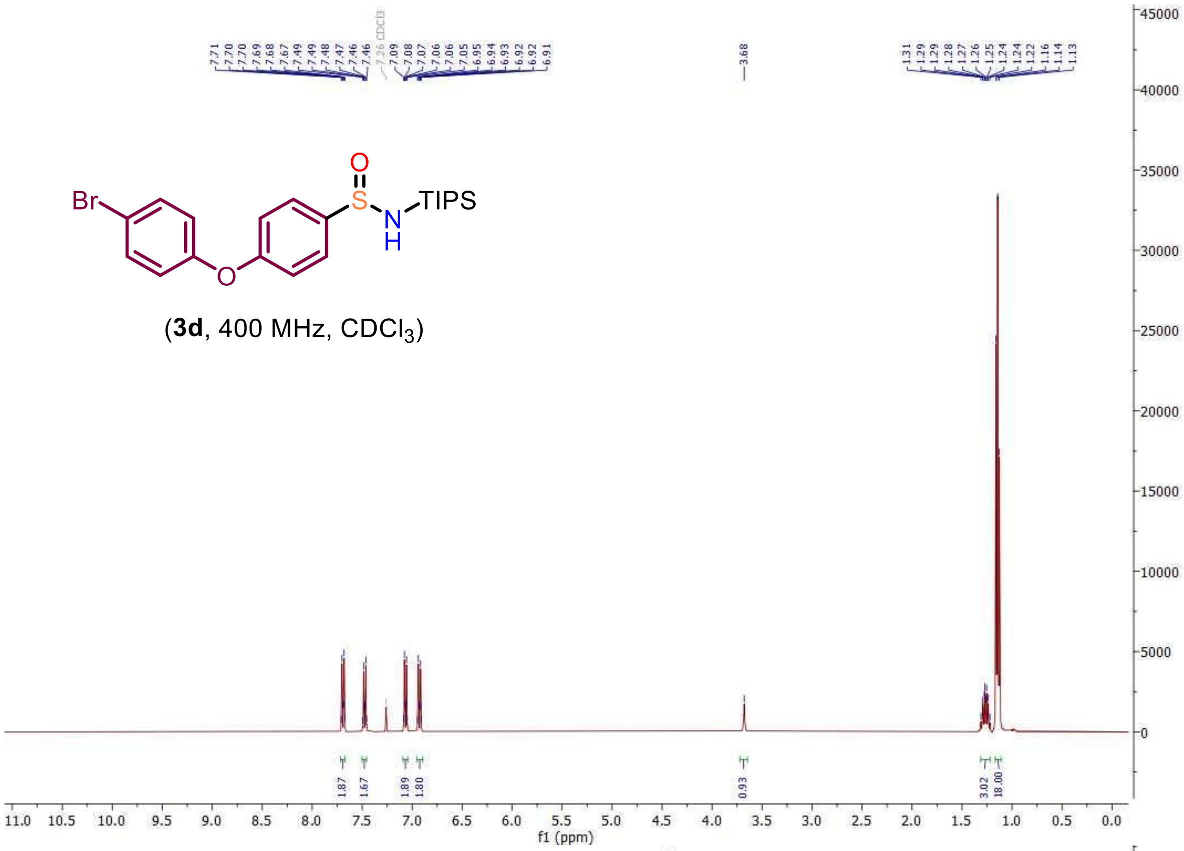
Br
O
S
N
H
TIPS
(3d, 400 MHz, CDCl3)
f1 (ppm)

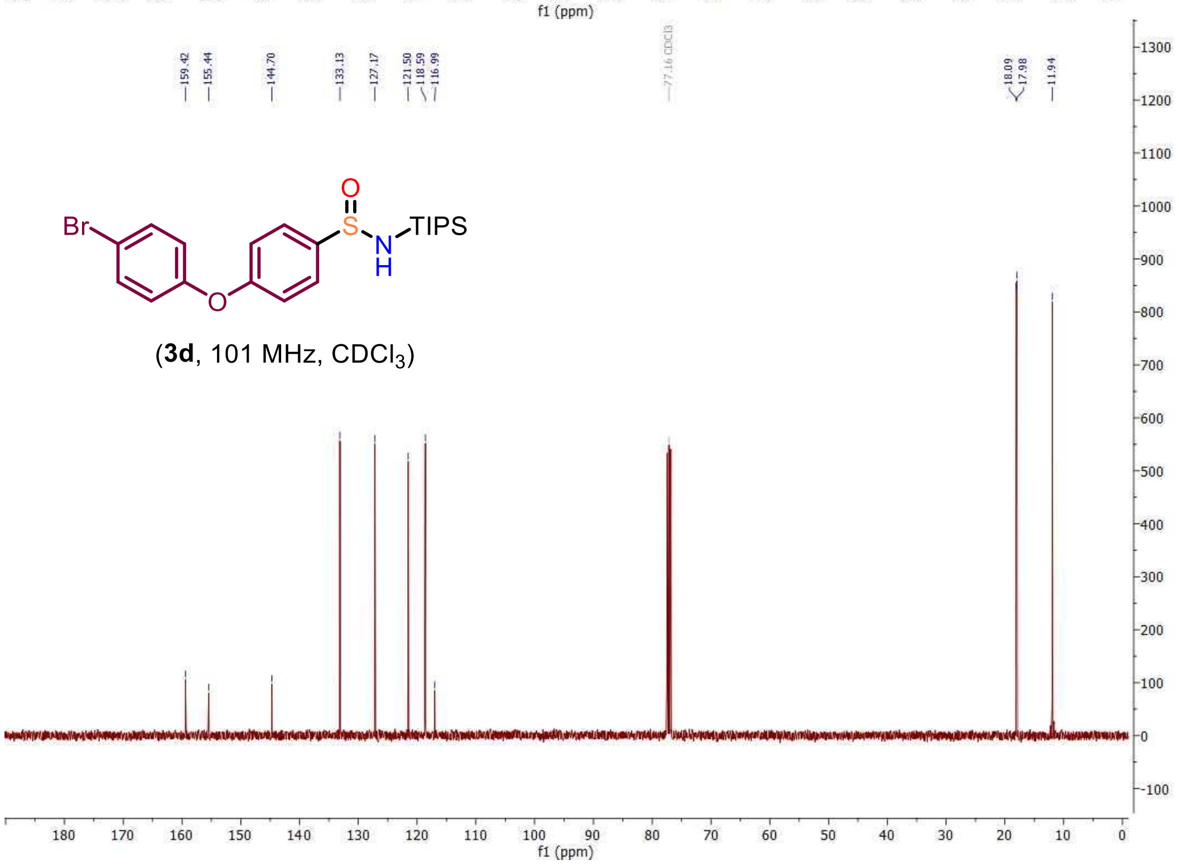
Br
O
S
N
H
TIPS
(3d, 101 MHz, CDCl3)
f1 (ppm)

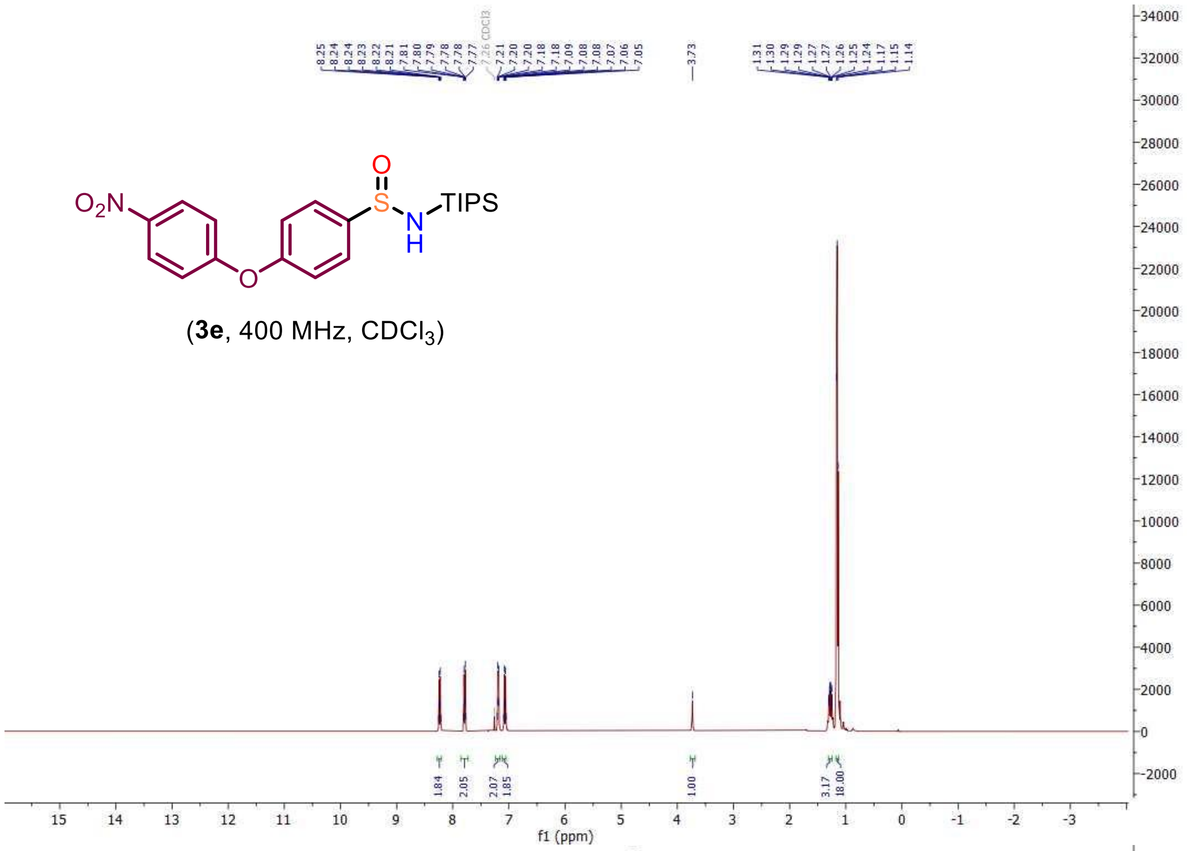

O2N
TIPS
(3e, 400 MHz, CDCl3)
f1 (ppm)

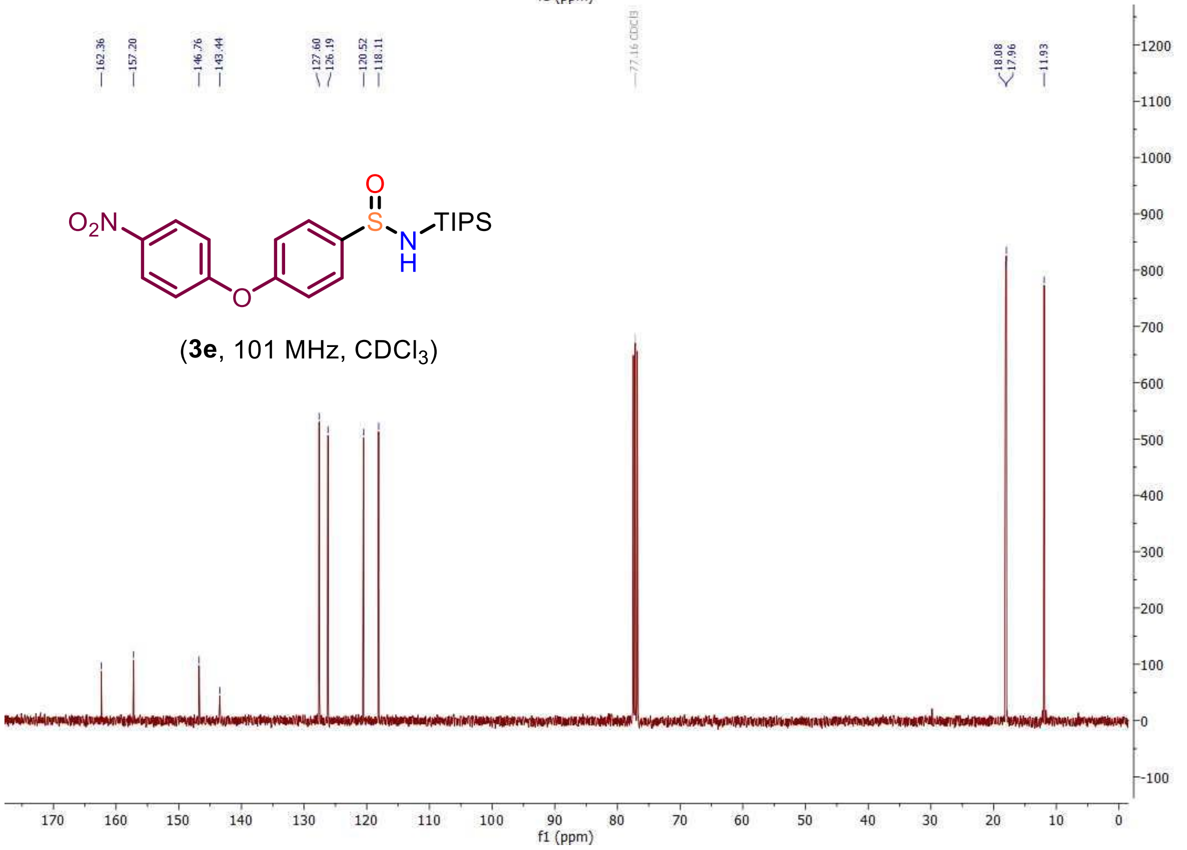

O2N
TIPS
(3e, 101 MHz, CDCl3)
f1 (ppm)

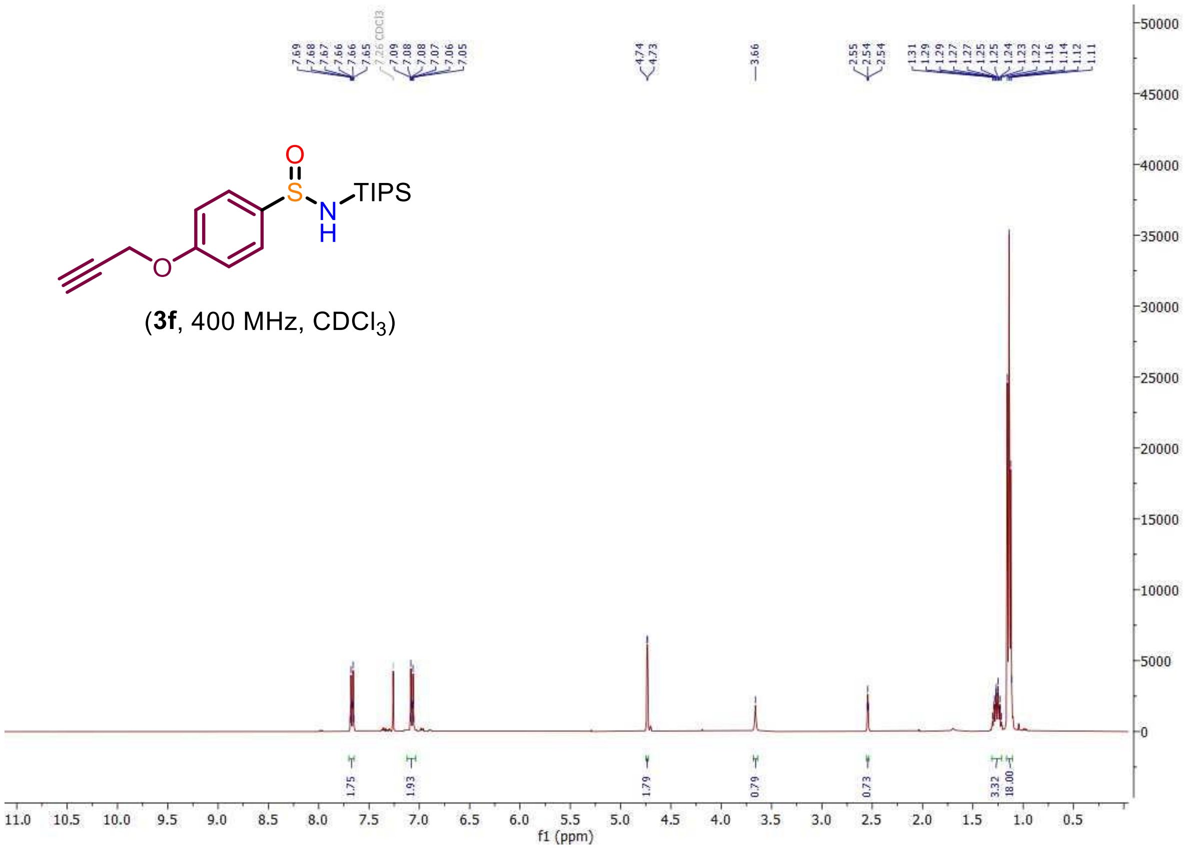

(**3f**, 400 MHz, $CDCl_3$)

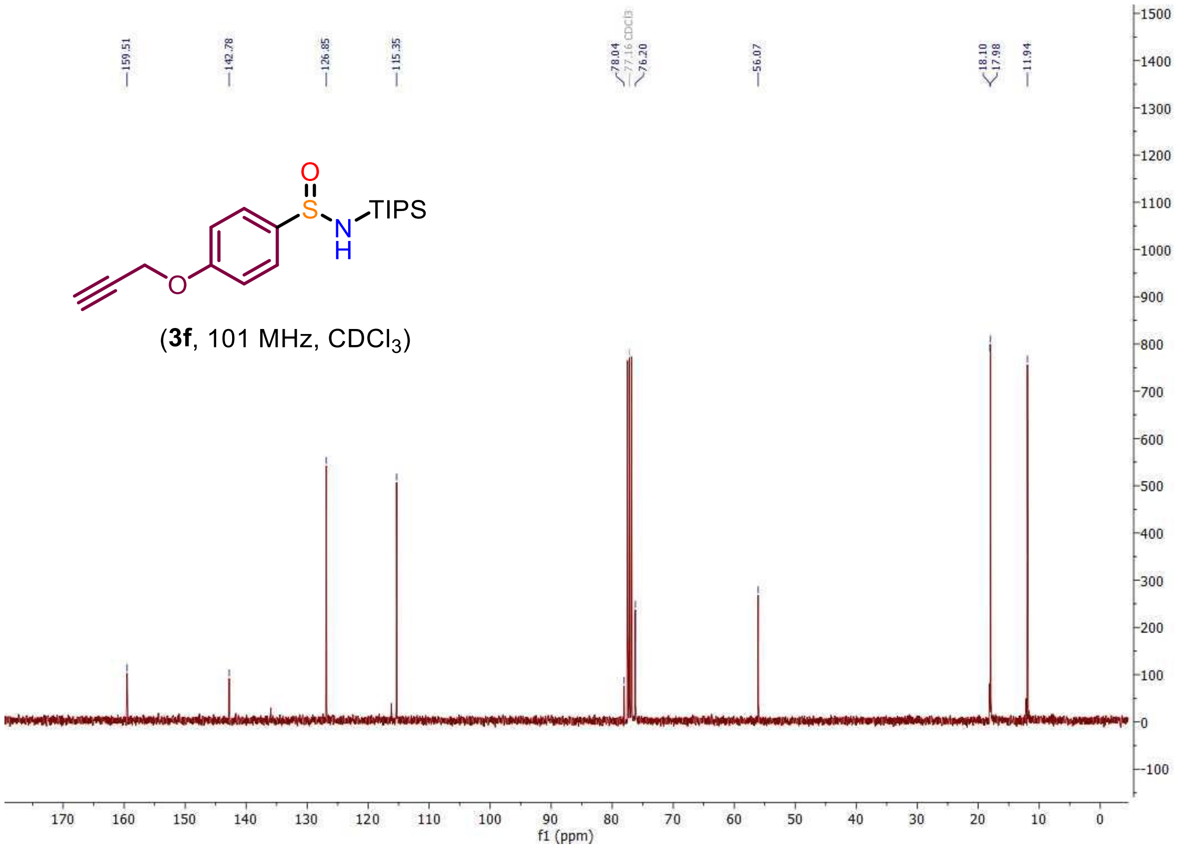

(**3f**, 101 MHz, $CDCl_3$)

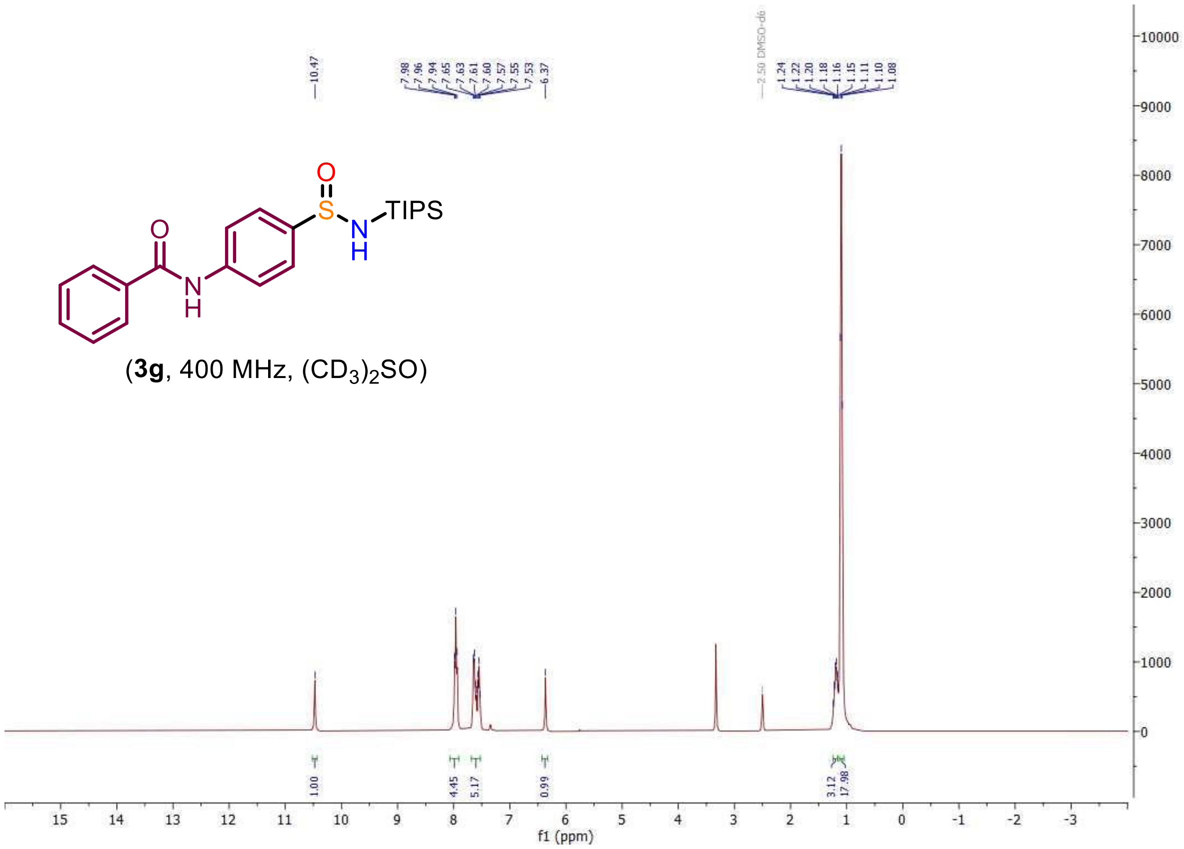

(**3g**, 400 MHz, $(CD_3)_2SO$)

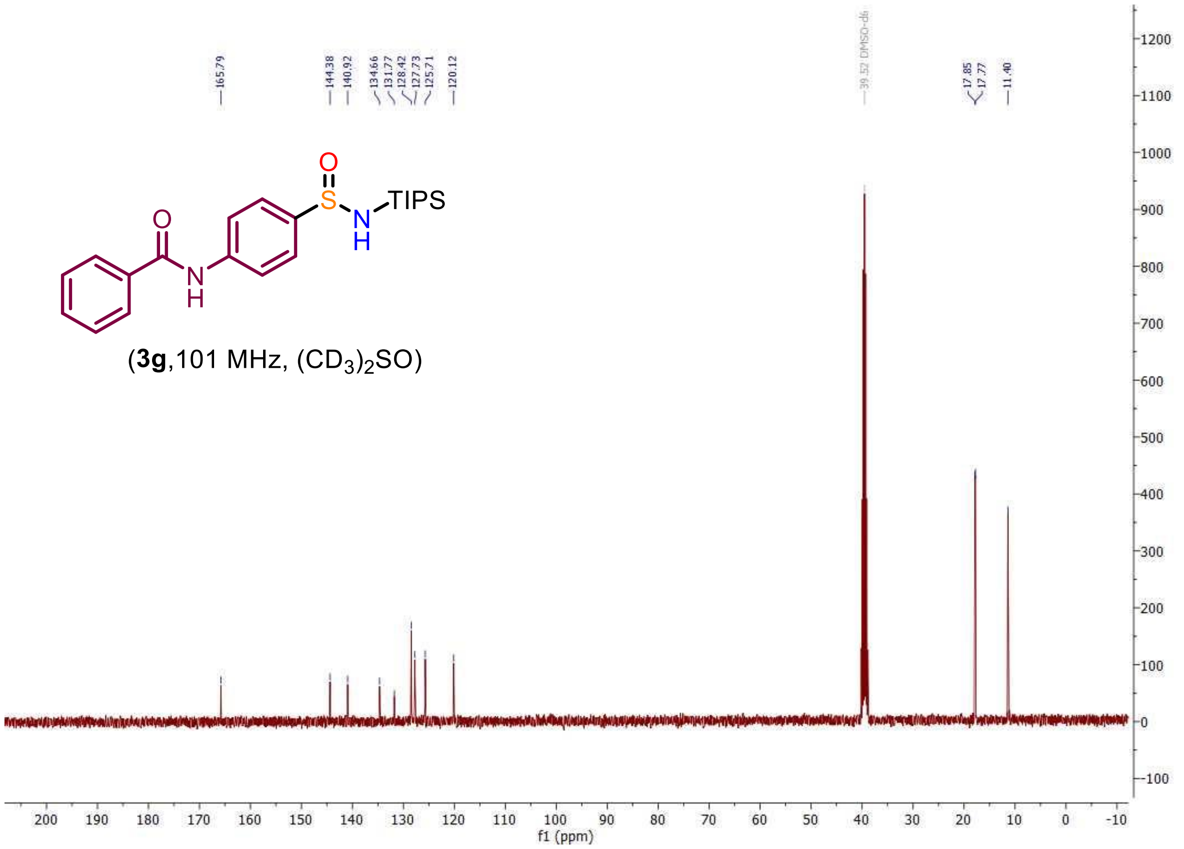

(**3g**,101 MHz, $(CD_3)_2SO$)

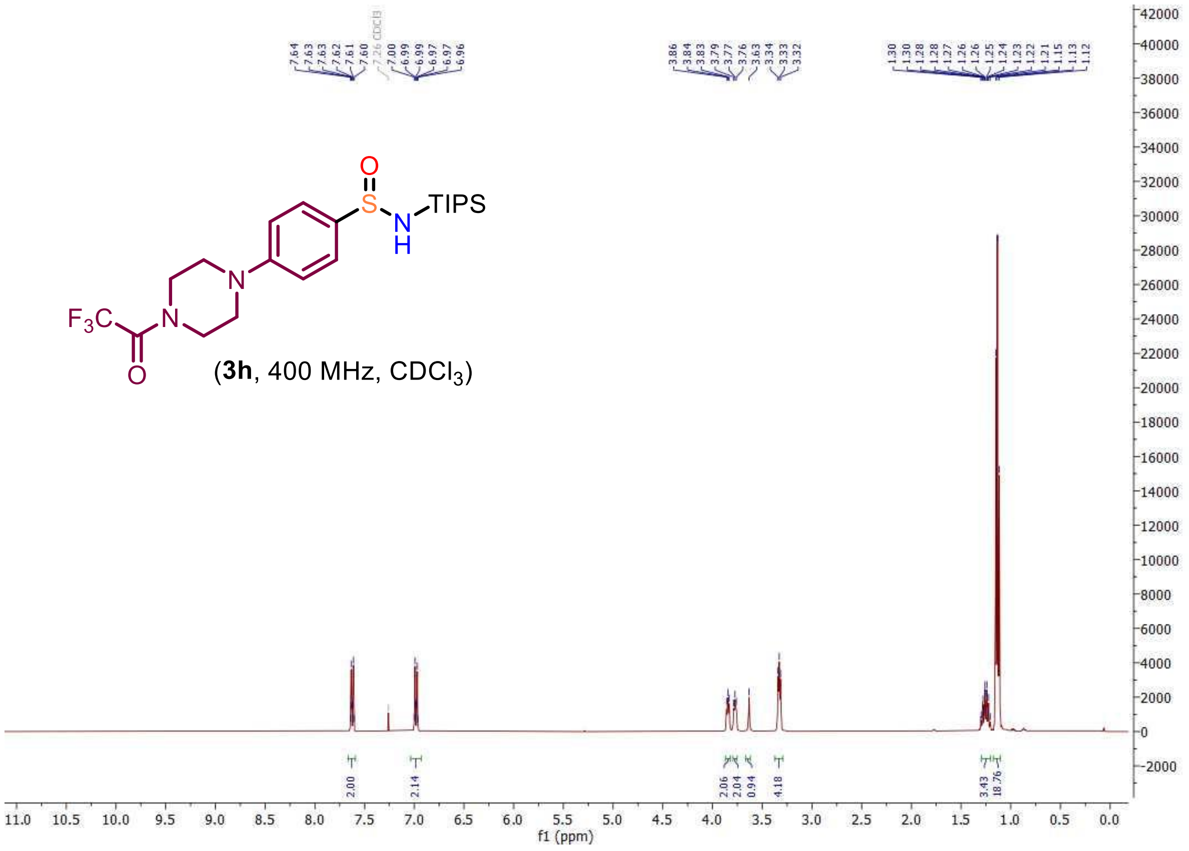
TIPS
F3C
(3h, 400 MHz, CDCl3)
7.64 7.63 7.63 7.62 7.61 7.60 7.26 CDCl3 7.00 6.99 6.99 6.97 6.97 6.96
3.86 3.84 3.83 3.79 3.77 3.76 3.63 3.34 3.33 3.32
1.30 1.30 1.28 1.28 1.27 1.26 1.26 1.25 1.24 1.23 1.22 1.21 1.15 1.13 1.12
2.00 2.14 2.06 2.04 0.94 4.18 3.43 18.76
f1 (ppm)

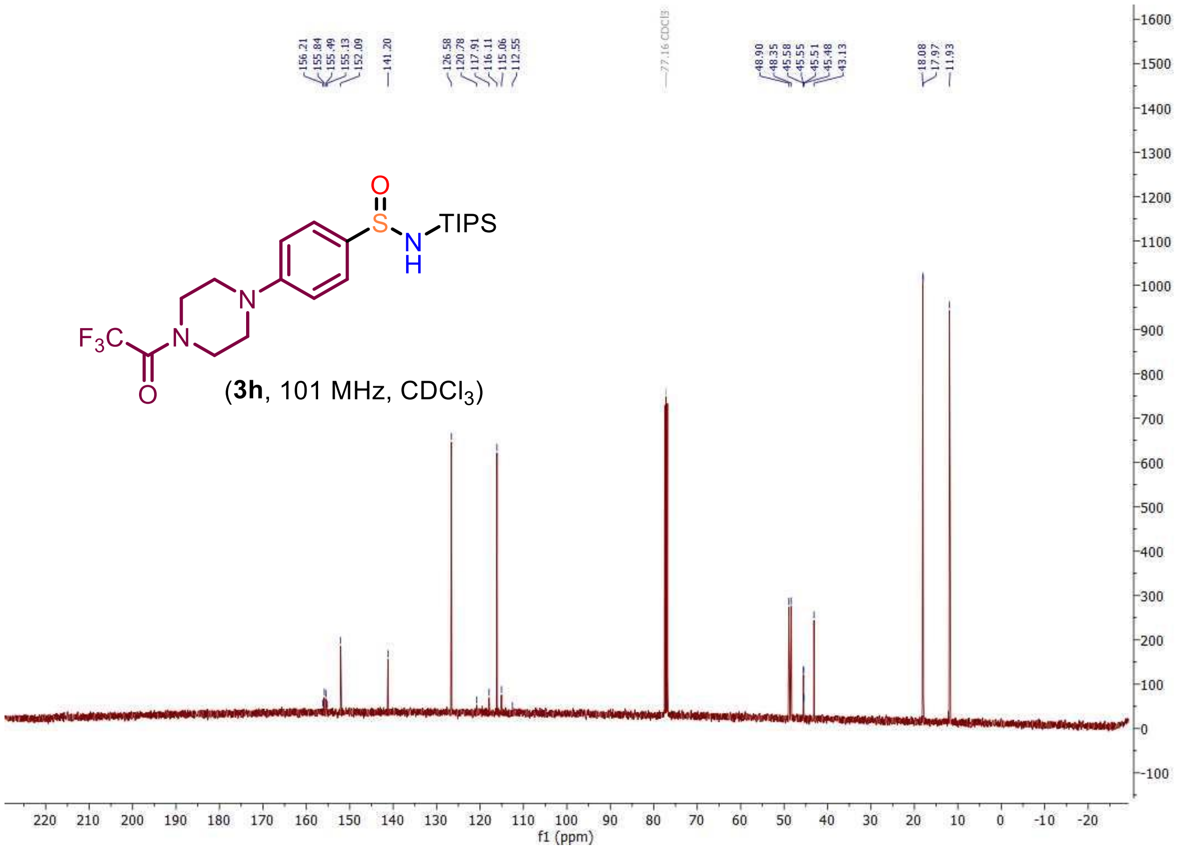
TIPS
F3C
(3h, 101 MHz, CDCl3)
156.21 155.84 155.49 155.13 152.09 141.20 126.58 120.78 117.91 116.11 115.06 112.55
77.16 CDCl3
48.90 48.35 45.58 45.55 45.51 45.48 43.13
18.08 17.97 11.93
f1 (ppm)

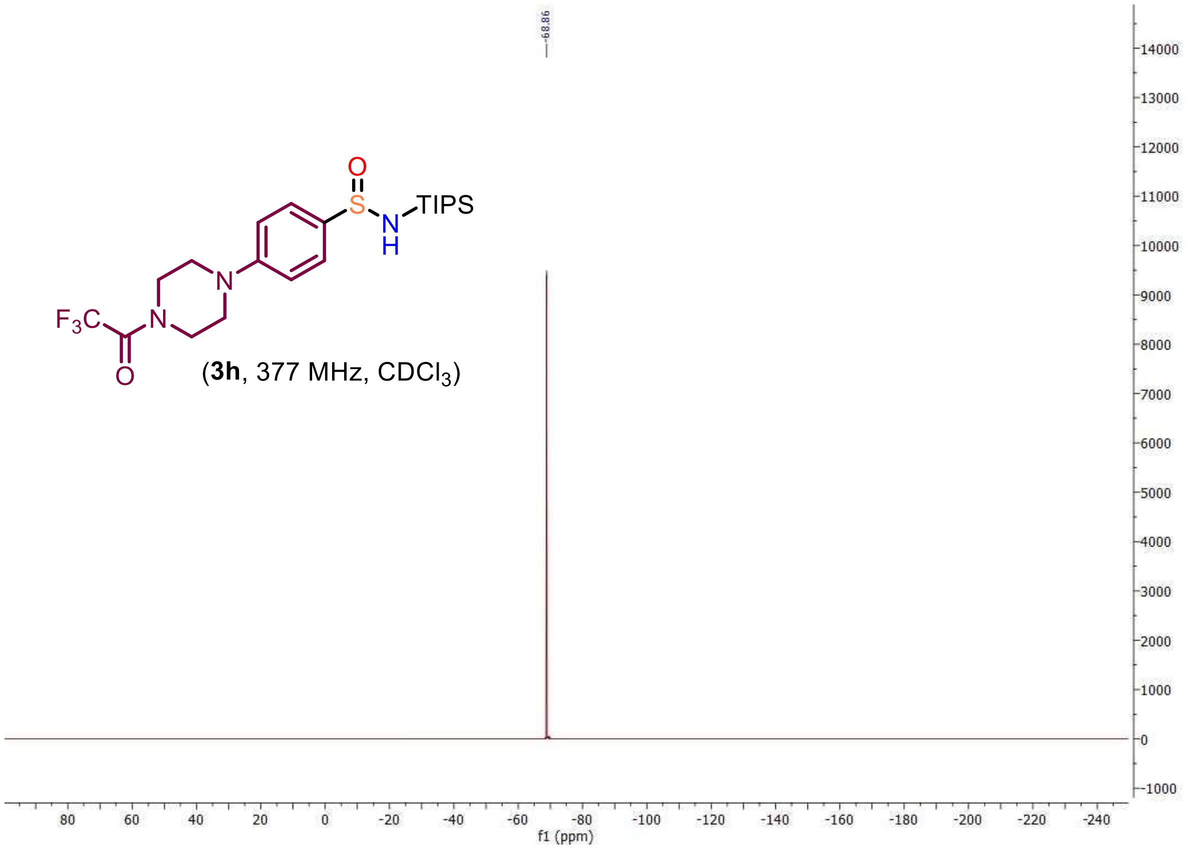

-68.86
TIPS
(3h, 377 MHz, CDCl3)
f1 (ppm)

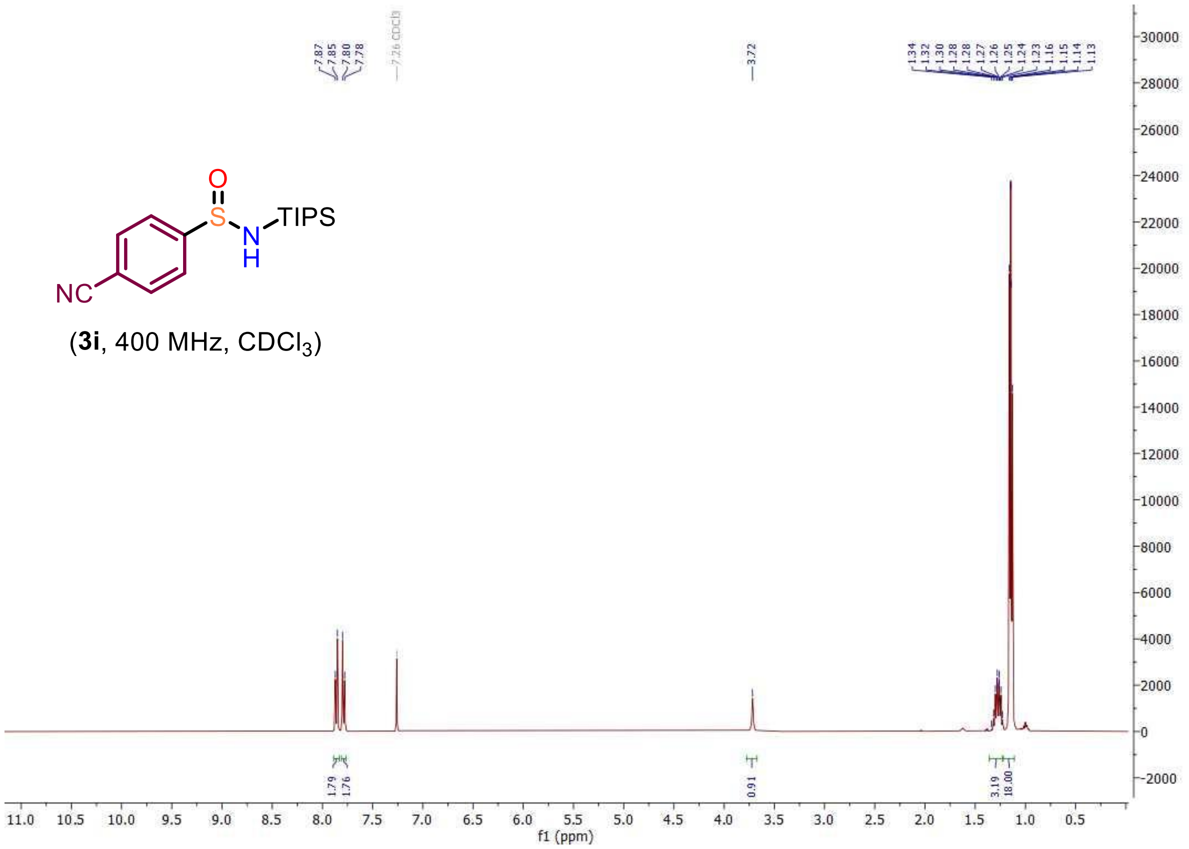

7.87
7.85
7.80
7.78
7.26 CDCl3
3.72
1.34
1.32
1.30
1.28
1.28
1.27
1.26
1.25
1.24
1.23
1.16
1.15
1.14
1.13
TIPS
(3i, 400 MHz, CDCl3)
1.79
1.76
0.91
3.19
18.00
f1 (ppm)

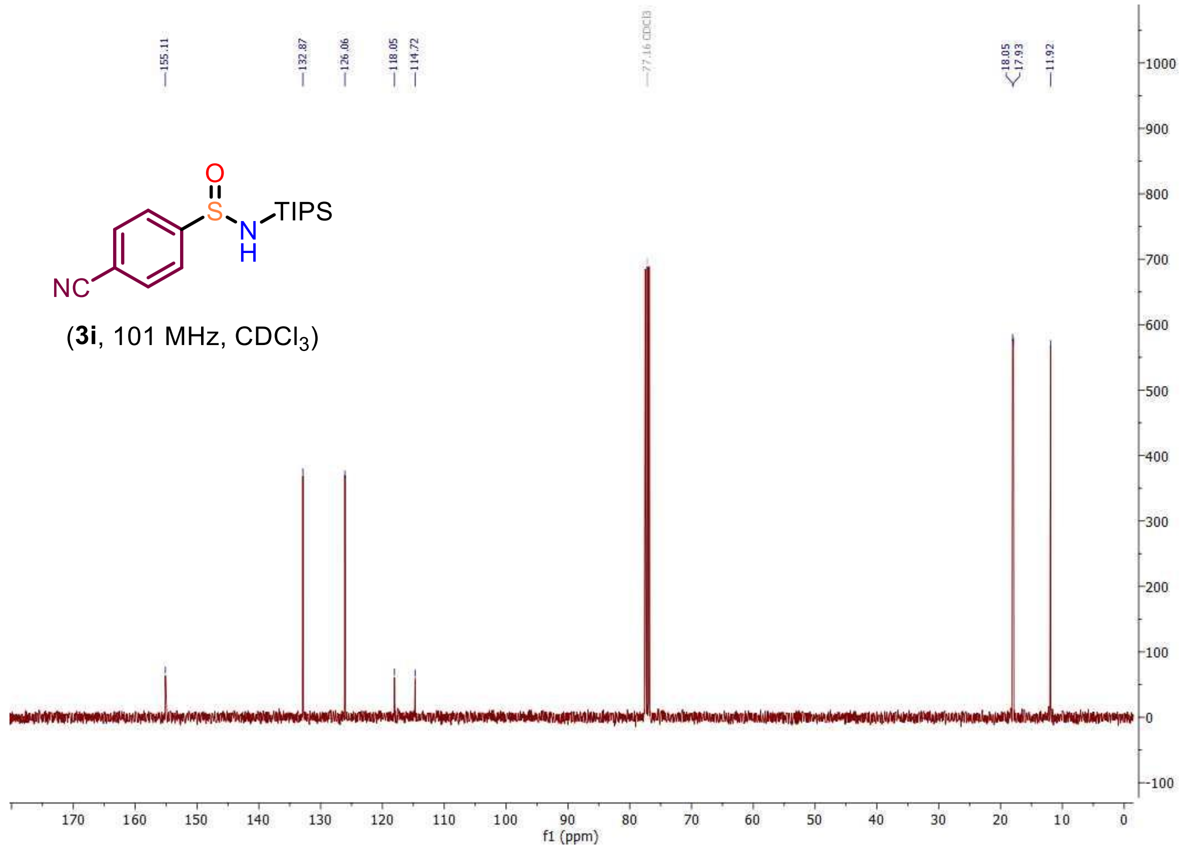
155.11
132.87
126.06
118.05
114.72
77.16 CDCl3
18.05
17.93
11.92
NC
O
S
N
H
TIPS
(3i, 101 MHz, CDCl3)
f1 (ppm)

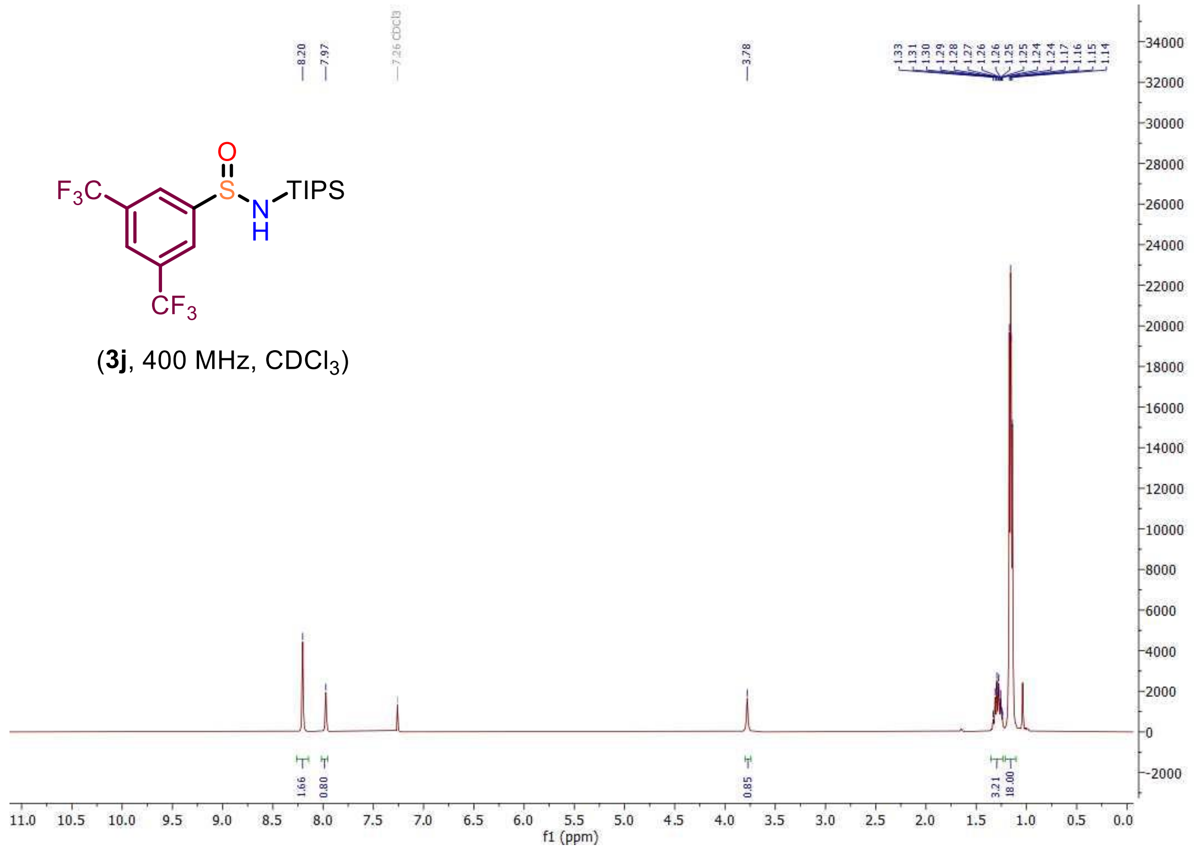
8.20
7.97
7.26 CDCl3
3.78
F3C
CF3
O
S
N
H
TIPS
(3j, 400 MHz, CDCl3)
1.66
0.80
0.85
3.21
18.00
f1 (ppm)

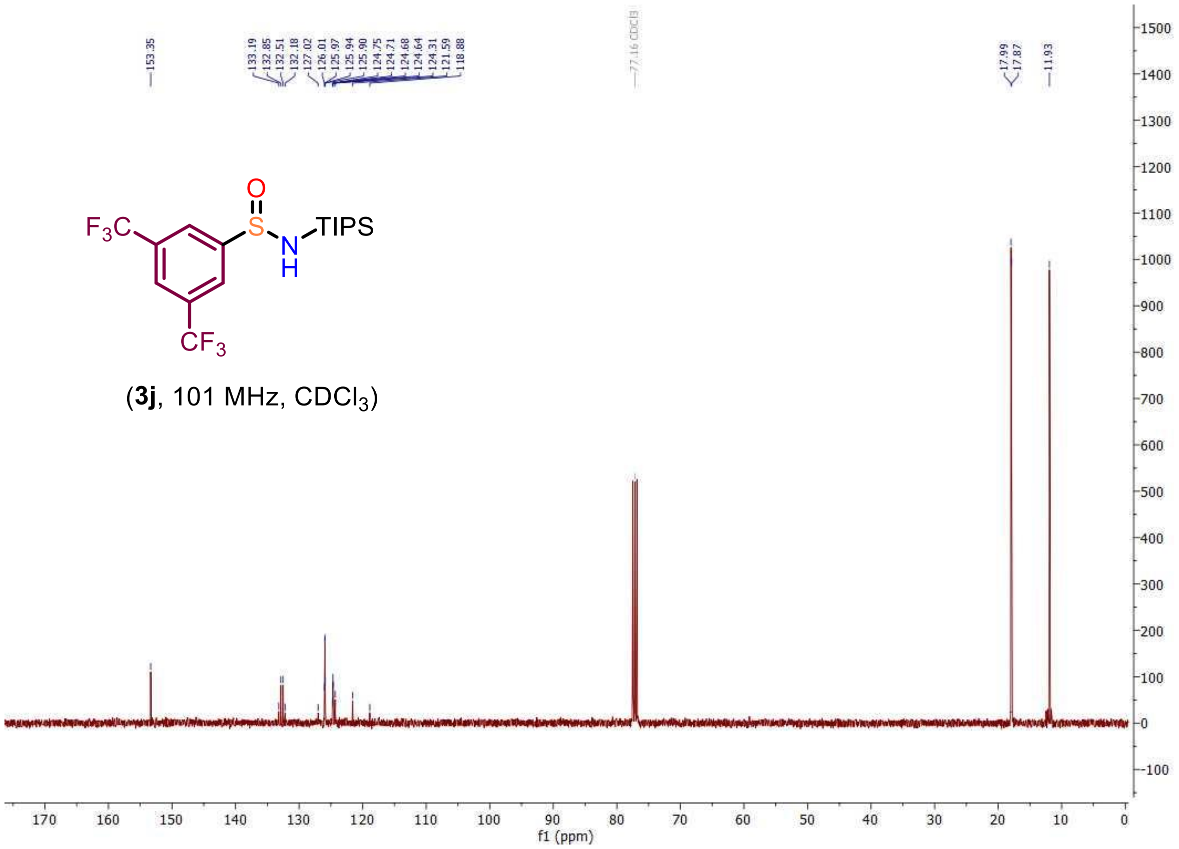
153.35
133.19
132.85
132.51
132.18
127.02
126.01
125.97
125.94
125.90
124.75
124.71
124.68
124.64
124.31
121.59
118.88
77.16 CDCl3
17.99
17.87
11.93
F3C
O
S
N
H
TIPS
CF3
(3j, 101 MHz, CDCl3)
f1 (ppm)

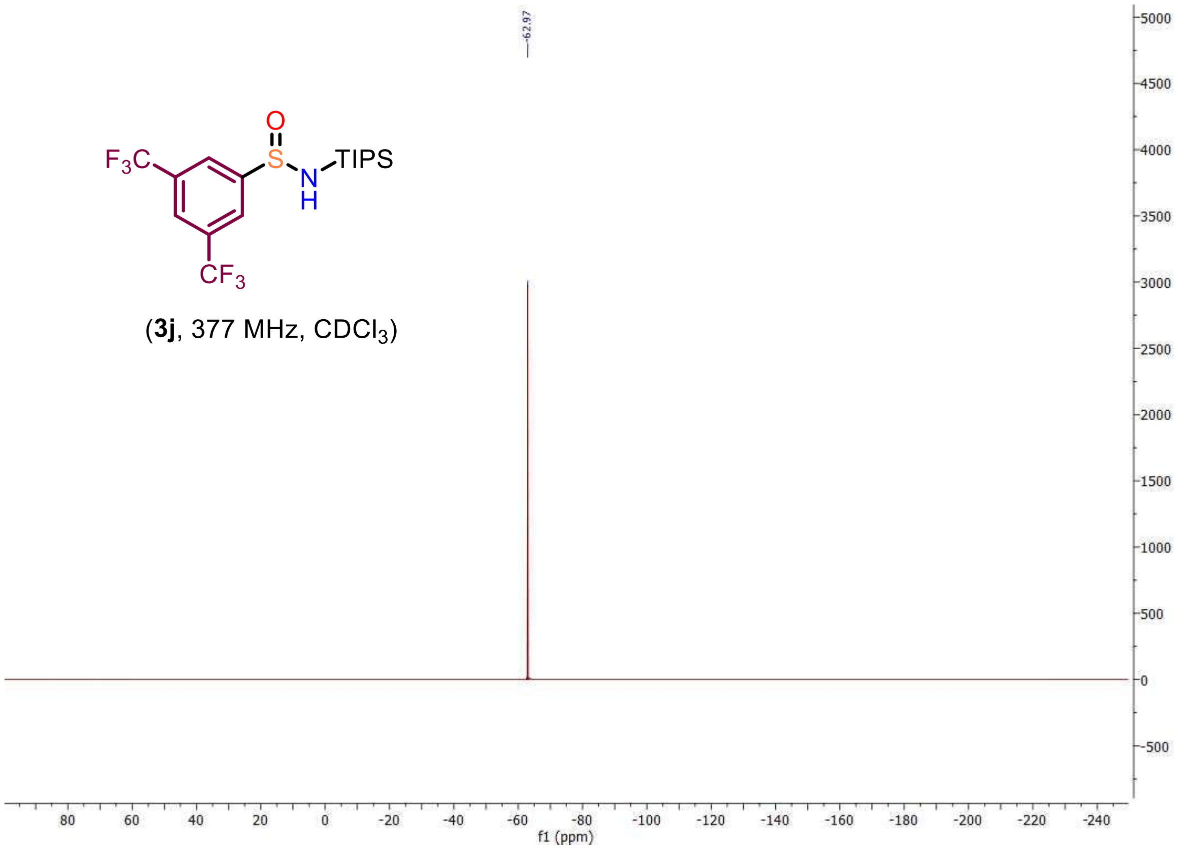
-62.97
F3C
O
S
N
H
TIPS
CF3
(3j, 377 MHz, CDCl3)
f1 (ppm)

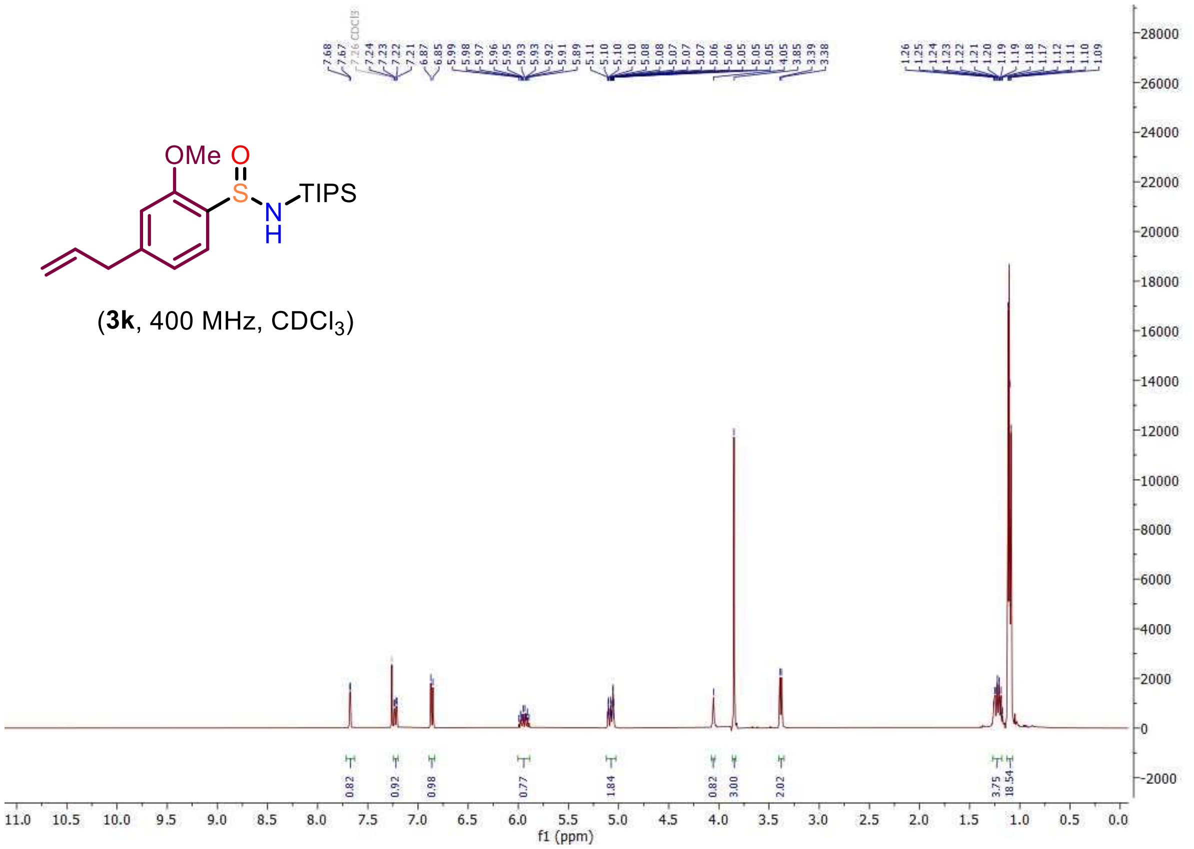

(**3k**, 400 MHz, $CDCl_3$)

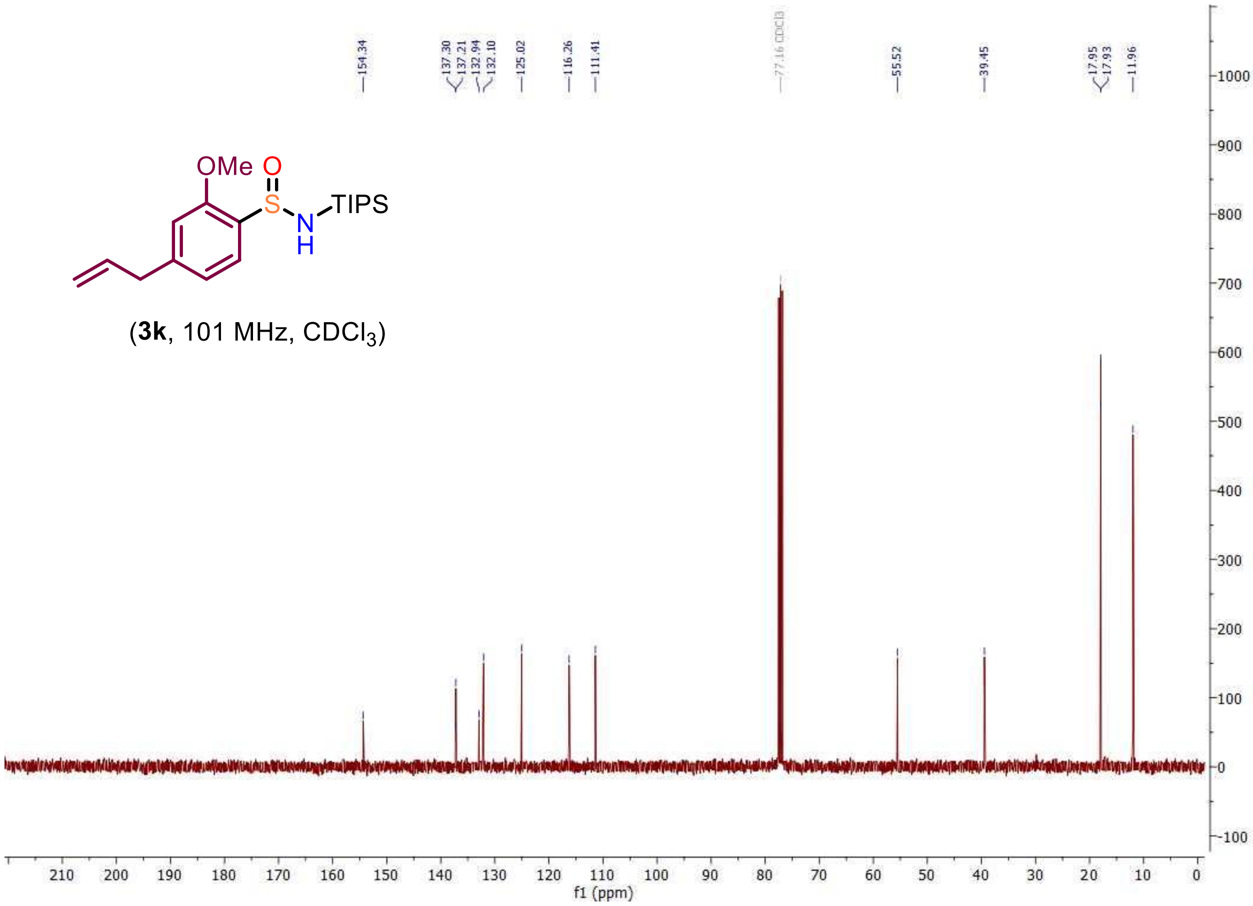

(**3k**, 101 MHz, $CDCl_3$)

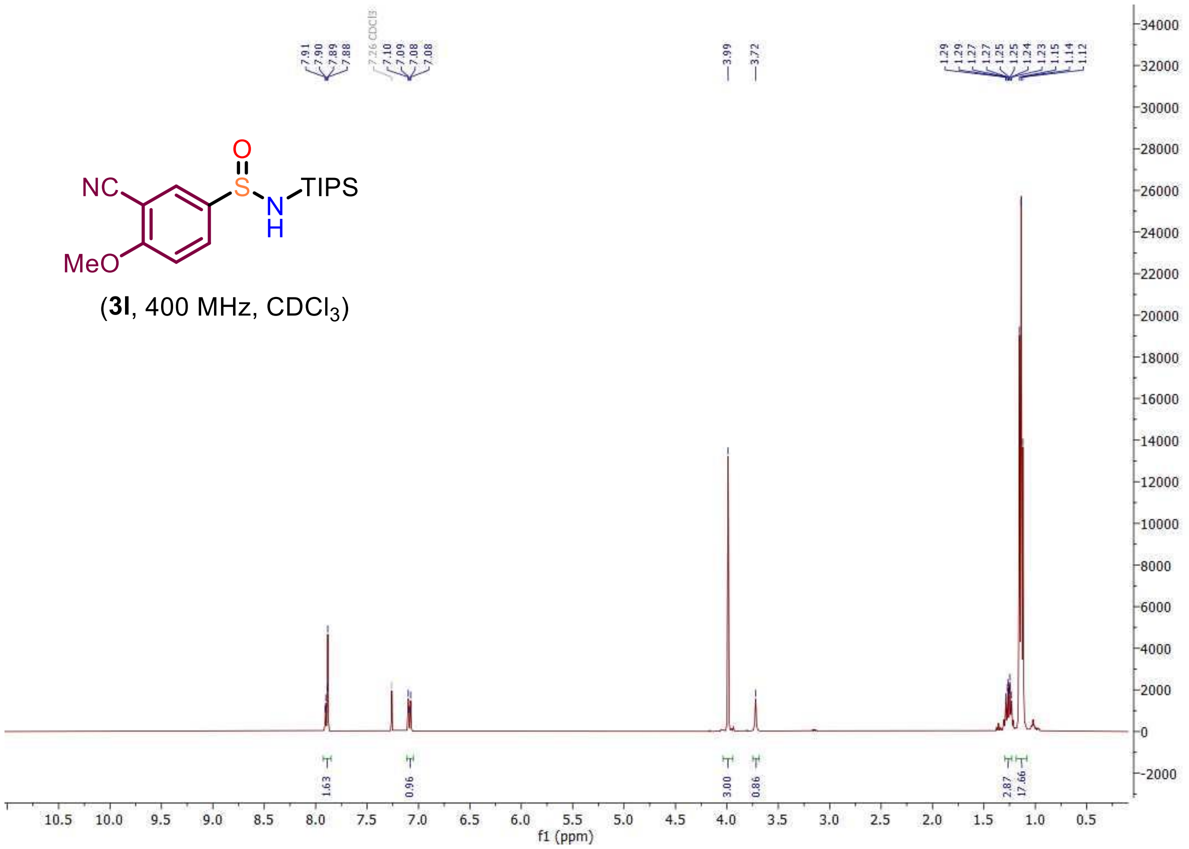

7.91
7.90
7.89
7.88
7.26 CDCl3
7.10
7.09
7.08
7.08
3.99
3.72
1.29
1.29
1.27
1.27
1.25
1.25
1.24
1.23
1.15
1.14
1.12
NC
MeO
O
S
N
H
TIPS
(3l, 400 MHz, CDCl3)
1.63
0.96
3.00
0.86
2.87
17.66
f1 (ppm)

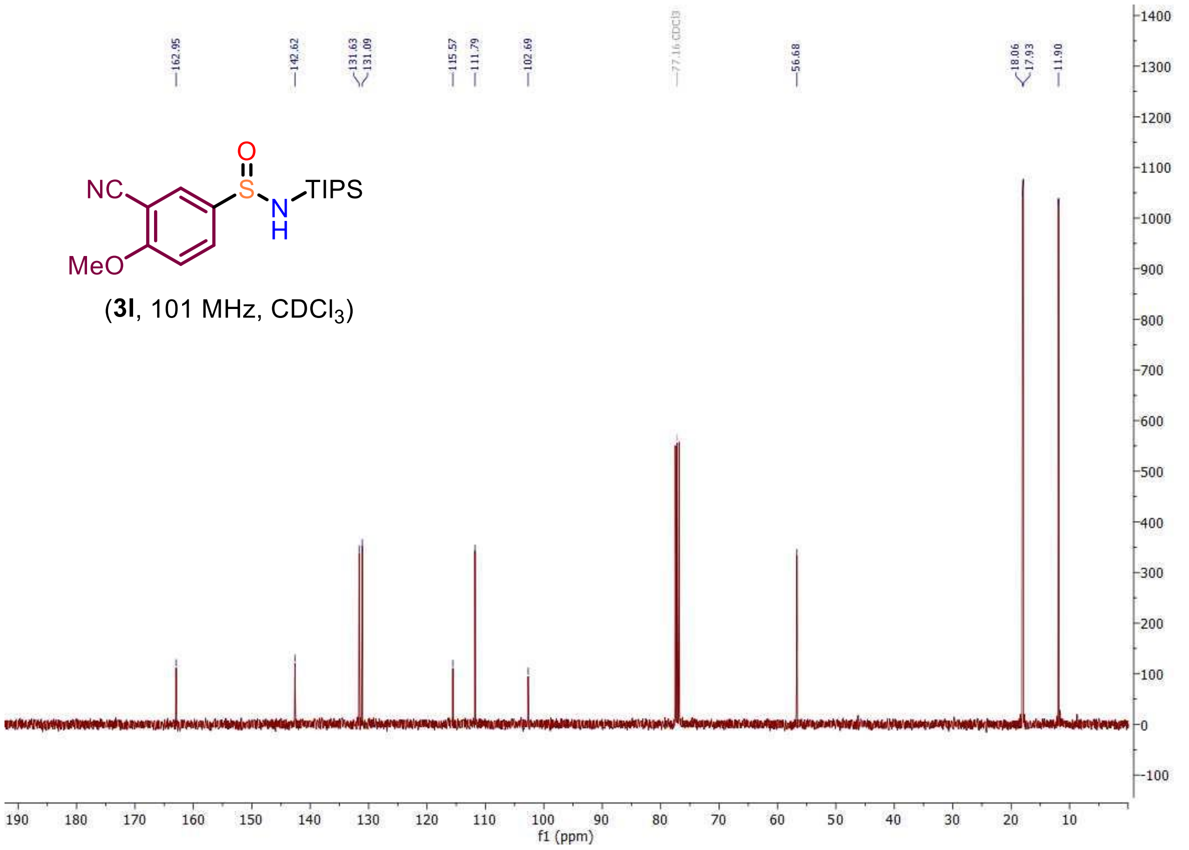

162.95
142.62
131.63
131.09
115.57
111.79
102.69
77.16 CDCl3
56.68
18.06
17.93
11.90
NC
MeO
O
S
N
H
TIPS
(3l, 101 MHz, CDCl3)
f1 (ppm)

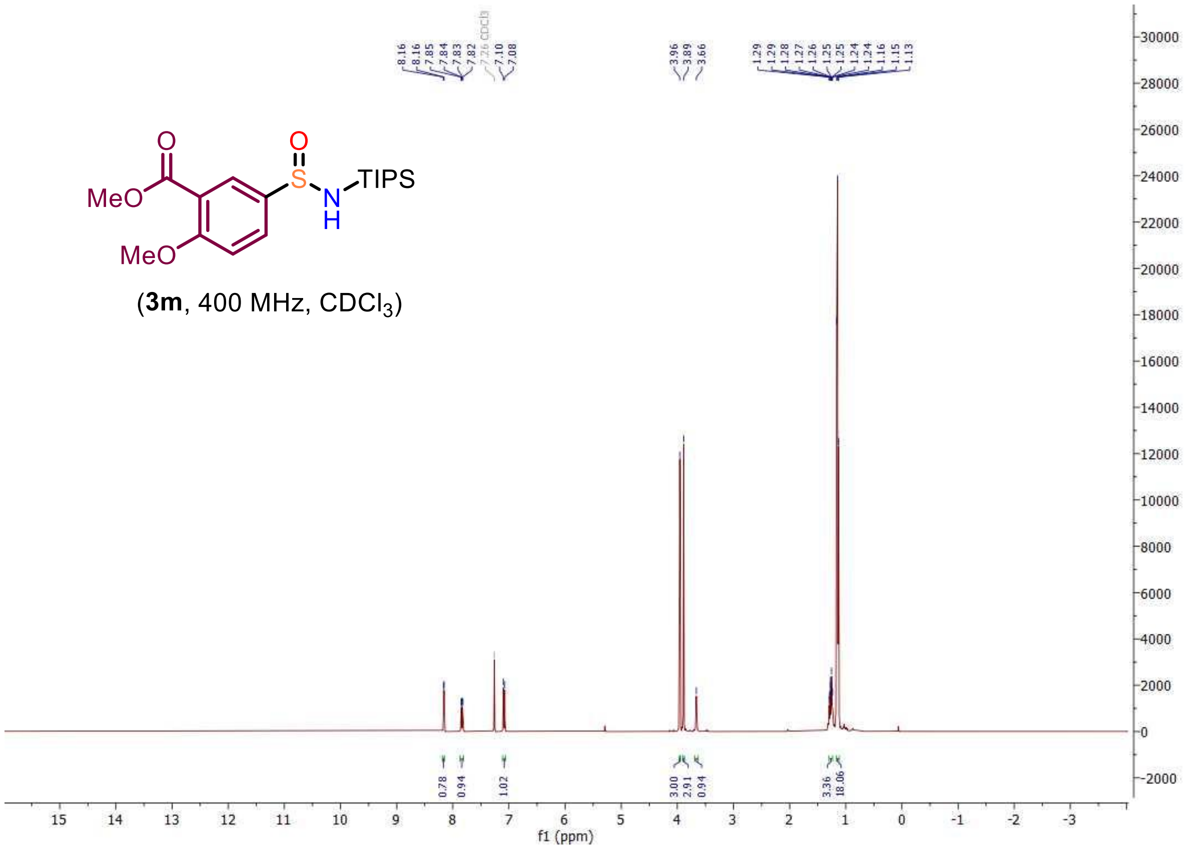

(**3m**, 400 MHz, $CDCl_3$)

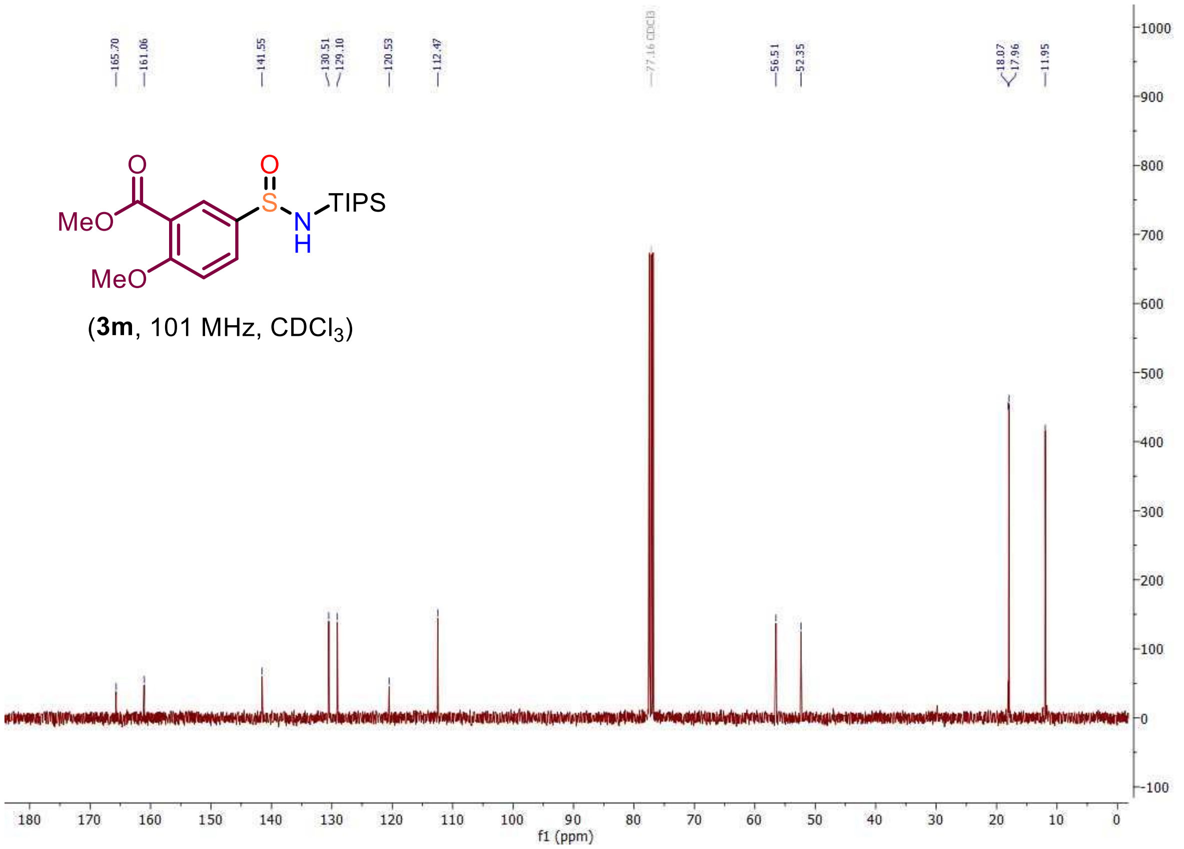

(**3m**, 101 MHz, $CDCl_3$)

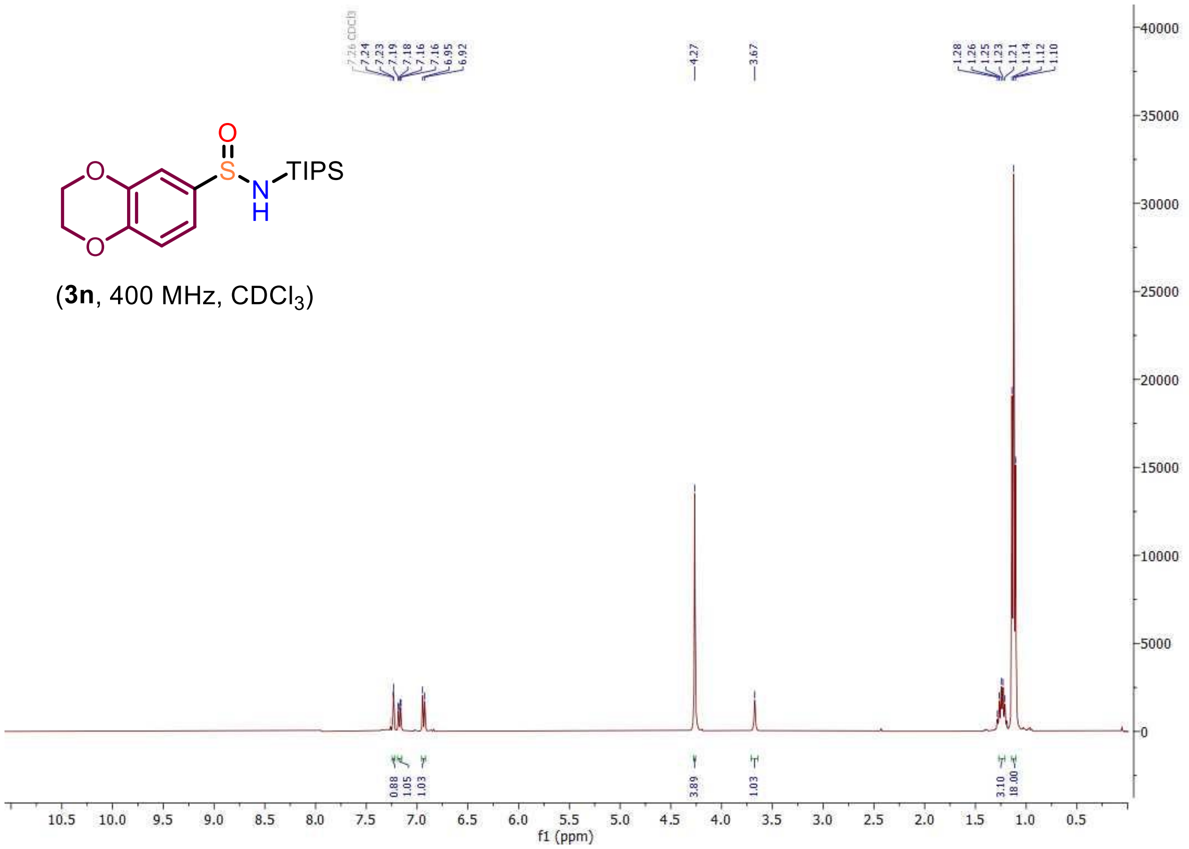

($\mathbf{3n}$, 400 MHz, $CDCl_3$)

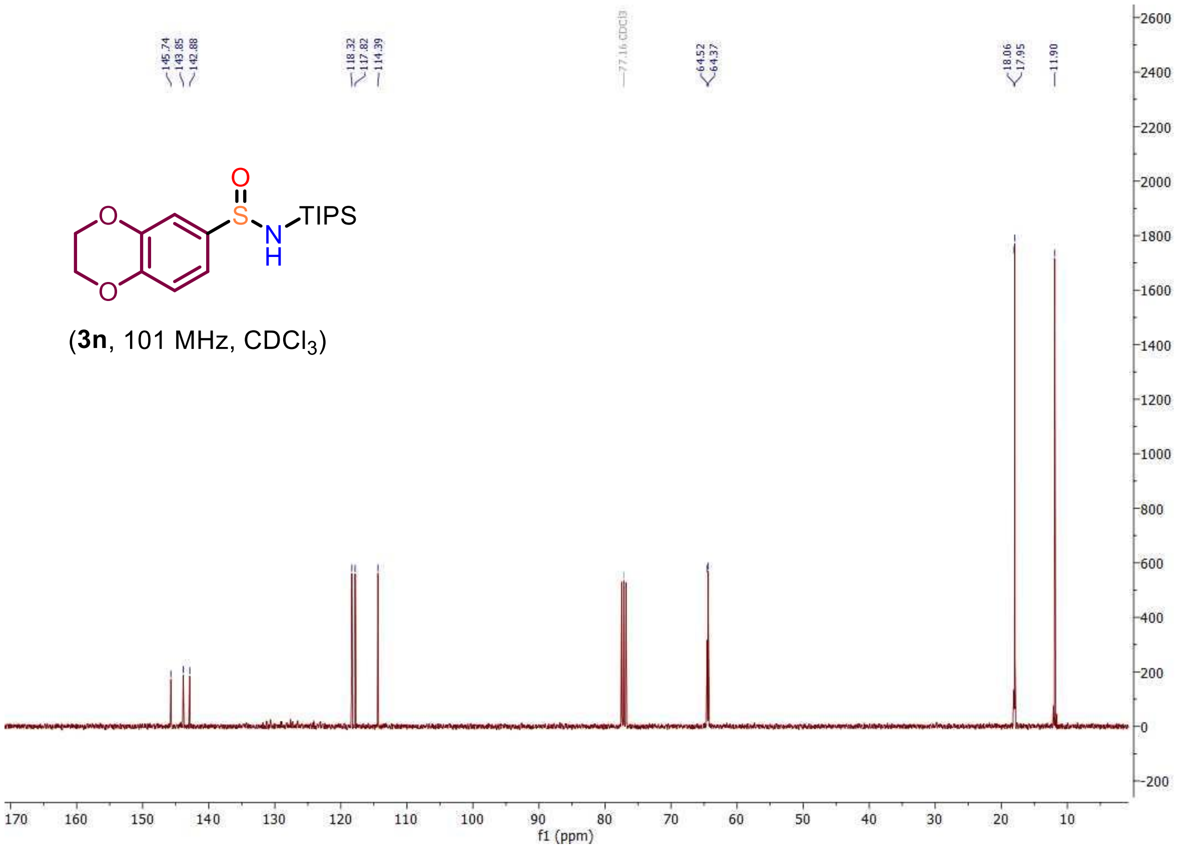

($\mathbf{3n}$, 101 MHz, $CDCl_3$)

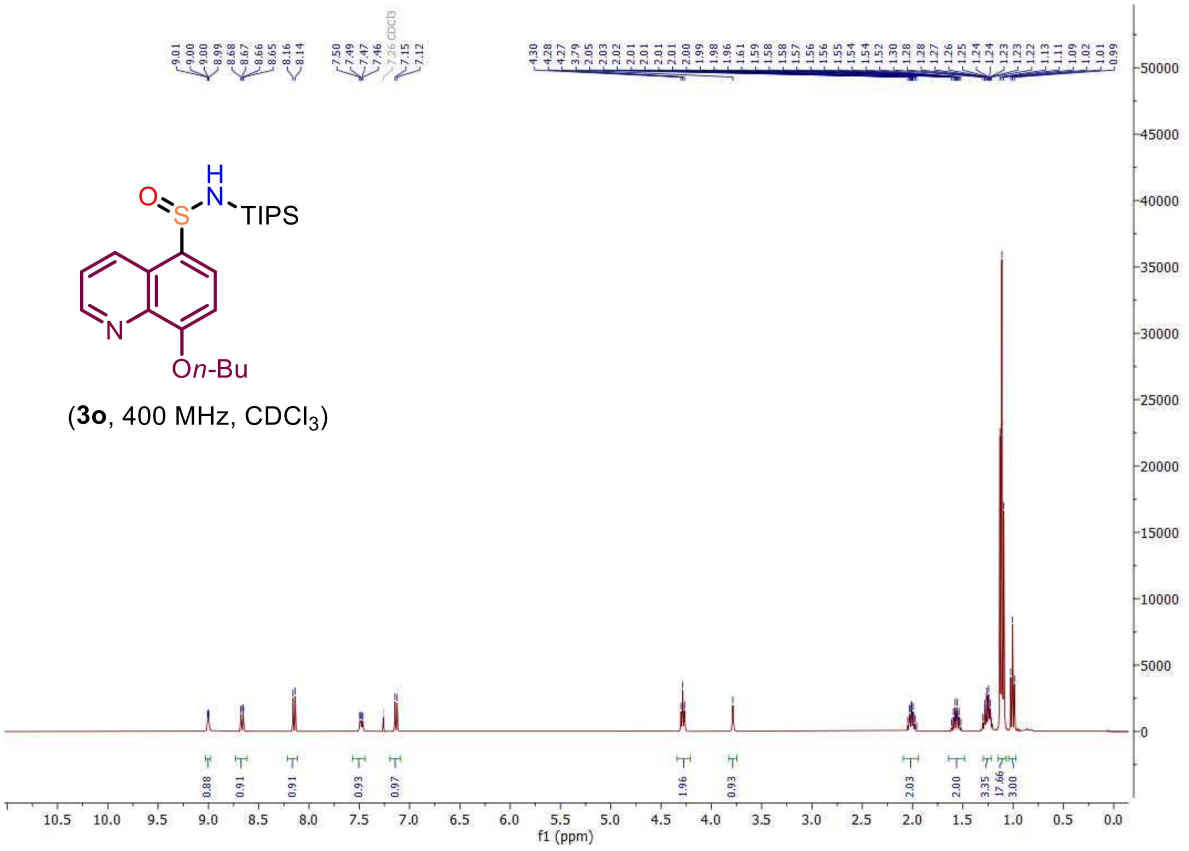
O
S
H
N
TIPS
N
On-Bu
(**3o**, 400 MHz, $CDCl_3$)
9.01 9.00 9.00 8.99 8.68 8.67 8.66 8.65 8.16 8.14
7.50 7.49 7.47 7.46 7.26 CDCl3 7.15 7.12
0.88 0.91 0.91 0.93 0.97 1.96 0.93 2.03 2.00 3.35 17.66 3.00
f1 (ppm)

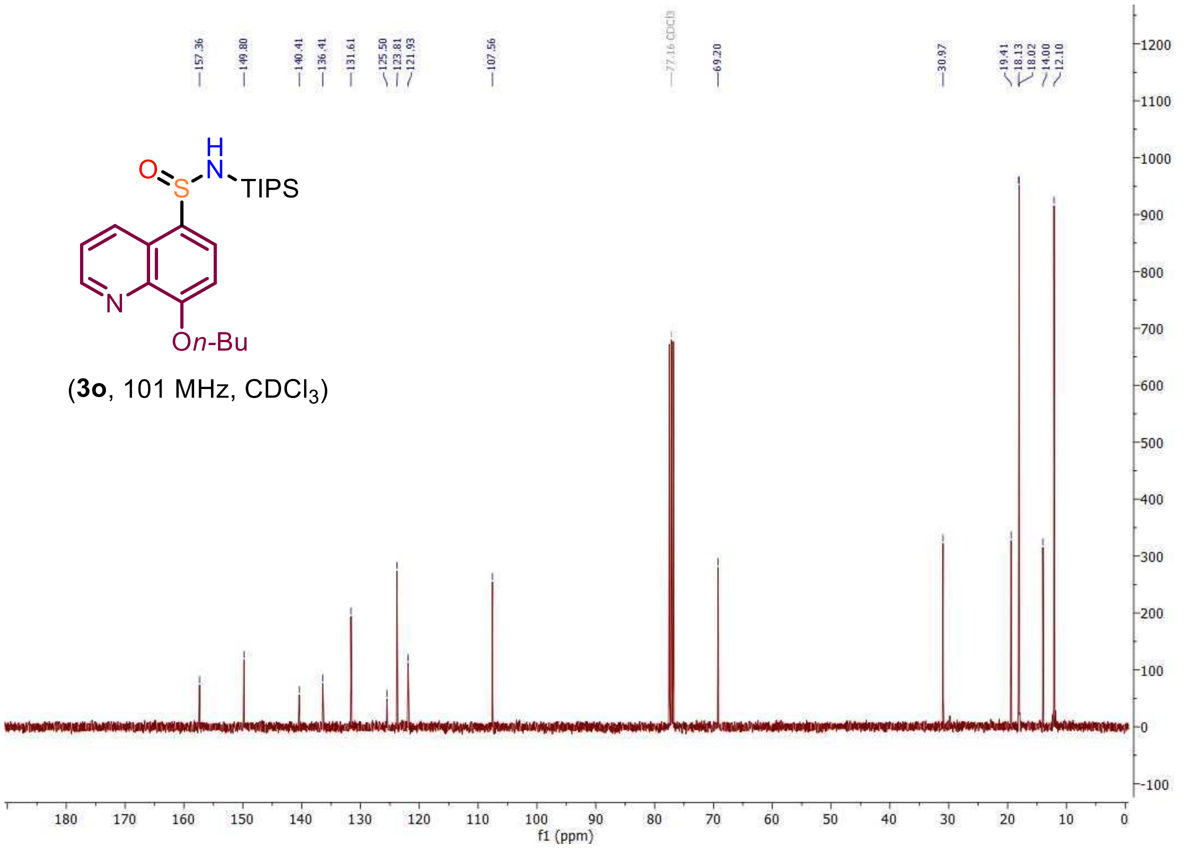
O
S
H
N
TIPS
N
On-Bu
(**3o**, 101 MHz, $CDCl_3$)
157.36
149.80
140.41
136.41
131.61
125.50
123.81
121.93
107.56
77.16 CDCl3
69.20
30.97
19.41
18.13
18.02
14.00
12.10
f1 (ppm)

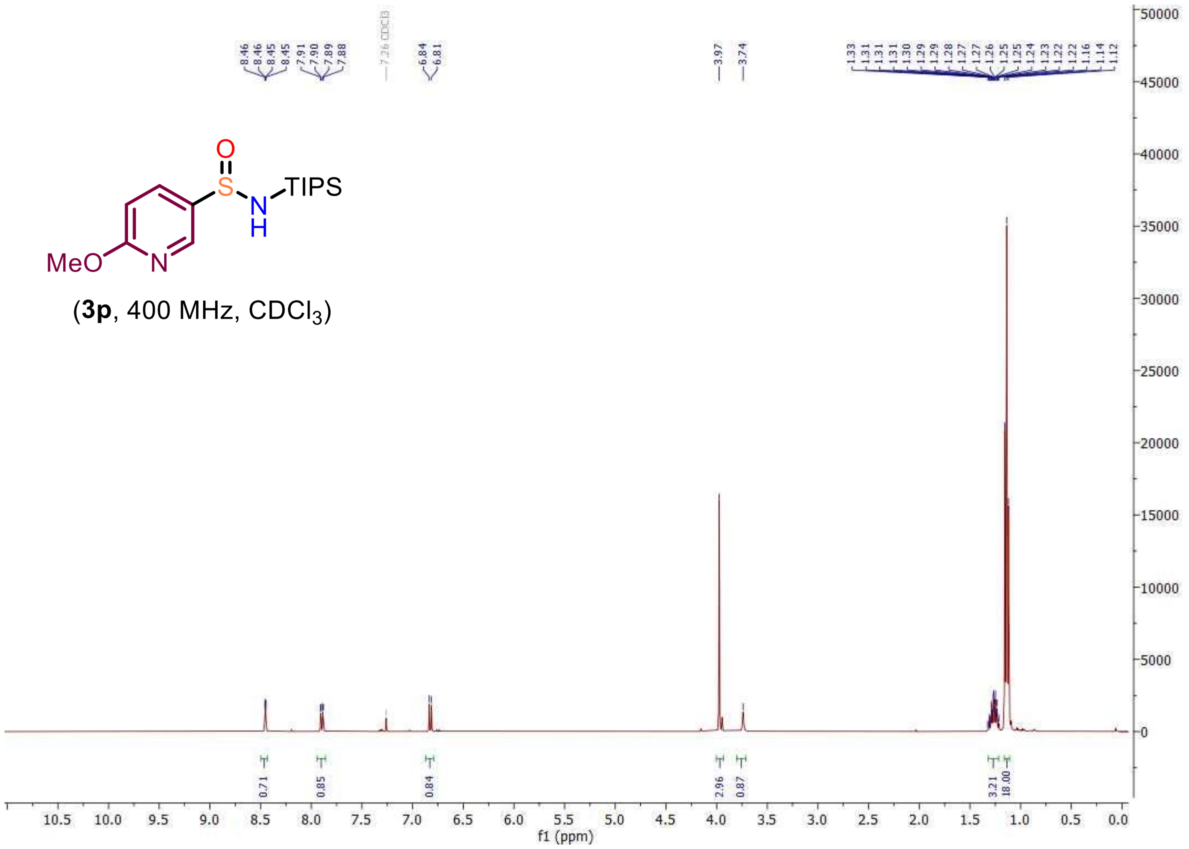
MeO
N
S
O
N
H
TIPS
(3p, 400 MHz, CDCl3)
f1 (ppm)

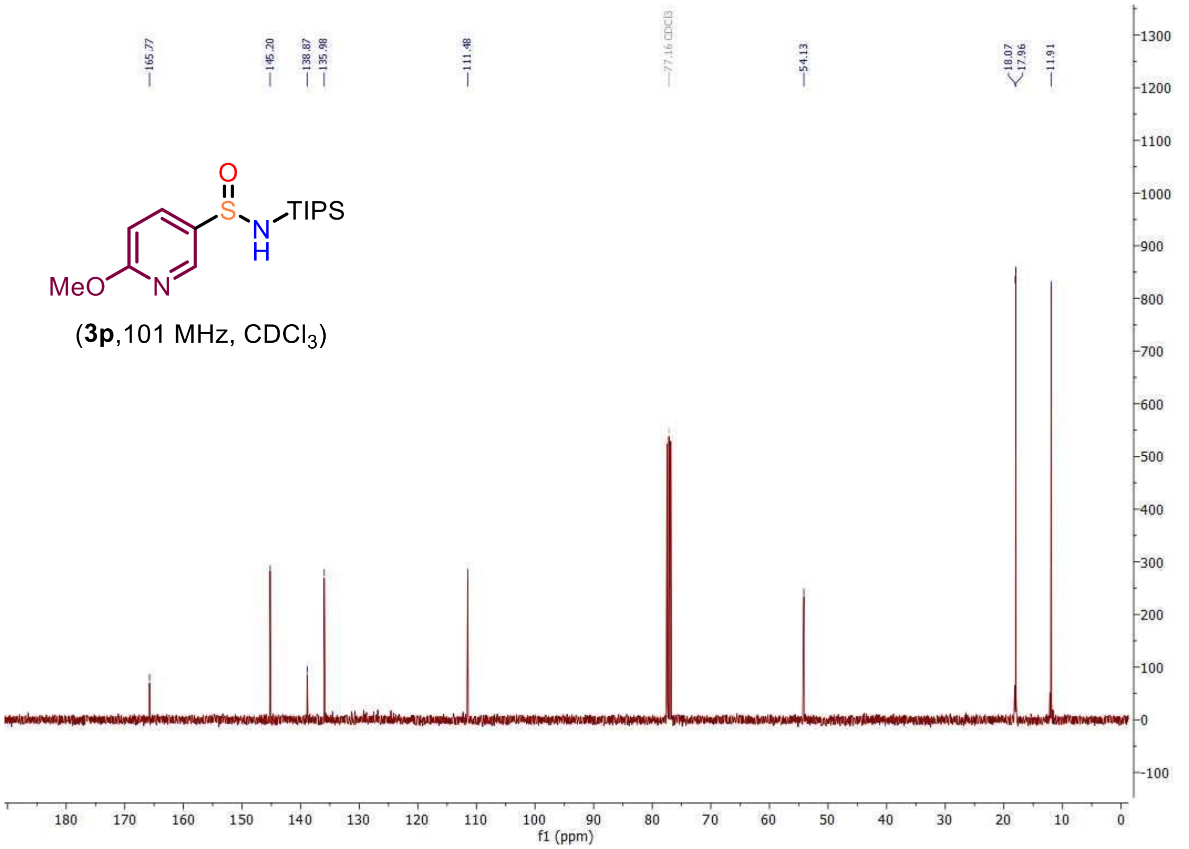
MeO
N
S
O
N
H
TIPS
(3p,101 MHz, CDCl3)
f1 (ppm)

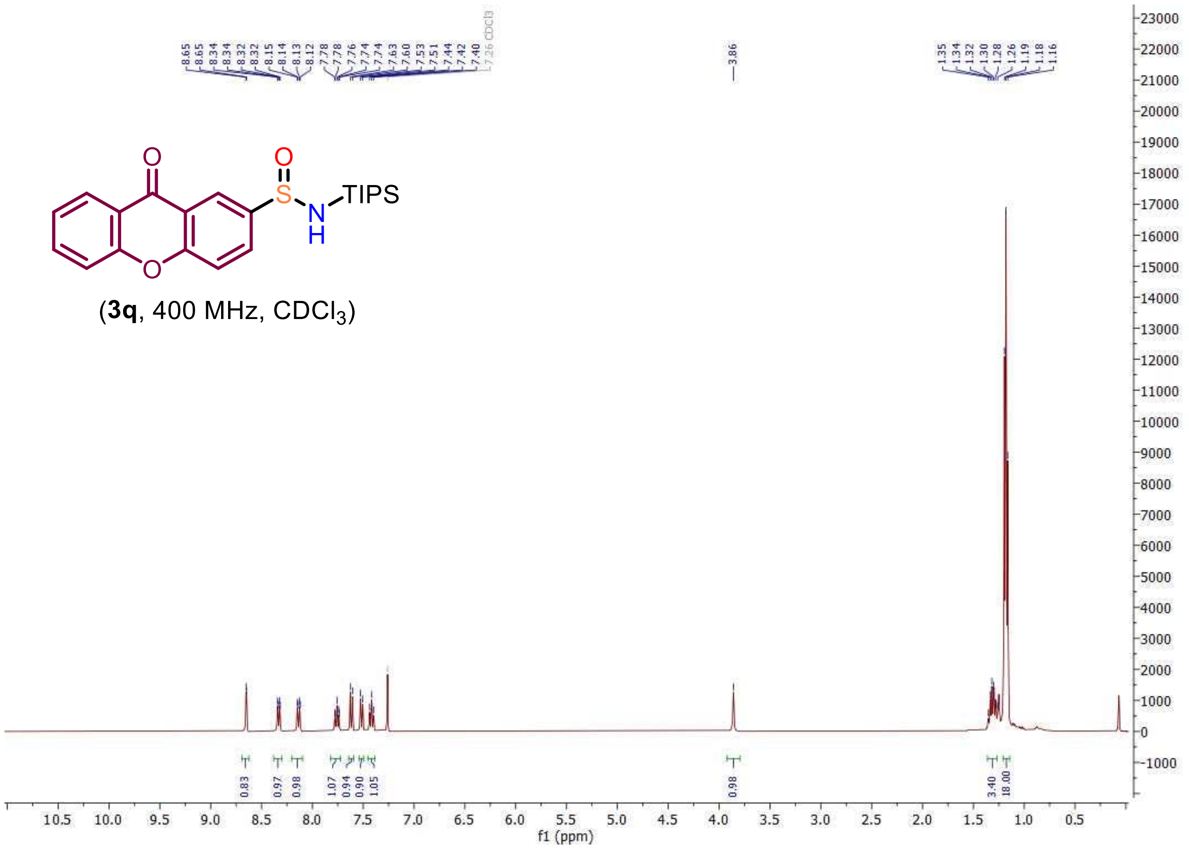

(**3q**, 400 MHz, $CDCl_3$)

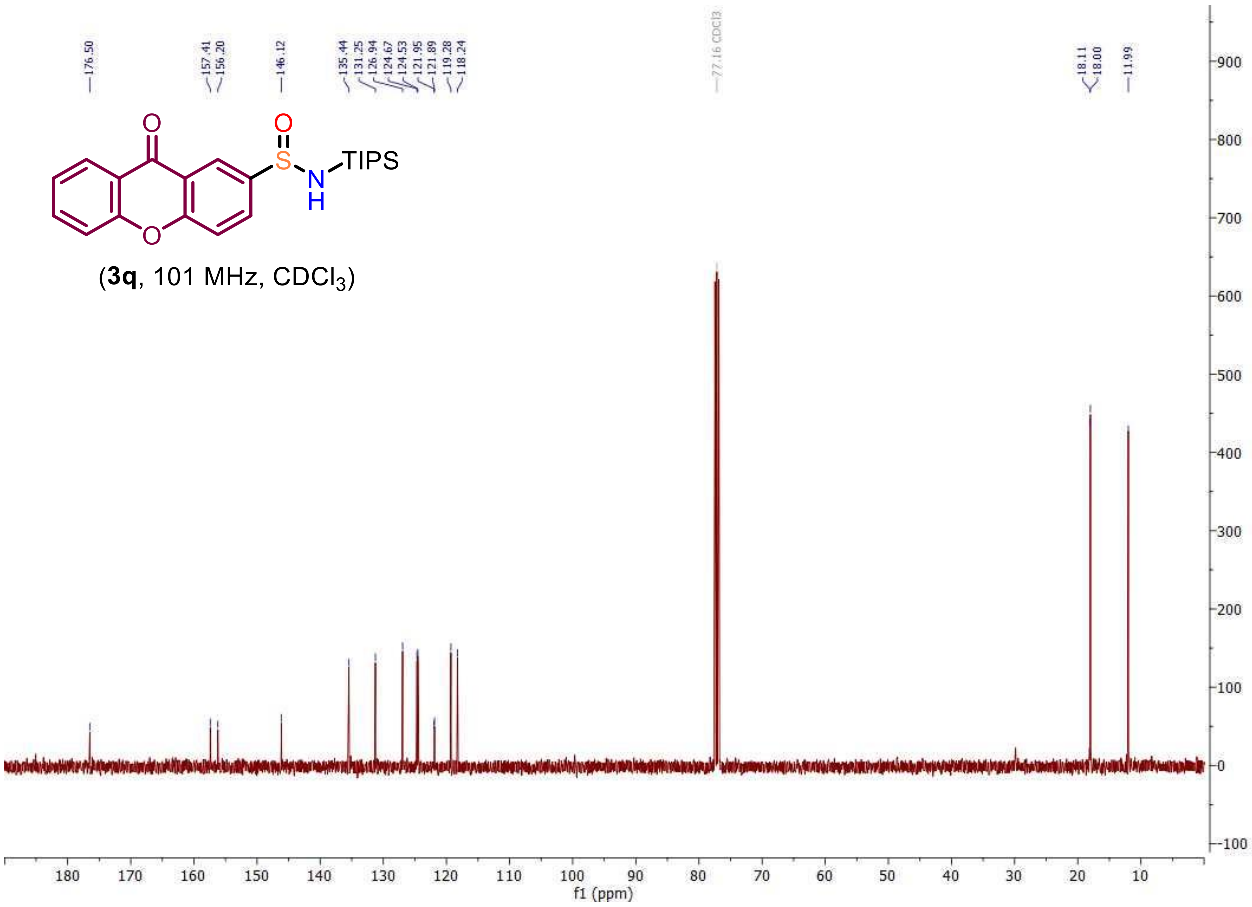

(**3q**, 101 MHz, $CDCl_3$)

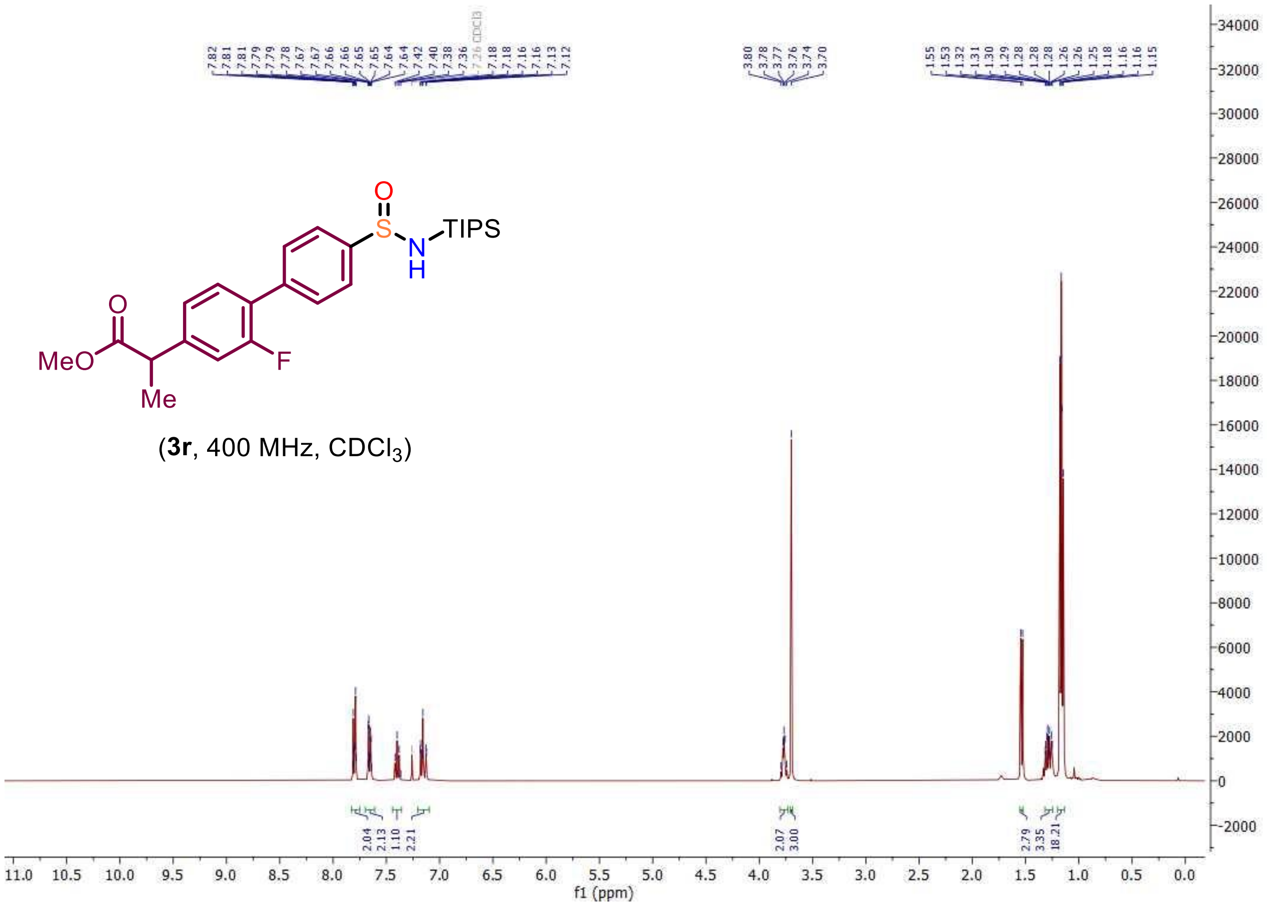

MeO
O
Me
F
S
O
N
H
TIPS
(3r, 400 MHz, CDCl3)
f1 (ppm)

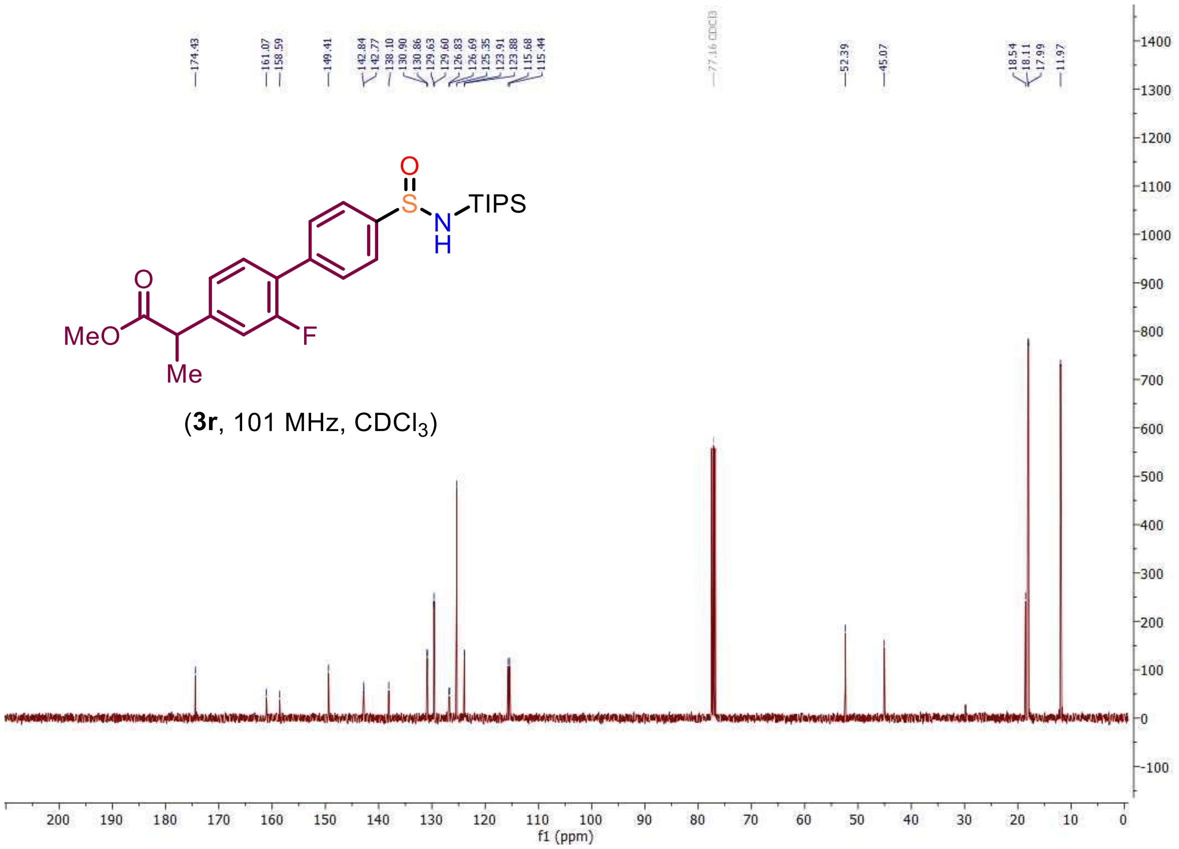

MeO
O
Me
F
S
O
N
H
TIPS
(3r, 101 MHz, CDCl3)
f1 (ppm)

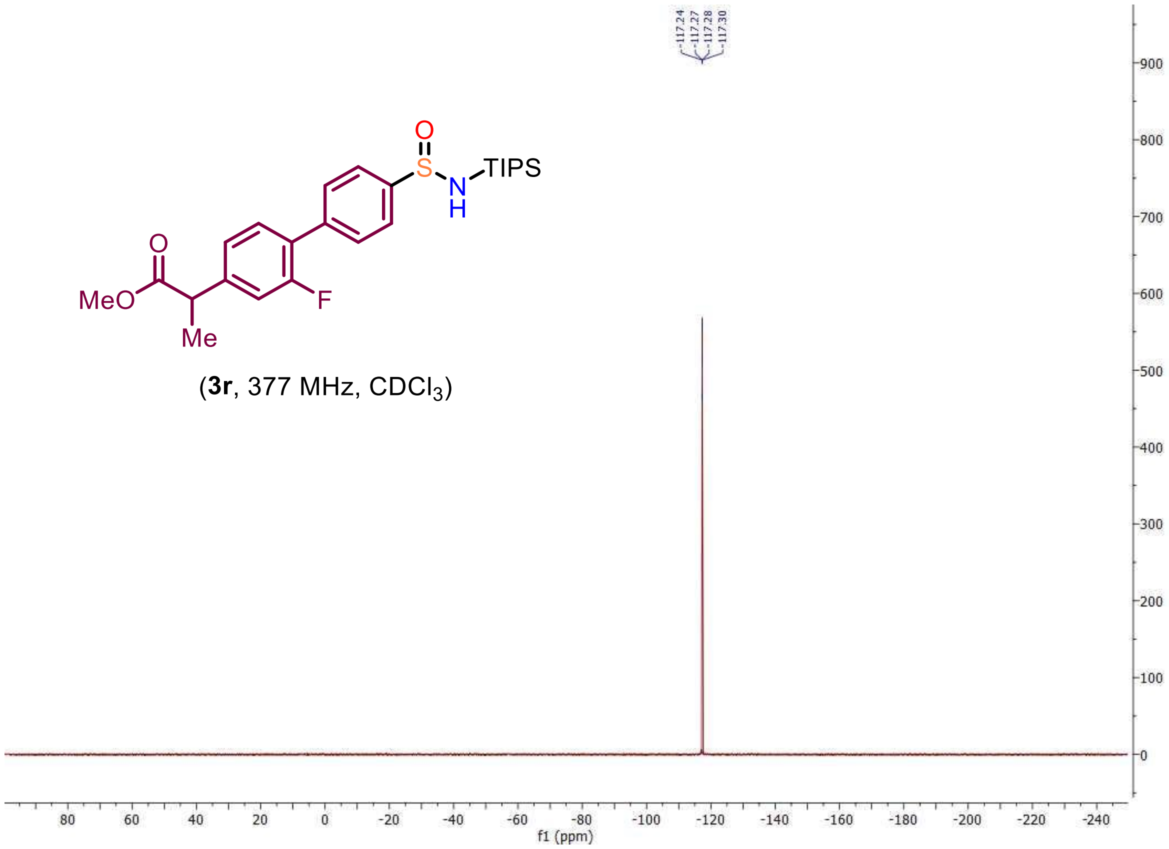

($\mathbf{3r}$, 377 MHz, $CDCl_3$)

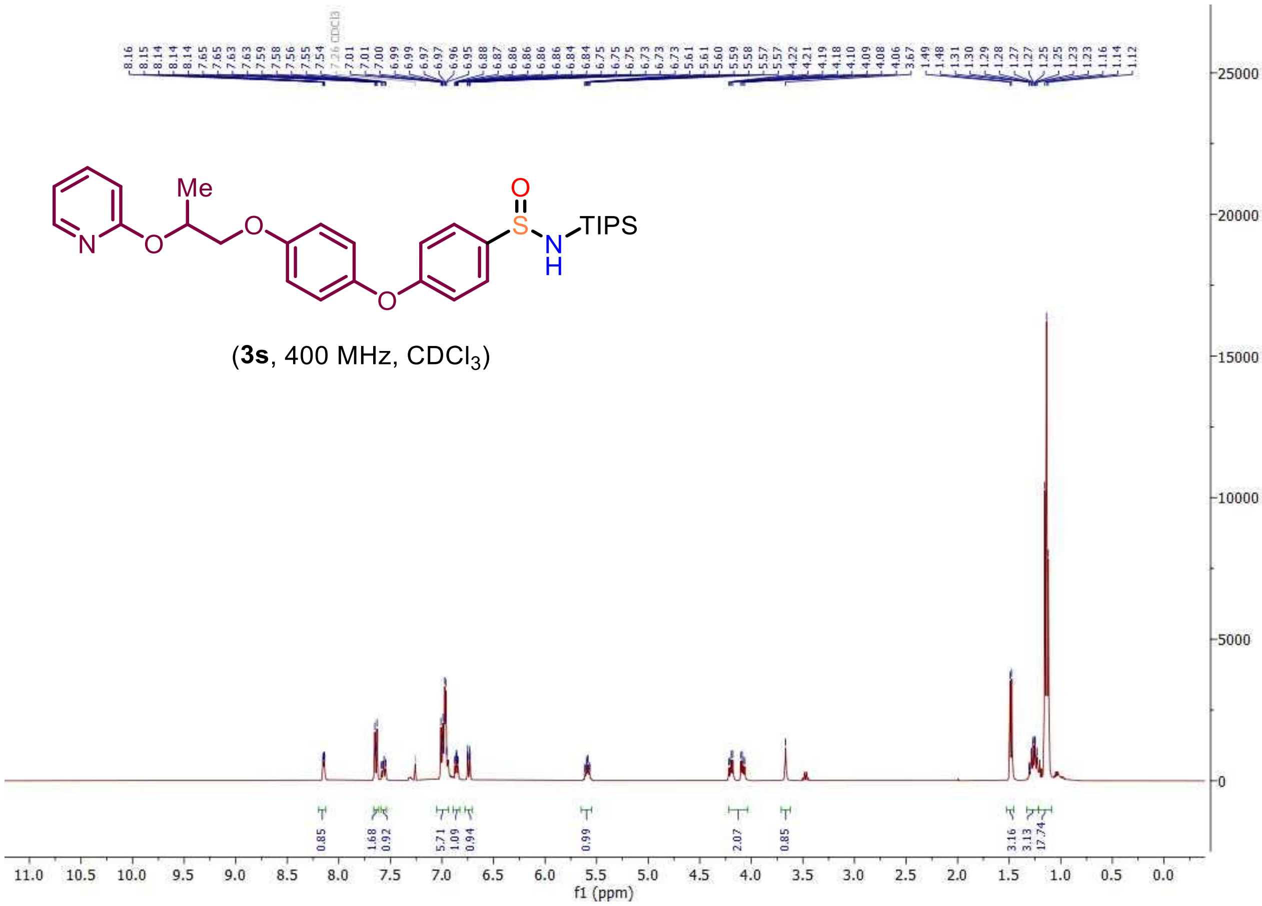

($\mathbf{3s}$, 400 MHz, $CDCl_3$)

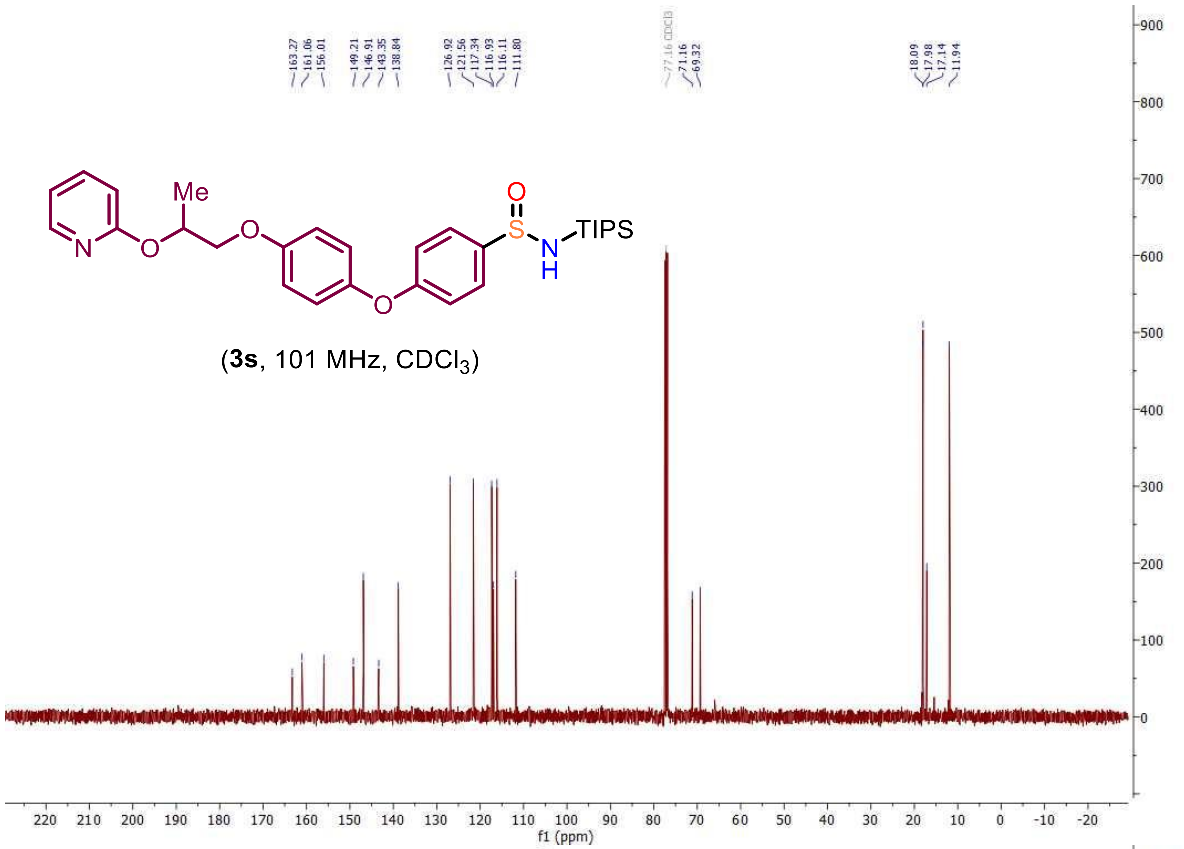

(**3s**, 101 MHz, $CDCl_3$)

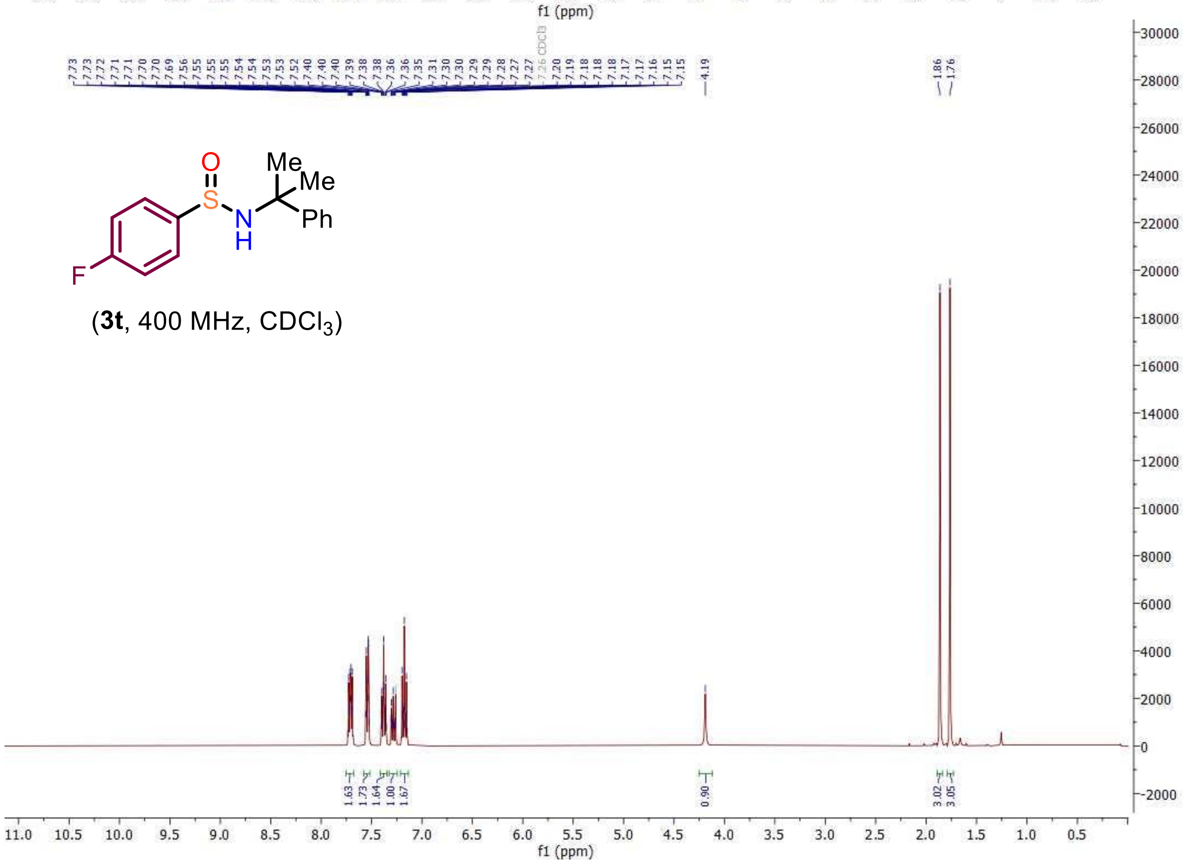

(**3t**, 400 MHz, $CDCl_3$)

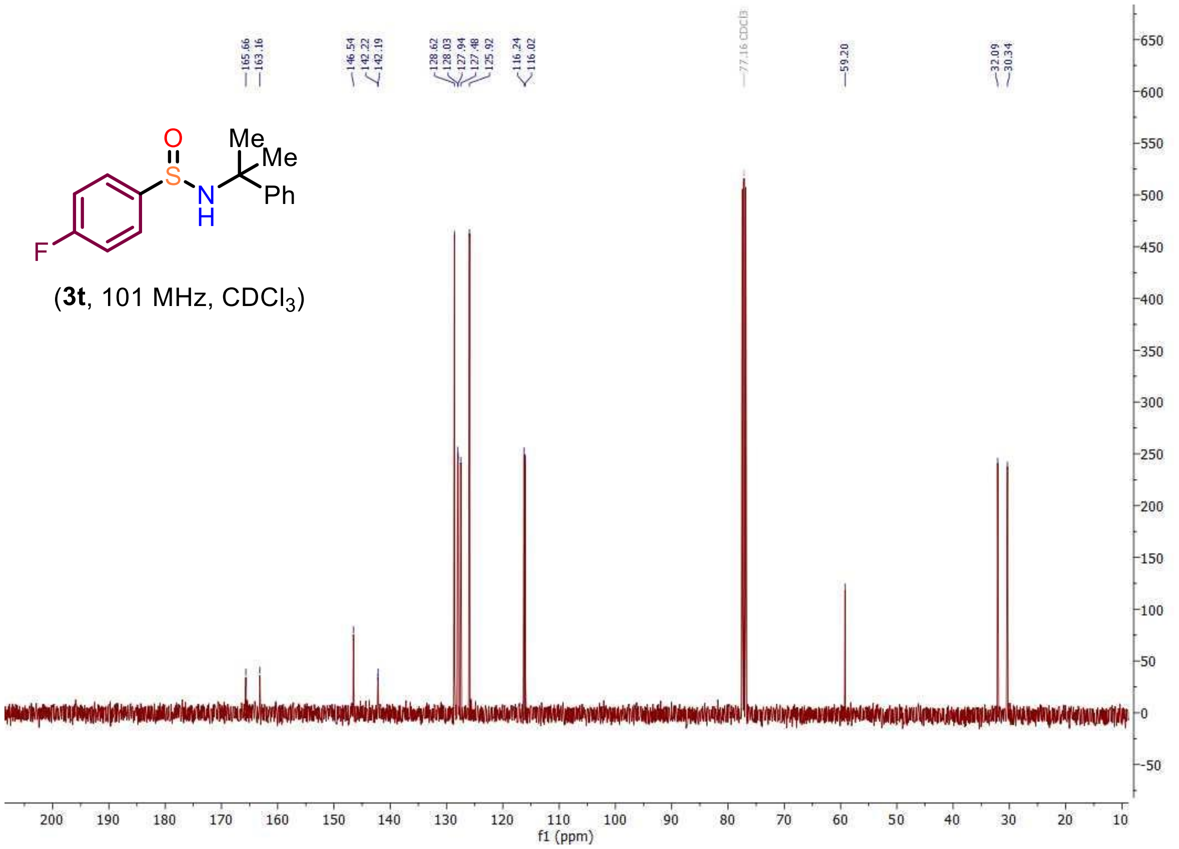

(**3t**, 101 MHz, $CDCl_3$)

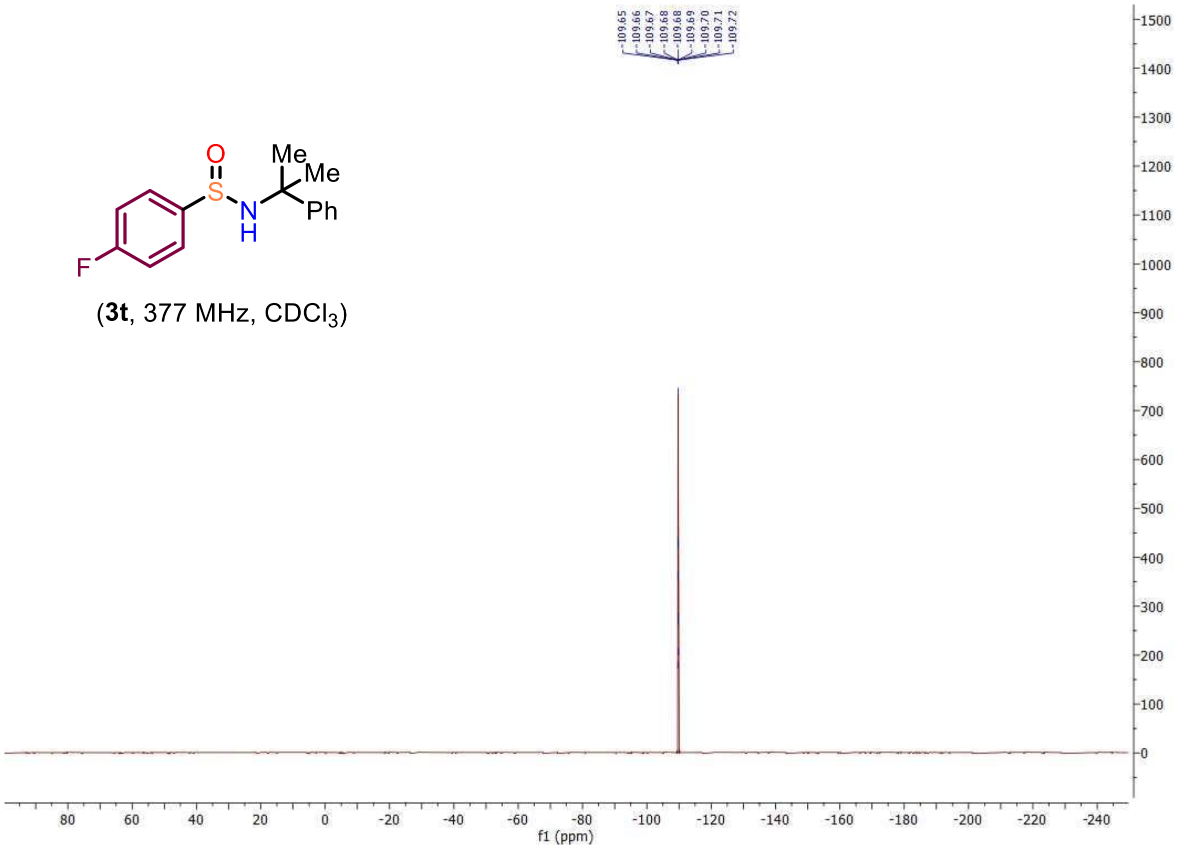

(**3t**, 377 MHz, $CDCl_3$)

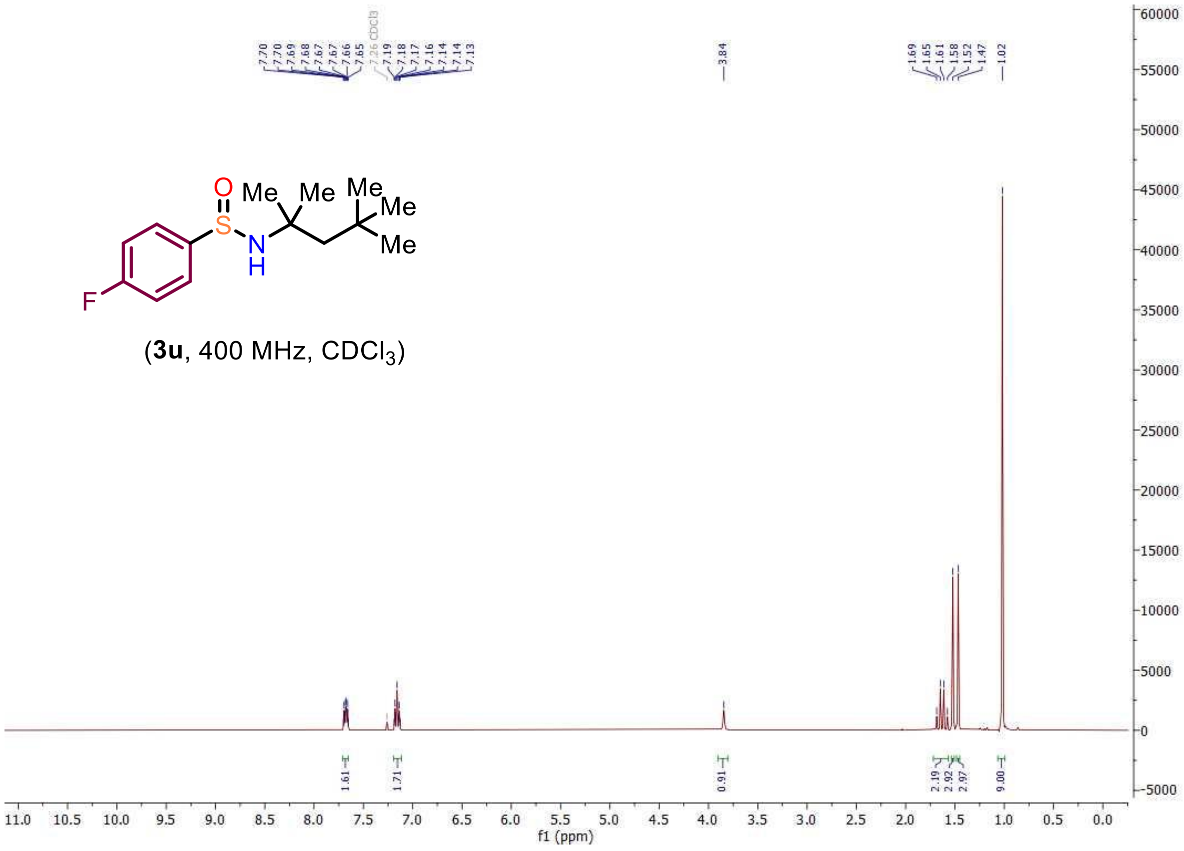
(3u, 400 MHz, CDCl3)
f1 (ppm)

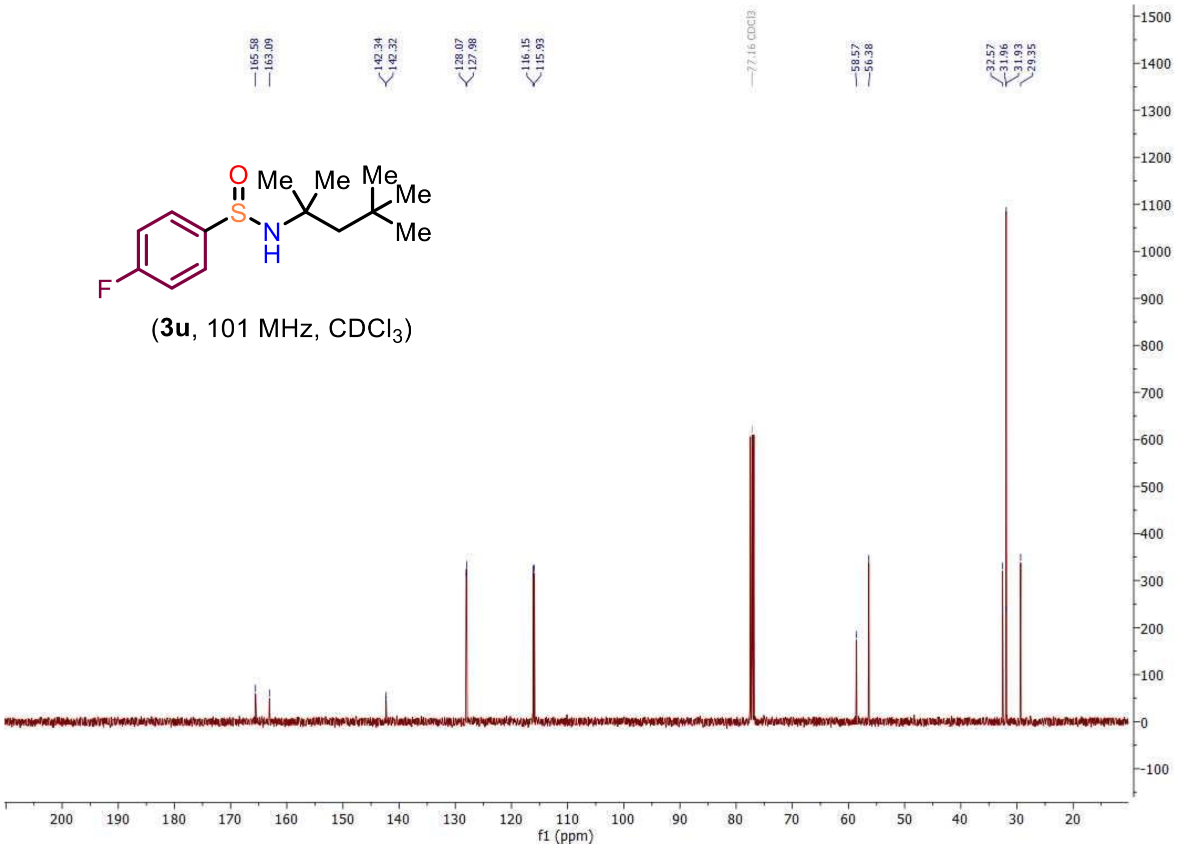
(3u, 101 MHz, CDCl3)
f1 (ppm)

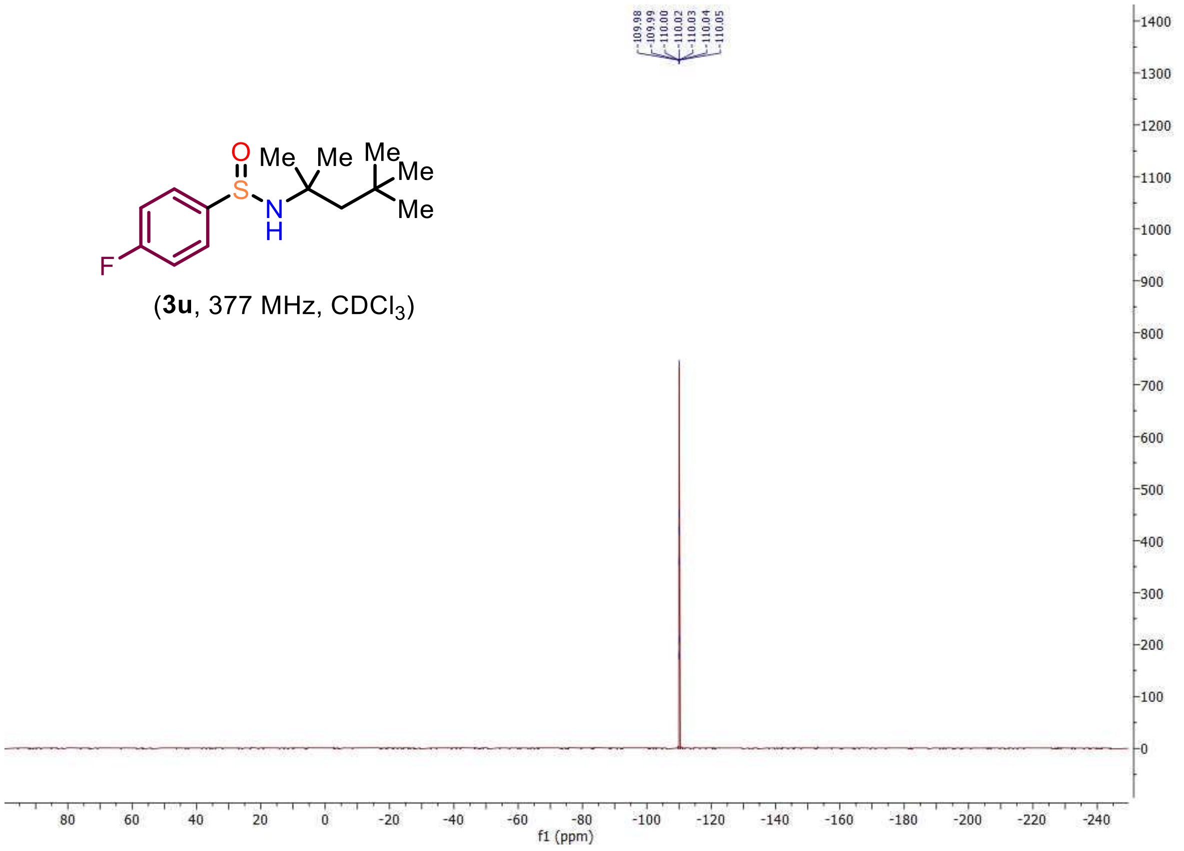
-109.98
-109.99
-110.00
-110.02
-110.03
-110.04
-110.05
(3u, 377 MHz, CDCl3)
f1 (ppm)

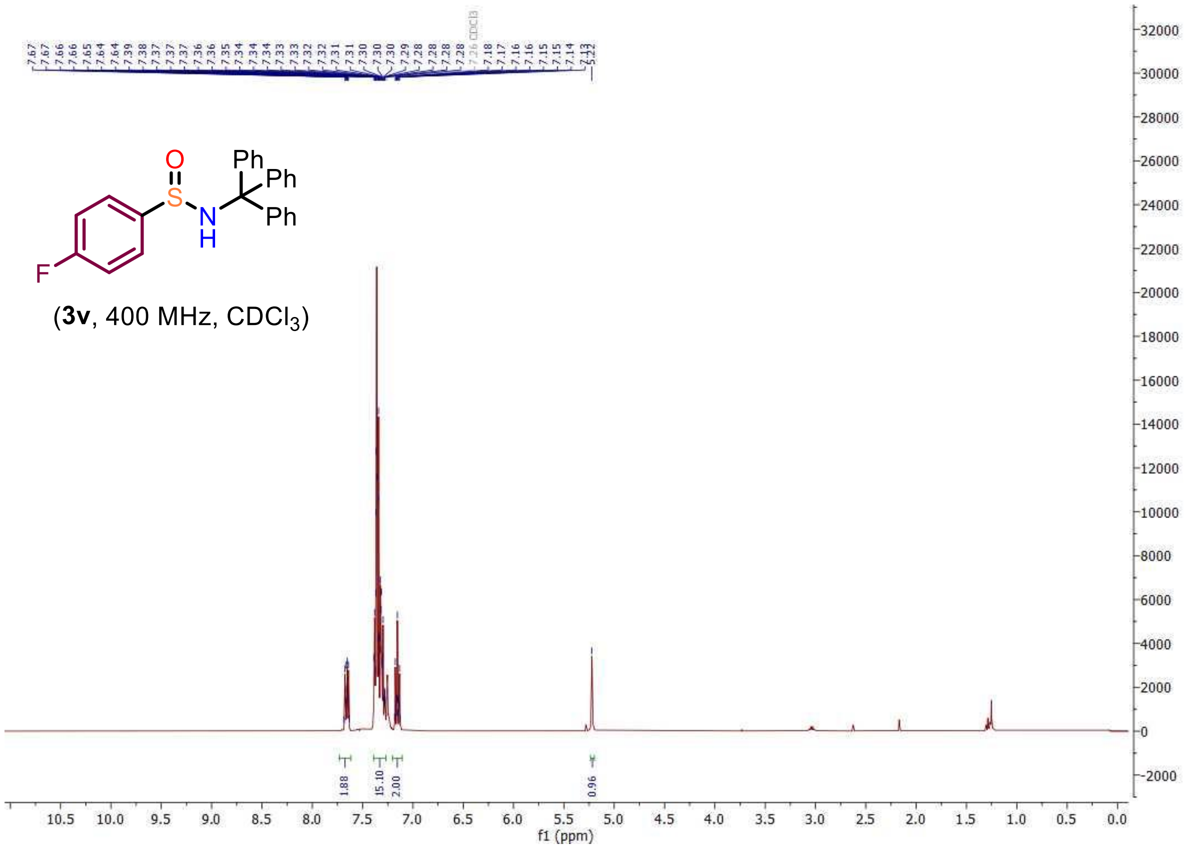
CDCl3
(3v, 400 MHz, CDCl3)
1.88
15.10
2.00
0.96
f1 (ppm)

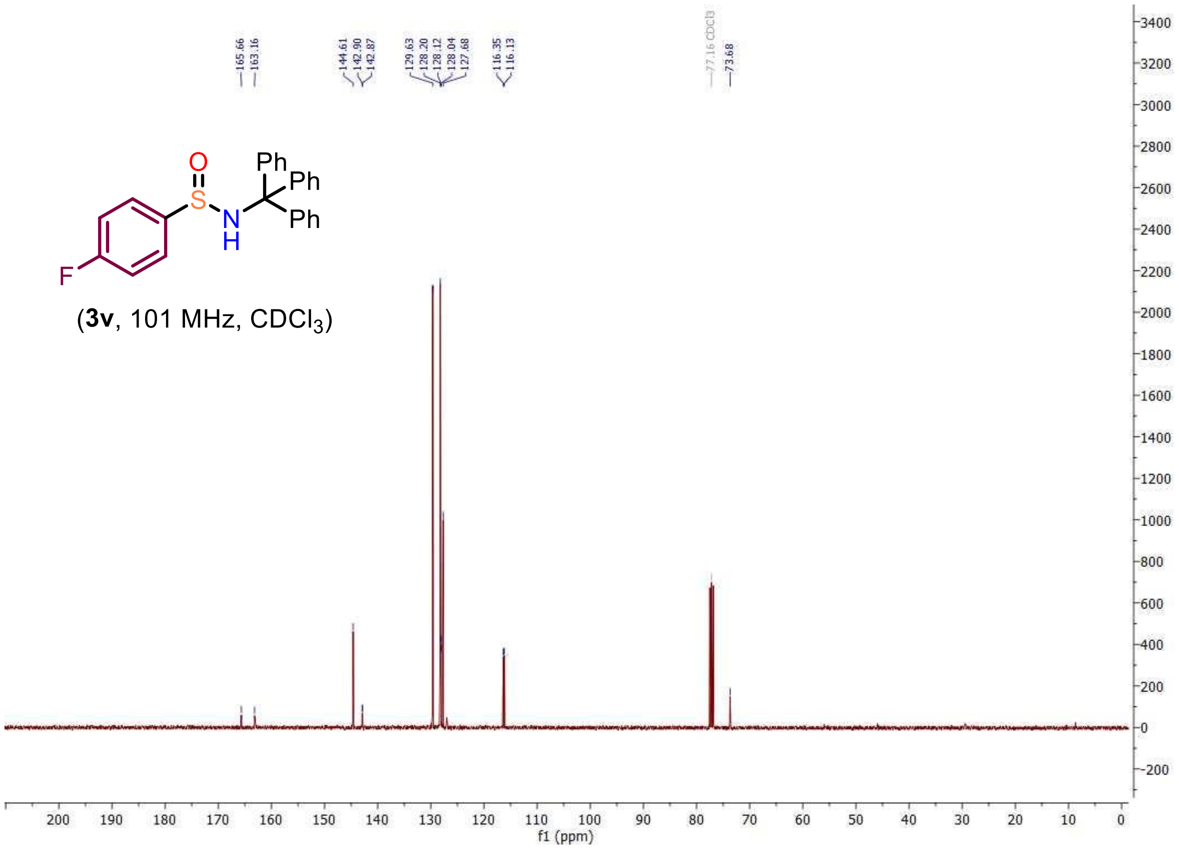

165.66
163.16
144.61
142.90
142.87
129.63
128.20
128.12
128.04
127.68
116.35
116.13
77.16 CDCl3
73.68
(3v, 101 MHz, CDCl3)
f1 (ppm)

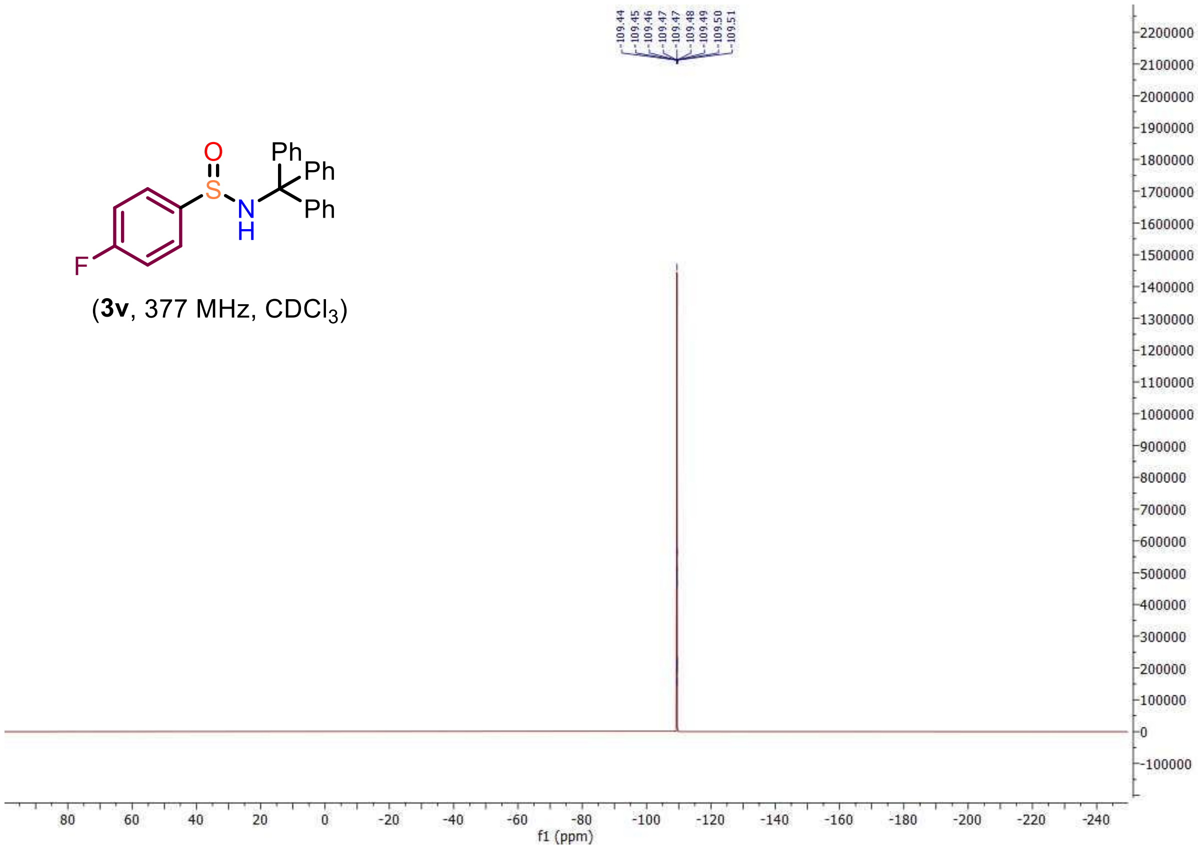

-109.44
-109.45
-109.46
-109.47
-109.47
-109.48
-109.49
-109.50
-109.51
(3v, 377 MHz, CDCl3)
f1 (ppm)

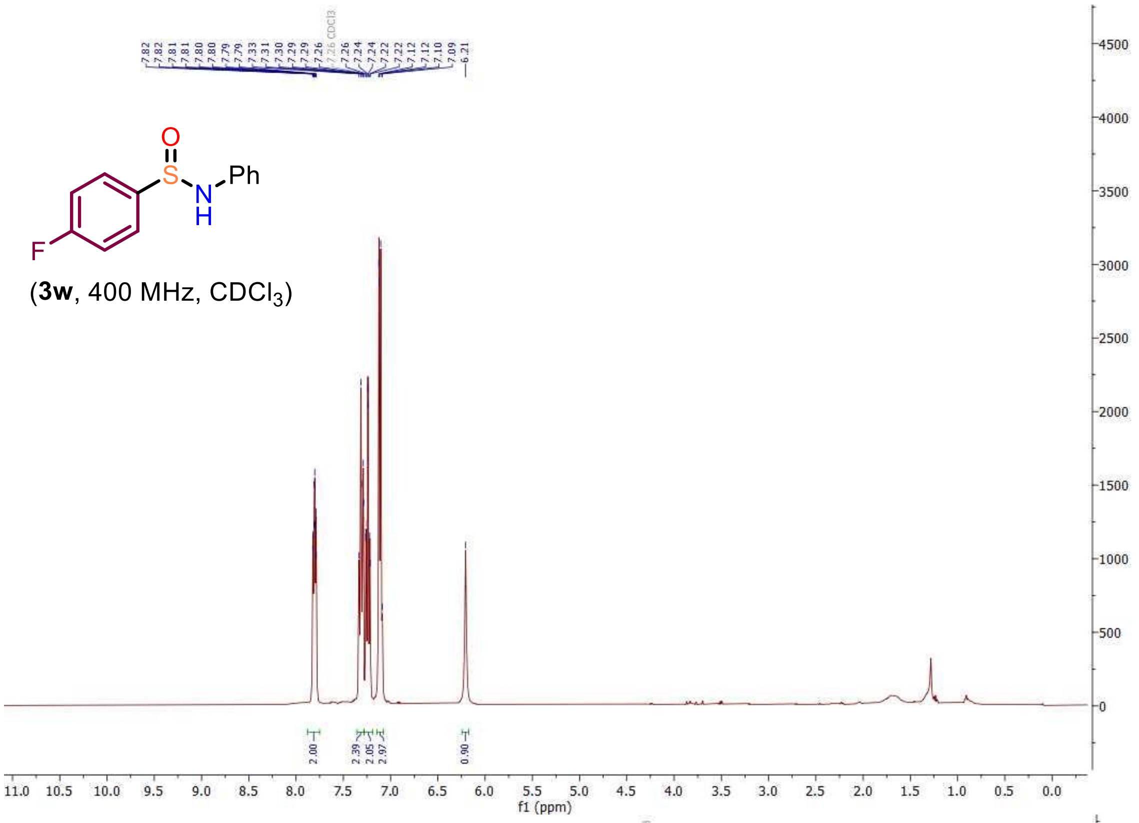
Ph
N
H
O
S
F
(3w, 400 MHz, CDCl3)
7.82 7.82 7.81 7.81 7.80 7.80 7.79 7.79 7.33 7.31 7.30 7.29 7.29 7.29 7.26 7.26 CDCl3 7.26 7.24 7.24 7.22 7.22 7.12 7.12 7.10 7.09 6.21
2.00
2.39
2.05
2.97
0.90
f1 (ppm)

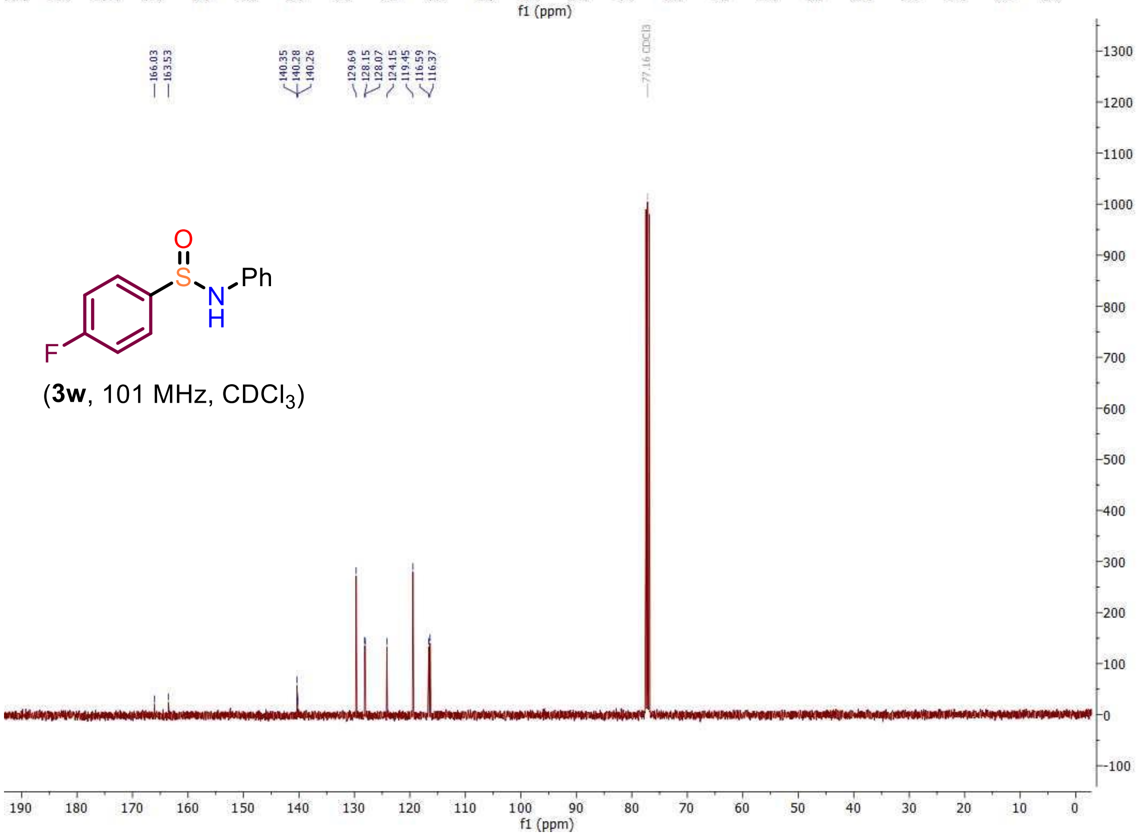
166.03
163.53
140.35
140.28
140.26
129.69
128.15
128.07
124.15
119.45
116.59
116.37
77.16 CDCl3
Ph
N
H
O
S
F
(3w, 101 MHz, CDCl3)
f1 (ppm)

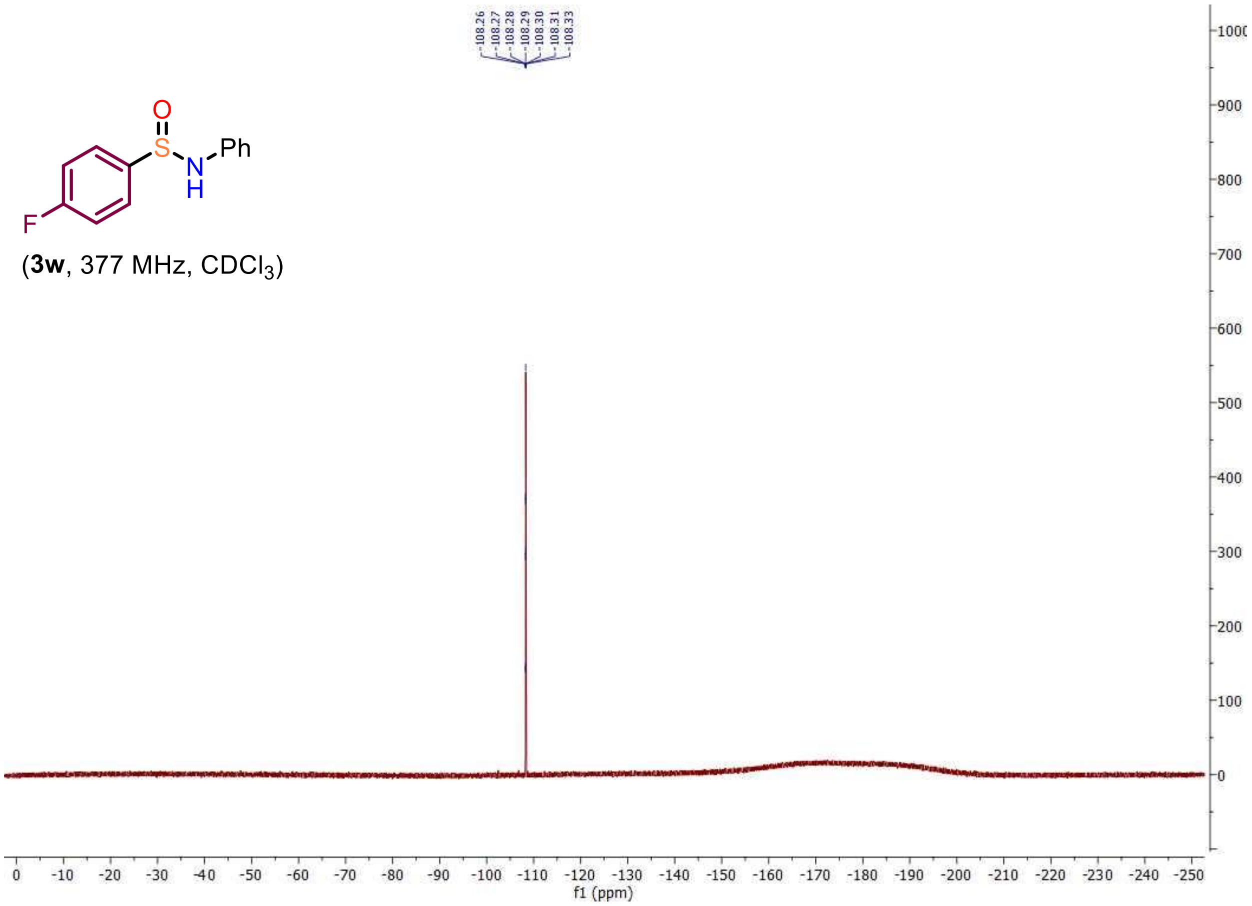

(**3w**, 377 MHz, $CDCl_3$)

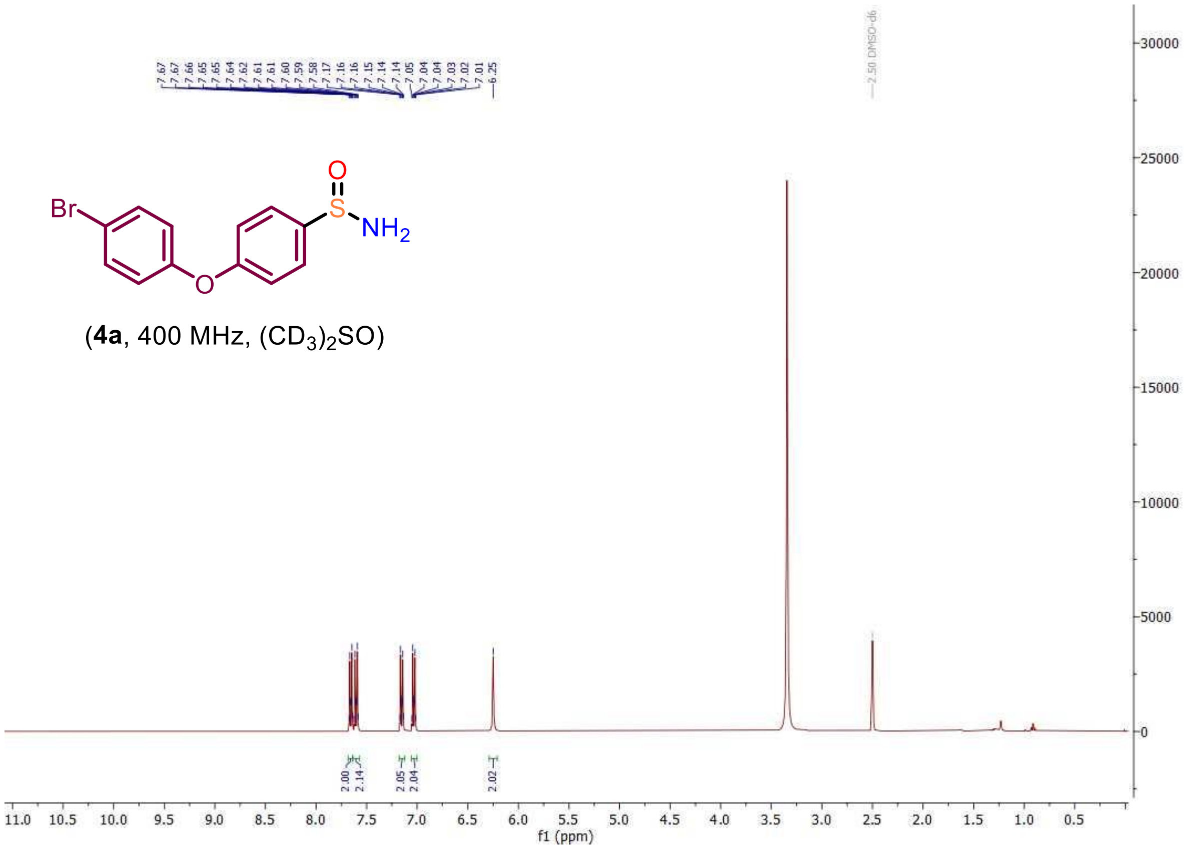

(**4a**, 400 MHz, $(CD_3)_2SO$)

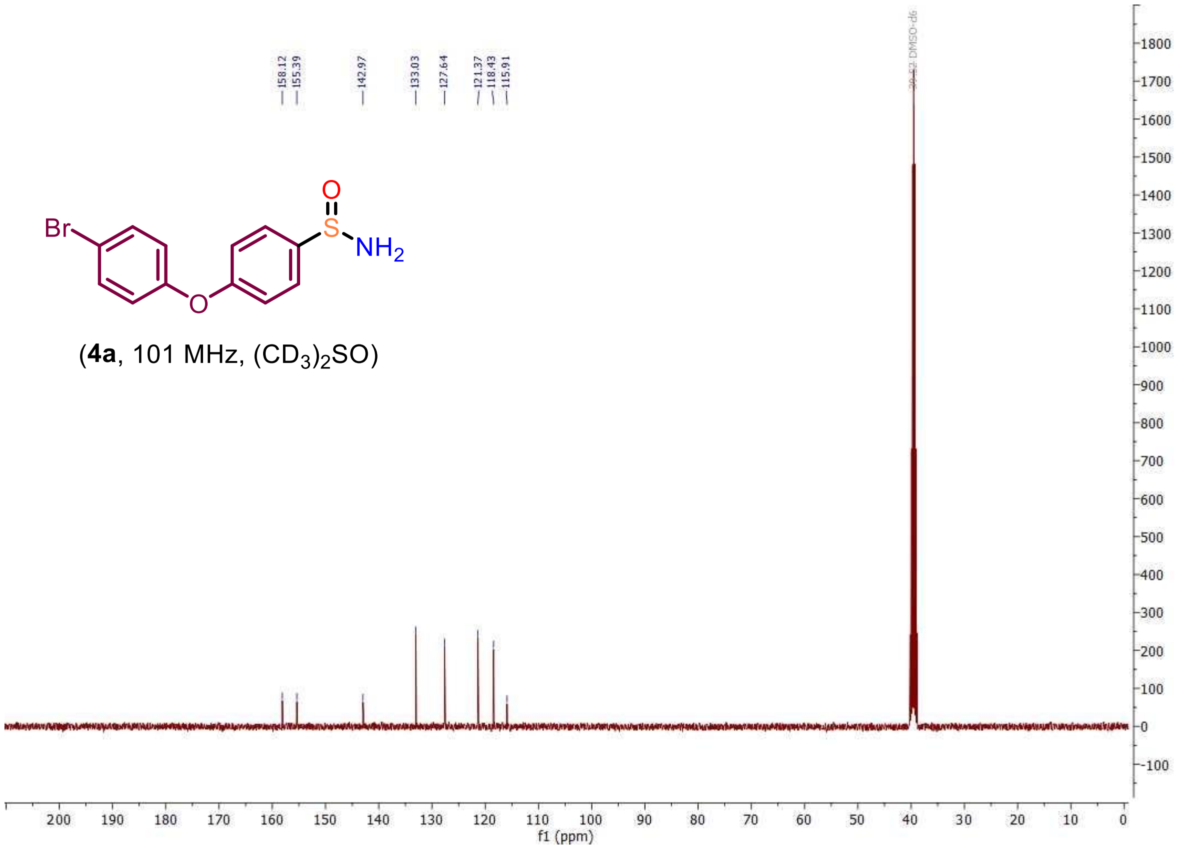
158.12
155.39
142.97
133.03
127.64
121.37
118.43
115.91
39.52 DMSO-d6
Br
O
S
NH2
O
(4a, 101 MHz, (CD3)2SO)
f1 (ppm)

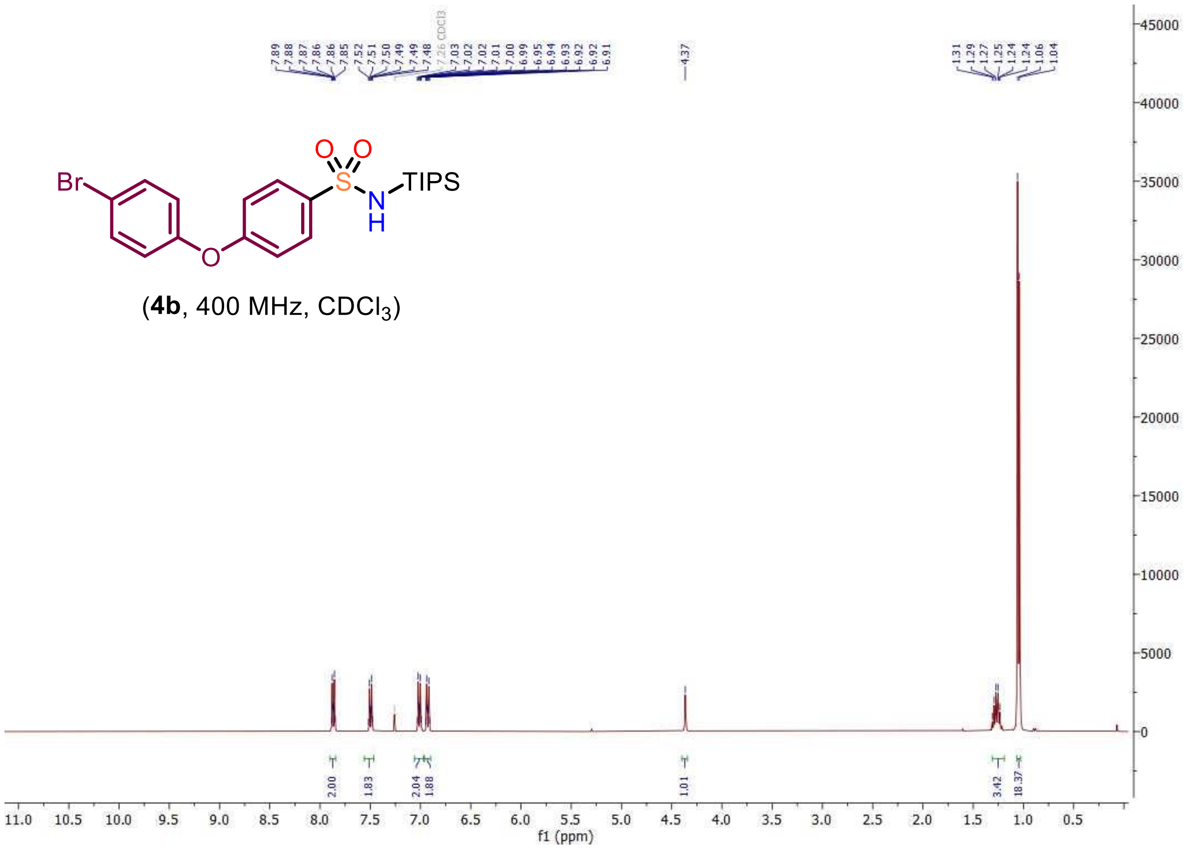
4.37
7.26 CDCl3
Br
O
O O
S
N
H
TIPS
(4b, 400 MHz, CDCl3)
2.00
1.83
2.04
1.88
1.01
3.42
18.37
f1 (ppm)

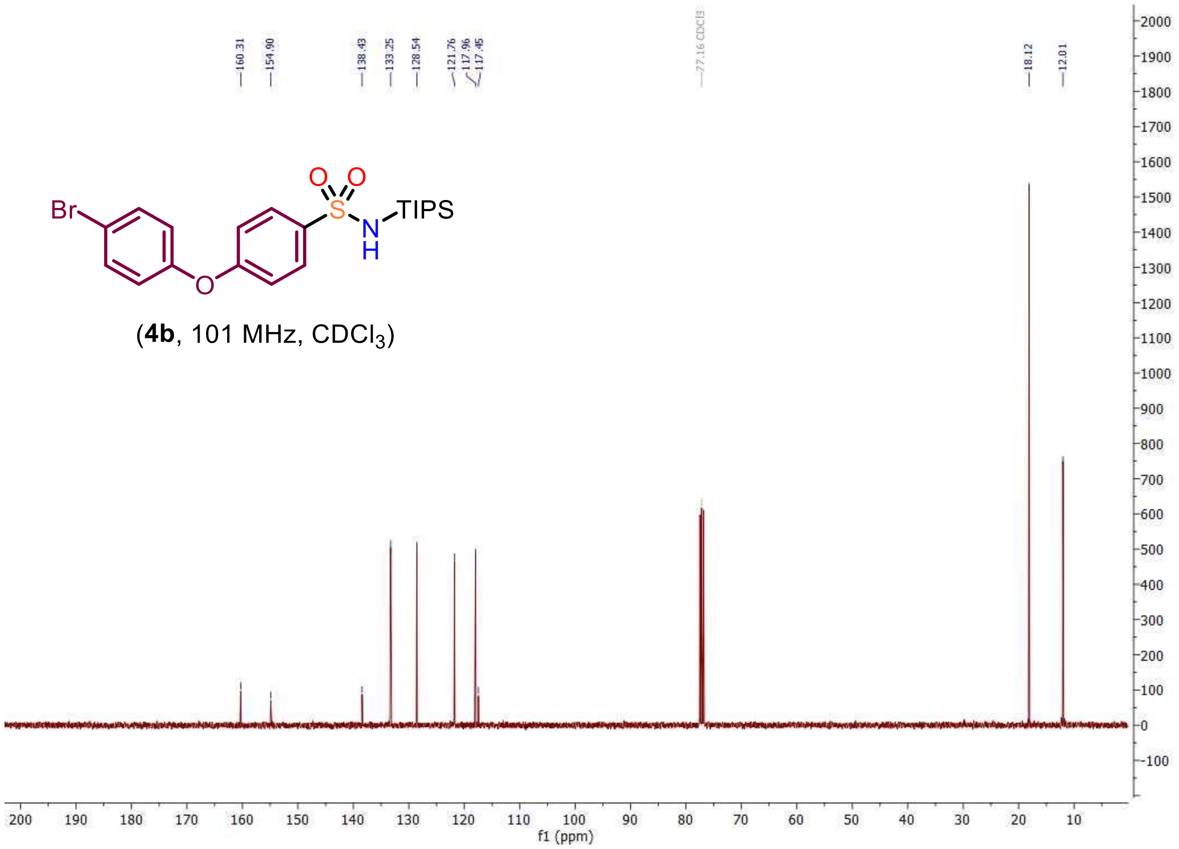

(**4b**, 101 MHz, $CDCl_3$)

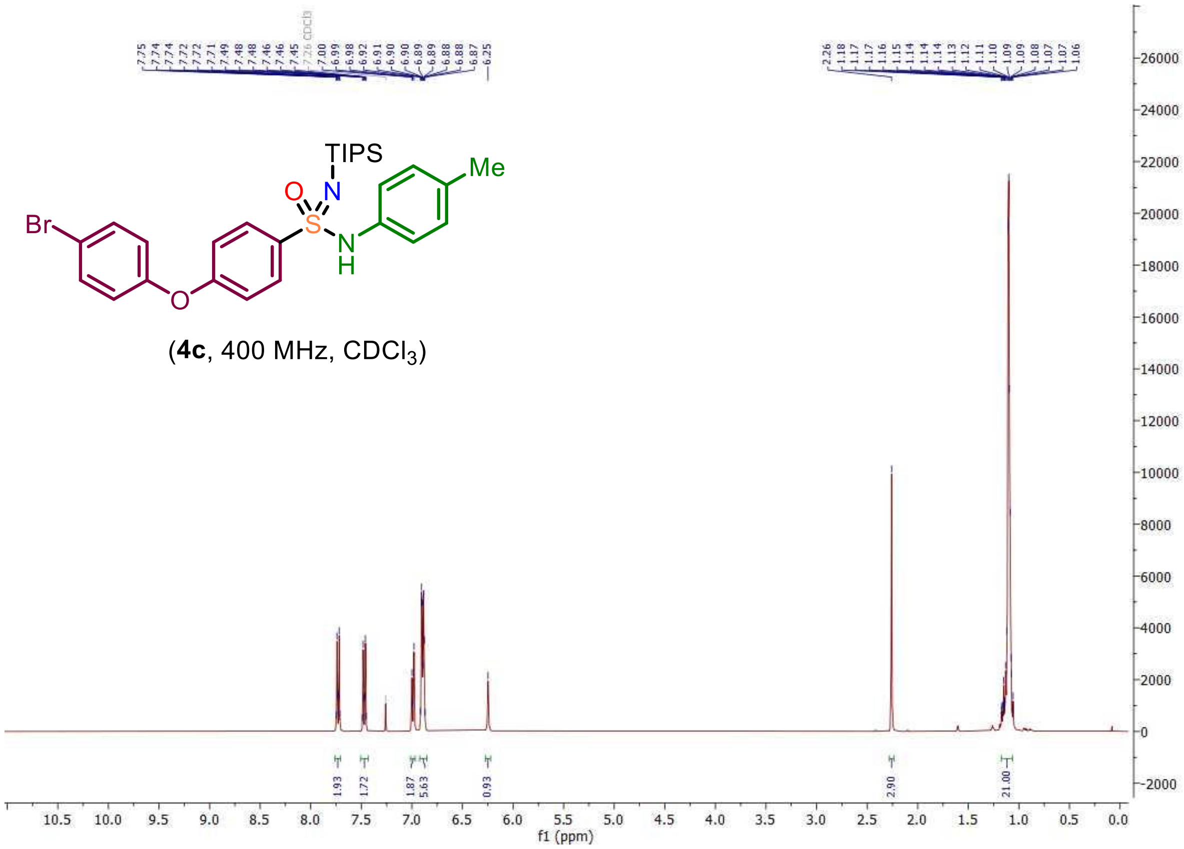

(**4c**, 400 MHz, $CDCl_3$)

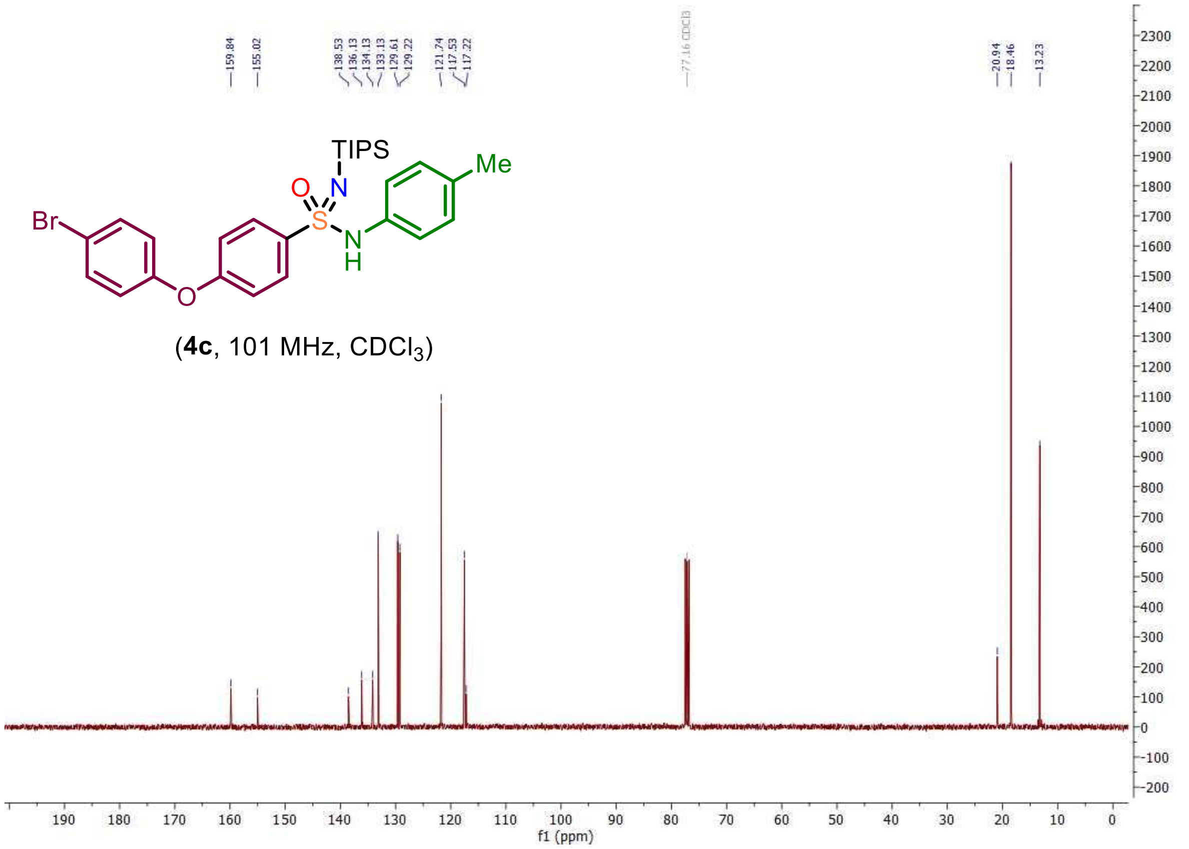

(**4c**, 101 MHz, $CDCl_3$)

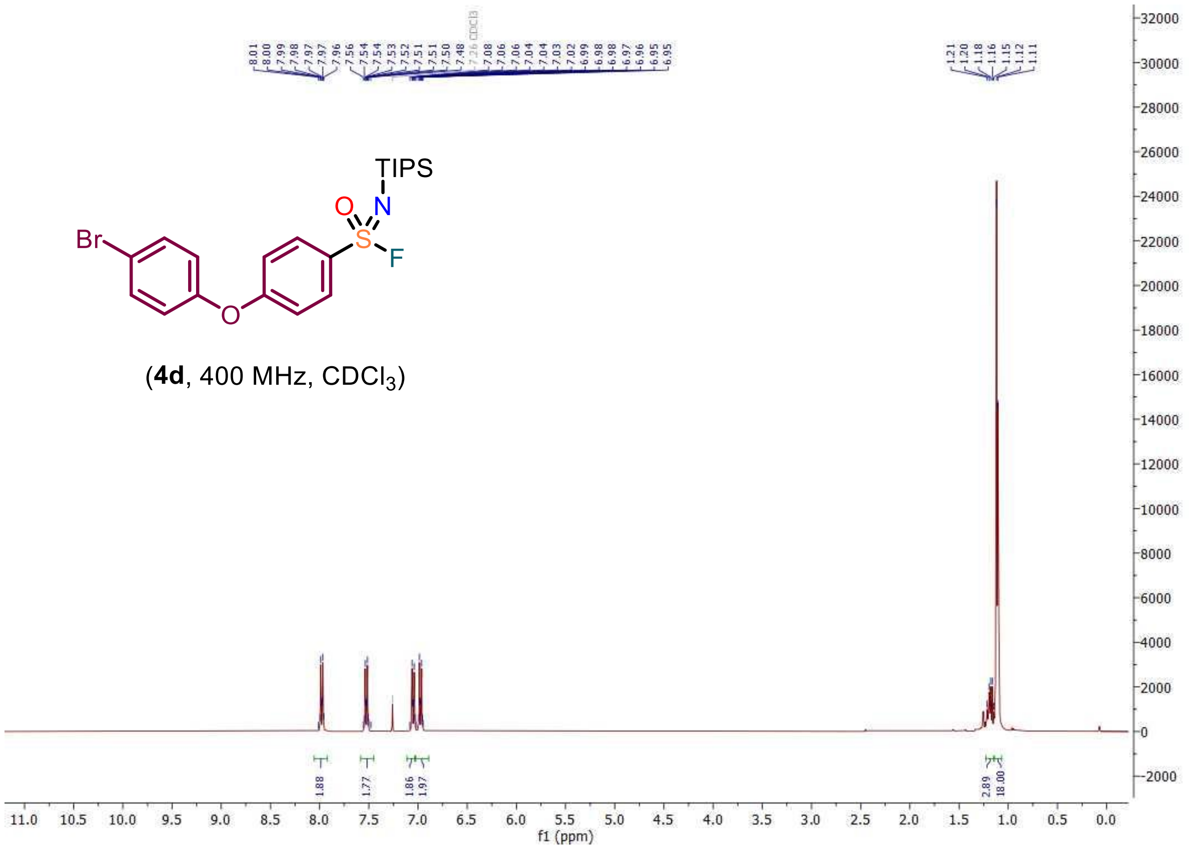

(**4d**, 400 MHz, $CDCl_3$)

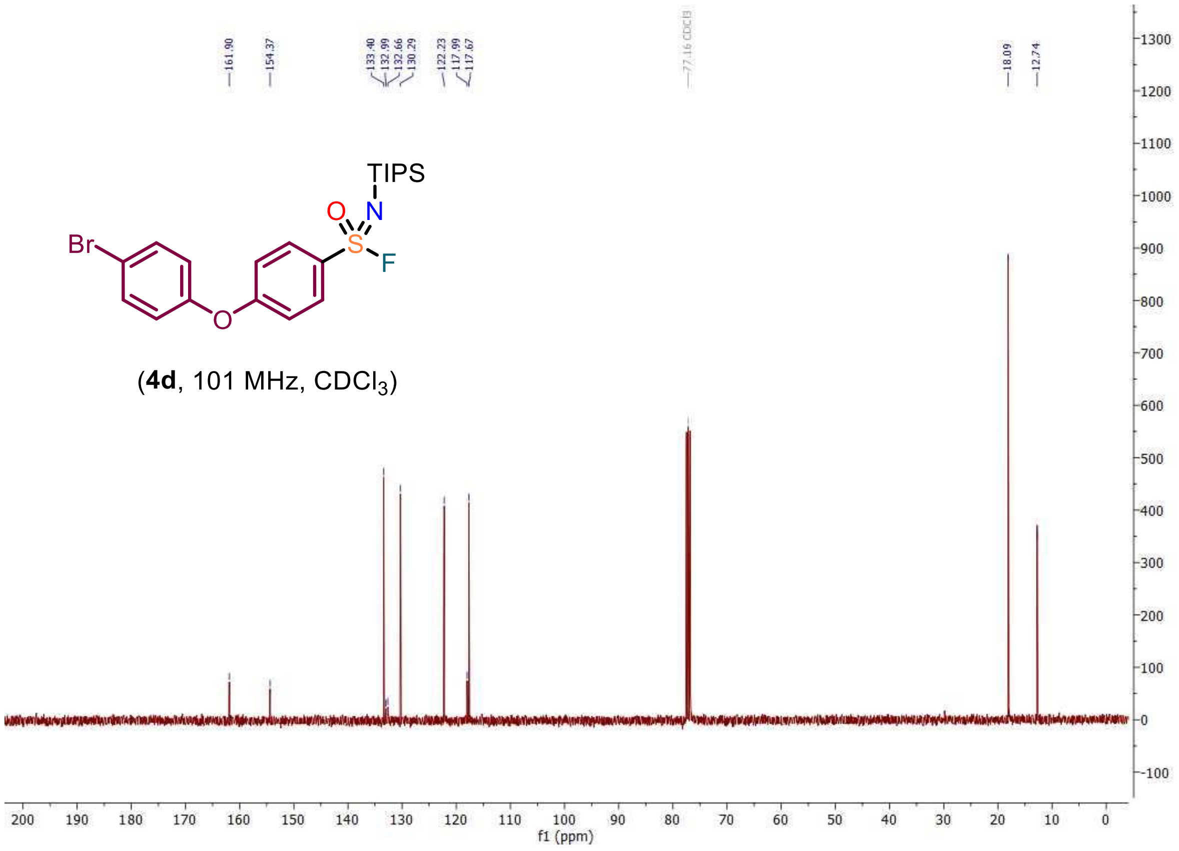

(**4d**, 101 MHz, $CDCl_3$)

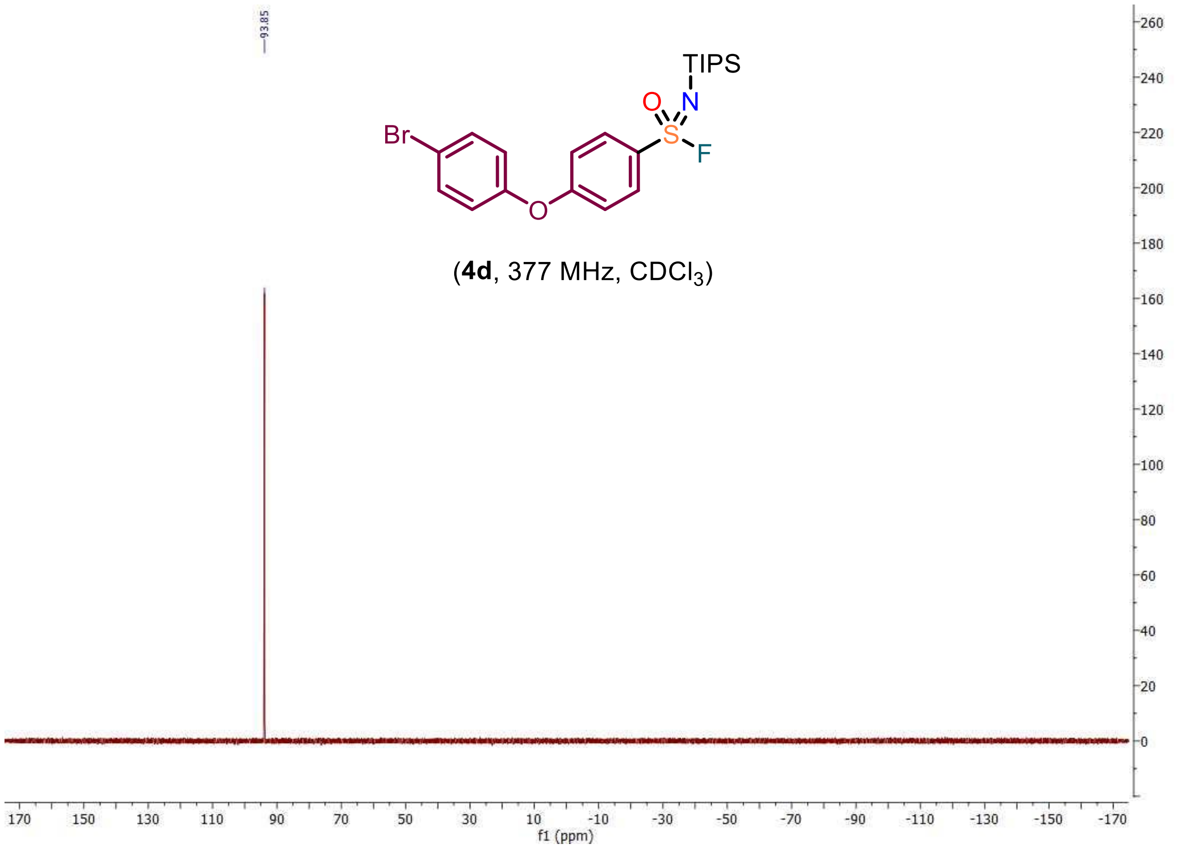

(**4d**, 377 MHz, $CDCl_3$)

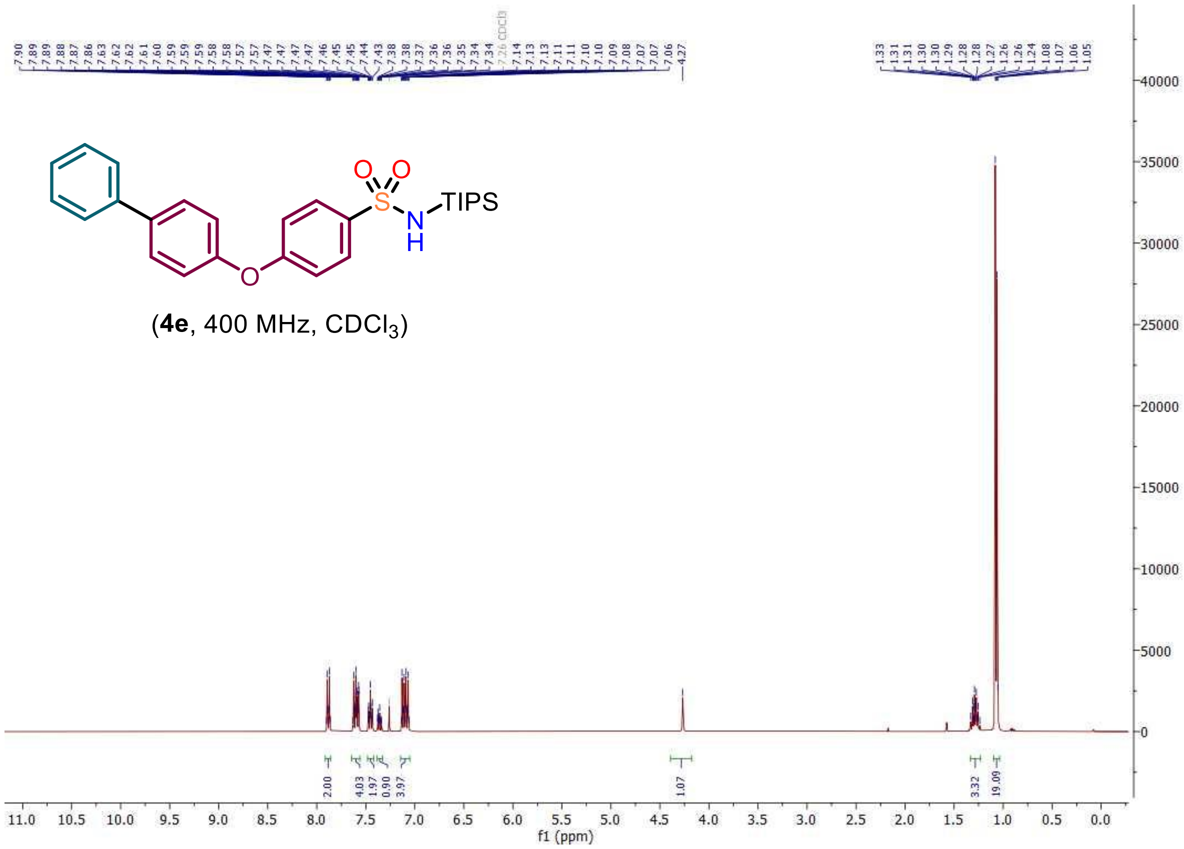
TIPS
(4e, 400 MHz, CDCl3)
f1 (ppm)

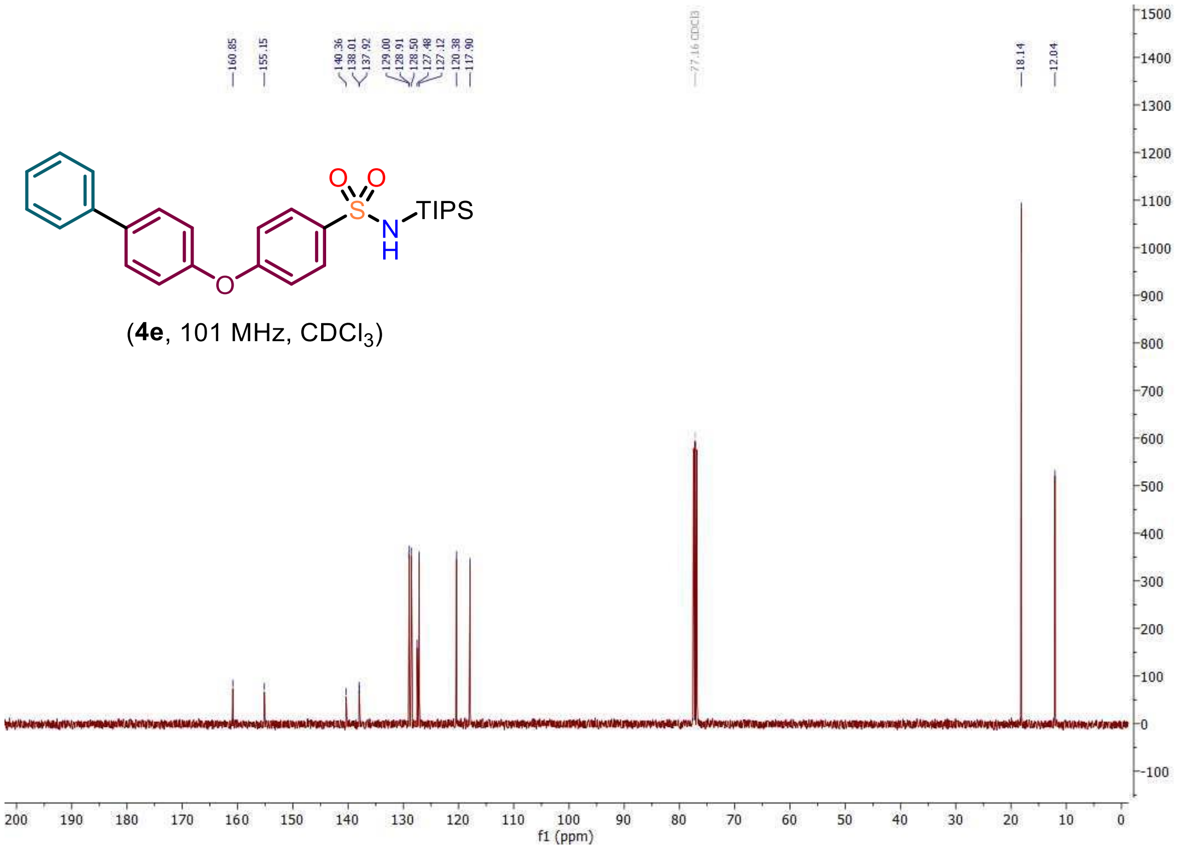
TIPS
(4e, 101 MHz, CDCl3)
f1 (ppm)
